# A holistic platform for accelerating sorbent-based carbon capture

**DOI:** 10.1038/s41586-024-07683-8

**Published:** 2024-07-17

**Authors:** Charithea Charalambous, Elias Moubarak, Johannes Schilling, Eva Sanchez Fernandez, Jin-Yu Wang, Laura Herraiz, Fergus Mcilwaine, Shing Bo Peh, Matthew Garvin, Kevin Maik Jablonka, Seyed Mohamad Moosavi, Joren Van Herck, Aysu Yurdusen Ozturk, Alireza Pourghaderi, Ah-Young Song, Georges Mouchaham, Christian Serre, Jeffrey A. Reimer, André Bardow, Berend Smit, Susana Garcia

**Affiliations:** 1 The Research Centre for Carbon Solutions (RCCS), School of Engineering and Physical Sciences, Heriot-Watt University, Edinburgh, UK; 2 Laboratory of Molecular Simulation (LSMO), Institut des Sciences et Ingénierie Chimiques, École Polytechnique Fédérale de Lausanne (EPFL), Sion, Switzerland; 3 Laboratory of Energy and Process Systems Engineering (EPSE), ETH Zurich, Zurich, Switzerland; 4 Solverlo Ltd, Dunbar, UK; 5 Institut des Matériaux Poreux de Paris, Ecole Normale Supérieure, ESPCI Paris, CNRS, PSL University, Paris, France; 6 Materials Science Division, Lawrence Berkeley National Laboratory, Berkeley, CA USA; 7 Department of Chemical and Biomolecular Engineering, University of California Berkeley, Berkeley, CA USA

**Keywords:** Carbon capture and storage, Chemical engineering, Metal-organic frameworks, Materials for energy and catalysis

## Abstract

Reducing carbon dioxide (CO_2_) emissions urgently requires the large-scale deployment of carbon-capture technologies. These technologies must separate CO_2_ from various sources and deliver it to different sinks^1,2^. The quest for optimal solutions for specific source–sink pairs is a complex, multi-objective challenge involving multiple stakeholders and depends on social, economic and regional contexts. Currently, research follows a sequential approach: chemists focus on materials design^3^ and engineers on optimizing processes^4,5^, which are then operated at a scale that impacts the economy and the environment. Assessing these impacts, such as the greenhouse gas emissions over the plant’s lifetime, is typically one of the final steps^6^. Here we introduce the PrISMa (Process-Informed design of tailor-made Sorbent Materials) platform, which integrates materials, process design, techno-economics and life-cycle assessment. We compare more than 60 case studies capturing CO_2_ from various sources in 5 global regions using different technologies. The platform simultaneously informs various stakeholders about the cost-effectiveness of technologies, process configurations and locations, reveals the molecular characteristics of the top-performing sorbents, and provides insights on environmental impacts, co-benefits and trade-offs. By uniting stakeholders at an early research stage, PrISMa accelerates carbon-capture technology development during this critical period as we aim for a net-zero world.

## Abstract (web summary)

A framework that integrates materials, process design, techno-economics and life-cycle assessment can be used to accelerate the development of carbon-capture technology as we aim for a net-zero world.

## Main

Solid adsorbent-based carbon capture can leverage modern reticular chemistry to synthesize millions of possible adsorbents^7^, including around 100,000 metal–organic frameworks (MOFs)^8,9^. To fully explore this potential, we must move beyond the conventional sequential, time-consuming trial-and-error approach. Computational groups have initiated material genomics to accelerate discovery, generating materials in silico and predicting their adsorption properties through molecular simulations^10–13^. Although these predictions are promising, their impact has been limited because they often assume that a few basic adsorption properties (for example, Henry selectivity or carbon dioxide (CO_2_) capacity) suffice to evaluate material’s performance. The optimal material depends on specific process requirements, scale-up, location and life-cycle assessment (LCA)^4,5,14–20^. The lack of system-level contextualization has hindered stakeholder engagement in materials discovery. A holistic approach is needed to link material properties with process design and techno-economic analysis (TEA). An LCA further evaluates environmental impacts beyond climate change, ensuring that the carbon-capture plant’s construction and operation do not result in higher CO_2_-equivalent (CO_2_e) emissions than it mitigates over its lifetime^21^.

## The PrISMa platform for carbon capture

**Fig. 1:**
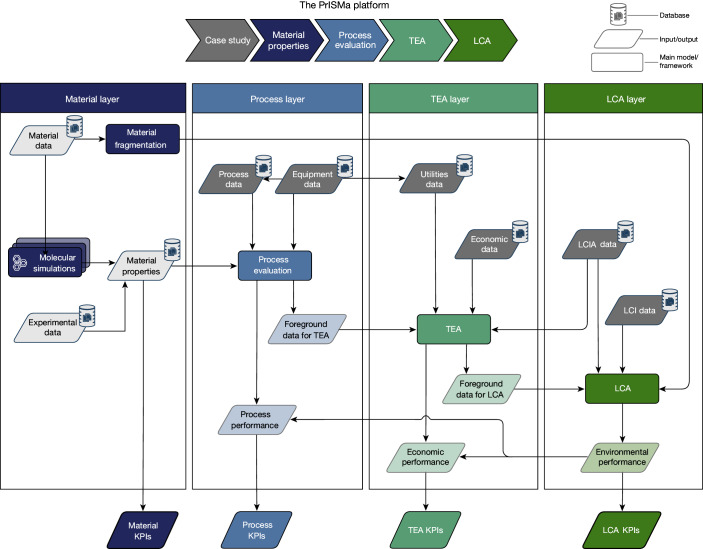
The PrISMa platform screens solid sorbents for CO_2_-capture applications. The data flowchart of the four layers of the platform. The figure illustrates the links between the LCA, the TEA, process evaluation and material characterization. On the basis of the crystal structure of a sorbent material, we evaluate its performance for a specific carbon-capture process, connecting a CO_2_ source with a CO_2_ sink in a region of the world, using a total of 50 KPIs. The platform integrates databases on material properties, process design parameters, utilities data, economic data, life-cycle impact assessment (LCIA) data and life-cycle inventory (LCI) data. A detailed description of the methods used in each layer can be found in Supplementary Information Section 3. Our interactive visualization tool provides access to all KPIs for all case studies and more than 1,200 materials^25^.

The PrISMa (Process-Informed design of tailor-made Sorbent Materials) platform (Fig. 1) allows for the interrogation and screening of materials for a given case study, which is defined by the CO_2_ source, the destination of the CO_2_ (sink), the capture technology, the available utilities and the geographical region (Extended Data Table 1). In the materials layer, we use experimental data or crystal structures to predict the adsorption thermodynamics of flue gas components (CO_2_, nitrogen (N_2_) and water (H_2_O)) through molecular simulations. These thermodynamic data and process and equipment data serve as input for the process layer, where we compute parameters such as purity, recovery, productivity and energy requirements. In the TEA layer, we assess the economic and technical viability. The LCA layer then evaluates the environmental impacts over the plant’s lifetime.

Following this holistic approach, the platform identifies top-performing materials for further study. These materials can then undergo more detailed process modelling and investigation (for example, sorbent durability and manufacturing) to advance the technology to pilot and demonstration scales.

## Informing stakeholders’ perspectives

**Extended Data Fig. 1:**
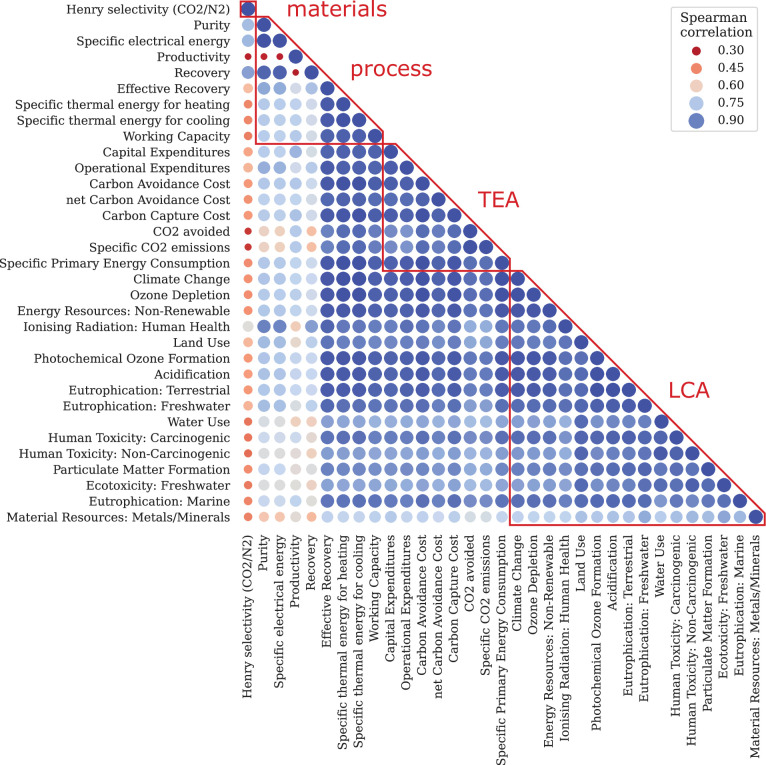
Spearman’s rank correlation matrix for the cement case in the UK with TVSA process at 0.6 bar. Spearman’s rank correlation matrix of the rankings considering one material KPI, eight process KPIs, eight TEA KPIs, and 16 LCA KPIs. Dark blue represents very strong correlations, while dark red represents lower correlations. The size of the circle is proportional to the absolute value of the correlation. The diagonal circles in the matrix have, by definition, a Spearman’s correlation coefficient of 1. A more detailed description can be found in Supplementary Information Section 7.

The PrISMa platform’s modular structure allows us to consider various stakeholders’ perspectives. For any combination of source, sink, technology, utilities and region, we compute a list of 50 key performance indicators (KPIs; Supplementary Table 5). A Spearman analysis (Extended Data Fig. 1) helped us identify six reference KPIs that capture the most important trends (Extended Data Table 2).

**Fig. 2:**
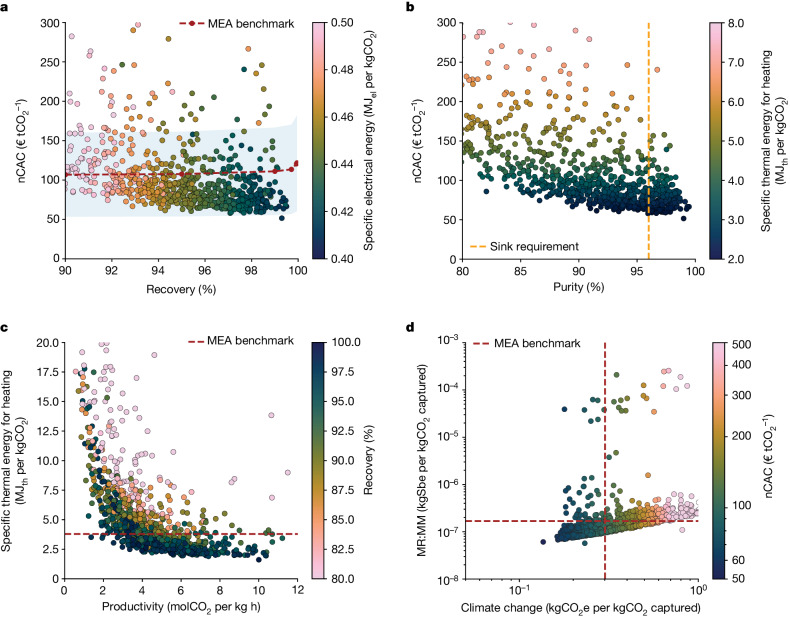
Materials performance for a TVSA carbon-capture process at 0.6 bar added to a cement plant in the UK. **a**, The nCAC versus recovery, with colour coding the specific electrical energy consumption. **b**, The nCAC versus purity, with colour coding the specific thermal energy consumption. **c**, Specific thermal energy consumption for heating versus productivity, with colour coding the recovery. **d**, MR:MM versus climate change, with colour coding the nCAC. Sbe, antimony equivalent. Our visualization tool^25^ gives an interactive version of this graph. The dotted lines in **a**, **c** and **d** show the MEA benchmark (Supplementary Information Section 4). In **b**, the vertical orange dotted line gives the purity required for geological storage (>96%) and in **a**, the blue shaded area gives the uncertainty. Each dot represents the corresponding KPI of a material.

Let us first focus on one case study: capturing CO_2_ using a temperature vacuum swing adsorption (TVSA) process (with a vacuum pressure of 0.6 bar) from a cement plant located in the UK. The captured CO_2_ is compressed and sent for geological storage. In Fig. 2, we compare the performance of the materials with the monoethanolamine (MEA) benchmark^22^ (Supplementary Information Section 4); many materials outperform the benchmark for the different process, TEA and LCA KPIs.

**Fig. 3:**
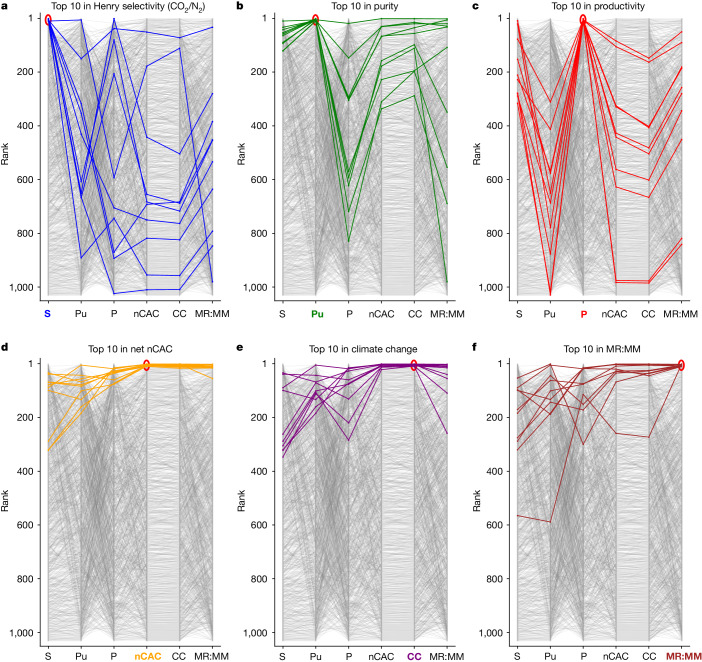
Comparison of materials ranking for a TVSA carbon-capture process at 0.6 bar added to a cement plant in the UK. Rankings according to Henry selectivity (S), purity (Pu), productivity (P), nCAC, climate change (CC) and MR:MM for a TVSA carbon-capture process added to a cement plant in the UK. In these graphs, the top-performing material is ranked number one. Coloured lines represent the top ten performers for the six reference KPIs. The same colour is used to highlight the KPI of interest. Every line illustrates how the ranking of a specific material (*y* axis) changes across all other KPIs (*x* axis). Our visualization tool^25^ gives an interactive version of this graph.

The net carbon avoidance cost (nCAC) is the KPI that quantifies the cost of avoiding CO_2_ emissions into the atmosphere over the plant’s life cycle. The nCAC is not the only criterion, and evaluating materials across all KPIs and from all stakeholders’ perspectives is important. Figure 3 highlights the top-performing materials for a given KPI and their ranking on the other KPIs across the platform. The comparison of the material rankings in Fig. 3 illustrates the complexity of selecting an optimal material; the top ten for a given KPI do not necessarily perform well for the other KPIs.

**Fig. 4:**
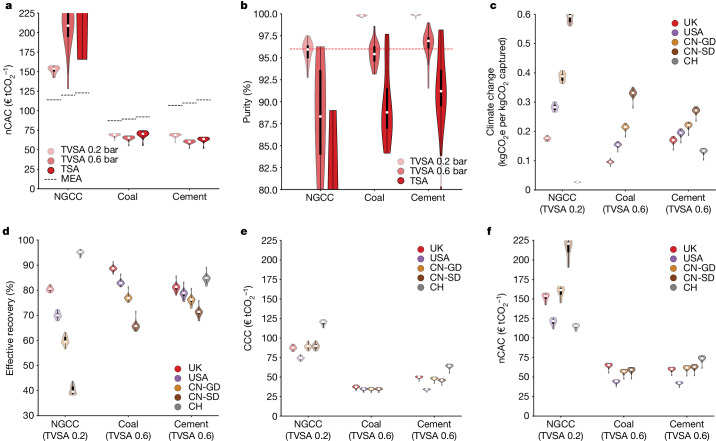
Comparison of process configurations and regions. This analysis of the stakeholders’ perspectives focuses on the 20 materials with the lowest nCAC. In these violin plots, the white circle gives the median, which we use as a (conservative) estimate of the performance. The bottom of the violin represents a few materials with an even better performance. The width indicates the number of structures with a particular *y* value and the thick black bar contains 50% of the structures. **a**, The nCAC jointly with the MEA benchmark (black dashed lines). **b**, The purity for three CO_2_ sources depending on the technologies (TSA and TVSA with two vacuum levels 0.2 bar and 0.6 bar) jointly with the required purity of the CO_2_ sink (red dashed line). **c**,**d**, The climate change (**c**) and effective recovery (**d**) for the five regions. **e**,**f**, The CCC (**e**) and the nCAC (**f**) for the five regions. CN-GD, China Guangdong region; CN-SD, China Shandong region; CH, Switzerland. See Supplementary Information Section 8 for the data.

From an engineering perspective, we are interested in identifying the best technology. Figure 4a compares the nCAC of the 20 top-performing materials for the 3 process configurations and 3 CO_2_ sources. For all three technologies, we find materials that outperform the benchmark for coal and cement. For cases with a low CO_2_ concentration in the feed stream (for example, natural gas combined cycle (NGCC) power plants), the vacuum step in the process configuration reduces the cost, but no materials are identified with a lower nCAC than the MEA benchmark.

The vacuum step increases the purity of the product stream. This increase is achieved by rapidly purging the weakly adsorbed components from the column’s gas phase after the adsorption step but at the expense of a lower recovery than a temperature swing adsorption (TSA) process. Figure 4b shows that with the vacuum step, most materials exceed 96% purity, whereas for TSA, only a few materials meet this requirement for geological storage. Therefore, we focus on operating TVSA with 0.6 bar for the cement and coal and the TVSA with 0.2 bar for the NGCC.

After optimization, many more materials meet the purity requirement (Supplementary Information Section 10.3.3). Optimization lowers the nCAC by about €7 tCO_2_^−1^ (about 12%) for a TVSA process (cement in the UK) and about €9 tCO_2_^−1^ (about 14%) for a TSA process and reduces the differences between the various process configurations. Importantly, we see that the ranking of the top-performing materials has not been impacted significantly.

Running a carbon-capture plant inherently produces emissions of CO_2_ and other greenhouse gases owing to an increased demand for energy and materials. The environmental manager’s perspective focuses on maximizing the captured CO_2_ while simultaneously minimizing these associated CO_2_e emissions and other possible environmental impacts.

**Extended Data Fig. 2:**
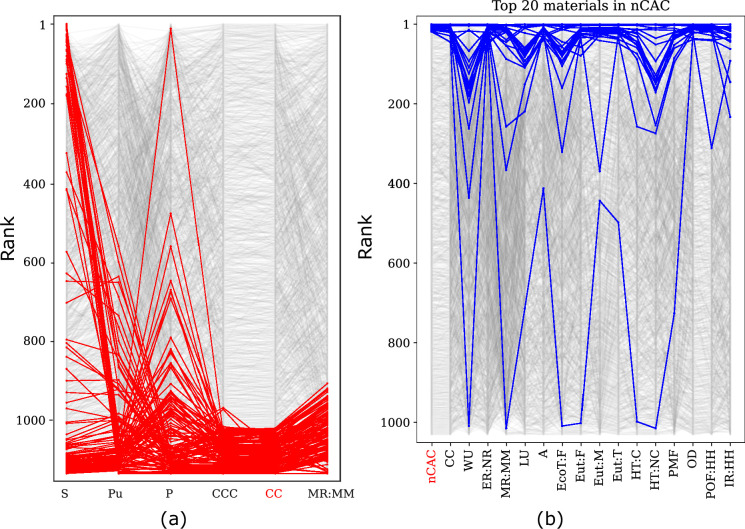
Materials ranking for LCA-KPIs for cement in the UK with TVSA process at 0.6 bar. (a) The materials that are colored red have a Climate Change KPI larger than 1, which implies that the total CO_2_-eq. emissions of the capture plant using this material are larger than the amount of CO_2_ that is captured. Note that we changed nCAC to the Carbon Capture Cost (CCC) (see Supplementary Information Section 6.3.6) because the nCAC is not defined for these materials. (b) Material ranking for all 16 main LCA KPIs: Climate Change (CC), Water Use (WU), Energy Resources: Non-Renewable (ER:NR), Material Resources: Metals/Minerals (MR:MM), Land Use (LU), Acidification (A), Ecotoxicity: Freshwater (EcoT:F), Eutrophication: Freshwater (Eut:F), Eutrophication: Marine (Eut:M), Eutrophication: Terrestrial (Eut:T), Human Toxicity: Carcinogenic (HT:C), Human Toxicity: Non-Carcinogenic (HT:NC), Particulate Matter Formation (PMF), Ozone Depletion (OD), Photochemical Ozone Formation: Human Health (POF:HH), and Ionising Radiation: Human Health (IR:HH). The blue lines show the top 20 materials for the nCAC; the one material showing significant environmental hotspots in 10/16 impact categories contains silver).

**Extended Data Fig. 3:**
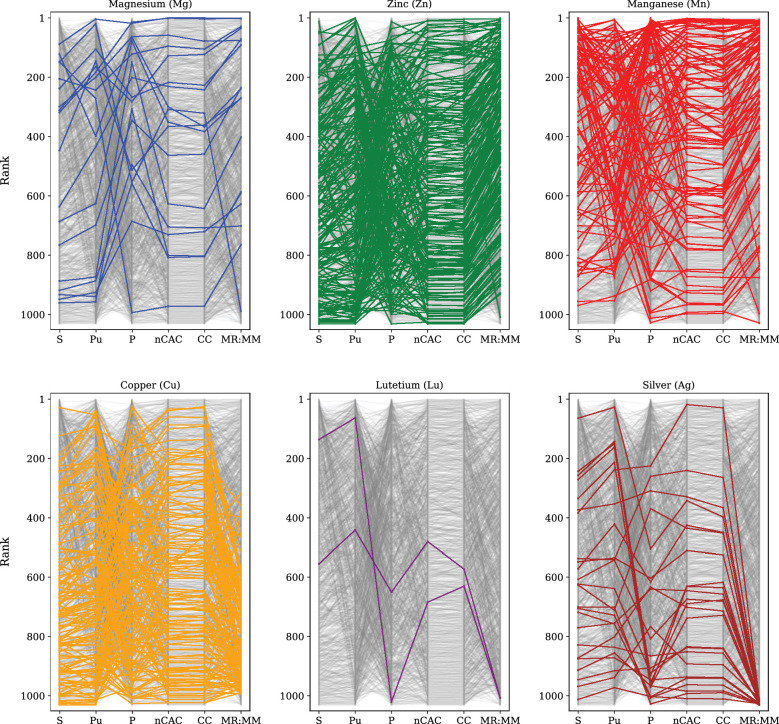
Ranking of the two classes of metals for the cement case in the UK with TVSA process at 0.6 bar. Top: abundant metals (Mg, Zn, Mn), and Bottom: more rare metals (Cu, Lu, Ag). Some MOFs contain more than one type of metal. All these metals are considered in the KPI MR:MM and can lower the ranks significantly. A combination of two or three metals is, for example, contained in the worst-performing Manganese (Mn) materials, leading to their bad performance in MR:MM compared to the other materials containing the same metal.

The effective recovery (Fig. 4d) adjusts the process recovery for the CO_2_e emissions associated with building and operating the carbon-capture plant, using the climate change KPI (Fig. 4c). For some materials, we find that the climate change KPI is >1 kgCO_2_e per kgCO_2_ captured (Extended Data Fig. 2a). This indicates that the capture process with these materials emits more CO_2_e over the plant’s lifetime than the total amount of CO_2_ captured. Several factors can contribute to this result. For example, some materials have a very low CO_2_ working capacity, resulting in high material and energy demands. Some others, with relatively good working capacities and moderate heat demands, contain metals such as gold or rhodium. The climate change impact of synthesizing such materials is so significant that it leads to a climate change KPI >1 kgCO_2_e per kgCO_2_ captured. An important environmental KPI is the material resources:metals/minerals (MR:MM), which relates to the use of minerals and metals resources. In Extended Data Fig. 3, we compare the ranking of materials based on their constituent metals, focusing on some abundant metals (magnesium, zinc and manganese) and rare metals (copper, lutetium and silver). The MR:MM ranking will be poorer if a greater amount of the corresponding MOF is required to remove a unit of CO_2_ or if the total energy demand is higher. The abundant metals rank better, whereas the rank drops for the rather rare metals. If a MOF scores poorly on MR:MM, it may inspire chemists to explore similar structures with more abundant metals.

Another important factor in MOF synthesis is solvent selection. The PrISMa platform identifies the greenest solvent from a list of frequently used ones. Supplementary Information Section 8.2.3 pinpoints anticipated environmental hotspots related to solvent selection.

The platform provides additional KPIs related to the process’s environmental impacts (Extended Data Fig. 2b), for example, impact on ecosystem quality, human health and the use of resources (land, water, materials and non-renewable energy), and allows us to flag materials that impact the environment.

The CO_2_-producer perspective seeks the most cost-effective capture technology. For instance, a cement producer can select different utilities based on their impact on the plant’s environmental footprint and cost. In Switzerland, CO_2_e emissions can be reduced using electric boilers instead of natural-gas-fired ones. This change significantly lowers the climate change KPI owing to the low carbon intensity of Switzerland’s electricity grid, resulting in nearly 100% effective recovery. However, this improvement comes with a cost increase of approximately €16 tCO_2_^−1^ (about 20%) owing to the high operating costs in Switzerland (Supplementary Information Section 8.2.2).

If one needs to perform large-scale carbon capture tomorrow, the default choice is often the well-established MEA technology. However, from an investor’s perspective, our platform shows that solid-sorbent-based capture processes can outperform the MEA benchmark. The cost reductions increase with CO_2_ concentration; for cement, the nCAC is about a factor of two lower than the benchmark (Fig. 4a). Investors are also interested in understanding the economics of deploying carbon-capture plants in different parts of the globe. The large cost differences and electricity grid characteristics will make specific regions economically more beneficial than others. Figure 4e highlights this region’s dependence on the carbon-capture cost (CCC). For the cement case, electricity and natural-gas costs are low in the USA, which makes it favourable in CCC, whereas it is highest in Switzerland. The region dependency of coal costs is rather small, whereas for natural gas, it is more substantial.

However, the CCC does not account for the CO_2_e emissions associated with operating the carbon-capture plant and the product loss (for example, electricity). The nCAC corrects the system-based CCC by the climate change KPI (Fig. 4f). The largest impact is observed in the NGCC case. The high CO_2_e emissions of the electricity grid owing to the many coal power plants in China, particularly in Shandong province, lead to the highest nCAC. In contrast, Switzerland has the lowest because its grid is dominated by hydroelectricity. The low energy cost and CO_2_e emissions of the electricity grid mix make the USA beneficial for coal and cement.

**Extended Data Fig. 4:**
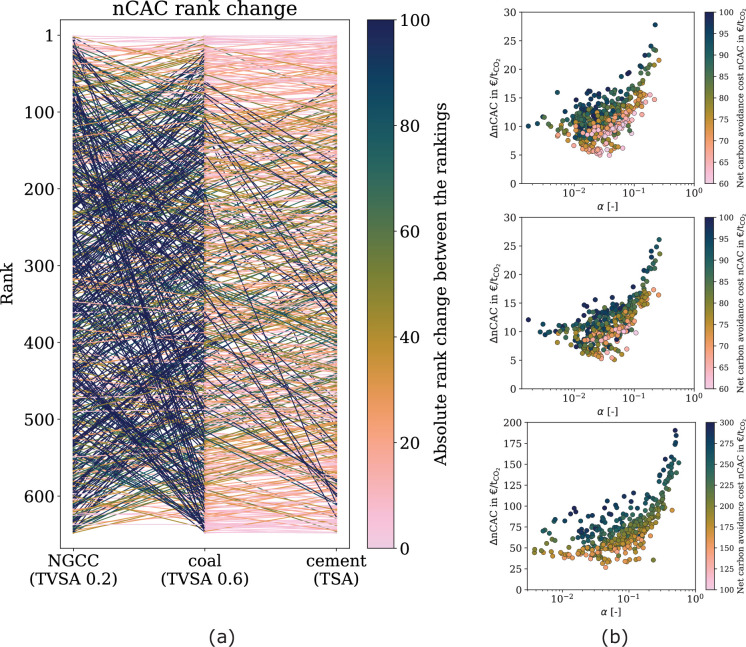
Ranking of materials and comparison between wet and dry flue gasses. (a) Ranking of materials for NGCC power plant (TVSA at 0.2 bar), coal power plant (TVSA at 0.6 bar), and cement plant (TVSA at 0.6 bar). The materials are ranked using the preferred technology according to the Net Carbon Avoidance Cost (nCAC). The color coding of the lines shows the number of ranks a material change ranking. (b) Scatter plots of the increase in nCAC from dry to wet conditions as a function of the fraction of bed that is moisture-loaded. (top) Cement plant (TVSA at 0.6 bar), (middle) coal power plant (TVSA at 0.6 bar), (bottom) NGCC power plant (TVSA at 0.2 bar).

The route from the first synthesis of a new material to its implementation into a commercial process can take many years. It is, therefore, important, from a chemist’s perspective, to provide some guidance on how molecular characteristics impact the material’s performance at the very early material’s design stage. An interesting practical question is whether one can synthesize materials that work well for any CO_2_ source. Extended Data Fig. 4a compares the nCAC ranking for NGCC, coal-fired power plants and cement plants. We observe a significant change in ranking when we go from the NGCC to coal. The changes are smaller but still considerable when we move from coal to cement. This indicates the need for tailored materials for different capture applications (see Supplementary Information Section 8.5.1 for more details).

Extended Data Fig. 4b shows the increase in nCAC with wet versus dry flue gases. As the value of *α*, indicating water penetration in the bed, increases, costs rise substantially, following exponential trends after a certain threshold. For cement, the increase in nCAC is at least €5.0 tCO_2_^−1^ (8%), and €26.7 tCO_2_^−1^ (22%) for NGCC. This underscores the necessity of managing moisture at lower feed-CO_2_ partial pressures to maintain cost competitiveness. In Supplementary Information Section 9, we discuss the limits of our (ideal) model. Under non-ideal mass-transfer conditions, about 60–70% of the materials remain top performers. However, for materials with high water affinity (for example, zeolite 13X), moisture slippage into the dry part of the bed can significantly undermine their capacity and shift their ranking.

**Extended Data Fig. 5:**
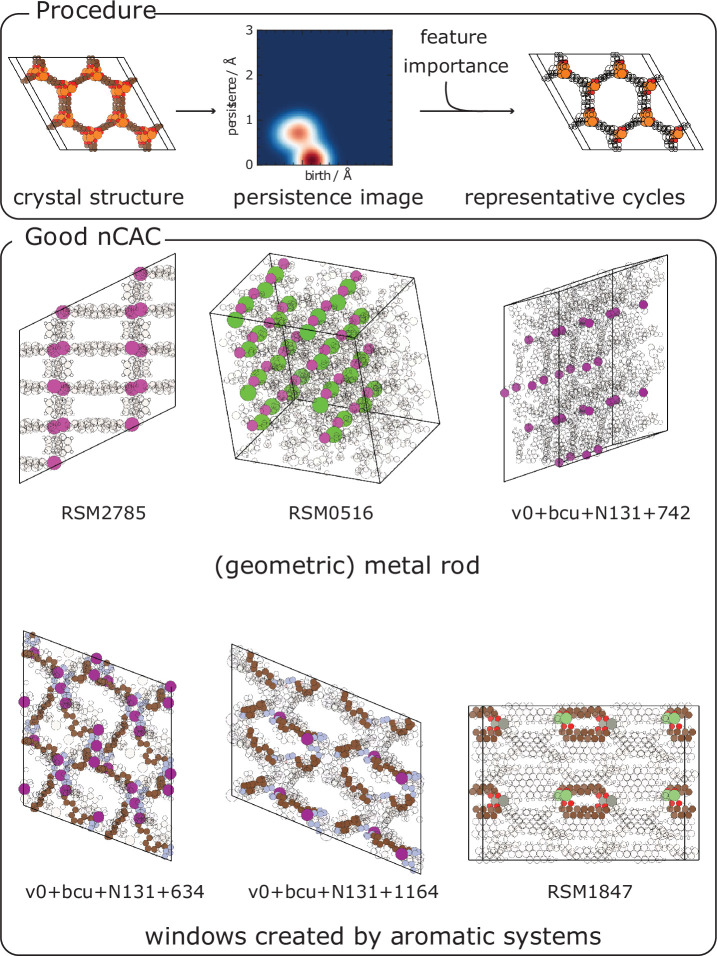
Identifying the adsorbaphore for the cement Case Study (TVSA, 0.6 bar) in the UK. The top figures illustrate the methodology; the crystal structure is converted into a persistence image. We extract the most relevant pixels of the persistence images from a model trained to predict whether the nCAC is lower than the MEA-based benchmark. We then identify representative cycles, which are collections of atoms that generate a corresponding topological feature (i.e., birth/persistence pair). The bottom figure shows examples of the top-performing structures’ recurring molecular features (adsorbaphores). Supplementary Information Section 8.5.3 provides more examples of these top-performing structures, and it gives the details of the methods that are used.

Screening more than a thousand materials enables us to use data-driven methods to identify the molecular characteristics of the top-performing materials. For cement, we demonstrate that by retaining the descriptor related to pore geometry (that is, persistence images), we can accurately predict whether a material has a lower nCAC than the MEA benchmark (Supplementary Information Section 8.5.3). These persistence images also rank the importance of each atom in these predictions, with the collection of these atoms characterizing the molecular features that define the adsorbaphore^23^. A common feature among materials outperforming MEA is a geometrical rod of metal atoms (highlighted in Extended Data Fig. 5). These features are often associated with stacked delocalized systems (aromatic rings) separated by 6 Å to 11 Å (see also Supplementary Fig. 62).

## Extending the chemical design space

Identifying more top-performing materials increases the likelihood of advancing some to the next technological readiness level. We use density functional theory and molecular simulations to predict material properties. Although these predictions are accurate and applicable across case studies, they require substantial central-processing-unit resources and do not scale to millions of materials. To address this, we leverage platform outcomes and implement a machine-learning feedback loop to screen a much larger chemical design space.

**Extended Data Fig. 6:**
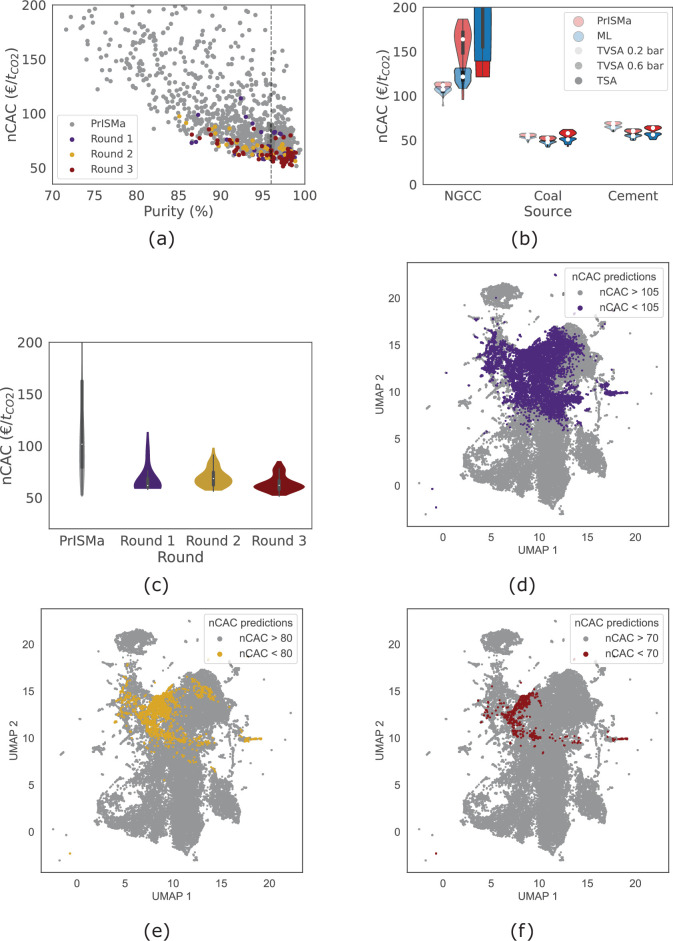
Iterative material discovery. The PrISMa platform was used to train a series of ML models to predict an nCAC below a set of thresholds (105, 80, and 70 €t^−1^_CO2_) for the cement Case Study in the UK (TVSA, 0.6 bar). (a) shows the nCAC versus purity for the different rounds. (b) shows how these materials perform for the other Case Studies. (c) shows the distribution of the nCAC per round. (d)–(f) visualize the screening of the chemical space through dimensionality reduction (UMAP embedding, see Supplementary Information Section 11). Each data point corresponds to one MOF. In (d)-(f), the colored dots are MOFs with an nCAC better than the threshold and the 30,000 grey dots are from the large database.

Our machine-learning model uses the crystal structure to predict whether a material yields an nCAC above or below a given threshold. We have limited top-performing materials, so we perform the training in steps. We start using an nCAC threshold corresponding to the MEA benchmark and use this model to screen a larger database. The most promising materials are added to the platform (round 1, in Extended Data Fig. 6a). We now have more top-performing materials, which allows us to retrain the model with a lower threshold and perform the next round. Extended Data Fig. 6a,c shows that in each round, we decrease the average nCAC. Extended Data Fig. 6d–f shows the evolution of the predictions of our machine-learning model in the chemical design space. Interestingly, there is not one single cluster of top-performing materials but several clusters of chemically different materials. This model needs to be trained for each case study. Indeed, Extended Data Fig. 6b shows that the added top-performing materials for cement do not similarly reduce the nCAC for the NGCC case.

## Experimental testing

**Extended Data Fig. 7:**
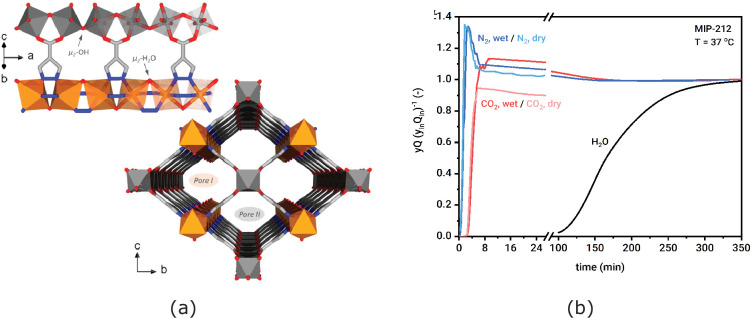
MIP-212. Panel (a) shows the structure of MIP-212. MIP-212 has 1D channels constructed from an alternation of chains of Al hydroxy-carboxylates and Cu pyrazolates. Panel (b) shows the breakthrough curve under a dry and wet flue gas and conditions corresponding to the cement Case Study.

**Extended Data Fig. 8:**
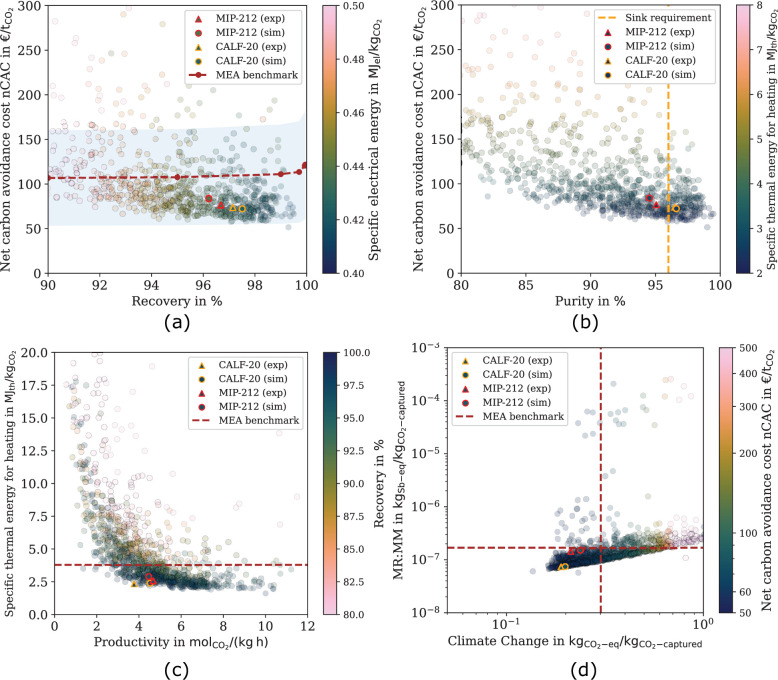
Materials performance for a TVSA carbon capture process at 0.6 bar added to a cement plant in the UK using experimental data. The dotted lines in (a), (c), and (d) show the MEA benchmark, in (b), the vertical orange dotted line gives the purity required for geological storage (> 96 %), and in (a), the blue-shaded area gives the uncertainty. Each dot represents the corresponding KPI of a material. The triangles are the structures for which experimental property data is used directly in the platform (see Supplementary Information Section 12). (a) Net Carbon Avoidance Cost (nCAC) versus recovery (R) with color coding the specific electrical energy consumption, (b) nCAC versus purity (Pu) with color coding the specific thermal energy consumption, (c) Specific thermal energy consumption for heating versus productivity (P) with color coding the recovery, and (d) Material Resources: Metals/Minerals (MR:MM) versus Climate Change (CC) with color coding the nCAC.

The impact of our in silico screening is limited if it cannot reflect the experimental performance of the material. As an example, we uploaded the crystal structure of a new material, MIP-212 (Extended Data Fig. 7a). Extended Data Fig. 8 shows this is a promising material, and we studied the performance in detail (Supplementary Information Section 12.1). The experimental breakthrough curves (Extended Data Fig. 7b) show the separation between the column’s predicted wet and dry fronts. The significant lapse between the breakthrough times of CO_2_ and H_2_O indicates moisture penetration below 5% of the bed length, which is in good agreement with our predictions (Supplementary Information Section 12.1.3).

We also ranked CALF-20 in Extended Data Fig. 8, which gives an nCAC of €72 tCO_2_^−1^.

CALF-20 is being commercialized, and the estimated CO_2_ capture cost for the Svante process is €50 tCO_2_^−1^ (ref. ^24^). A head-to-head comparison is, however, difficult as the two processes fundamentally differ.

## Outlook

The PrISMa platform’s holistic approach identifies promising sorbent materials for carbon-capture applications. This modular platform extends beyond carbon capture, allowing for additional modules, for example, other gas separations and hydrogen or methane storage. Bridging fundamental research and large-scale deployment accelerates the successful implementation of innovations.

## Online content

Any methods, additional references, Nature Portfolio reporting summaries, source data, extended data, supplementary information, acknowledgements, peer review information; details of author contributions and competing interests; and statements of data and code availability are available at 10.1038/s41586-024-07683-8.

## Supplementary information

Supplementary information Supplementary text and data, including Supplemenatry Figs. 1–91, Tables 1–11 and refs. 1–241. See contents for details.

Peer Review file

Data isotherms Supplementary Figs. 8–26 Experimental and computational data of CO_2_, N_2_ and H_2_O adsorption isotherms in different MOFs.

Breakthrough data

## Figures and Tables

**Extended Data Table 1:**
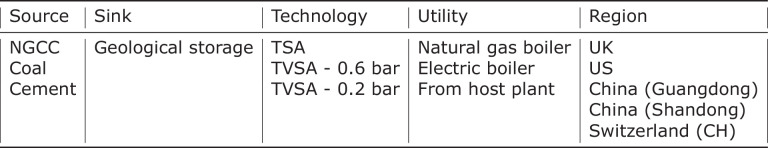
Available case studies in the PrISMa platform

Any source, sink, technology, utility, and region in the platform can be combined. Switzerland has no coal-fired power plants, so this combination of source and region is not considered. Three technologies are available: temperature swing adsorption (TSA) and temperature vacuum swing adsorption (TVSA) with two vacuum levels, 0.2 and 0.6 bar. We have a total of 66 Case Studies. The complete input parameters defining these Case Studies are in Supplementary Information Table S2.

**Extended Data Table 2:**
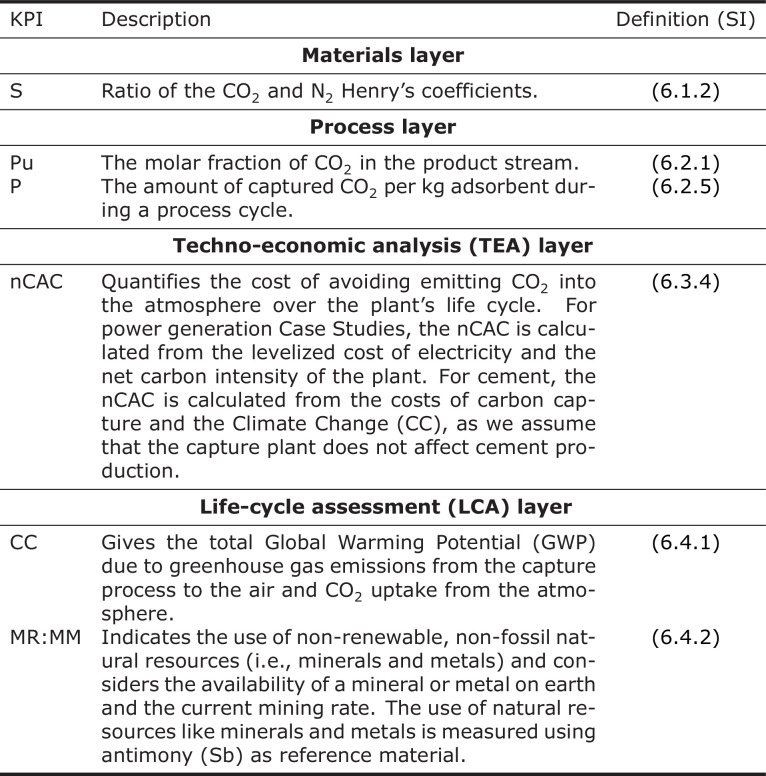
The six reference key performance indicators (KPIs)

Henry selectivity (S), Purity (Pu), Productivity (P), Net Carbon Avoidance Cost (nCAC), Climate Change (CC), and Material Resources: Metals/Minerals (MR:MM). Based on Spearman analysis, we have identified six key performance indicators that describe the most important trends in each layer of the PrISMa platform (see Supplementary Information Section 7). A description of all KPIs and data generated by the platform can be found in Supplementary Information Section 6.

## Data Availability

All the results obtained by the platform for all case studies presented in this work have been deposited on Zenodo at 10.5281/zenodo.11244258 (ref. ^26^). On this website, one can also find the crystal structure (cif files) of all materials studied in this work, together with the simulated isotherms, values of the heat capacity and the data that characterize the materials. The results of this work can also be accessed through our visualization tool on the Materials Cloud at https://prisma.materialscloud.io/ (ref. ^25^). This tool allows users to inspect all case studies and all KPIs. In addition, the Materials Cloud provides interactive versions of the graphs presented in this work. Updates and new case studies will be made available through the Materials Cloud. This tool also allows the crystal structure of materials to be uploaded and analysed across various case studies.

## Code availability

The code for the analysis of the persistence images and the interactive visualization tool can be found at https://github.com/kjappelbaum/prisma-adosorbaphore and https://github.com/ElMouba/PrISMa_VisTool, respectively. The software to run the different layers of the platform can be obtained from the corresponding authors upon request.

## References

[1] *Net Zero Roadmap A Global Pathway to Keep the 1.5* *°C Goal in Reach* (IEA, 2023); https://www.iea.org/reports/net-zero-roadmap-a-global-pathway-to-keep-the-15-0c-goal-in-reach#previous-editions.

[2] Smit, B., Reimer, J. R., Oldenburg, C. M. & Bourg, I. C. *Introduction to Carbon Capture and Sequestration* (Imperial College Press, 2014).

[3] Schoedel, A., Ji, Z. & Yaghi, O. M. The role of metal–organic frameworks in a carbon-neutral energy cycle. *Nat. Energy* 10.1038/nenergy.2016.34 (2016).

[4] Farmahini, A. H., Krishnamurthy, S., Friedrich, D., Brandani, S. & Sarkisov, L. Performance-based screening of porous materials for carbon capture. *Chem. Rev.* **121**, 10666–10741 (2021). 34374527 10.1021/acs.chemrev.0c01266 PMC8431366

[5] Balogun, H. A., Bahamon, D., AlMenhali, S., Vega, L. F. & Alhajaj, A. Are we missing something when evaluating adsorbents for CO_2_ capture at the system level?. *Energy Environ. Sci.* **14**, 6360–6380 (2021). 10.1039/D1EE01677F

[6] Deutz, S. & Bardow, A. Life-cycle assessment of an industrial direct air capture process based on temperature-vacuum swing adsorption. *Nat. Energy* **6**, 203–213 (2021). 10.1038/s41560-020-00771-9

[7] Bui, M. et al. Carbon capture and storage (CCS): the way forward. *Energy Environ. Sci.* **11**, 1062–1176 (2018). 10.1039/C7EE02342A

[8] Furukawa, H., Cordova, K. E., O’Keeffe, M. & Yaghi, O. M. The chemistry and applications of metal–organic frameworks. *Science* **341**, 1230444 (2013). 23990564 10.1126/science.1230444

[9] Moghadam, P. Z. et al. Development of a Cambridge structural database subset: a collection of metal–organic frameworks for past, present, and future. *Chem. Mater.* **29**, 2618–2625 (2017). 10.1021/acs.chemmater.7b00441

[10] Boyd, P. G., Lee, Y. J. & Smit, B. Computational development of the nanoporous materials genome. *Nat. Rev. Mater.* **2**, 17037 (2017). 10.1038/natrevmats.2017.37

[11] Wilmer, C. E. et al. Large-scale screening of hypothetical metal–organic frameworks. *Nat. Chem.* **4**, 83–89 (2012). 10.1038/nchem.1192 22270624

[12] Daglar, H. & Keskin, S. Recent advances, opportunities, and challenges in high-throughput computational screening of MOFs for gas separations. *Coord. Chem. Rev.*10.1016/j.ccr.2020.213470 (2020).

[13] Findley, J. M. & Sholl, D. S. Computational screening of MOFs and zeolites for direct air capture of carbon dioxide under humid conditions. *J. Phys. Chem. C* **125**, 24630–24639 (2021). 10.1021/acs.jpcc.1c06924

[14] Leperi, K. T., Chung, Y. G., You, F. Q. & Snurr, R. Q. Development of a general evaluation metric for rapid screening of adsorbent materials for postcombustion CO_2_ capture. *ACS Sustain. Chem. Eng.* **7**, 11529–11539 (2019). 10.1021/acssuschemeng.9b01418

[15] Burns, T. D. et al. Prediction of MOF performance in vacuum swing adsorption systems for postcombustion CO_2_ capture based on integrated molecular simulations, process optimizations, and machine learning models. *Environ. Sci. Technol.* **54**, 4536–4544 (2020). 32091203 10.1021/acs.est.9b07407

[16] Hu, J. Y., Gu, X. M., Lin, L. C. & Bakshi, B. R. Toward sustainable metal–organic frameworks for post-combustion carbon capture by life cycle assessment and molecular simulation. *ACS Sustain. Chem. Eng.* **9**, 12132–12141 (2021). 10.1021/acssuschemeng.1c03473

[17] Ajenifuja, A., Joss, L. & Jobson, M. A new equilibrium shortcut temperature swing adsorption model for fast adsorbent screening. *Ind. Eng. Chem. Res.* **59**, 3485–3497 (2020). 10.1021/acs.iecr.9b05579

[18] Maring, B. J. & Webley, P. A. A new simplified pressure/vacuum swing adsorption model for rapid adsorbent screening for CO_2_ capture applications. *Int. J. Greenhouse Gas Control* **15**, 16–31 (2013). 10.1016/j.ijggc.2013.01.009

[19] Danaci, D., Bui, M., Mac Dowell, N. & Petit, C. Exploring the limits of adsorption-based CO_2_ capture using MOFs with PVSA—from molecular design to process economics. *Mol. Syst. Des. Eng.* **5**, 212–231 (2020). 10.1039/C9ME00102F

[20] Taddei, M. & Petit, C. Engineering metal–organic frameworks for adsorption-based gas separations: from process to atomic scale. *Mol. Syst. Des. Eng.* **6**, 841–875 (2021). 10.1039/D1ME00085C

[21] Terlouw, T., Bauer, C., Rosa, L. & Mazzotti, M. Life cycle assessment of carbon dioxide removal technologies: a critical review. *Energy Environ. Sci.* **14**, 1701–1721 (2021). 10.1039/D0EE03757E

[22] Anantharaman, R., Fu, C., Roussanaly, S. & Voldsund, M. *D3.2 CEMCAP Framework for Comparative Techno-economic Analysis of CO*_*2*_*Capture from Cement Plants* (2017); https://tinyurl.com/ycyk7t6y.

[23] Boyd, P. G. et al. Data-driven design of metal–organic frameworks for wet flue gas CO_2_ capture. *Nature* **576**, 253–256 (2019). 31827290 10.1038/s41586-019-1798-7

[24] *Carbon Capture & Removal Solutions Provider, Svante, Responds to the US’s New Inflation Reduction Act* (Svante Inc., 2022); https://tinyurl.com/2s3fv6zs.

[25] Moubarak, E. & Van Herck, J. PrISMa Visualization Tool (2024); https://prisma.materialscloud.io/.

[26] Charalambous, C. et al. A holistic platform for accelerating sorbent-based carbon capture. *Zenodo*10.5281/zenodo.11244258 (2024). 10.1038/s41586-024-07683-8 PMC11291289 39020168

