# Supplementary material for: A holistic platform for accelerating sorbent-based carbon capture
Source: Nature. 2024 Jul 17;632(8023):89–94. doi: 10.1038/s41586-024-07683-8 (PMC11291289; doi:10.1038/s41586-024-07683-8)
Supplement: Supplementary file 1 — Supplementary text and data, including Supplemenatry Figs. 1–91, Tables 1–11 and refs. 1–241. See contents for details. [file 41586_2024_7683_MOESM1_ESM.pdf]

nature portfolio

https://doi.org/10.1038/s41586-024-07683-8

Supplementary information

A holistic platform for accelerating sorbent-based carbon capture

In the format provided by the authors and unedited

Supporting Information: A Holistic Platform for Accelerating Sorbent-Based Carbon Capture

Charithea Charalambous,[†,#] Elias Moubarak,[‡,#] Johannes Schilling,[¶,#] Eva Sanchez Fernandez,[§] Jin-Yu Wang,[†] Laura Herraiz,[†] Fergus McIlwaine,[†] Shing Bo Peh,[†] Matthew Garvin,[†] Kevin Maik Jablonka,[‡] Seyed Mohamad Moosavi,[‡] Joren Van Herck,[‡] Aysu Yurdusen Ozturk,[‖] Alireza Pourghaderi,[⊥] Ah-Young Song,[⊥] Georges Mouchaham,[‖] Christian Serre,[‖] Jeffrey A. Reimer,[⊥] André Bardow,[¶] Berend Smit,[*,‡] and Susana Garcia[*,†]

†*The Research Centre for Carbon Solutions (RCCS), School of Engineering and Physical Sciences, Heriot-Watt University, EH14 4AS Edinburgh, United Kingdom*

‡*Laboratory of Molecular Simulation (LSMO), Institut des Sciences et Ingénierie Chimiques, École Polytechnique Fédérale de Lausanne (EPFL), Rue de l'Industrie 17, CH-1951 Sion, Valais, Switzerland*

¶*Laboratory of Energy and Process Systems Engineering (EPSE), ETH Zurich, 8092 Zurich, Switzerland*

§*Solverlo Ltd, EH42 1TL Dunbar, United Kingdom*

‖*Institut des Matériaux Poreux de Paris, ENS, ESPCI Paris, CNRS, PSL university, Paris, France*

⊥ *Materials Science Division, Lawrence Berkeley National Laboratory, Berkeley, California 94720, United States; Department of Chemical and Biomolecular Engineering, University of California, Berkeley, California 94720, United States*

#*Contributed equally to this work.*

E-mail: berend.smit@epfl.ch; s.garcia@hw.ac.uk

Summary

This supplementary information document contains a detailed description of the PrISMa platform, including:

- A description of all investigated Case Studies (Section 8) and various databases used in each layer of the platform (Section 2).
- A detailed description of all the layers in the platform and the methods used in each of the layers (Section 3).
- A description of the assumptions for the MEA benchmark case (Section 4), as well as a detailed comparison of the predicted versus experimental isotherms (Section 5).
- A description of all the KPIs and data generated by the platform can be found in Table S5. This table links to the sections in which the properties and KPIs are defined.
- A description of the Spearman analysis we conducted to identify our reference KPIs (Section 7).
- A detailed description of the different stakeholder's views on all case studies is in Section 8.
- A detailed analysis of the impact of water on KPIs and the limits of the model (Section 9).
- A description of the applied optimization and obtained results (Section 10), and the different feedback loops of the platform (Section 11).
- A description of the experimental structures investigated and/or evaluated in this work (Section 12).

Contents

Summary **S-2**

1 Case studies **S-9**

2 The PrISMa databases **S-14**

3 Methods **S-17**

3.1 Materials layer S-17

3.1.1 Material properties S-17

3.1.2 Molecular simulations S-20

3.1.3 Flexibility of the MOFs S-22

3.1.4 Output of the layer S-23

3.2 Process layer S-23

3.2.1 Process description S-23

3.2.2 Cycle time S-30

3.2.3 Energy requirements S-31

3.2.4 Validation of the layer S-37

3.2.5 Output of the layer S-38

3.3 Techno-Economic Analysis layer S-38

3.3.1 TEA system boundaries S-38

3.3.2 CAPEX calculation S-38

3.3.3 OPEX calculation S-40

3.3.4 Utilities calculation S-41

3.3.5 Validation of the layer S-43

3.3.6 Output of the layer S-43

3.4 LCA layer S-44

3.4.1 Goal and scope definition S-44

3.4.2 Life cycle inventory S-45
3.4.3 Life cycle impact assessment S-50
3.4.4 Validation of the layer S-51
3.4.5 Output of the layer S-52

4 MEA Benchmark **S-53**

4.1 TEA assumptions for the MEA benchmark S-53
4.2 LCA assumptions for the MEA benchmark S-54
4.3 Additional remark S-56

5 Materials layer: Comparison with experimental data **S-58**

5.1 Gas sorption experiments S-58
5.2 Al-containing MOFs S-58
5.2.1 MIL-160 . . . . S-60
5.2.2 MIL-96 . . . . S-62
5.2.3 CAU-10 . . . . S-63
5.2.4 MIL-53-NH2 . . . . S-64
5.2.5 MIP-212 . . . . S-66
5.2.6 NOTT-300 . . . . S-68
5.3 Other MOFs S-69
5.3.1 CALF-20 . . . . S-69
5.3.2 MOF-74(Mn) S-71
5.3.3 FMOF-1 . . . . S-72
5.3.4 PCN-14 . . . . S-73
5.3.5 UiO-66 . . . . S-74
5.3.6 MOF-5 . . . . S-75
5.3.7 MOF-205 . . . . S-76
5.3.8 UiO-66-NH2 . . . . S-77

5.3.9 UiO-67 . . . S-78
5.3.10 HKUST-1 . . . S-79
5.3.11 UMCM-1 . . . S-80
5.3.12 Ca-SQA . . . S-81
5.4 Zeolites . . . S-82
5.4.1 Zeolite-13X . . . S-82
5.5 Discussion of the results . . . S-83
5.6 Error propagation . . . S-84

6 Output of the PrISMa platform S-86

6.1 Output at the materials level . . . S-88
6.1.1 Henry coefficient . . . S-88
6.1.2 Henry selectivity . . . S-88
6.1.3 Pure component capacity . . . S-89
6.1.4 Water resistance coefficient . . . S-89
6.1.5 Foreground data for the Process layer . . . S-89
6.1.6 Flags at the materials layer . . . S-91
6.2 Output at the process level . . . S-91
6.2.1 Purity . . . S-91
6.2.2 Recovery . . . S-92
6.2.3 Effective recovery . . . S-92
6.2.4 Working Capacity . . . S-92
6.2.5 Productivity . . . S-93
6.2.6 Specific thermal energy . . . S-93
6.2.7 Specific thermal energy for cooling . . . S-94
6.2.8 Specific electrical energy . . . S-94
6.2.9 Foreground data for the TEA layer . . . S-94
6.3 Output at the TEA layer . . . S-96

6.3.1 Capital Expenditure (CAPEX) S-96
6.3.2 Operational Expenditure (OPEX) S-96
6.3.3 Carbon Avoidance Cost (CAC) S-97
6.3.4 Net Carbon Avoidance Cost (nCAC) S-98
6.3.5 Levelised Cost of Capture (LCOC) S-99
6.3.6 Carbon Capture Cost (CCC) S-99
6.3.7 Levelized Cost Of Electricity (LCOE) S-100
6.3.8 Carbon Dioxide Avoided S-101
6.3.9 Specific carbon dioxide emissions S-102
6.3.10 SPECCA S-102
6.3.11 Foreground data for the LCA layer S-104
6.4 Output at the LCA layer S-105
6.4.1 Climate Change S-106
6.4.2 Resources S-106
6.4.3 Ecosystem Quality S-107
6.4.4 Human Health S-108

7 Spearman analysis S-110

8 The stakeholders' perspectives S-113

8.1 The engineer's perspective S-115
8.2 The environmental manager's perspective S-119
8.2.1 Climate Change S-119
8.2.2 Impact of different utilities S-127
8.2.3 Trends in environmental impact categories S-131
8.3 The Carbon dioxide producer's perspective S-137
8.3.1 Different carbon capture cost metrics S-137
8.3.2 Different sources S-141

8.4 The investor's perspective . . . S-144
8.4.1 Different regions in the world . . . S-144
8.4.2 Utility prices . . . S-146
8.4.3 The cost and replacement rates of the MOFs . . . S-155
8.5 The chemist's perspective . . . S-158
8.5.1 Materials across Case Studies . . . S-158
8.5.2 Effect of the flexiblity . . . S-158
8.5.3 Identifying the adsorbaphores . . . S-160

9 Impact of water S-170
9.1 Direct impact of moisture on the capture performance . . . S-170
9.2 Impact of moisture on the material rankings . . . S-171
9.3 Limits of the model . . . S-179
9.3.1 Impact of mass transfer limitations on the wet performance . . . S-179
9.3.2 Impact of moisture slippage . . . S-180

10 Process optimization S-187
10.1 Tornado plot analysis . . . S-187
10.2 Problem formulation . . . S-194
10.3 Optimization results . . . S-197
10.3.1 Optimization results for the TSA case . . . S-197
10.3.2 Optimization results for the TVSA case . . . S-198
10.3.3 Comparison of the TSA and TVSA cases . . . S-200

11 Feedback loops of the platform S-205
11.1 Feedback to the materials layer . . . S-205
11.1.1 Heat capacity . . . S-206
11.1.2 Adsorption isotherms . . . S-206
11.2 Feedback to extend the materials database . . . S-206

11.2.1 Detailed description of the methodology S-207
11.2.2 Performance of the Machine learning model S-210
11.2.3 Results of the feedback loop S-211

12 Experimental structures **S-216**

12.1 MIP-212 . . . . S-216
12.1.1 Structure and synthesis S-216
12.1.2 NMR-characterisation of residual water S-217
12.1.3 Performance testing: Breakthrough curves S-219
12.2 CALF-20 . . . . S-222

Acronyms **S-224**

Symbols **S-228**

References **S-231**

1 Case studies

The **Pr**ocess-**I**nformed design of tailor-made **S**orbent **Ma**terials (PrISMa) platform has been designed to screen solid adsorbent materials for carbon capture. The PrISMa platform can be applied to a range of case studies that are defined by a CO_2 source, a CO_2 sink, a process configuration, and a particular geographic region in the world. The specifications of the flue/industrial gas (e.g., flow rate, composition, temperature, and pressure) and the availability of resources and utilities (e.g., heat, electricity) change depending on the CO_2 source. The CO_2 sink defines the specifications of the product stream from the capture process (e.g., purity, temperature, and pressure). The techno-economic analysis and the life cycle assessment consider parameters specific to each geographic region.

This work analyzes a range of case studies (66 in total) to demonstrate the versatility of the PrISMa platform and assess the effect of CO_2 concentration, region-specific parameters, availability of utilities, fuel, and electricity prices on the selection of the optimal materials for a specific case study. Table S1 summarizes the case studies presented in this work, corresponding to 22 source-sink-region-utility combinations combined with three process configurations.

Three CO_2 sources are investigated: an onshore Natural Gas Combined Cycle (NGCC) power plant, a pulverized coal-fired power plant, and a cement plant. The plants can be located in five geographic regions, i.e., the UK, the USA, Switzerland, and China (two provinces). The northern and southern electricity grids of China differ strongly in carbon intensity, and we use the Guangdong and Shandong regions as representatives for the North and South, respectively. Coal power plants are not used in Switzerland and are therefore excluded in the case studies related to coal. Energy prices in the UK are taken for two years, 2019 and 2022, to consider the considerable increase in electricity and natural gas prices due to unforeseen circumstances. The captured CO_2 is sent to geological storage in all case studies. As capture technologies, we consider Temperature Swing Adsorption (TSA) and Temperature Vacuum Swing Adsorption (TVSA) with two vacuum levels 0.2 bar and

0.6 bar. As utilities, we either use the available utilities at the host plant and/or, if required, additional thermal energy for heating can be generated using natural gas or electric boilers, and the electricity grid can supply electricity demand.

In Table S2, we summarize the main flue/industrial gas and host plant characteristics, the required CO_2 product purity, and conditions from the CO_2 sink. In Table S3, the regional energy costs and main cost factors used for the case studies are presented.

Table S1: Summary of all case studies.

Source	Sink	Process Conf.	Region	Ref. Year	Heating Supply	Cooling Supply	Electricity Supply
NGCC	Geo. storage	TSA<br>TVSA (0.6 bar)<br>TVSA (0.2 bar)	UK	2019	Steam extraction<br>NG boiler	Existing cooling<br>Additional cooling	Onsite power<br>Electricity grid
					NG boiler	Additional cooling	
				2022	Steam extraction<br>NG boiler	Existing cooling<br>Additional cooling	
			US	2019	Steam extraction<br>NG boiler	Existing cooling<br>Additional cooling	
			China - Shandong	2019	Steam extraction<br>NG boiler	Existing cooling<br>Additional cooling	
			China - Guangdong	2019	Steam extraction<br>NG boiler	Existing cooling<br>Additional cooling	
			Switzerland	2019	Steam extraction<br>NG boiler	Existing cooling<br>Additional cooling	
					NG boiler	Additional cooling	
					Steam extraction<br>Electric boiler	Existing cooling<br>Additional cooling	
					Electric boiler	Additional cooling	
Coal	Geo. storage	TSA<br>TVSA (0.6 bar)<br>TVSA (0.2 bar)	UK	2019	Steam extraction<br>NG boiler	Existing cooling<br>Additional cooling	Onsite power<br>Electricity grid
					NG boiler	Additional cooling	
			US	2019	Steam extraction<br>NG boiler	Existing cooling<br>Additional cooling	
			China - Shandong	2019	Steam extraction<br>NG boiler	Existing cooling<br>Additional cooling	
			China - Guangdong	2019	Steam extraction<br>NG boiler	Existing cooling<br>Additional cooling	
Cement	Geo. storage	TSA<br>TVSA (0.6 bar)<br>TVSA (0.2 bar)	UK	2019	Waste heat<br>NG boiler	Additional cooling	Electricity grid
				2022	Waste heat<br>NG boiler		
			US	2019	Waste heat<br>NG boiler		
			China - Shandong	2019	Waste heat<br>NG boiler		
			China - Guangdong	2019	Waste heat<br>NG boiler		
			Switzerland	2019	Waste heat<br>NG boiler		
					Waste heat<br>Electric boiler		

Table S2: Parameters characterizing each case study.

Parameter	Unit	Value	Description	Ref.
		**CO_2 Source**		
**NGCC power plant**				
y_{all}	-, wet molar basis	[0.0396, 0.0838, 0.7438, 0.1239, 0.0089]	molar fraction of all species (i.e., CO_2, H_2O, N_2, O_2, Ar) in feed stream	S1
$y_{feed,dry}$	-, dry molar basis	[0.0432, 0.9568]	molar fraction of CO_2 and N_2 in the dry feed stream	S1
$y_{feed,wet}$	-, wet molar basis	[0.0396, 0.0838, 0.8766]	molar fraction of CO_2, H_2O and N_2 in the feed	S1
T_{feed}	$^{\circ}C$	86.8	temperature of feed stream	S1
P_{feed}	bar	1.01	pressure of feed stream	S1
F_{feed}	$kg\,s^{-1}$	1330.6	mass flow rate of feed stream (wet)	S1
N_{feed}	$mol\,s^{-1}$	42912.41	molar flow rate of feed stream (dry)	S1
N_{water}	$mol\,s^{-1}$	3924.97	molar flow rate of water in feed stream	S1
Construction time	year	3	construction period for reference plant	S2
Plant lifetime	year	25	lifetime of reference plant	S2
Allocation	year	[0.4, 0.3, 0.3]	total capital requirement during construction	S2
CO_2 intensity	kg_{CO_2}/MWh	351.67	direct specific emissions of reference plant	S2
primary energy demand	MJ(LHV)/MWh	6170.7	direct primary energy demand of reference plant	S2
operating hours	h/year	[3500, 5700, 7500, ..., 7500]	operating hours for 1st, 2nd, 3rd, ..., Nth year	S2
Host plant utilities			power generation system (available power, heat, cooling)	
Low pressure steam flow	$kg\,s^{-1}$	205.0	low pressure steam mass flow to turbine	S1
Available power	MW	830.0	maximum power available in the plant	S2
**Coal power plant**				
y_{all}	-, wet molar basis	[0.1373, 0.0973, 0.72855, 0.0365, 0.00005]	molar fraction of all species (i.e., CO_2, H_2O, N_2, O_2, Ar) in feed stream	S1
$y_{feed,dry}$	-, dry molar basis	[0.1521, 0.8479]	molar fraction of CO_2 and N_2 in the dry feed stream	S1
$y_{feed,wet}$	-, wet molar basis	[0.1373, 0.0973, 0.7651]	molar fraction of CO_2, H_2O and N_2 in the feed	S1
T_{feed}	$^{\circ}C$	50	temperature of feed stream	S1
P_{feed}	bar	1.016	pressure of feed stream	S1
F_{feed}	$kg\,s^{-1}$	735.12	mass flow rate of feed stream (wet)	S1
N_{feed}	$mol\,s^{-1}$	24016.34	molar flow rate of feed stream (dry)	S1
N_{water}	$mol\,s^{-1}$	2589.53	molar flow rate of water in feed stream	S1
Construction time	year	3	construction period for reference plant	S2
Plant lifetime	year	40	lifetime of reference plant	S2
Allocation	year	[0.2, 0.3, 0.3, 0.2]	total capital requirement during construction	S2
CO_2 intensity	kg_{CO_2}/MWh	771.9	direct specific emissions of reference plant	S2
primary energy demand	MJ(LHV)/MWh	7955.8	direct primary energy demand of reference plant	S2
operating hours	h/year	[3500, 5700, 7500, ..., 7500]	operating hours for 1st, 2nd, 3rd, ..., Nth year	S2
Host plant utilities			power generation system (available power, heat, cooling)	
Low pressure steam flow	$kg\,s^{-1}$	396.36	low pressure steam mass flow to turbine	S1
Available power	MW	758.6	maximum power available in the plant	S2
**Cement plant**				
y_{all}	-, wet molar basis	[0.18, 0.09, 0.63, 0.10, 0.00]	molar fraction of all species (i.e., CO_2, H_2O, N_2, O_2, Ar) in feed stream	S3
$y_{feed,dry}$	-, dry molar basis	[0.1978, 0.8022]	molar fraction of CO_2 and N_2 in the dry feed stream	S3
$y_{feed,wet}$	-, wet molar basis	[0.18, 0.09, 0.73]	molar fraction of CO_2, H_2O and N_2 in the feed	S3
T_{feed}	$^{\circ}C$	110	temperature of feed stream	S3
P_{feed}	bar	1.013	pressure of feed stream	S3
F_{feed}	$kg\,s^{-1}$	107.81	mass flow rate of feed stream (wet)	S3
N_{feed}	$mol\,s^{-1}$	3229.18	molar flow rate of feed stream (dry)	S3
N_{water}	$mol\,s^{-1}$	319.37	molar flow rate of water in feed stream	S3
Construction time	year	3	construction period for reference plant	S3
Plant lifetime	year	25	lifetime of reference plant	S3
Allocation	year	[0.4, 0.3, 0.3]	total capital requirement during construction	S2
CO_2 intensity	kg_{CO_2}/t cement	653	total specific emissions of reference plant	S4
primary energy demand	MJ(LHV)/MWh	2306.67	direct primary energy demand of reference plant	S4
operating hours	h/year	[7998, 7998, 7998, ..., 7998]	operating hours for 1st, 2nd, 3rd, ..., Nth year	S4
Host plant utilities			industrial site (available waste heat)	
Available waste heat	MW	8.2	heat recovered from clinker cooler calculated in this work	–
Cooler air exhaust temperature	$^{\circ}C$	285	temperature of the air leaving the clinker cooler	S4
		**CO_2 sink**		
purity	-	0.96	required purity of CO_2 product stream before compression	S1
pressure	bar	110.0	final pressure of CO_2 product stream after compression	S1
temperature	$^{\circ}C$	40.0	temperature for CO_2 compression	S1

Table S3: Parameters characterizing each region

Parameter	Unit	Value					Description	Ref.
**Region**		**UK**	**Switzerland**	**US**	**China Guangdong**	**China Shandong**		
Location factor	–	1	1	1	0.77	0.77	Location factor multiplies Total Direct Cost	S5
CEPCI	US2019<br>US2022			607.5<br>804.6			Chemical Engineering Plant Cost Index	S6
Operator cost factor	–	1	1	1	0.2	0.2	Multiplies yearly cost per operator	S5
Electricity cost (2019)	€/MW h	131.7	107.0	60.9	98.25	80.80	annual average electricity cost	S7 S8
NG cost (2019)	€/MW h	24.63	59.65	11.52	37.12	37.12	annual average natural gas cost	S9 S10
Coal cost (2019)	€/MW h	9.42	–	5.43	10.91	10.97	annual average coal cost	S9 S11 S12
Electricity cost (2022)	€/MW h	216.3	–	–	–	–	Jan-Sep 2022 average electricity cost	S7
NG cost (2022)	€/MW h	54.99	–	–	–	–	Jan-Sep 2022 average natural gas cost	S7
Coal cost (2022)	€/MW h	32.14	–	–	–	–	Jan-Sep 2022 average coal cost	S9

2 The PrISMa databases

The PrISMa platform uses various databases (see Figure S1) at each layer:

Materials layer: In this layer, *"material properties"* is the database that contains the required physical properties of the materials together with the thermodynamic adsorption properties. These data can be obtained experimentally or from molecular simulations. These molecular simulations use as input the crystal structures stored in *"material data"*.

Process layer: The process model uses data on equipment (stored in *"equipment data"*) as well as process parameters and operational conditions (stored in *"process data"*) that are characteristic for the case study. The process database uses the information from the case study to provide a list of equipment and operating conditions of the process that best suit the case study.

Techno-Economic Analysis layer: The TEA uses region-specific data on the utilities (stored in *"utilities data"*), including the availability of utilities at the CO_2 source, as well as region-specific data related to the cost of materials and construction (stored in *"economic data"*). The utility database contains all the information necessary for its in-built utility models. Both process and utility databases are linked to the equipment database, which provides the design, input, and cost data necessary to evaluate process and utilities performance and cost.

Life Cycle Assessment layer: The LCA uses region-specific Life Cycle Impact Assessment (LCIA) scores of the background system taken from commercial LCA databases (stored in *"LCIA data"*) and Life Cycle Inventory (LCI) data for the predictive LCA model (stored in *"LCI data"*).

Additionally, in the PrISMa platform, the case studies are defined and characterized in a *"Case Study Database"*. This overarching case study database (not shown in Figure S1) is

developed to provide all parameters needed to describe a case study fully and shared across all layers. This database lists CO_2 sources, CO_2 sinks, and regions worldwide.

Two other aspects of relevance to describe the case study are the economic parameters and the region. Economic parameters are case-specific (e.g., the plant's lifetime might vary depending on the type of industry) and are retrieved from the economic database. The region impacts the carbon footprint of utilities and their cost. The relevant information is obtained by linking the information of the CO_2 source and region to the economic database. Region-specific LCIA scores of the background system are taken from commercial LCA databases and shared across the TEA and LCA layers to ensure consistent data use. LCI data for the predictive LCA model is taken from the literature (for details on the LCIA and LCI databases, see Section 3.4).

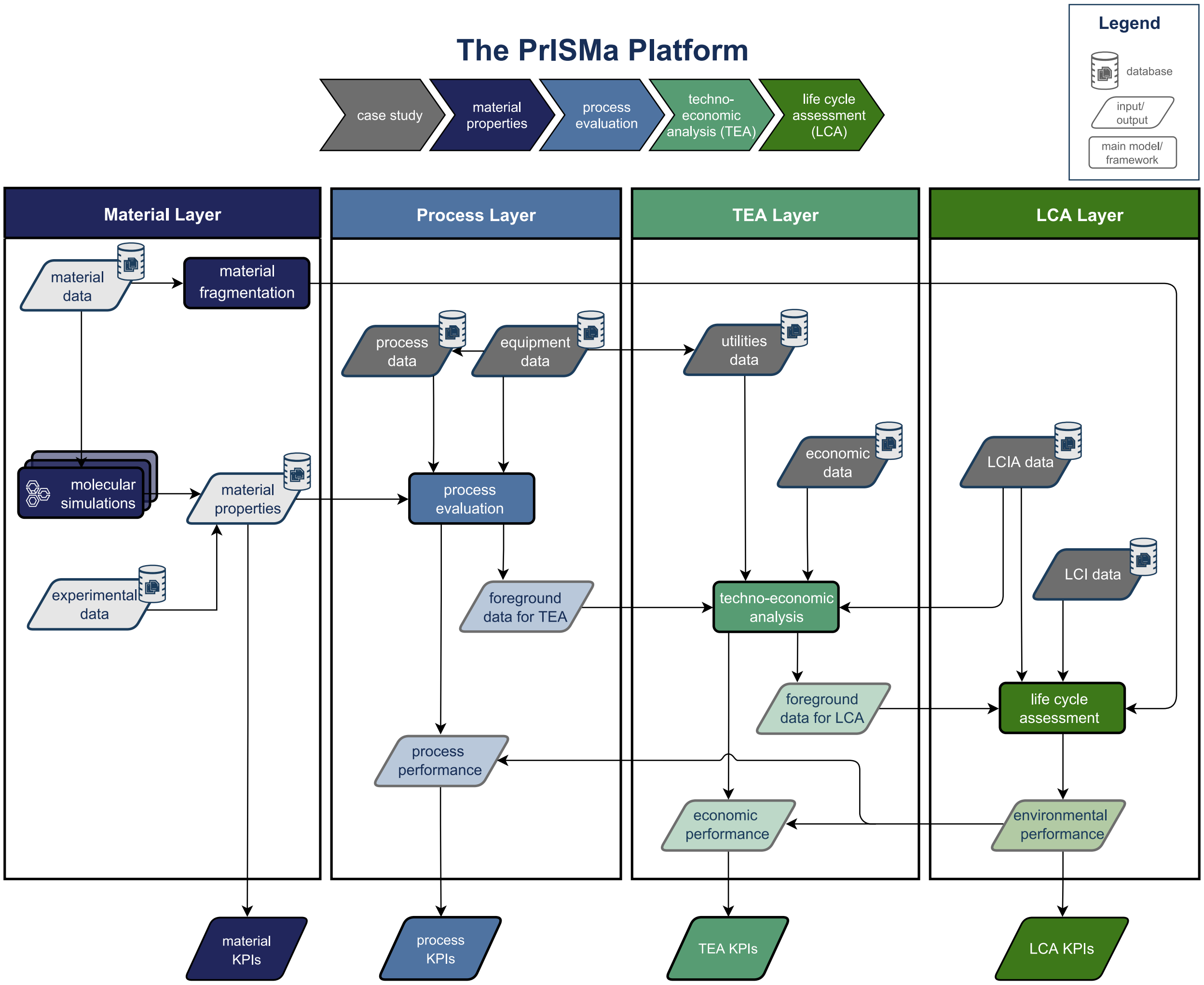

Figure S1| A diagram of the PrISMa platform. The flowchart consists of the processing steps (main models/frameworks that are represented as rectangles, i.e., molecular simulations, process evaluation, techno-economic analysis, and life cycle assessment), the input (data and databases) and output (KPIs) information (represented as rhomboids). Databases in the case study (see process bar banner) are highlighted in dark gray.

3 Methods

In this section, we describe in detail the methods that we use in the four layers of the Process-Informed design of tailor-made Sorbent Materials (PrISMa) platform (see Figure S1).

3.1 Materials layer

In the materials layer, we ensure that all thermodynamic data needed for the process layer are available. This layer differs from the other layers as the workflow that generates the data (*"molecular simulations"*) is a separate workflow that computes the thermodynamic data for all case studies in the PrISMa *"Case Studies Database"* and for all materials in the *"materials data"* database. For those materials for which the required thermodynamic data is available experimentally, the data can be uploaded directly in the *"material properties"* database.

3.1.1 Material properties

The materials screening starts from the material layer, which computes all the material properties necessary for its subsequent process evaluation. In this layer, nanoporous crystalline structures, in this case, MOFs, are retrieved from open-access databases or by accessing experimentally obtained data.

We either use the experimental or the in silico crystal structure as a starting point for those materials without experimental data. From the crystal structure, we can directly compute the properties of the crystal (e.g., crystal density or heat capacity), and we also use the crystal structure as input for a molecular simulation. From the molecular simulation, we obtain the adsorption thermodynamics for CO_2, N_2, and H_2O (see Section 3.1.2).

Crystal structures. We developed a procedure to curate experimental MOF structures for computational screening studies summarized in Figure S2. We start with the Computation-Ready Experimental MOF (CoRE-MOF) database-2019.[S13,S14] This database is a MOF subset of structures from the Cambridge Structural Database (CSD).[S15] The disorder has been

removed for those structures, missing hydrogen atoms have been added, and coordinated and floating solvents in the pores have been removed.

In our curation, as a first step, we keep only those MOF structures, of which the chemical structure contains carbon and a metal. From the remaining structures, we remove all the non-porous structures by constraining our set to structures that have an accessible pore volume to the N_2 probe radii 1.86 Å.[S16] In addition, we remove all structures that contain floating atoms in the crystal structure. After these basic checks, we remove the structures which are not physically realistic. For this, we check chemical structures that contain close contact atoms (pairwise distance smaller than 0.5 Å) or have atoms with large point charges (absolute value larger than 3 A.U. computed using the extended charge equilibration method[S17,S18]). The last step is to remove the duplicate structures. In this work, two MOFs are identified to be identical if their labeled crystal graphs are isomorphic using NetworkX.[S19–S21] The crystal graphs are computed using the bond cutoff distances implemented in VESTA program[S22] and then labeled using atom types. The codes and procedures for performing the calculations are made available in the MOFChecker program.[S23]

To further refine these structures, we use periodic Density Functional Theory (DFT) to optimize the chemical structures. The procedure for these DFT calculations is described in full detail in our previous publications.[S24,S25] In addition, the workflows for these calculations can be accessed via *AiiDa-lsmo* plugin (`https://github.com/lsmo-epfl/aiida-lsmo`), where we published the Automated Interactive Infrastructure and Database for Computational Science (AiiDA),[S26] workflows. All calculations are performed using CP2K.[S27] We use our machine-learning algorithm[S28] to assign the oxidation state of metal atoms in MOF structures, which is used to infer the initial magnetization of the system. The DFT calculations are performed within the generalized gradient approximation (GGA) level of theory using Perdew–Burke–Ernzerhof (PBE) exchange-correlation functional with DFT-D3(BJ) dispersion corrections.[S29,S30] We use GTH pseudopotentials[S31] and DZVP-MOLOPT-SR contracted Gaussian with an auxiliary plane wave basis set.

From these Density Functional Theory (DFT) calculations we obtain the partial charges using the Density Derived Electrostatic and Chemical net atomic charges (DDEC)[S32] protocol.[S17]

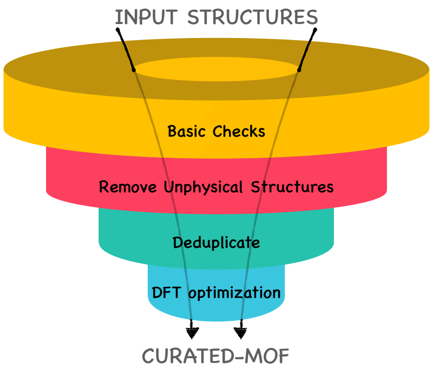

Figure S2| CURATED-MOF. Schematic of the steps used to curate MOF structures.

In silico MOF structures are obtained from the work of Boyd et al.[S33], Park et al.[S34], and Majumdar et al.[S35]. These structures are generated using the ToBasCCo,[S36] PORMAKE,[S37] and ToBaCCo[S38] codes, respectively and optimized using the Universal Force Field (UFF) force-field.[S39] In addition, we used the database assembled by,[S21] which contains about 30,000 structures. The atomic partial charges are generated using the extended charge equilibration method.[S18]

Crystal properties. Physical properties such as crystal density and void fraction are obtained using Zeo^{++}.[S16] For example, the density is computed by summing up the mass of all atoms of the unit cell and dividing it by the volume. The pore volume is computed by filling the pores with a probe molecule (see Ongari et al.[S40] for more details).

Heat capacity. We estimate the heat capacity of each material using the machine learning tool developed by Moosavi et al.[S25]. This method accurately predicts the heat capacity for a MOF, Covalent Organic Framework (COF), or zeolite based on its crystal structure.

3.1.2 Molecular simulations

Force field The adsorption properties are obtained using molecular simulation[S41] and the Automated Interactive Infrastructure and Database for Computational Science (AiiDA).[S26] The crystals are assumed to be rigid in these simulations. The Transferable Potentials for Phase Equilibria Force Field (TraPPE)[S42] force-field is used to describe the CO_2 and N_2 molecules, whereas, the TIP4P/2005 model[S43] is used to describe the H_2O molecules. The crystal atoms are described using the Universal Force Field (UFF) force-field.[S39] The Lorentz-Berthelot combining rules are used to define the interactions between atom pairs.

Henry coefficient and zero loading heat of adsorption Constant Number of Particles, Volume, and Temperature (NVT) Monte Carlo simulations with one adsorbed molecule are used to compute the Henry coefficients and enthalpy of adsorption at zero loading of CO_2, N_2, and H_2O.

Pure component isotherms The pure component adsorption isotherms are computed using Grand-Canonical Monte Carlo (GCMC) simulations using the procedure developed by Moubarak et al.[S44]. In this method, we efficiently compute the adsorption isotherm at a reference temperature of 298.15 K and use the loading-dependent enthalpy of adsorption to compute the isotherms at other temperatures using the Clausius-Clapeyron equation. Moubarak et al.[S44] have shown that this procedure typically gives the binary data within an error of 10 % compared to a GCMC simulation at the same conditions. In this work, we corrected the calculation of the loading dependence of the heat of adsorption for the non-ideal gas behavior of the gas phase. This correction showed a 2–3% improvement in our mixture predictions at high temperatures and pressures.

Mixture isotherms Based on the assumptions in our process model, the adsorption column is segregated into a dry and a wet part (see Section 3.2.1), which requires binary and ternary mixture data, respectively.

Mixture isotherms: Binary We use the pure component isotherms from the GCMC simulations to predict the mixture isotherms using Ideal Adsorbed Solution Theory (IAST).[S45] Moubarak et al.[S44] have shown that at the conditions of all our case studies, the IAST predictions agree with mixture simulations for most materials.

To identify cases for which IAST fails to give an accurate prediction, we perform a single-state point binary GCMC simulation for each material. These binary simulations are carried out at the adsorption conditions of each of the case studies, and we compute the logarithm of the ratio of loading of CO_2 or N_2 as obtained from IAST and GCMC. We find this flag to be much smaller than 0.1 for most materials. If this test shows a higher value, the predictions of the mixture isotherms might not be reliable, and one should do additional simulations of mixture GCMC and fit the data separately.

Mixture isotherms: Ternary The process model (see Section 3.2.1) requires as input the N_2, CO_2, H_2O loading at the adsorption conditions, which are obtained from a ternary GCMC simulation. For the desorption step, the loadings are much lower, and we can assume that the water adsorption is independent of N_2 and CO_2 and therefore follows directly from a pure component H_2O GCMC simulation at desorption conditions. This is then combined with the IAST predictions for N_2 and CO_2.

Water Resistance coefficient To assess which structures are affected the most/least by the presence of water vapor in the flue gas, we perform multi-component GCMC simulations at adsorption conditions of each case study (e.g., 1.013 25 bar and 37 °C for the cement case study).

First, we run binary GCMC simulation corresponding to a dry flue gas and extract the binary CO_2 capacity ($q_{CO_2,\mathrm{dry}}$). Then, we run ternary GCMC calculations on wet basis, extract the ternary CO_2 capacity ($q_{CO_2,\mathrm{wet}}$), and compute the ternary selectivity of CO_2/N_2, CO_2/H_2O and N_2/H_2O. From these results, we compute the ratio of the CO_2 capacity in

the ternary mixture over its capacity in the binary mixture (see Section 6.1.4), which we refer to as Water resistance coefficient (WRC).

3.1.3 Flexibility of the MOFs

In our simulations, we assume that the MOFs are rigid. Flexibility can have different impacts on the adsorption isotherms. We observe a structural change for some MOFs at a particular gas pressure. An example of such a material is MIL-53-NH2 (see Section 5.2.4). We have developed two different computational approaches to detect these structural changes,,[S46,S47] and we would apply these methods to assess the mechanical stability of a material. However, these methods are relatively computationally intensive, and, therefore, we would do this only once we select a material for subsequent synthesis.

Another important effect is that flexibility can impact the “binding site". Witman et al.[S48] have shown that, in the case of an optimal binding site, in which a ligand is exactly at the right position to give a very low binding site, thermal motion can have a significant impact on these binding sites. These binding sites have an important contribution to the Henry coefficients. We used the procedure of Witman et al.[S48] to test whether linker flexibility impacts the Henry coefficients. In this procedure, we perform molecular dynamics simulations of the MOF using LAMMPS[S49] and UFF4MOF.[S50] If flexibility impacts adsorption properties, one would expect to observe large fluctuations in the Henry coefficient along this molecular dynamics trajectory. To quantify this effect, we simulate 100 ps, and we collect 20 snapshots along this trajectory and compute the Henry coefficient for each of these snapshots. The standard deviation of the log of these Henry coefficients is of interest. If this standard deviation is low, one can expect that the assumption we can use rigid crystals is valid. We then rank the materials according to their standard deviation, and we use as a threshold a value of 1 to flag materials for which flexibility significantly impacts the adsorption thermodynamics. This value of 1 corresponds to the accuracy at which our model can predict the experimental data (see Section 5.6).

3.1.4 Output of the layer

The outputs of the materials layer are four KPIs and the foreground data for the process layer. The complete list of all materials outputs is given in Table S5.

3.2 Process layer

The process layer aims to evaluate the technical performance of solid sorbent-based carbon capture technologies. In the process layer, we focus on the operation of a single column. The process accounts for all steps that an adsorption column undergoes for separating the CO_2 from the feed stream and the regeneration of the material. At the next layer, i.e., the TEA, we determine the number of columns required for the specific case study. The process layer is developed flexibly and can be integrated with various process configurations. The process model enables the evaluation of the performance of a material in a pre-defined process configuration and provides essential data (i.e., process KPIs and foreground data listed in Table S5) that are necessary for TEA and LCA. In addition to the operation of the adsorption column, the process layer considers equipment that is necessary for the CO_2 capture process (i.e., vacuum pump) and the delivery of the CO_2 at sink conditions (i.e., compressor). Any pre-treatment of the flue gas before it enters the capture plant is not considered other than the cooling required to bring its temperature down to the temperature of the adsorption step.

3.2.1 Process description

In this work, we consider a 3-step Temperature Swing Adsorption (TSA) and a 5-step Temperature Vacuum Swing Adsorption (TVSA) process (see Figure S3), that consists of the following steps: (i) for TVSA: Adsorption Step (AS), Vacuum Step (VS), Intermediate Heating Step (IHS), Heating Step (HS) and Cooling Step (CS), and (ii) for TSA: AS, HS and CS. It is expected that for less concentrated feed gas streams, the 5-step TVSA process will enable more materials to meet the CO_2 purity requirements for geological storage compared

to a simple 3-step TSA. It has been shown that a TVSA process is efficient in recovering the dilute component.[S51,S52]

In our screening exercise, we consider that our process conditions are fixed. Given these conditions, we screen for the top-performing materials. These conditions include: A T_{FEED} or T_{LOW} of 37 °C, a T_{MED} of 47 °C (10 °C higher than T_{LOW}), and a T_{HIGH} of 120 °C. The selection of T_{LOW} and T_{HIGH} follows from typical adsorption and regeneration temperatures when considering the limitations of the flue gas cooling system in the host plant and the materials' stability at high temperatures, respectively. The value of T_{MED} was set as 10 °C higher than T_{LOW} to avoid losing too much CO_2 in the Intermediate Heating Step (IHS) when heating (and discharging CO_2). From this initial screening, we identify the top-performing materials, and for these materials, we perform an optimization step in which we relax the most important process conditions (see Section 10).

In the current process configurations, the adsorption column is modeled as a packed multi-tubular heat exchanger column where all tubes are filled with the pelletized adsorbent material. The outer column has fixed dimensions, i.e., 8 m length and 1.3 length-to-diameter ratio. The adsorbent material is assumed to be in pellet form with a porosity $\epsilon_{\text{pellet}} = 0.35$ and a porosity of the bed $\epsilon_{\text{bed}} = 0.37$. The column undergoes sequential adsorption, vacuum, heating, and cooling steps. The impact of water on the process performance has been segregated into two major contributions: 1) reduction in working capacity of CO_2 due to competitive co-adsorption, and 2) direct impact on regeneration energy requirements due to cyclic adsorption/desorption of H_2O. The different steps are described in more detail in the next sections.

Adsorption step In the Adsorption Step (AS), the flue gas is assumed to be a mixture of N_2, CO_2, and H_2O, where all gases get adsorbed.

During adsorption, we assume ideal breakthrough behavior, so equilibrium is reached instantaneously (i.e., negligible mass transfer resistances) for both the adsorbed and gas

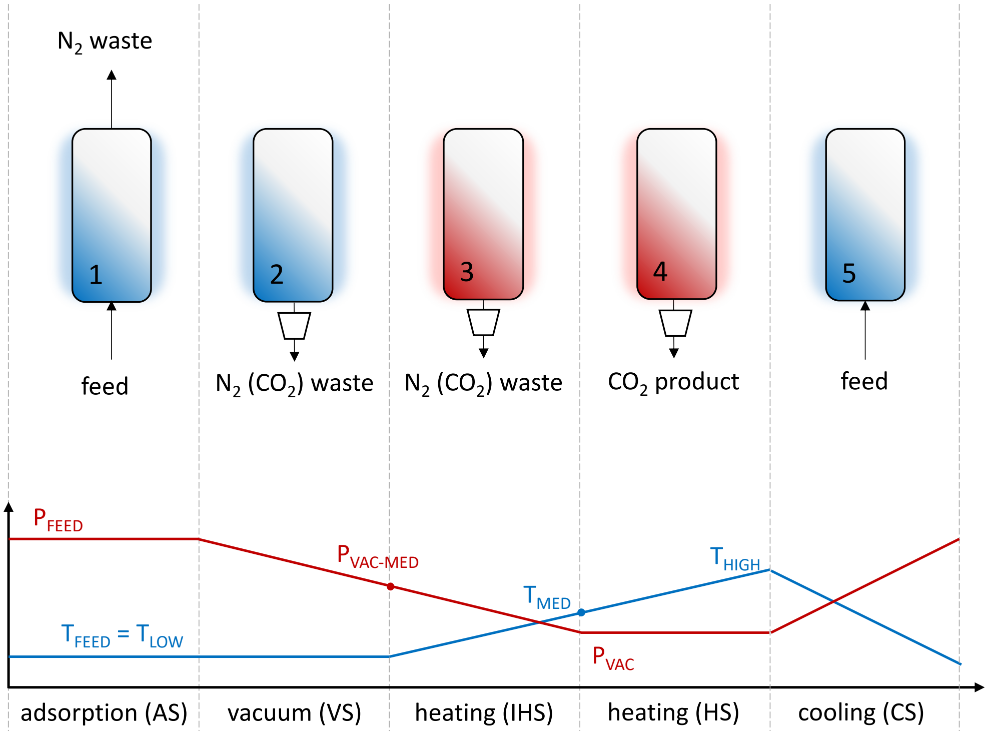

Figure S3| 5-step TVSA process. A schematic illustration of the 5-step TVSA process: Adsorption Step (AS), Vacuum Step (VS), Intermediate Heating Step (IHS), Heating Step (HS), and Cooling Step (CS).

phases, and the adsorption front moves as a step profile through the column. Such an ideal front follows from assuming that we have negligible radial gradients, thermal dispersion, and axial mixing, no pressure drop along the column, and the temperature of the heat exchange fluid is homogeneous along the column. All properties are homogeneous over a phase, i.e., there is no difference in property between the bulk and the boundary within a phase.

The adsorption step is operated until the incipient breakthrough of the CO_2 front from the column. The total amount of CO_2 that is captured during this step is calculated as the difference between the equilibrium loading of CO_2 at the adsorption conditions and that at the start of the adsorption step/end of CS for the respective dry and wet regions in the column. During the breakthrough, a nearly pure N_2 stream leaves the column, which is part of the "waste stream." As shown in Figure S3, during this step, the temperature and pressure of the column remain constant, and we refer to these as the adsorption conditions.

The performance evaluation of the adsorption process is conducted under Cyclic Steady state (CSS) conditions, under which the column undergoes an identical operating trajectory from cycle to cycle.[S53] We first determine the penetration length of the water concentration front (L_{H_2O}, m) under CSS to permit further assessment of moisture-related impact on the average working capacity of the bed.

Generally, the propagation velocities of the respective component concentration fronts within the adsorption column during dynamic cyclic operation are non-equal.[S54,S55] If H_2O travels at a lower velocity relative to CO_2, the bed will segregate into two distinct water-loaded and water-free zones wherein competitive co-adsorption manifests only in the former. This is the case for nearly all materials studied in this work. Experimental breakthrough measurements show that this behaviour applies for numerous CO_2 sorbents, including 13X zeolite[S56,S57] and several MOFs (such as UTSA-16[S58] and CALF-20[S59]). The different speeds of concentration wave propagation serve as the foundation for managing moisture and other strongly adsorbing trace components during adsorptive separations.[S60,S61] The feasibility of preserving relative CO_2 and H_2O concentration front positions under cyclic steady state

operation of adsorptive CO_2 capture processes has been proposed based on numerical process simulations[S62] and corroborated by experimental observations.[S56,S63]

For CSS, there is no accumulation from cycle to cycle; therefore, the amount of water fed into the bed will equal the amount of water recovered by heating/evacuation. This mass balance at CSS is formulated below in which the penetration length of the water concentration front $L_{\mathrm{H_2O,m}}$ is a variable:[S63]

$$\int_0^{L_{\mathrm{H_2O}}} \left(q_{\mathrm{i,H_2O}} - q_{\mathrm{f,H_2O}}\right) \rho_{\mathrm{bulk}} A_{bed}\, \mathrm{d}Z \;= \int_0^{t_{\mathrm{AS}}} \frac{A_{bed} u_{\mathrm{f}} P_{\mathrm{w}}}{RT}\, \mathrm{d}t \;= t_{\mathrm{(AS)}}\, \dot{n}_{\mathrm{feed}}\, y_{\mathrm{H_2O,feed}} \tag{S1}$$

A_{bed} ($\mathrm{m^2}$) is the cross-sectional area of the column, P_{w} (kPa) is the partial pressure of water in the flue gas, ρ_{bulk} ($\mathrm{kg\,m^{-3}}$) is the bulk density of the sorbent, and u_{f} ($\mathrm{m\,s^{-1}}$) is the velocity of the feed flue gas. $q_{\mathrm{i,H_2O}}$ and $q_{\mathrm{f,H_2O}}$ ($\mathrm{mol\,kg^{-1}}$) denote the local solid phase water loadings at the beginning and end of the adsorption step, respectively. $y_{\mathrm{H_2O,feed}}$ is the mole fraction of H_2O in the feed (flue gas), and $\dot{n}_{\mathrm{feed}}$ ($\mathrm{mol\,s^{-1}}$) is the molar flow rate of the feed stream. The integral $\int_0^{L_{\mathrm{H_2O}}} (q_{\mathrm{i,H_2O}} - q_{\mathrm{f,H_2O}})\,\mathrm{d}Z$ may be interpreted as the cyclic working capacity for water.

An idealized analysis can be performed by assuming shock front profiles for adsorption and desorption and instantaneous mass transfer between the fluid and the adsorbent. Then, the integral may be estimated by:[S64]

$$\int_0^{L_{\mathrm{H_2O}}} (q_{\mathrm{i,H_2O}} - q_{\mathrm{f,H_2O}}) \rho_{\mathrm{bulk}} A_{bed}\, \mathrm{d}Z \;= \rho_{\mathrm{bulk}} A_{bed} L_{\mathrm{H_2O}} (q_{\mathrm{H_2O,feed}} - q_{\mathrm{H_2O,des}}) \tag{S2}$$

where $(q_{\mathrm{H_2O,feed}} - q_{\mathrm{H_2O,des}})$ is the difference between the equilibrium uptakes at the feed and final desorption conditions. Under realistic operation conditions, spreading at either or both the adsorption and desorption concentration fronts is anticipated, reducing the separation performance.[S65] In addition, we assume equilibrium single-component H_2O uptake is representative of the mixture co-adsorption uptake of H_2O. This is a reasonable assumption for some materials (e.g., zeolitic sorbents),[S66] but exceptions have been observed. For exam-

ple, suppression of water uptake by CO_2 for CALF-20[S59] has been observed at CO_2partial pressures of ~97 kPa, but these conditions are way in excess of the range considered in the present case studies (4 to 20 kPa). Observing that the water content at the adsorber inlet for our various case studies is near saturation levels, we further approximate the feed uptake by the estimated geometric saturation loading of H_2O from Zeo^{++} calculations. After applying the above assumptions, solution of Equation (S1) yields a simplified expression for L_{H_2O}:

$$L_{H_2O} = \frac{\frac{t_{(AS)} A_{bed} u_f P_w}{RT}}{\rho_{bulk} A_{bed} (q_{H_2O,sat,geom} - q_{H_2O,des})} = \frac{t_{(AS)}\, \dot{n}_{feed}\, y_{H_2O,feed}}{A_{bed} WC_{H_2O}} \tag{S3}$$

In Equation (S3), we make a conservative assumption and use the duration of the adsorption step $t_{(AS)}$ as determined for the dry case (see Section 3.2.2) to approximate $t_{(AS)}$, thus avoiding an iterative solution.

We can derive a similar expression as Equation (S3) for L_{CO_2} by combining Equations (S6), (S40) and (S42) to give:

$$L_{CO_2} = \frac{n_{CO_2,\,out\,(HS)}}{A_{bed}\, WC_{CO_2}} \approx \frac{n_{CO_2,\,in\,(AS)}\, \text{Recovery}_{CO_2}}{A_{bed}\, WC_{CO_2}} = \frac{t_{(AS)} \dot{n}_{feed}\, y_{CO_2,feed}}{\eta_{sat}} \frac{\text{Recovery}_{CO_2}}{A_{bed}\, WC_{CO_2}} \tag{S4}$$

See Section 6.2.4 for more details on how the CO_2 working capacity is calculated considering the impact of water sorption.

Vacuum step The Vacuum Step (VS) together with the Intermediate Heating Step (IHS) and Heating Step (HS) complete the regeneration process, but we do not collect the product until the HS starts. The aim of the VS is to increase the purity of the product (CO_2 rich) stream. At the end of the AS, the void space in the column[1] contains gas with a composition that is equal to that in the feed gas stream. During the VS, the bed is exposed to a rapid (instantaneous) vacuum, and as a result, we remove (part of) the gas phase, which goes to the waste stream. During this step, the adsorbed phase is assumed to be "frozen" and, therefore,

[1]We assume that the total void space is the sum of the void space in the pellets and the void space in the bed.

it is expected that the quick change in the bulk pressure will not affect the adsorbed phase concentration. Santori et al.[S52] and Charalambous[S67] have given a detailed description of this step.

Once we stop applying the vacuum, the column will equilibrate again but at a lower total pressure than the initial feed pressure. We can compute the new partial pressures in the gas phase and the new loadings in the adsorbed phase from the mass balance and adsorption thermodynamics. In our model, we follow a step-wise approach in which we apply this vacuum step several times until the rate of pressure change calculated from the last three steps is below a set value. The final equilibrium pressure in the bed is $P_{\mathrm{VAC-MED}}$ (see Figure S3, step 2). During these vacuum steps, the removed gas phase is rich in N_2, and we increase the CO_2 concentration in the column. Hence, the purity of our product stream increases, but as we add some CO_2 to the waste stream, we lower the recovery.

Intermediate Heating Step We can further increase the purity of our product if we heat the bed to an intermediate temperature. At this higher temperature, the gas phase will contain a higher concentration of the weaker bound component (i.e., N_2). This heating process is conducted in a series of ten consecutive steps until we reach a temperature T_{MED} (see Figure S3, step 3). At the start of the intermediate heating step, the pressure in the column will be the final pressure of the Vacuum Step (VS), and the pressure is further decreased during the Intermediate Heating Step (IHS) to the final vacuum pressure, P_{vac}. The pressure decrease happens as a step change during the first sequential step in the temperature profile and is kept constant at P_{vac} during the remaining steps.

Heating Step During the Heating Step (HS), the bed is further heated in ten steps to the final heating temperature, T_{HIGH} (see Figure S3, step 4). In this step, we have an open end for collecting the CO_2 rich product at constant vacuum pressure.

Cooling Step During the Cooling Step (CS), the bed is pressurized and cooled down in ten steps by flowing the feed stream through an open end of the bed. This results in a pressure increase from the vacuum to the pressure of the feed stream P_{FEED} and simultaneous cooling of the bed to the temperature of the feed stream T_{FEED} (see Figure S3, step 5). During this step, no gas leaves the column. Hence, all the feed gas that enters the bed and gets adsorbed stays in the bed. In this step, the total amount of flue gas we must let in to achieve the desired temperature and pressure in the column is unknown. This amount is then calculated from the mass balance, i.e., the difference between the amount of CO_2 and N_2 at the end of the HS and the end of the CS. At the end of the CS, the partial pressure of CO_2 in the gas phase will be (significantly) lower than its partial pressure in the flue gas.

3.2.2 Cycle time

For the process and subsequent Techno-Economic Analysis (TEA) and Life Cycle Assessment (LCA) evaluation, it is important to compute the effective cycle time of the carbon capture process. We define our t_{cycle} (h) as the total time required to operate all steps that a single column undergoes, i.e., is the sum of the five steps in our TVSA process:

$$t_{\text{cycle}} = t_{(\text{AS})} + t_{(\text{VS})} + t_{(\text{IHS})} + t_{(\text{HS})} + t_{(\text{CS})}. \tag{S5}$$

The time to operate the AS is given by the time it takes for the adsorption front to break through, which can be obtained from the total amount of CO_2 that is captured during the AS:

$$t_{(\text{AS})} = \frac{\eta_{\text{sat}}\, n_{\text{AS,CO}_2}}{\dot{n}_{\text{feed}}\, y_{\text{feed}}}, \tag{S6}$$

where η_{sat} is the saturation efficiency of the column, which is set to one for this study, $n_{\text{AS,CO}_2}$ is the total number of moles of CO_2 captured in the AS, y_{feed} is the mole fraction of CO_2 in the feed (flue gas), and $\dot{n}_{\text{feed}}$ (mol s^{-1}) is the molar flow rate of feed stream.

The time to operate the VS is determined by the total volume of gas that is removed during the vacuum step ($V_{\mathrm{VS,out}}$) over the flux of the pump:

$$t_{(\mathrm{VS})} = \frac{V_{\mathrm{VS,out}}}{v_{\mathrm{out}}\, A_{\mathrm{tubes}}}, \tag{S7}$$

in which v_{out} ($\mathrm{m\,s^{-1}}$) is the velocity of the gas leaving the column and A_{tubes} ($\mathrm{m^2}$) is the total cross-sectional area of the tubes.

The heating and cooling times associated with IHS, HS, and CS follow from the time it takes to heat/cool the bed from an initial temperature T_0 to a final temperature T_1.

$$t_{(\mathrm{IHS}),\,(\mathrm{HS}),\,(\mathrm{CS})} = \frac{c_{\mathrm{p,\,bed}}\, V_{\mathrm{bed}}\, |T_1 - T_0| + H_{\mathrm{ads}}}{U\, A\, V_{\mathrm{bed}}\, (T_{\mathrm{HEX}} - \frac{T_1 + T_0}{2})}, \tag{S8}$$

where $c_{\mathrm{p,\,bed}}$ ($\mathrm{kJ\,m^{-3}\,K^{-1}}$) is the heat capacity of the packed bed, which is calculated using $c_{\mathrm{p,\,solid}}$ from material layer; V_{bed} ($\mathrm{m^3}$) is the bed volume; H_{ads} (kJ) is the (multi-component) enthalpy of adsorption; U is the heat transfer resistance ($\mathrm{kJ\,m^{-2}\,s^{-1}\,K^{-1}}$), which is assumed to be constant, i.e., independent of composition, temperature, and pressure of the fluid and adsorbed phase, and fixed for all the materials. A ($\mathrm{m^2\,m^{-3}}$) refers to the heat transfer area and T_{HEX} (K) is the temperature of the heat exchanger media.

3.2.3 Energy requirements

In the process layer, we compute the specific thermal energy for heating, the specific thermal energy for cooling, and the specific electrical energy.

Specific thermal energy for heating The specific thermal energy for heating, q_{th} in $\mathrm{kJ\,mol^{-1}}$, is defined as the sum of the total (multi-component) enthalpy of adsorption, H_{ads}

(kJ), and the sensible heat, Q_{sens} (kJ), in the Intermediate Heating Step (IHS) and Heating Step (HS) per amount of CO_2 collected in the HS, $n_{\text{HS,CO}_2}$,

$$q_{\text{th}} = \frac{Q_{\text{sens}} + H_{\text{ads}}}{n_{\text{HS,CO}_2}}. \tag{S9}$$

where Q_{sens} is the sum of heat required to bring the adsorbent from the feed temperature T_{LOW} to the intermediate temperature, T_{MED}, in the IHS, and the heat required to further increase the temperature of the bed to the desorption temperature, T_{HIGH}, in the HS (see Figure S3). In this calculation, we assume that the contribution of the gases in the adsorbed and bulk-gas phase is negligible (see Moosavi et al.[S25]):

$$Q_{\text{sens}} = m_{\text{solid}} \int_{T_{\text{LOW}}}^{T_{\text{HIGH}}} c_{\text{p, solid}}\, \mathrm{d}T = m_{\text{solid}}\, c_{\text{p, solid}} \left[(T_{\text{MED}} - T_{\text{LOW}}) + (T_{\text{HIGH}} - T_{\text{MED}})\right] \tag{S10}$$

Here, m_{solid} (kg) is the total mass of the adsorbent in the bed. The heat capacity of the solid $c_{\text{p, solid}}$ ($\text{kJ}\,\text{kg}^{-1}\,\text{K}^{-1}$) is obtained from the foreground data of the material layer (see Section 3.1.1).

To compute the energy needed to undo the adsorption (H_{ads}), we assume that the enthalpy of adsorption of each component is independent of the composition and loading. With these assumptions, H_{ads} follows from:

$$H_{\text{ads}} = \sum_{i=1}^{N_{\text{comp}}} -\Delta h_i\, n_i \tag{S11}$$

where n_i is the total number of moles of component i adsorbed in the AS and for Δh_i ($\text{kJ}\,\text{mol}^{-1}$), we use the average pure component isosteric heat of adsorption, which is obtained the data in the material layer (see Section 3.1.2).

The thermal energy associated with regenerating the water-loaded sorbents, H_{H_2O} (kJ),was calculated by an analogous expression to Equation (S11).

$$H_{H_2O} = -\Delta(h_{H_2O}\, n_{H_2O}) = -\Delta h_{H_2O} \cdot \rho_{\text{bulk}}\, AL_{H_2O} \cdot \left(q_{H_2O,\text{ sat, geom}} - q_{H_2O,\text{ des}}\right) \tag{S12}$$

The change in the solid phase loading of water, Δn_{H_2O}, was derived by weighting the approximated change in uptake $(q_{H_2O,\text{sat,geom}} - q_{H_2O,\text{des}})$ (mol kg^{-1}) by the total mass of sorbent in the water-loaded zone ($\rho_{\text{bulk}} AL_{H_2O}$, kg). Meanwhile, the pure component isosteric heat of adsorption for H_2O, $-\Delta h_{H_2O}$ (kJ mol^{-1}), is obtained from molecular simulations (see Section 3.1.2).

Specific thermal energy for cooling In the process, cooling is needed in the following steps, each demanding distinct amount of energy:

- $Q_{\text{Therm Cool}}$ (kJ), is the thermal cooling energy required in the Cooling Step (CS);
- Q_{DCC} (kJ), is the cooling energy required in the direct contact cooler (DCC), where the flue gas stream is cooled down to the temperature required in the Adsorption Step (AS), T_{FEED} (K), and where part of the water is condensed out, $n_{\text{cond water}}$;
- $Q_{\text{condenser}}$ (kJ), is the cooling energy required in the condenser, where the CO_2 rich product stream collected in the Heating Step (HS) is cooled down to T_{cool} (K) prior to entering the compression train, and;
- Q_{compr} (kJ) is the cooling energy required in the intercoolers located between compressor stages. A compressor train is needed for those case studies in which the CO_2 sink is geological storage, and the CO_2 product needs to be pressurized to 110 bar. For TVSA process configurations, a compressor is also needed to bring the CO_2 rich product stream from P_{vac} to atmospheric pressure before it is sent to the compressor train.

Hence, the total specific thermal energy for cooling, $q_{\text{th, cooling}}$ (kJ mol^{-1}), can be calculated by adding all the different terms and normalizing them by the amount of CO_2 collected in the Heating Step (HS), n_{HS,CO_2}, mol, as follows:

$$q_{\text{th, cooling}} = \frac{Q_{\text{Therm Cool}} + Q_{\text{compr}} + Q_{\text{DCC}} + Q_{\text{condenser}}}{n_{\text{HS},CO_2}}. \tag{S13}$$

The thermal energy in the Cooling Step (CS) is computed similarly to the specific thermal energy for heating (see Section 3.2.3); the sum of the sensible heat, Q_{sens} (kJ), and (multi-component) enthalpy of adsorption, H_{ads} (kJ):

$$Q_{\text{Therm Cool}} = Q_{\text{sens}} + H_{\text{ads}} \tag{S14}$$

The thermal energy required to cool down the flue gas feed stream from $T_{\text{flue gas}}$ to T_{FEED} is given by:

$$Q_{\text{DCC}} = [n_{\text{flue gas, in}}\, h_{\text{fg}}(T_{\text{flue gas}}) - n_{\text{flue gas, out}}\, h_{\text{fg}}(T_{\text{FEED}})] + n_{\text{cond water}}\, h_{\text{latent}}(T_{\text{FEED}}). \tag{S15}$$

Here $n_{\text{flue gas, in}}$ and $n_{\text{flue gas, out}}$ (mol) are the number of moles of flue gas entering and exiting the direct contact cooler; h_{fg} (kJ mol^{-1}) is the enthalpy of the flue gas, $T_{\text{flue gas}}$ (K) is the temperature of the flue gas leaving the host plant; T_{FEED} (K) is the temperature required at the Adsorption Step (AS); $n_{\text{cond water}}$ (mol) is the number of moles of condensed water; and h_{latent} (kJ mol^{-1}) is the latent heat of condensation at a given temperature.

The thermal energy required to cool the CO_2 rich product stream from the desorption temperature, T_{HIGH}, to T_{cool} is given by:

$$Q_{\text{condenser}} = [n_{\text{product, in}}\, h_{\text{gas}}(T_{\text{HIGH}}) - n_{\text{product, out}}\, h_{\text{gas}}(T_{\text{cool}})] + n_{\text{cond water}}\, h_{\text{latent}}(T_{\text{cool}}). \tag{S16}$$

Here $n_{\text{product, in}}$ and $n_{\text{product, out}}$(mol) are the number of moles of product entering and exiting the condenser; h_{gas} (kJ mol^{-1}) is the enthalpy of the mixture in the product stream, T_{cool} (K) is the outlet temperature, which is 313 K for our cooling system.

The cooling duty of the compressor intercoolers is given by:

$$Q_{\text{compr}} = \sum_{\text{j}=1}^{Nc} \left[n_{2\text{j}}\, h_{\text{gas}}(T_{2\text{j}}) - n_{2\text{j}+1}\, h_{\text{gas}}(T_{\text{cool}}) + n_{\text{cond water}}\, h_{\text{latent}}(T_{\text{cool}}) \right]. \tag{S17}$$

Here, $n_{2\text{j}}$ and $n_{2\text{j}+1}$ (mol) are the number of moles of each of the mixture components in the product stream entering and leaving the j-intercooling stage, respectively; $T_{2\text{j}}$ (K) is the temperature of the fluid downstream of the compressor stage j, and; T_{cool} (K) is the intercooling temperature, which is 313 K for our cooling system.

The amount of condensed water, $n_{\text{cond water}}$ (mol), and molar composition of the outlet stream in a given cooling step, i.e., DCC, cooler, compressor intercoolers, when the gas stream is cooled down below the dew temperature, T_{dew}, follows directly from a mass balance around the cooler and water knock-out drum and is evaluated as:

$$n_{\text{cond water}} = n_{\text{in}} - n_{\text{out}}, \tag{S18}$$

$$n_{\text{out}} = n_{\text{in}} \frac{(1 - y_{\text{H}_2\text{O, in}})}{(1 - y_{\text{H}_2\text{O, sat}})}, \tag{S19}$$

$$y_{\text{H}_2\text{O, out}} = y_{\text{H}_2\text{O, sat}}, \tag{S20}$$

$$y_{\text{i, out}} = y_{\text{i, in}} \frac{(1 - y_{\text{H}_2\text{O, sat}})}{(1 - y_{\text{H}_2\text{O, in}})} \text{ for i} \neq \text{H}_2\text{O}, \tag{S21}$$

where n_{in} and n_{out} (mol) are the inlet and outlet total number of moles to a given cooling stage, respectively; $y_{\text{i, in}}$ and $y_{\text{i, out}}$ are the molar fraction of a component i in the inlet and outlet streams; $y_{\text{H}_2\text{O, in}}$ and $y_{\text{H}_2\text{O, out}}$ are the molar fractions of water in the inlet and outlet streams; and $y_{\text{H}_2\text{O, sat}}$ is the molar fraction of water at saturation in the outlet stream, which

is calculated as the saturation (vapor) pressure of water at T_{cool}, $P_{\text{sat}}(T_{\text{cool}})$(bar), divided by the total pressure of the gas stream, P_{total} (bar), according to:

$$y_{\text{H}_2\text{O, sat}} = \frac{P_{\text{sat}}(T_{\text{cool}})}{P_{\text{total}}}. \tag{S22}$$

Specific electrical energy The specific electrical energy, w_{el} (kJ mol^{-1}), is defined as the energy needed to operate the vacuum pump during the VS and IHS of the capture process and to operate the compressor used to pressurize the product stream that leaves the capture plant to the conditions required for geological storage:

$$w_{\text{el}} = \frac{W_{\text{el, vacuum}} + W_{\text{el, compression}}}{n_{\text{HS,CO}_2}}. \tag{S23}$$

We compute each term using the expression of the polytropic compression work (a reversible process which involves both heat and work transfer)[S68] shown in Equation (S24).

$$W_{\text{el,vacuum}} = \frac{1}{\eta_p}\frac{n}{n-1}\, n_1\, R\, T_1 \left(\left(\frac{P_2}{P_1}\right)^{\frac{n-1}{n}} - 1\right), \tag{S24}$$

$$\left(\frac{n-1}{n}\right) = \left(\frac{\gamma_{\text{mix}}-1}{\gamma_{\text{mix}}}\right)\cdot\frac{1}{\eta_p}. \tag{S25}$$

Here, η_p is the polytropic efficiency of the equipment (i.e., vacuum pump or compressor), n is the polytropic coefficient, γ_{mix} is the ratio of the heat capacity at constant pressure to heat capacity at constant volume of the mixture, and '1' and '2' indices correspond to the stream upstream and downstream of the equipment, respectively.

For a N_c-multistage compression system that is composed of N_c compressor alternated with N_c intercoolers, one divides the thermodynamic path from suction to discharge pressure and temperature into a number of steps with the same (polytropic) efficiency. In each j-

compression stage, the fluid is compressed from the state (2j-1) to the state (2j), and the fluid goes through a cooling process from the state 2j to the state (2j+1). The compression work is calculated as follows:

$$W_{\mathrm{el,compression}} = \sum_{\mathrm{j=1}}^{N_\mathrm{c}} \frac{1}{\eta_p} \frac{n}{n-1} n_{\mathrm{2j-1}}\, R\, T_{\mathrm{2j-1}} \left(\left(\frac{P_{\mathrm{2j}}}{P_{\mathrm{2j-1}}} \right)^{\frac{n-1}{n}} - 1 \right), \tag{S26}$$

$$n_{\mathrm{2j}} = n_{\mathrm{(2j+1)}} \, \frac{(1 - y_{\mathrm{H_2O,(2j)}})}{(1 - y_{\mathrm{H_2O,\ sat\ at\ T_{cool}}})}, \tag{S27}$$

$$P_{\mathrm{2j}} = P_{\mathrm{2j+1}}, \tag{S28}$$

$$T_{\mathrm{2j-1}} = T_{\mathrm{2j+1}} = T_{\mathrm{cool}}. \tag{S29}$$

Here, T_1 and P_1 are the suction temperature and pressure, respectively, and T_{2Nc} and P_{2Nc} are the discharge temperature and pressure. Iterative calculations are employed to determine the number of compression stages, N_c, ensuring that the exit temperature from the initial compression stage remains below 200°C.[S69] The process assumes a constant pressure ratio for each compression stage. Assumptions include constant polytropic efficiency η_p for each compression stage, ideal gas behavior of the working fluid with constant heat capacity, and neglecting pressure drop in the intercoolers (Equation (S28)).

3.2.4 Validation of the layer

Our 3-step TSA model is based on the approach of Ajenifuja et al.[S70]. We used the authors' results to validate our model. For this validation, we considered the same case study as the authors, i.e., the CO_2 capture from post-combustion carbon capture systems. We also consider a binary mixture of CO_2 and N_2 adsorbed on zeolite 13X with a CO_2 molar fraction of 0.12 in the feed stream. All results were in good agreement in terms of purity, recovery, working capacity, and specific thermal energy.

The validated TSA model was then modified to the 5-step TVSA model presented above. The performance of this model was compared against an in-house detailed model developed in gPROMS[S71] and described by Young et al.[S51].

The modifications to model the water distribution in the column are derived from the strategy of Webley and coworkers.[S63] They reflect the qualitative behavior observed from dynamic wet experiments[S57,S58] and cyclic separation involving wet mixtures.[S56,S63] The penetration length concept has also been used in tandem with detailed numerical modeling of PVSA cycles by Leperi et al.[S64].

3.2.5 Output of the layer

The outputs of the process layer are eight process-KPIs and TEA foreground data. The complete list of all process outputs is in Table S5.

3.3 Techno-Economic Analysis layer

In the Techno-Economic Analysis (TEA) layer, we provide estimates of economic KPIs as well as foreground data required for the LCA layer.

3.3.1 TEA system boundaries

For the TEA calculations, the first step is to use the output of the process layer for scaling out from the process design (i.e., the operation of a single column) to the capture plant design. The capture plant boundaries are illustrated in Figure S4. Similar to the process layer, the pre-treatment of the flue gas and the transport and storage/utilization processes are excluded.

3.3.2 CAPEX calculation

The Capital Expenditure (CAPEX) of the capture plant is calculated by following the guidelines of Woods, which are based on the Bare Module method for developing screening cost

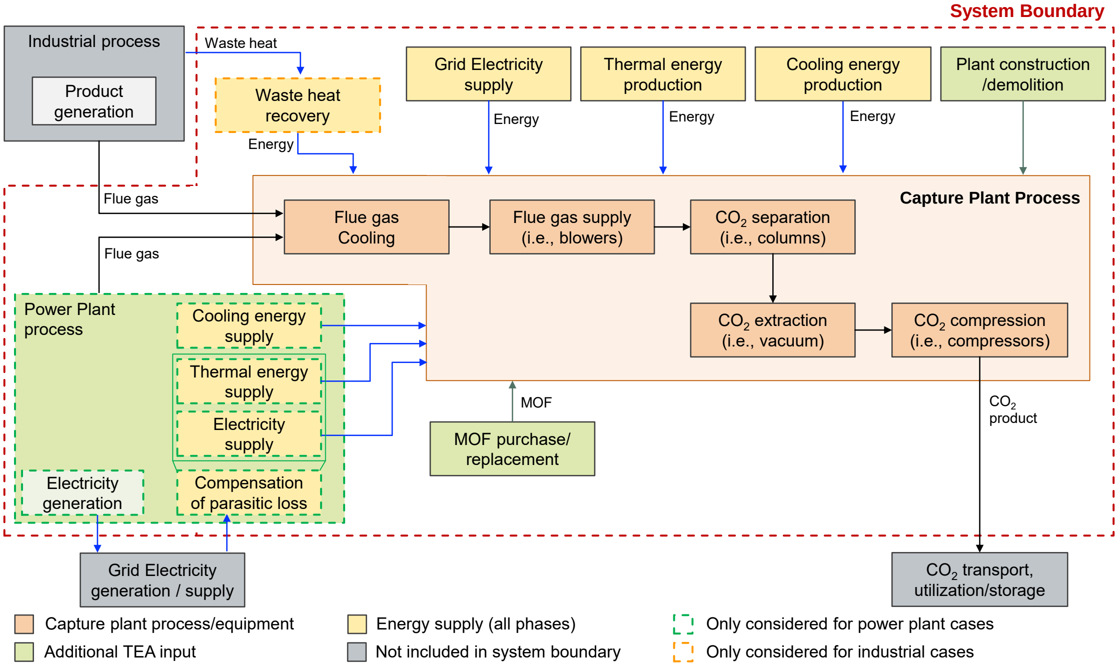

Figure S4| TEA system boundaries. Schematic illustration of the system boundaries considered within the TEA of the adsorption-based carbon capture process.

estimates. In this method, the Free-On-Board (FOB) cost of a specific piece of equipment is multiplied by factors that account for all the concrete, piping, electrical, insulation, painting, and supports needed in a space approximately 1 m out from the sides of the equipment. This whole space is called a module, and the factors are referenced to as the Labour and Materials (LM) costs. The key equipment required at the screening level consists of the adsorption column and the compressor and/or vacuum pumps necessary to maintain the required conditions inside the adsorption column.

The Total Direct Cost (TDC) is then estimated by adding the FOB cost multiplied by the equipment-specific LM factors for all relevant equipment (Section 3.3.2). Next, the Engineering, Procurement and Construction (EPC) cost is estimated by multiplying the TDC by the process contingencies (P_1), indirect costs (P_2), and project contingencies (P_3) factors (Section 3.3.2), respectively. Finally, the Capital Expenditure (CAPEX) is estimated

by multiplying the EPC cost by a factor that takes into account spare parts, and owners cost, etc. (P_4) (Section 3.3.2).

$$\text{TDC} = \sum \text{FOB} \cdot \text{LM} \tag{S30}$$

$$\text{EPC} = (1 + P_1) \cdot (1 + P_2) \cdot (1 + P_3) \cdot \text{TDC} \tag{S31}$$

$$\text{CAPEX} = (1 + P_4) \cdot \text{EPC}, \tag{S32}$$

with $P_1 = 0.10$, $P_2 = 0.14$, $P_3 = 0.30$, and $P_4 = 0.07$.

For these calculations, Aspen Capital Cost Estimator[S72] and the database from Woods[S73] have been used as a source of FOB and LM costs. The work of Rubin et al.[S74] has been used as a source for process and project contingencies.

3.3.3 OPEX calculation

The operational expenditure, OPEX, is calculated annually and considers all plant operation costs. It is calculated as the sum of fixed operational costs, variable operational costs, and adsorbent replacement. Fixed costs, such as labor, maintenance, and insurance, are estimated following the guidelines of EBTF.[S75] Variable costs are material dependent and include materials and costs of utilities that need to be generated in addition to the available ones in the host plant (see Section 3.3.4). The adsorbent replacement cost, which will depend on material durability, is calculated based on the assumption that the adsorbent is replaced every five years[S76] through the lifetime of the capture plant. This cost is allocated to the year when the adsorbent needs to be replaced. As there are huge uncertainties regarding the costs of MOF synthesis, our TEA model assumes the price for all MOFs to be the same (30 € kg^{-1}). This value is based on a recent analysis of the cost of some MOFS.[S77] In Section 8.4.3, we study the impact of the price on the overall cost of the process. In addition, we obtained some insights into the cost associated with the metal from the life-cycle assessment KPIs.

Equation (S33) shows how the lifetime discounted expenditure of the capture plant is calculated.

$$\text{OPEX} = \sum_{yr=1}^{\text{Plant lifetime}} \frac{\text{OPEX}_{\text{fixed}}(yr) + \text{OPEX}_{\text{variable}}(yr) + \alpha_{yr,\text{lifetime}} C_{\text{material, repl}}}{(1 + DCF)^{yr}} \tag{S33}$$

Here, the summation runs over the years the plant is operating, $\alpha_{yr,\text{lifetime}}$ is one if the year is a multiple of the lifetime of the adsorbent; otherwise, zero. $C_{\text{material, repl}}$ (M€) refers to the cost of material replacement, and DCF is the discounted cash flow rate.

While the OPEX defined in (S33) reflects the costs from a plant's operator perspective, the overall cost can be larger if the systems perspective is taken into account. This perspective is particularly important for power plant generation technologies because the operation of the carbon capture plant leads to a loss of electricity (i.e., parasitic load) that has to be compensated by the system. Thus, in our PrISMa platform, we consider an additional definition of the operational cost that accounts for this compensation by the system, where we assume the electricity market price for electricity production. For industrial plants, the system-based operational cost is equal to the OPEX defined in Equation (S33) if the production of the industrial site is not affected by the integration of the carbon capture plant, and no product loss has to be compensated by the system.

3.3.4 Utilities calculation

In Equation (S33), the operational variable costs include the costs of the utilities. Calculating this cost requires assumptions on how the utilities are supplied to the capture plant. In the PrISMa platform, we have developed a utility database (see Figure S1) that allows for the creation of different scenarios.

There are two distinct relevant situations:

1. Utilities (heat, cooling, and power) are available at the CO_2 source (the host plant): In some cases, the use of these utilities in the capture plant leads to a reduction in

production. This is the case of power plants, where it has been assumed that the fuel input to the power plant is the same with and without carbon capture. This results in a reduction of power output and net efficiency when the capture plant is integrated. In other cases, production is not affected by the use of utilities. For instance, some industrial sites (e.g., cement plants and oil platforms) might have waste heat streams that can be used in the capture plant without any impact on production.

2. Utilities (heat, cooling, and power) are not or insufficiently available at the CO_2 source: In this case, the utilities need to be generated at the site or purchased, and they have to be included in the overall capital and operational expenditure of the plant.

The case study description provides scenarios on how the utilities are supplied to the capture plant. The generic utility integration method then follows three steps:

1. **Availability of utilities and limitations:** The database contains upper and lower limits of energy flows, pressure, and temperature for available utilities. A scenario for estimating carbon footprint and primary energy use is provided for each utility. These scenarios are region-dependent. Each case in the database is linked to a region that considers local energy costs and local carbon footprints.

2. **Estimation of required energy flows:** These flows are computed and compared with available utilities at CO_2 source. This step evaluates how much energy can be supplied from the CO_2 source and how much energy needs to be purchased or generated. The current database contains models for fuel-fired boilers, electrical boilers, and steam generation from waste heat.

3. **Integration or generation of utilities:** Utilities can be either integrated from the CO_2 source or generated by adding extra equipment.

For every material, the overall utility consumption is estimated. If additional utility generation must be included, the TEA framework follows a pre-defined case study-dependent

list of utilities until the requirements for all utilities are satisfied. In each case study, at least one utility is unlimited to guarantee the energy supply for each material.

3.3.5 Validation of the layer

Sorbent-based carbon capture is an innovative emerging technology that is in the early stages of development, and, as a consequence, not all design and cost data are available in the standard resources. For those components whose data is missing, the design and cost results are calibrated with data from other industrially available components that share a high degree of design similarity to the ones in our framework. In this context, design similarity is established by only choosing cost models and data for comparison from components with similar upper and lower design limits (pressure and temperature) and similar limits in their main dimensions (e.g., diameter and length). Commercial software has been used to generate the data when such data cannot be found in the literature. Table S4 shows a list of the sources and models used to calibrate the main equipment components of the techno-economic layer.[S78]

Table S4: **Main equipment components:** Data, models, and correlations used to calibrate the main equipment components in the techno-economic layer

Equipment	Design models/ correlations	Performance data	Cost correlations	Cost Data
Turbine/ Power plant	S79,S80	S75	[-]	S75
Compressor	S72	S78,S81	[-]	S75,S78,S81
Adsorption Column	S72	From process layer	S73	[-]
Boilers, coolers, and other heat exchangers	S72	[-]	S73	[-]
Cooling tower	[-]	[-]	[-]	S75
Direct Contact Cooler	S72	S75	[-]	S75

3.3.6 Output of the layer

The outputs of the TEA layer are ten TEA KPIs and LCA foreground data, all listed in Table S5.

3.4 LCA layer

LCA aims to quantify a product's or process's environmental impacts. Generally, this quantification requires detailed data on all life cycle phases, which is usually unavailable in the early design phase of material discovery. To still incorporate environmental KPIs, we developed a predictive LCA model for carbon capture using metal-organic frameworks that predicts the environmental impacts of a carbon capture solution solely from the foreground data of the capture plant and the molecular structure of the used material.

3.4.1 Goal and scope definition

The goal of the Life Cycle Assessment (LCA) is to estimate the environmental impacts of the selected carbon capture process for all adsorbent materials to guide material selection based on LCA KPIs. Generally, the carbon capture plant considered within the PrISMa platform captures the CO_2 from flue gas to reduce the CO_2 emissions to the atmosphere. However, the environmental benefit of reducing the CO_2 in the atmosphere is decreased by direct and indirect emissions, e.g., from utility demand required for plant operation. Moreover, environmental impact categories beyond climate change are increased and must be analyzed to ensure overall environmentally benign solutions. To analyze the environmental impacts, we account for the energy and material exchanges of the capture plant with the environment during the life cycle of the capture plant. Because our PrISMa platform aims to compare the KPIs of using different materials for carbon capture serving a specified CO_2 sink, we predict the environmental impacts of the captured CO_2 from cradle-to-gate. We define the functional unit that defines the function of our investigated process as '1 kg CO_2 captured'. Consequently, our system boundaries include the carbon capture plant (i.e., the energy demand of utilities, compensation of parasitic load for power plant case studies, steel demand for plant construction, material synthesis, and material disposal (see Figure S5)). Within our system boundaries, we do not consider the environmental impacts of the CO_2 source and flue gas treatment and the CO_2 transportation, utilization, or storage, which are

expected to be the same for all materials and the Mono-Ethanol-Amine (MEA) benchmark process.

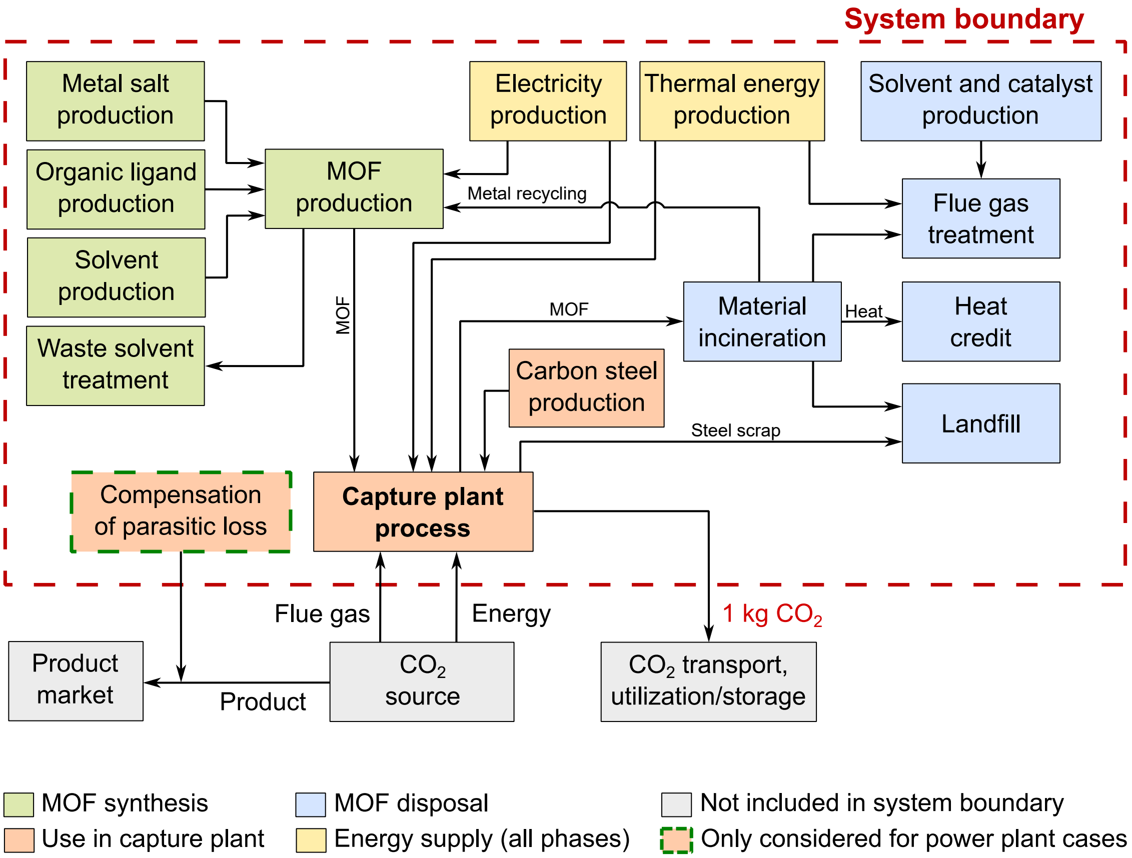

Figure S5| LCA system boundaries. Schematic illustration of the system boundaries considered within the LCA of the adsorption-based carbon capture process.

3.4.2 Life cycle inventory

To assess the environmental impacts of the entire life cycle of the carbon capture plant, we divide our model into three phases: 1) the use phase of the carbon capture plant, 2) the materials synthesis phase, and 3) the materials disposal phase.

Use phase in carbon capture plant. Our predictive LCA model for the use phase of the carbon capture plant calculates the environmental impact using foreground data provided by the TEA model (cf., Section 6.3.11). The model captures the environmental impacts from

the energy supply of the utility systems, i.e., for power, heating, and cooling, depending on the energy supply scenario of the specific utility system as defined in the TEA model. For power plant case studies, the energy demand of the carbon capture plant is primarily supplied by the power plant (e.g., by steam extraction) until the limits of the power plant are reached. Due to the operating limits of the power plant, it cannot compensate for the resulting parasitic load (loss of electricity due to the operation of the carbon capture plant). This parasitic load needs to be compensated by other electricity producers, and the resulting indirect emissions must be accounted for in LCA.[S82] Here, we assume a compensation of the parasitic load by the average grid characteristics in the defined region, following standard LCA practice.[S83]

The environmental impacts for carbon capture plant construction, disposal, and recycling at the end of life are considered based on rough estimations of the carbon steel demand of the equipment provided by the TEA model (cf., Section 6.3.11). At the end of life, we assume a recycling rate of the carbon steel of 85 % based on Reuter et al.[S84] and a transport distance to a landfill of 15 km by lorry based on Frischknecht et al.[S85].

Material synthesis phase. Due to the diversity of metal-organic framework synthesis, the detailed consideration of material synthesis in a high-throughput materials screening is challenging. Thus, we have developed a simplified, predictive LCA model that estimates the environmental impacts of material synthesis from the 3D molecular structure. The model focuses on a detailed assessment of the metal used within the material.

We initially fragment the MOF based on its molecular structure in the model using the so-called Moffragmentor tool.[S86] We obtain information on the molecular structure of the metal node and all organic linkers within the MOF structure. Because we obtain molecular fragments from the structure, we perform a similarity search based on the PubChem database[S87] to identify the chemicals with the most similar molecular structure as the molecular fragments of the organic linkers. The identified chemical is considered a reactant of the

organic linker. If the PubChem search fails, we approximate the molecular structure of the organic linker reactant by the molecular structure of the fragment. From the molecular structure of the identified chemicals and their occurrences within the MOF structure, the demand of the organic linkers for material synthesis is estimated from the carbon content of the material. If no carbon is contained in a linker, the organic linker demand is estimated from the nitrogen, phosphorus, or sulfur content in a hierarchical order. The metal demand is estimated from the metal content. We assume a fixed MOF yield rate of 85 % based on Sathre and Masanet[S88]. For simplicity, we assume that all organic linkers have the same environmental impact as terephthalic acid, the most occurring organic linker of the materials in our database. To assess the environmental impact of the metal precursor, we select a proper metal or metal compound market process from the LCIA database following a hierarchical procedure:

1. Select the market process of the metal oxide, if available.
2. Select the market process of a metal compound other than oxide as a proxy, if available (e.g., a carbamate).
3. Select the market process of the pure metal, if available.
4. If no metal or metal compound market process is available in the LCIA database, use the mean environmental impact per kg of metal of the market processes of all metal oxides of the corresponding elemental family (e.g., alkali metals or transition metals).

The environmental impacts of metal compounds are transferred into the functional unit '1 kg pure metal' based on the chemical formula.

The solvent in which a MOF is synthesized can have an important impact on several LCA-KPIs.[S89] In this work, we developed a methodology to predict the greenest solvent that can be selected for synthesizing a given MOF. This selection approach uses the (limited) information available at this initial screening stage.

We follow a hierarchical solvent selection procedure that pursues two aims: 1) the use of green solvents and 2) the use of solvents that are miscible with the organic linker. To select the solvent used for the synthesis, we follow a sequential approach where a set of five solvents that are frequently used in MOF synthesis (i.e., water, ethanol, methanol, DMF, and DMA[S90]) are considered and tested (from greenest to least green).

We assume that a MOF can be synthesized in a particular solvent if no phase split occurs between the solvent and the organic linkers, i.e., no liquid-liquid-equilibrium (LLE) exists. Our sequential approach starts by testing water as the solvent. If water forms an LLE with one of the organic linkers, we continue with the next, less green, solvent until we find a solvent that matches our criteria.

The LLE calculations are performed using the nonrandom two-liquid model (NRTL) model considering two synthesis conditions: 1) at 298.15 K, as room temperature requires less energy and is thus preferred and 2) 373.15 K to test if the phase split can be prevented for a green solvent with increased temperature. For the LLE calculations, we use the Framework for Equations of State and Classical Density Functional Theory (FeOs)[S91] combined with the SMILES-to-Properties-Transformer (SPT) model to predict NRTL parameters from the SMILES codes of the organic linkers and the solvents.[S92,S93] We exclude LLEs with a miscibility gap smaller than 0.05 (molar) or occurring at molar organic linker concentrations larger than $x_{\mathrm{Organiclinker}} = 0.5$. A solvent is then selected in the hierarchical order, starting with water if no LLE occurs for one of both tested temperatures.

The input of the SPT model is a combined string of the SMILES codes of both binary mixture components, i.e., the organic linker and the solvent. The used version of SPT allows a maximal length of the combined SMILES string of 128 characters. Moreover, solely characters considered in the training process of the SPT model can be used. Thus, for input strings longer than 128 characters or organic linkers SMILES codes containing characters not trained in SPT, we assume a proxy solvent with average environmental impacts of all

five tested solvents. The proxy solvent is also assumed if none of our five tested solvents matches our criteria.

For all solvents, we consider a mass ratio solvent/MOF of 70 and solvent recycling rate of 90 % based on Sathre and Masanet[S88]. The non-recycled solvent is discarded in a wastewater treatment process if water is selected or a spent solvent mixture treatment process with energy recovery if an organic solvent is selected.

In the current model, we use proxy data from the literature for the power demand of the synthesis of 4.178 $kW\,h\,kg^{-1}$ based on Hu et al.[S89]. The grid supplies the power demand with average characteristics in the defined region. Finally, we consider the transport distances of the material to the capture plant of 600 km by train and 100 km by lorry.[S85] The assumptions of the simplified, predictive model introduce uncertainties into the model. Thus, a detailed LCA based on laboratory synthesis data has to be performed a posteriori for the most promising candidates.

Materials disposal phase. For the material disposal, we assume incineration of the material and subsequent metal recycling and landfill. The input of the material disposal model is the materials' element weight fraction. For metal-organic frameworks, we calculate the element weight fraction from the molecular structure or, for the experimental structures, from the chemical formula defined in the corresponding literature. The material is first transported to the incineration plant considering a transport distance of 10 km by lorry. The emissions of the incineration to the air are calculated from the formed CO_2, fuel- and thermal NOx, N_2O, NH_3, and CN emissions, as well as particulates based on the waste incineration model of Doka[S94],[S95] with updated data from Meys et al.[S96]. The fuel-NOx emissions are calculated from the nitrogen content of the material. Doka[S94] assumes an equal share of fuel-NOx and thermal-NOx emissions for average wastes in municipal solid waste incinerators. To account for proper thermal-NOx emissions for materials without or with low nitrogen content, we assume fixed thermal-NOx emissions of average wastes[S94]

for materials with fuel-NOx emissions lower than the thermal-NOx emissions of the average waste. An equal share of fuel-NOx and thermal-NOx emissions is assumed for materials with fuel-NOx emissions higher than the thermal-NOx emissions of the average waste. For flue gas treatment, Selective Catalytic Reduction (SCR) is assumed as an industrial standard in waste incineration. For the SCR, we consider NH_3, heat (natural gas boiler), and catalyst (TiO_2 and V_2O_5) demands according to Doka[S94],[S95].

If sulfur, phosphorus, or halogens (Br, Cl, F, or I) are contained in the MOF, we assume that acids are formed and neutralized using calcium carbonate in a stoichiometric reaction. The neutralization leads to additional CO_2 emissions to air and calcium carbonate demand. The formed salts from sulfur or phosphorus ($Ca_3(PO_4)_2$, $CaSO_4$) are transported to a landfill. For halogens, the formed ions (Br^-, Cl^-, F^-, I^-, and Ca^{2+}) are considered as emissions to water.

The produced heat of the incineration is calculated depending on the amount of carbon and hydrogen remaining according to the mass balance of all reactions. For the heat of incineration, credit to the environmental impacts is given for replacing state-of-the-art heat-production technologies, i.e., here, the market for the district or industrial heat based on natural gas. A recycling rate of 95 % is assumed for the metals,[S89] and credits to the environmental impacts are given for the recycled material based on the hierarchical approach for selecting the process for metals from the LCIA database in the materials synthesis model. All material amounts neither recycled nor emitted to air or water are transported to a landfill considering a transport distance of 15 km by lorry.[S85]

3.4.3 Life cycle impact assessment

We use the LCIA method Environmental Footprint 3.0, recommended by the European Commission's Joint Research Centre.[S97,S98] The LCIA method comprises 28 impact categories (16 main categories and 12 subcategories) that can be used as KPIs for material selection. To account for the environmental impact of the background system, we use the LCA database

Ecoinvent V3.8 with the Ecoinvent system model at the Point of Substitution (APOS).[S99] We choose the ecoinvent system model APOS for a consistent consideration of material recycling in a future application.[S100] The Ecoinvent database provides total environmental impacts that capture both the direct emissions of a process and the indirect emissions of the background supply chains.

3.4.4 Validation of the layer

The lack of LCA data makes the validation of the outputs of the layer challenging. However, each individual part of the LCA model is validated or taken from the literature:

- **Use Phase:** The accuracy of the model used for the use phase depends strongly on the quality of the foreground data obtained from the TEA layer. Thus, the expected accuracy is in a similar order of magnitude as the TEA layer.
- **Disposal Phase:** For the disposal phase, we use an established approach from the literature to assess the environmental impacts of waste incineration from the elementary composition.
- **Synthesis Phase:** For the synthesis of MOFs, the data basis for LCA is most challenging. Thus, we focused in the synthesis model on the key learnings from LCA studies from the literature that identified two hotspots: the impact of the metal and solvent. Moreover, we use validated proxy data from the literature for the synthesis parameters.[S89]

We use detailed and accurate LCIA scores from a commercial LCA database for the background data.[S99] By combining the individual, validated parts, the LCA model can capture trends between the materials and guide the selection of sustainable adsorption materials for carbon capture.

3.4.5 Output of the layer

The outputs of the LCA layer are 28 impact categories of the Environmental Footprint 3.0 per 1 kg CO_2 captured.

4 MEA Benchmark

4.1 TEA assumptions for the MEA benchmark

In the TEA layer, the assumptions for the MEA benchmark for the power plant case studies are based on the work of the EBTF.[S1,S2] The following modifications have been introduced to align the methods of Manzolini et al.[S2] to the methodology of the PrISMa platform (cf., Section 3.3):

1. All costs have been updated from 2008 to 2019 using the Harmonised Index of Consumer Prices (HICP) average historical value for the Euro Area (EA) zone.

2. The process, project contingencies, and owner's costs for the current platform methodology have been adopted to align the methods to estimate the total capital required.

3. The natural gas and coal costs are changed to reflect the UK-specific average cost for 2019.

4. The calculation of the levelized cost of electricity for the power plant with MEA based capture is changed to follow the approach of the TEA layer in the PrISMa platform, as described in the Section 3.3.

For the industrial case studies, in this particular case for cement plants, the assumptions for the MEA benchmark are based on the work conducted in the CEMCAP[S3,S4] project. Performance parameters (i.e., specific CO_2 emissions of the reference plant, electricity, and thermal energy consumption in the MEA-based capture plant) and equipment cost for the CO_2 capture plant, compression train, and steam generation plant are taken from the report. Waste heat is recovered from the cooling air exiting the hot clinker grate cooler. However, additional thermal energy is required for the solvent regeneration in the form of a natural gas boiler. Additional electricity is taken from the grid for the solvent pumps, water pumps, and CO_2 compression train. The CO_2 emission intensity of the electricity grid and natural

gas boilers are taken from the same LCA database as for the sorbent-based carbon capture process (cf., Section 3.4).

The following modifications have been introduced to align the methods to the methodology followed in the PrISMa platform (cf., Section 3.3):

1. All costs have been updated from the year 2014 to 2019 using the Harmonised Index of Consumer Prices (HICP) average historical value for the Euro Area (EA) zone.

2. The process, project contingencies, and owner's costs for the current platform methodology have been adopted to align the different methods to estimate the total capital required.

3. The natural gas and electricity costs are changed to reflect the UK-specific average cost for 2019.

4.2 LCA assumptions for the MEA benchmark

Goal and scope definition. As for sorbent-based carbon capture technologies, we calculate the environmental impacts of the captured CO_2 for the MEA benchmark cradle-to-gate and define the functional unit as '1 kg CO_2 captured'. Our system boundaries of the MEA benchmark include the MEA-based carbon capture plant and the corresponding synthesis of the material demands, disposal of degradation products, and compensation of parasitic load for the power plant case studies (see Figure S6). For the NGCC and coal power plant case studies, no additional energy demand supplied by utilities is required for the MEA benchmark capture process because the power plants can supply the total required energy.

Life cycle inventory. The TEA model of the MEA benchmark provides the LCA foreground data, i.e., the mass of CO_2 captured, the MEA demand, and the parasitic load or heating and power demand for power plant and cement case studies, respectively. As for the sorbent-based capture plant, for power plant case studies, the parasitic load is compen-

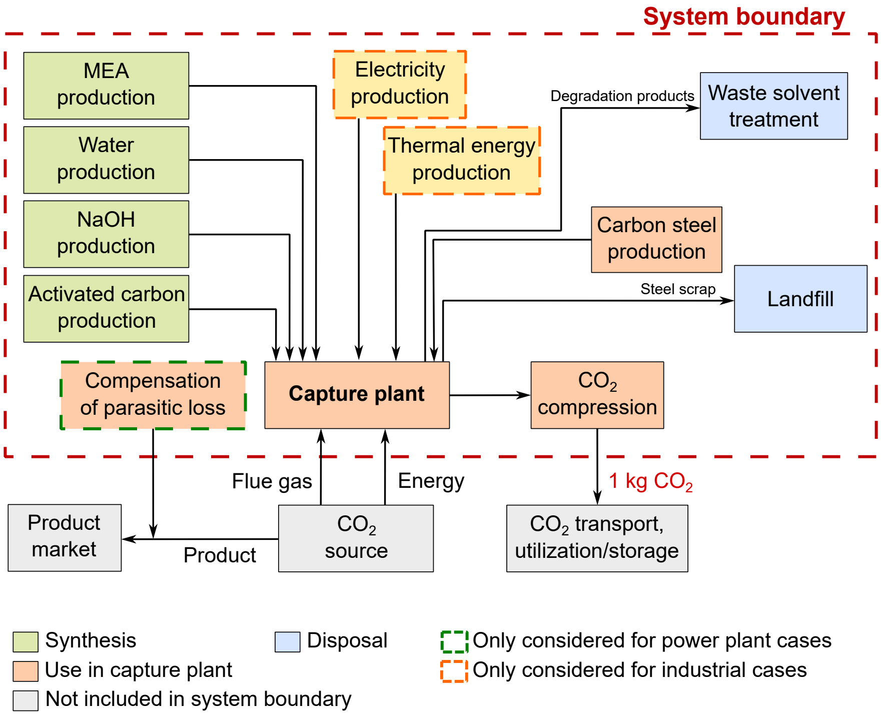

Figure S6| General LCA system boundaries for MEA benchmark processes. Schematic illustration of the system boundaries considered within the LCA of the MEA benchmark processes.

sated by the average grid characteristics in the defined region.[S83] We calculate the material demand for MEA, water, sodium hydroxide (NaOH), activated carbon, and steel for plant construction if available using LCI data from Singh et al.[S101] for the NGCC power plant and from Koornneef et al.[S102] for the coal power plant. Moreover, we assume the mass of degradation products and the emissions to air (i.e., MEA, ammonia, formaldehyde, and acetaldehyde) proposed by Singh et al.[S101] and Koornneef et al.[S102]. No detailed LCI data is available for the cement case in the literature. Therefore, we approximate the missing LCI data by the data from the coal power plant case[S102], (i.e., the demand for water, sodium hydroxide, activated carbon, and steel and the emissions to air). Since this data is defined per amount of captured CO_2 and the environmental impact is generally dominated by energy-related emissions calculated from the LCI data taken from the TEA model, the resulting error is expected to be small. The degradation products are discarded in a hazardous waste incineration plant for spent solvents with energy recovery.

Life cycle impact assessment. We consider the same database and assumptions for the LCIA as for the sorbent-based carbon capture process (cf., Section 3.4).

The same LCA outputs obtained from the MOF screening are obtained for the MEA benchmark (i.e., 28 impact categories of the Environmental Footprint 3.0 per 1 kg CO_2 captured).

4.3 Additional remark

Generally, the assumptions have been adopted from MEA-based capture studies available for NGCC power plants. However, it should be noted that these assumptions have not been validated experimentally. Based on the recent publication by Moser et al.[S103], previous technical and environmental evaluations of MEA-based capture processes have underestimated both degradation and emissions. The work of Moser et al.[S103] has not been used in the present study due to differences in flue gas characteristics. Their experimental studies based

on flue gas from a lignite-based power plant, which contains much less oxygen than that of NGCC flue gas, already suggest higher degradation rates, mainly due to oxidative degradation. This uncertainty is covered by applying sensitivity factors of ±50 % to the CAPEX and OPEX estimates of the MEA benchmark. Overall, the expected underestimation of the amine degradation and emissions leads to underestimating the environmental impacts of the MEA benchmark. Thus, the comparison to the MEA benchmark using LCA KPIs is a conservative assessment of the actual potential of adsorption-based carbon capture processes.

5 Materials layer: Comparison with experimental data

The basic thermodynamic data for a given material for the PrISMa platform comes from experimental data or molecular simulations. For the molecular simulations, we use the UFF force field (see 3.1.2). To obtain some insights into the accuracy of these predictions, we compare the predicted pure component isotherms with experimental isotherms previously reported in the literature for a representative set of 18 structures. For 7 out of those 18 structures, we provided additional experimental data using the procedures described in Section 5.1 (see Figure S7). We also present a comparison of the simulated isotherms with the experimental ones for zeolite 13X, as we compare the performance of zeolite 13X with some MOFs in Section 9.3.2.

5.1 Gas sorption experiments

The CO_2 (and N_2) isotherms measurements at 298 K (or 303 K) for the samples (Ca-squarate, MIP-212(Al/Cu), CAU-10(Al), MIL-160(Al), MIL-53(Al)-NH2, MIL-96(Al), and MIL-91(Ti)) were performed on a Micromeritics Triflex instrument with a temperature-controlled bath. In all the cases, the measurements were recorded using ultra-high purity gas ($\geq$ 4.8 grade). Before the adsorption measurement, each sample was degassed under heating for at least 8 hours. The degassing was done in one step using a Micromeritics SmartVacPrep degas unit ($P = 10 \times 10^{-6}$ mbar; at which point the outgassing rate was $\leq 2\,\mu\text{bar min}^{-1}$).

5.2 Al-containing MOFs

Boyd et al.[S33] noticed that for Al-containing MOFs the UFF force field overestimates the adsorption of CO_2. As Al is an abundant metal, Al-MOFs are potentially attractive for large-scale carbon capture applications. Therefore, we compared the predictions of the force field used by Boyd et al.[S33] with the regular UFF parameters for Al.

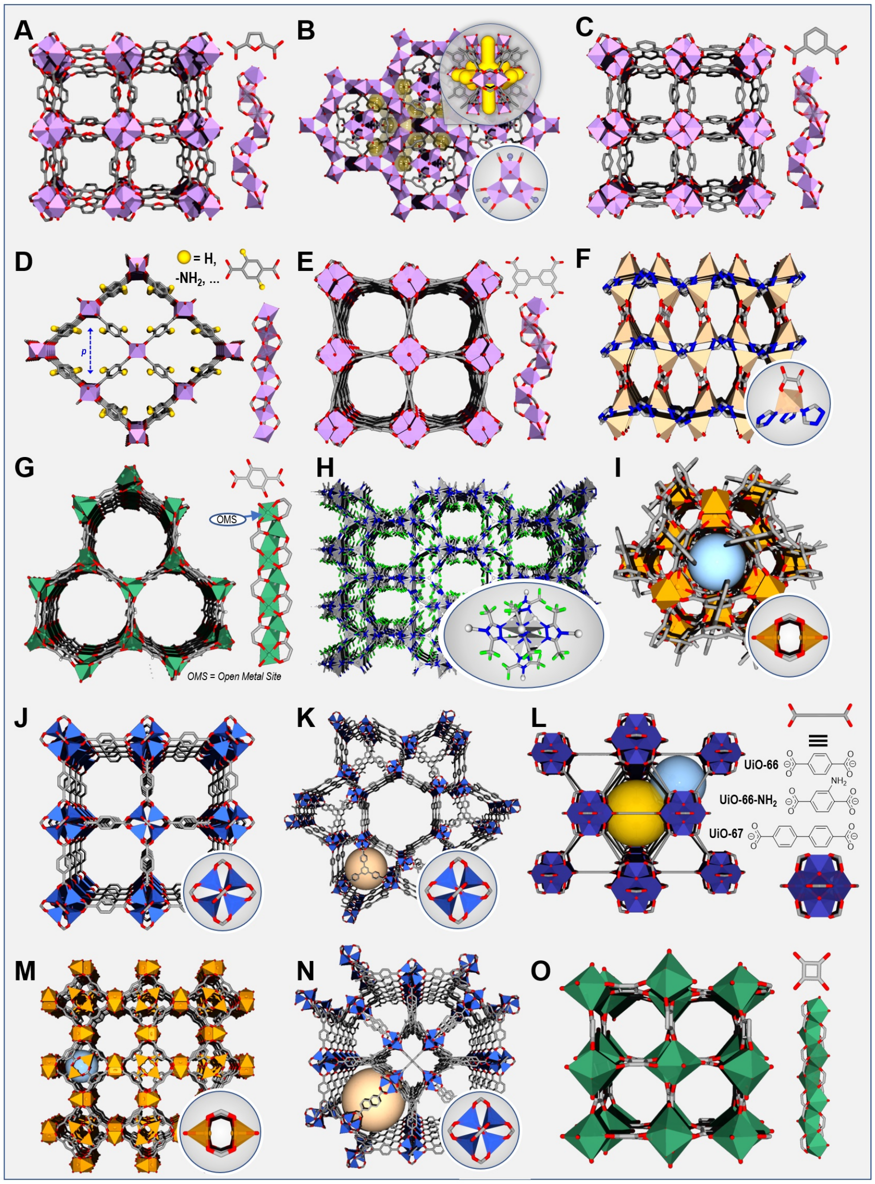

Figure S7| MOFs of which the experimental isotherms are compared with the predictions from molecular simulations Crystal structures of MOFs studied in section 5. (A) MIL-160(Al), (B) MIL-96(Al), (C) CAU-10(Al), (D) MIL-53(Al)'s, (E) NOTT-300(Al), (F) CALF-20(Zn), (G) MOF-74(Mn), (H) FMOF-1(Ag), (I) PCN-14(Cu), (J) MOF-5(Zn), (K) MOF-205(Zn), (L) UiO-66(Zr), UiO-66(Zr)-NH2 and UiO-67(Zr), (M) HKUST-1(Cu), (N) UMCM-1(Zn) and (O) Ca-Squarate. Color code: AlO_6, pink polyhedron; ZnO_2N_3, light yellow polyhedron; MnO_5, green polyhedron; AgN_4, grey polyhedron; CuO_5, orange polyhedron; ZnO_4, bleu polyhedron; ZrO_8, dark blue polyhedron; CaO_7, dark green polyhedron; O, red; C, grey; N, blue and F, green. The crystal structure of MIP-212 is presented in Section 12.1.

In these figures, unless indicated, the isotherms are at 298 K. Colored closed circles are experimental isotherms. Open black circles are the predictions from our simulations, where a solid line is added for the scaled force field.

5.2.1 MIL-160

MIL-160 was first described by Cadiau et al.[S104]. The chemical formula of the dry MOF is $Al_8H_{24}C_{48}O_{48}$ or $Al(OH)(O_2C{-}C_4H_2O{-}CO_2)$. In this work, the sample was prepared using the synthesis optimized by Permyakova et al.[S105]. The metal nodes are AlO_6 octahedra, arranged in a chain-like building unit. The carboxylate groups of the linker link these units together.

In Figure S8, we compare the predicted CO_2, N_2, and H_2O isotherms with the experimental data of Cadiau et al.[S104], Permyakova et al.[S105], Shade et al.[S106], Damasceno Borges et al.[S107], Silva et al.[S108], Wang et al.[S109], Wahiduzzaman et al.[S110], and this work.

For CO_2 and N_2, we see a significant improvement if we use the scaled force field instead of the regular UFF force field. Our predictions are in excellent agreement with one set of experimental data. Also, for H_2O, our model agrees with the experimental data.

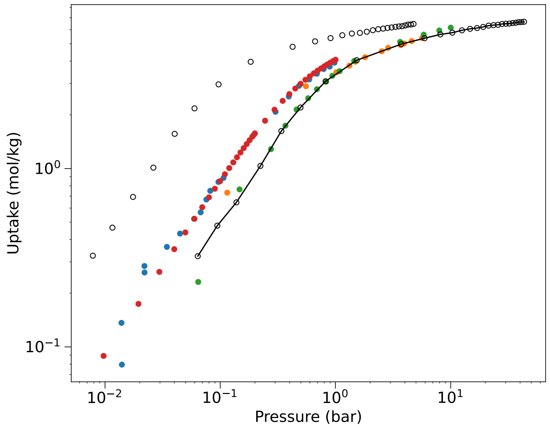

(a) - CO_2

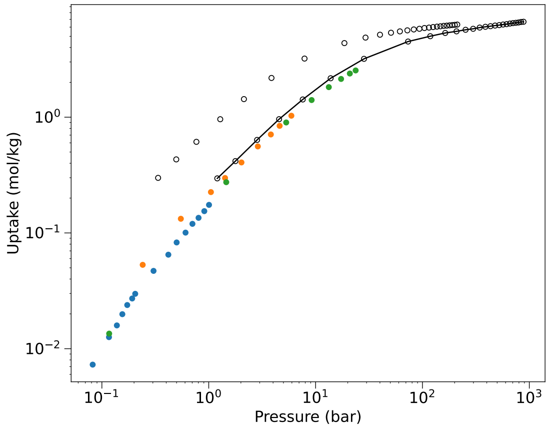

(b) - N_2

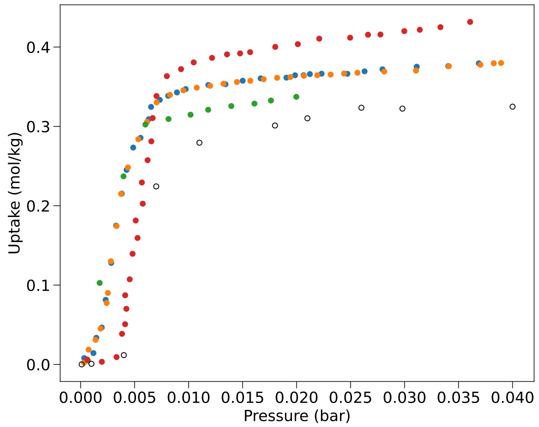

(c) - H_2O

Figure S8| MIL-160 | Comparison of the experimental isotherms (blue,[S106] orange,[S108] green,[S107] and red; this work) with the computational isotherms (open symbols, with solid line, is the scaled model) for (a) CO_2, (b) N_2, and (c) H_2O. For the water experimental isotherms: blue,[S104] orange,[S109] green,[S105] and red[S110]

5.2.2 MIL-96

MIL-96 was first synthesized by Loiseau et al.[S111]. The chemical formula is $Al_{12}O_{87}C_{54}H_{102}$, or $Al_{12}O(OH)_{16}(H_2O)_5[BTC]_6 \cdot nH_2O$ (n = 29). The structure of MIL-96 (revisited by[S112]) consists of 18-member hexagonal honeycomb layers from the infinite chains of $AlO_4(OH)_2$ and $AlO_2(OH)_3(H_2O)/AlO_2(OH)_4$ octahedra together with μ_3-oxo-centered isolated Al-trimers (all Al(III) adopt octahedral coordination), all linked together through 1,3,5-benzenetricarboxylate (BTC) ligands. In Figure S9, we compare the computational isotherms with the experimental ones of Liu et al.[S113] and this work for CO_2.

For this MOF, the difference between the experimental data of the different groups makes it impossible to conclude which force field gives better predictions.

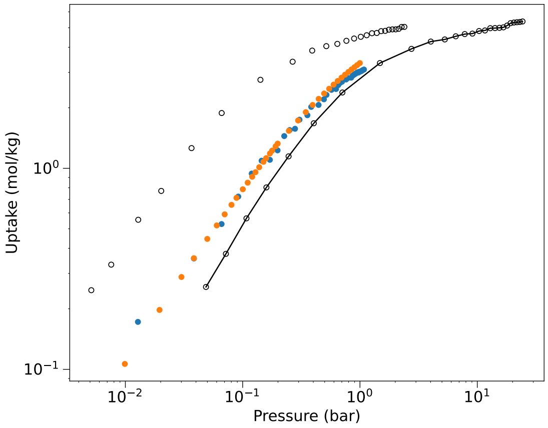

Figure S9| MIL-96 | Comparison of the experimental isotherms (blue[S113] and orange: this work) with the computational isotherms (open symbols, with solid line is the scaled model) for CO_2.

5.2.3 CAU-10

The CAU-10-X family of MOFs was first synthesized by,[S114] where X can be H, NO_2, OH or CH_3. Here, we study CAU10-H. The chemical formula is $Al_8H_{40}C_{64}O_{40}$ or $[Al(OH)-(O_2C-C_6H_4OH-CO_2)]\cdot 0.5(H_2O)$. The structure is built up of helical chains of *cis*-connected AlO_6-octahedra linked together through 1,3-isophthalate linkers.

In Figure S10, we compare the predicted CO_2 and N_2 isotherms with the experimental data of Abascal and Vega[S43], Pei et al.[S115], and this work.

Also, for this material, we see that the scaled UFF force field gives significantly better predictions of the experimental isotherms for both CO_2 and N_2.

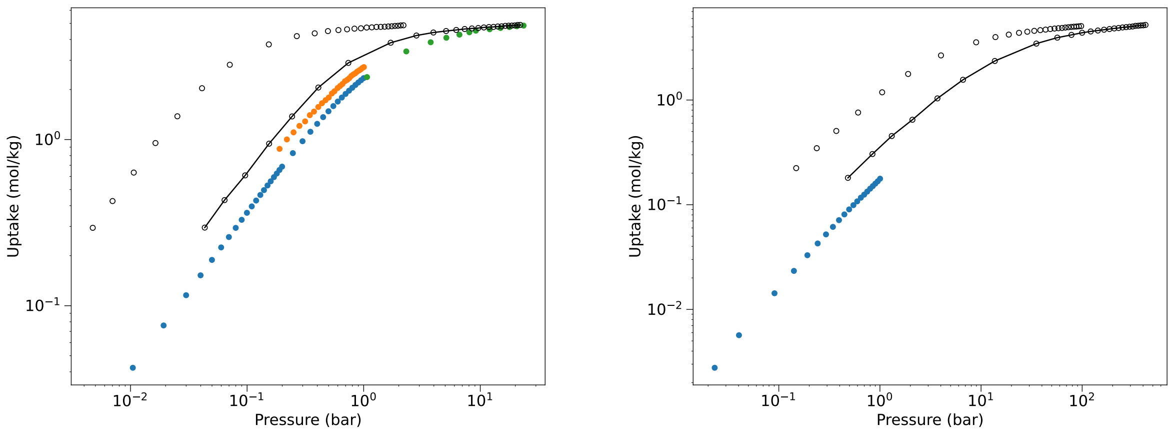

(a) - CO_2 **(b)** - N_2

Figure S10| CAU10 | Comparison of the experimental isotherms (orange,[S115] green,[S116] and blue; this work) with the computational isotherms (open symbols, with solid line is the scaled model) for (a) CO_2 and (b) N_2.

5.2.4 MIL-53-NH2

MIL-53-NH2 is part of the MIL-53 family of MOFs, which was first synthesized from Cr(III) ions and terephthalate ligands (BDC) by Millange et al.[S117] and later from Fe(III) or Al(III) as well as with various substituted BDC (X-BDC; X = NH_2, NO_2, COOH, halides, etc.) analogues.[S118] The MIL-53s are built of straight chains of trans-connected MO6-octahedra (M = Al(III), Fe(III), Cr(III), ...) linked with X-BDC ligands. This family of MOFs has been studied extensively because of their breathing behavior caused by the flexibility in the structure.[S119] One of the interesting analogs for the CO_2 capture/separation is the one obtained with the 2-amino-terephthalate (NH_2-BDC).[S120] The chemical formula of MIL-53-NH2 is $Al_8H_{48}C_{64}N_8O_{40}$ or $Al(OH)(NH_2-BDC)\cdot 3.5H_2O$.

In Figure S11, we compare the predicted CO_2 and N_2 isotherms with the experimental data of Serra-Crespo et al.[S121], Martinez Joaristi et al.[S122], Couck et al.[S123], Garcia-Perez et al.[S124], and this work. The experimental isotherms for H_2O have been obtained by Jeremias et al.[S125], Teo et al.[S126], Gaab et al.[S127], Bozbiyik et al.[S128], Wöllner et al.[S129], Coelho et al.[S130].

This is an example of a material that undergoes a structural transition upon gas loading rates, i.e., we increase loadings of CO_2 or N_2. This transition occurs at the steps in the isotherms. Our model assumes that we have a rigid framework, so we can't observe these types of transitions. However, these types of transitions can be detected using the approach of Sarkisov et al.[S46]. For H_2O, our model agrees well with the experiments.

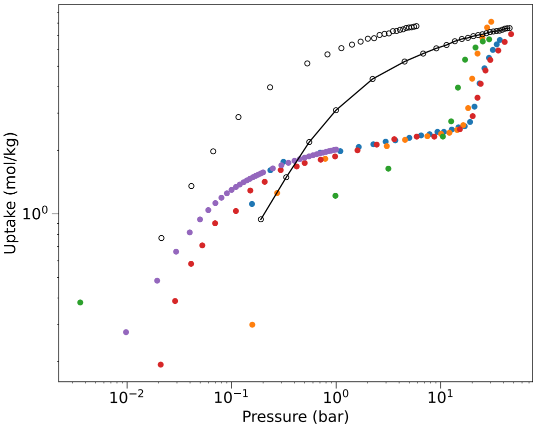

(a) - CO_2

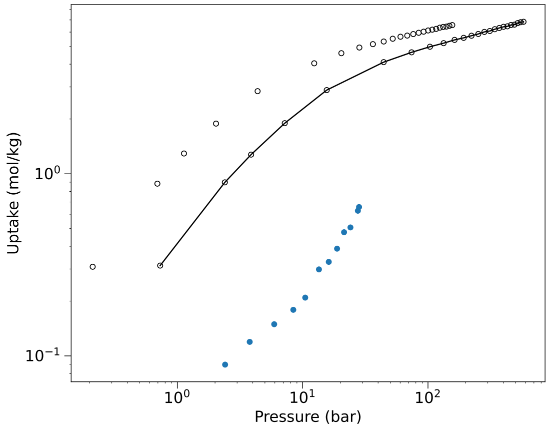

(b) - N_2

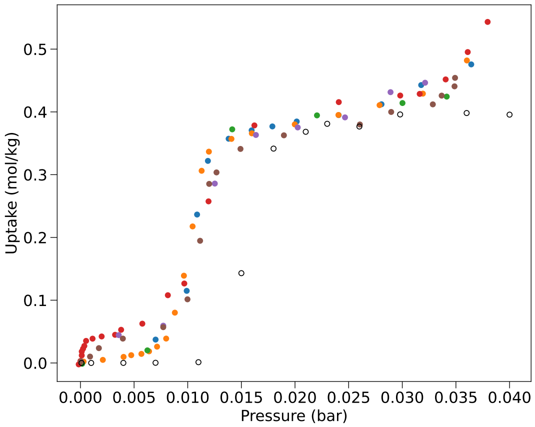

(c) - H_2O

Figure S11| MIL-53-NH2 | Comparison of the experimental isotherms (blue,[S121] orange,[S122] green,[S123] red,[S124] and purple; this work) with the computational isotherms (open symbols, with a solid line, is the scaled model) for (a) CO_2, (b) N_2 and (c) H_2O. For the water experimental isotherms: blue,[S125] orange,[S126] green,[S127] red,[S128] purple,[S129] and brown.[S130]

5.2.5 MIP-212

MIP-212 is a novel in-house MOF. Its structure is described in Section 12.1. The chemical formula is $Al_{16}Cu_{16}H_{112}C_{128}N_{64}O_{96}$ or $[Al(\mu_2-OH)Cu(\mu_2-H_2O)(PyC)_2]\cdot n-$solvent, where PyC stands for the pyrazolate 4-carboxylate ligand. The inorganic building units consist of Al-chains with $AlO_5(OH)$ octahedra and Cu-chains built of $CuN_4(OH_2)_2$ octahedra (when fully hydrated). As this material was synthesized in the context of this work, we present more details in Section 12.1.

In Figure S12, we compare the predicted CO_2, N_2, and H_2O isotherms with our experimental data. The scaled force field also better predicts the CO_2 and N_2 isotherms of this material. The predicted H_2O isotherm shows larger deviations. Interestingly, similar kinks are seen in the experimental isotherm.

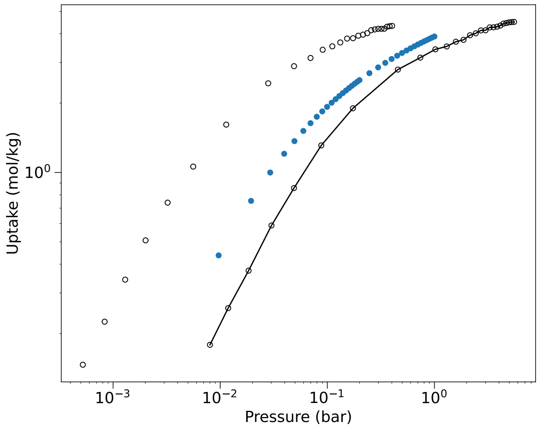

(a) - CO_2

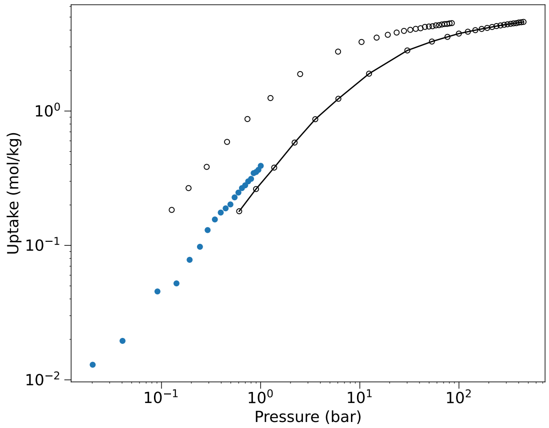

(b) - N_2

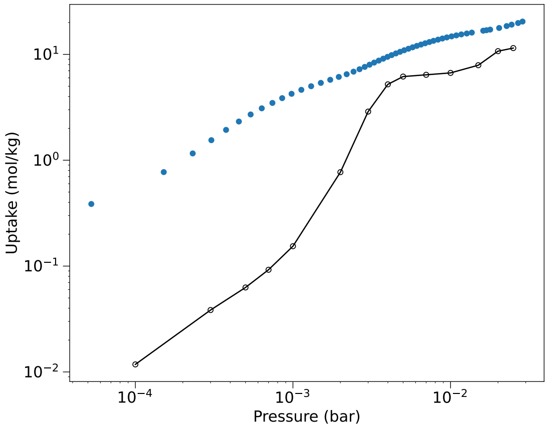

(c) - H_2O

Figure S12| MIP-212 | Comparison of the experimental isotherm (blue; this work) with the computational isotherms (open symbols, with a solid line, is the scaled model) for CO_2, N_2, and H_2O.

5.2.6 NOTT-300

NOTT-300 was first synthesized by Yang et al.[S131]. The chemical formula of the dry MOF is $Al_8H_{32}C_{64}O_{40}$, while the structure consists of 1D helical chains of corner-shared AlO_6-octahedra, linked together by tetracarboxylate linkers to form square-shaped channels.

In Figure S13, we compare the predicted CO_2 and N_2 isotherms with the experimental data of Shade et al.[S106] and Yang et al.[S131]. The scaled force field also better predicts the CO_2 and N_2 isotherms of this material.

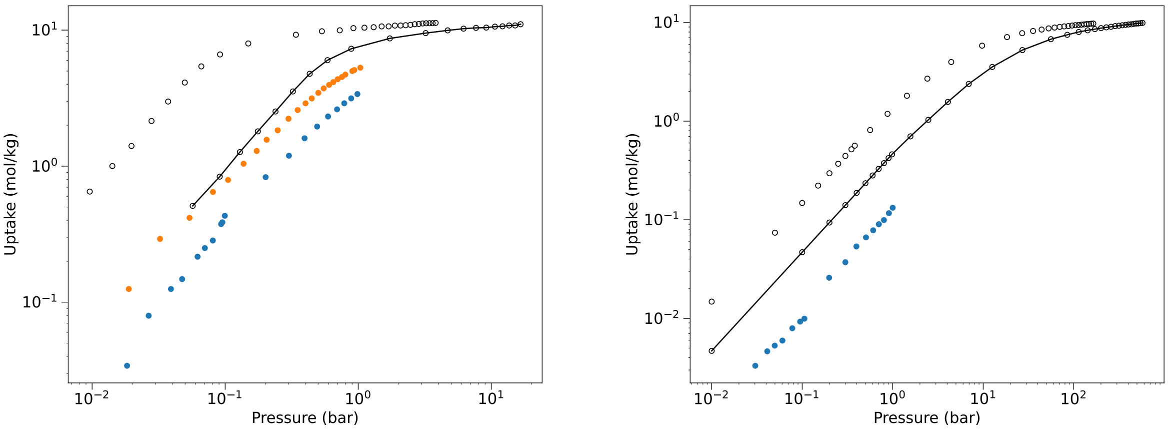

(a) - CO_2 **(b)** - N_2

Figure S13| NOTT-300 | Comparison of the experimental isotherms (blue[S106] and orange[S131]) with the computational isotherms (open symbols, with a solid line, is the scaled model) for (a) CO_2 and (b) N_2.

5.3 Other MOFs

5.3.1 CALF-20

CALF-20 was first synthesized by Lin et al.[S59]. The chemical formula is $Zn_4H_8C_{12}N_{12}O_8$ or Zn_2(1,2,4−triazolate)$_2$(oxalate). The isolated Zn(II) metal nodes are five-coordinated (ZnN_3O_2)in a (distorted) trigonal bipyramidal geometry where the structure can be described as "layers" of Zn-triazolates linked together via oxalate ligands.

In Figure S14, we compare the predicted CO_2 and N_2 isotherms with the experimental data of Lin et al.[S59] and Nguyen et al.[S132]. The predicted water isotherm is compared with data from Lin et al.[S59].

The predicted isotherms for CO_2 and N_2 are in excellent agreement with the experimental data. The agreement for H_2O is less good; we underestimate the amount of water adsorbed.

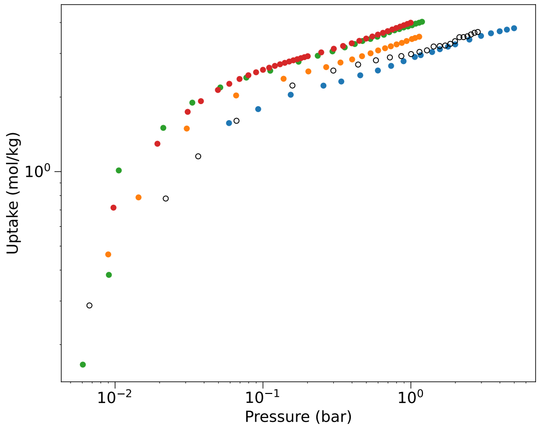

(a) - CO_2

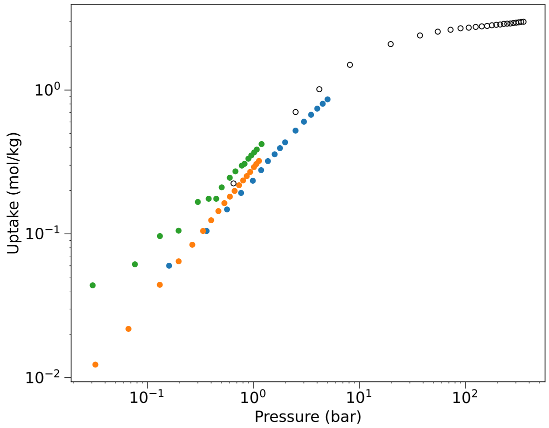

(b) - N_2

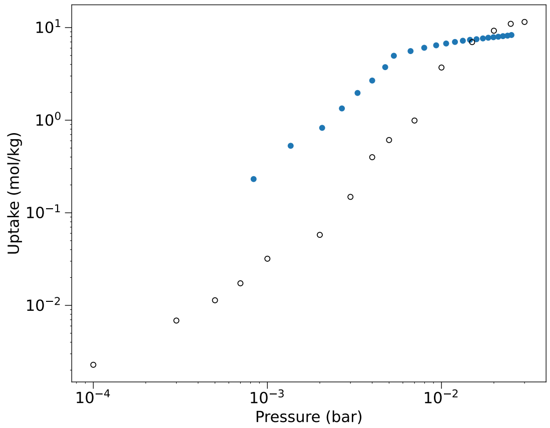

(c) - H_2O

Figure S14| CALF-20 | Comparison of the experimental isotherms (blue,[S132] orange,[S133] green[S59]) and red; this work, with the computational isotherms (open symbols) for (a) CO_2, (b) N_2, and (c) H_2O. The experimental water isotherm (blue points) was obtained from Lin et al.[S59].

5.3.2 MOF-74(Mn)

The MOF-74 (also known as CPO-27) structure was first synthesized with Zn(II) as the metal node.[S134] Subsequently, the MOF-74 structure has been synthesized with different metal ions by different groups (e.g., Ni, Co, Zn, Mg, and Mn by Dietzel et al.[S135], Co, Ni, Zn, and by Mg Caskey et al.[S136], and Fe by Bloch et al.[S137]). The chemical formula is $Mn_6H_6C_{24}O_{18}$, or $Mn_2(DHBDC) \cdot (H_2O)_2$ with Mn as metal node and 2,5-dihydroxy-1,4-benzenedicarboxyl (DHBDC) as linker.

In Figure S15, we compare the computational isotherms with the experimental ones of Yu et al.[S138] and Queen et al.[S139] for CO_2 and N_2.

As MOF-74(Mn) has an open metal site, one can expect the UFF force field to underestimate the adsorption isotherms, as shown by Dzubak et al.[S140] for Mg-MOF74. However, the scatter in the experimental data for the Mn version of the materials does not allow us to draw firm conclusions for this material.

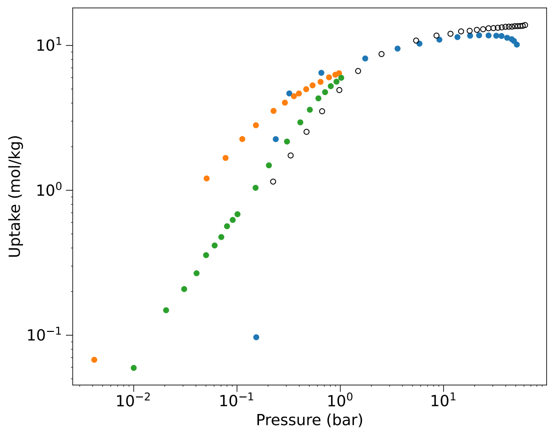

(a) - CO_2

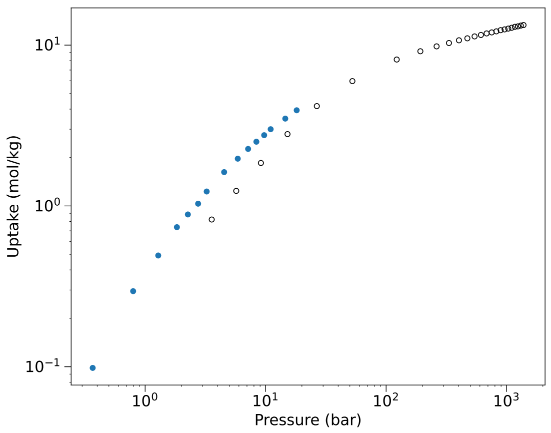

(b) - N_2

Figure S15| MOF-74(Mn) | Comparison of the experimental isotherms (blue,[S138] orange,[S138] and green[S139]) with the computational isotherms (open symbols) for (a) CO_2 and (b) N_2.

5.3.3 FMOF-1

FMOF-1 was first synthesized by Yang et al.[S141]. The chemical formula is $Ag_{24}C_{96}N_{72}F_{144}$ or $Ag_2[Ag_4Tz_6]$. FMOF-1 is a fluorous MOF and is formed by the reaction of a perfluorinated ligand (3,5-bis(trifluoromethyl)-1,2,4-triazolate(Tz)) with Ag(I) as a metal node.

In Figure S16, we compare the computational isotherms with the experimental ones of Moghadam et al.[S142] for CO_2.

We obtain excellent agreement at low pressure. At high pressure, we underestimate the loading, which is due to the flexibility of the structure.[S142]

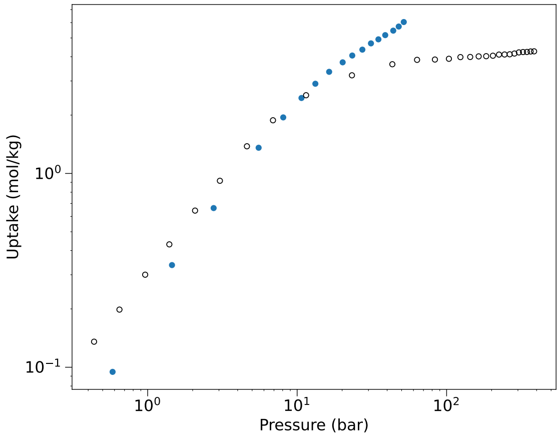

Figure S16| FMOF-1 | Comparison of the experimental isotherm (blue[S142]) with the computational isotherm for (a) CO_2.

5.3.4 PCN-14

PCN-14 was first synthesized by Ma et al.[S143]. The chemical formula is $Cu_{12}H_{84}C_{180}O_{48}$ or $Cu_2(H_2O)_2$(adip) with adip = 5,5-(9,10-anthracenediyl)di-isophthalate. The metal nodes are Cu, and the linker is derived from an anthracene derivative.

In Figure S17, we compare the computational isotherms with the experimental ones of Perry IV et al.[S144] and Parkes et al.[S145] for N_2.

The predicted N_2 isotherm is in excellent agreement with the experimental data.

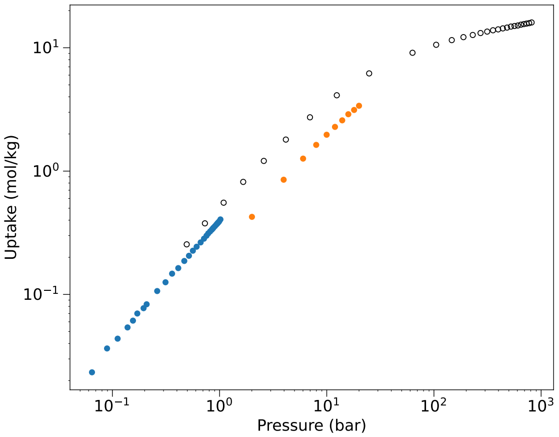

Figure S17| PCN-14 | Comparison of the experimental isotherms (blue[S144] and orange[S145]) with the computational isotherm for N_2.

5.3.5 UiO-66

UiO-66 was first synthesized by Cavka et al.[S146]. The chemical formula is $Zr_6H_{28}C_{48}O_{32}$, with a Zr_6-cluster as metal node and as linker benzene-1,4-dicarboxylate (BDC).

In Figure S18, we compare the computational isotherms with the experimental ones,[S147–S154] and Li et al.[S155] for CO_2 and N_2.

Our model slightly overestimates the adsorption in the Henry regime, yet the overall agreement for both CO_2 and N_2 is good.

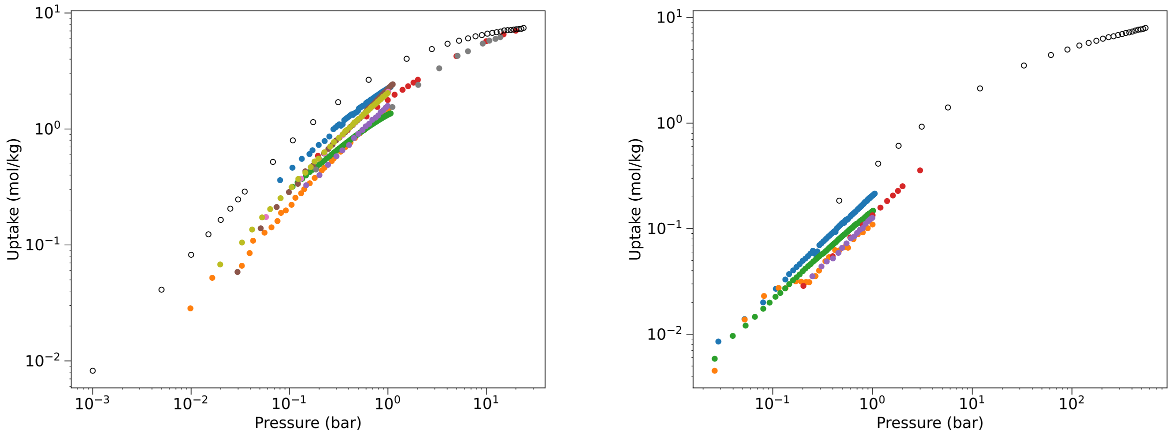

(a) - CO_2 **(b)** - N_2

Figure S18| UiO-66 | Comparison of the experimental isotherms (blue,[S147] orange,[S148] green,[S149] red,[S150] purple,[S151] brown,[S152] pink,[S153] grey,[S154] and yellow[S155]) with the computational isotherms (open symbols) for (a) CO_2 and (b) N_2.

5.3.6 MOF-5

MOF-5 (or IRMOF-1) was first synthesized by Li et al.[S156]. The chemical formula of MOF-5 is $Zn_8H_{24}C_{48}O_{26}$, or $Zn_4O(BDC)_3$, where Zn_4O is the metal node and the linker is 1,4-benzenedi-carboxylate (BDC).

In Figure S19, we compare the experimental isotherms,[S157–S160,S160,S161] and Millward and Yaghi[S162] with the computational ones for CO_2 and N_2.

The predicted isotherms for CO_2 and N_2 are in excellent agreement with the experimental data.

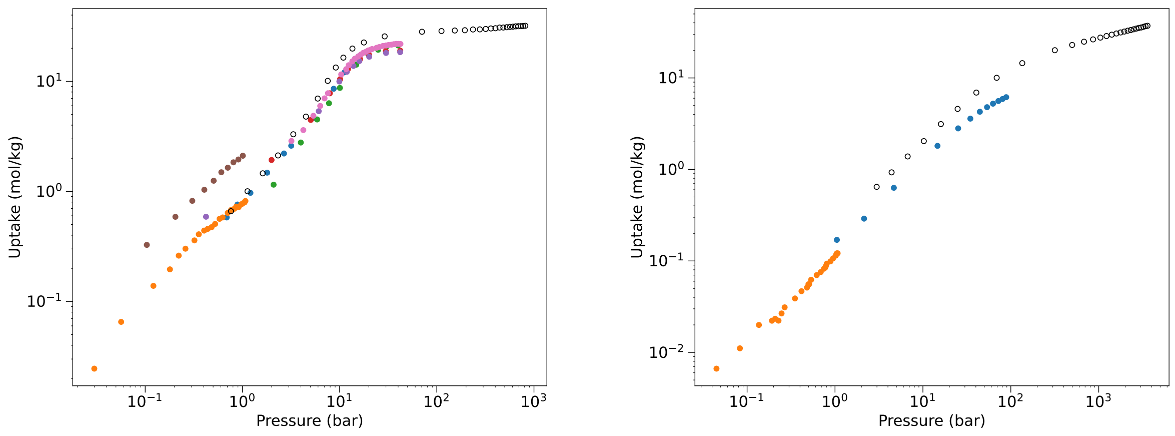

(a) - CO_2 **(b)** - N_2

Figure S19| MOF-5 | Comparison of the experimental isotherms (blue,[S157] orange,[S158] green,[S159] red,[S160] purple,[S160] brown,[S161] and pink[S162]) with the computational isotherms (open symbols) for (a) CO_2 and (b) N_2.

5.3.7 MOF-205

MOF-205 was first synthesized by Furukawa et al.[S163]. The chemical formula of MOF-205 is $Zn_{24}H_{156}C_{288}O_{78}$. MOF-205 has as metal node Zn_4O and two different linkers: 4,4,4-benzene- 1,3,5-triyl-tribenzoate (BTB) and 2,6-naphthalenedicarboxylate (NDC).

In Figure S20, we compare the experimental isotherms by Sim et al.[S164],[S164], and Furukawa et al.[S163] with the computational ones for CO_2.

The predicted N_2 isotherm is in excellent agreement with the experimental data.

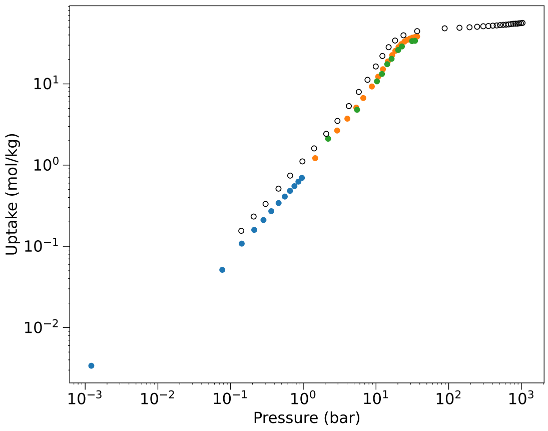

Figure S20| MOF-205 | Comparison of the experimental isotherms (blue,[S164] orange,[S164] and green[S163]) with the computational isotherm for CO_2.

5.3.8 UiO-66-NH2

UiO-66 is a benchmark Zr-MOF built up from Zr(IV)-hexanuclear oxo-clusters (as nodes) linked together by terephthalate (X-BDC; X=H, NH_2, COOH, halides, etc.) linkers, to yield a cubic structure endowed with octahedral and tetrahedral cages. It can be synthesized with linkers that have different functional groups (e.g., NH_2, NO_2, and Br).[S165,S166] The chemical formula of UiO-66-NH2 which is obtained with the linker 2-amino-1,4-benzenedicarboxylate ($H_2N{-}BDC$) is $Zr_6H_{34}C_{48}N_6O_{32}$. The inorganic building unit is a Zr_6-cluster and the linker is 2-amino-1,4- benzenedicarboxylate (H_2N-BDC).

In Figure S21, we compare the experimental isotherms of Hu et al.[S149], Cmarik et al.[S150], and Zhang et al.[S151] with the computational ones for CO_2.

For the N_2 isotherms, we obtain excellent agreement. For CO_2, We observe a similar overestimation of the isotherms at the Henry regime as for UiO-66 (see Section 5.3.5).

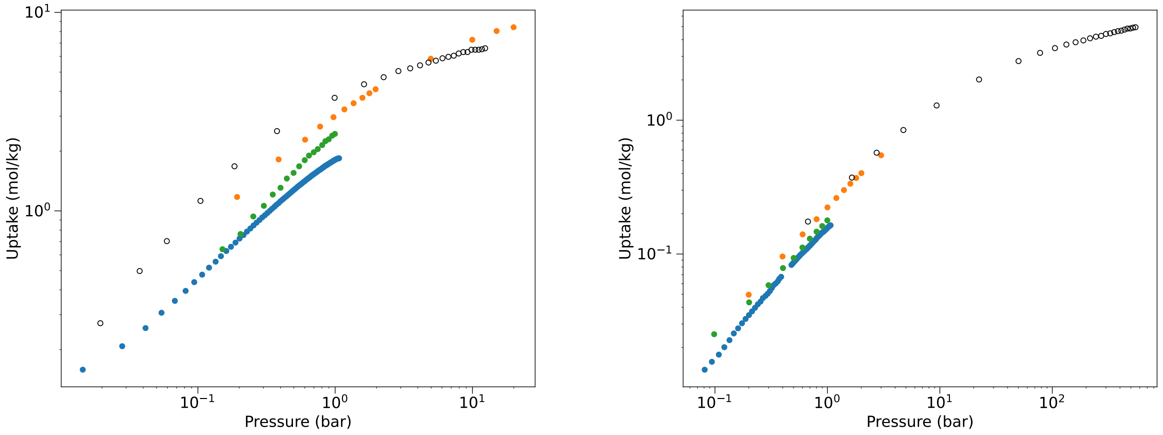

(a) - CO_2 **(b)** - N_2

Figure S21| UiO-66-NH2 | Comparison of the experimental isotherms (blue,[S149] orange,[S150] and green[S151]) with the computational isotherms (open symbols) for (a) CO_2 and (b) N_2.

5.3.9 UiO-67

UiO-67 was first synthesized by Cavka et al.[S146]. This MOF is isoreticular to UiO-66-NH2 but obtained with the extend linker 4,4-biphenyl-dicarboxylate (BPDC). Its chemical formula is $Zr_6H_{48}C_{84}O_{32}$.

In Figure S22, we compare the experimental isotherms of Yoon and Moon[S167] with the computational one for CO_2. We see that our model is in excellent agreement.

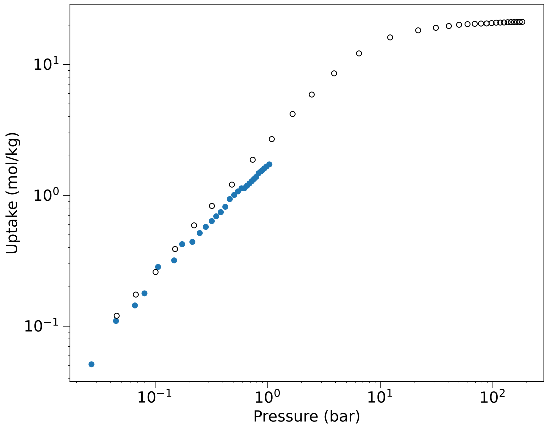

Figure S22| UiO-67 | Comparison of the experimental isotherm (blue[S167]) with the computational isotherm for CO_2.

5.3.10 HKUST-1

HKUST-1 ([$Cu_3(TMA)_2(H_2O)_3$]n, where TMA is benzene-1,3,5-tricarboxylate) has intersecting nanochannels that form a 3D connected network of pores. The chemical formula for HKUST-1 is $Cu_{12}H_{24}C_{72}O_{48}$. HKUST-1 was first synthesized by Chui et al.[S168], and since then, many groups have synthesized this material (see, for example,[S169]).

In Figure S23, we compare the experimental isotherms,[S170–S175] and Millward and Yaghi[S162] with the computational ones for CO_2 and N_2.

The predicted isotherms for CO_2 and N_2 are in excellent agreement with the experimental data.

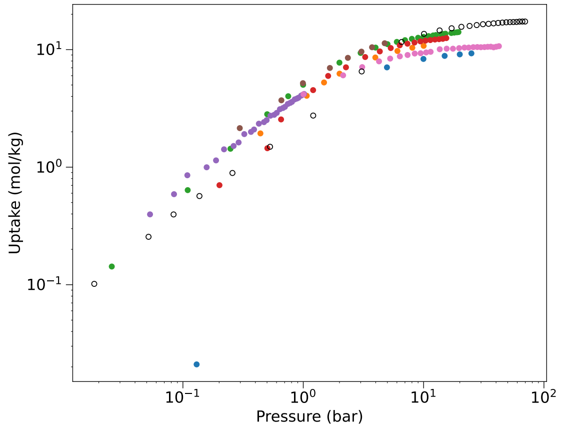

(a) - CO_2

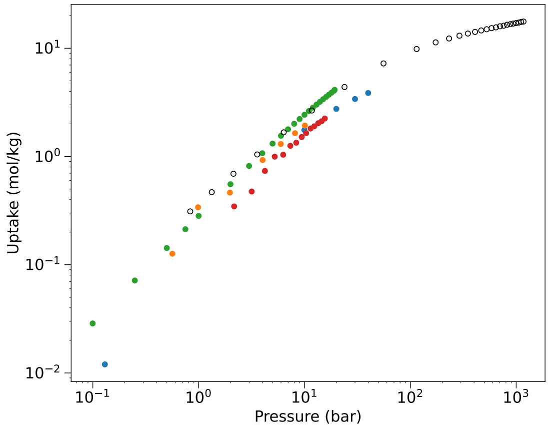

(b) - N_2

Figure S23| HKUST-1 | Comparison of the experimental isotherms (blue,[S170] orange,[S171] green,[S172] red,[S173] purple,[S174] brown,[S175] and pink[S162]) with the computational isotherms (open symbols) for (a) CO_2 and (b) N_2.

5.3.11 UMCM-1

UMCM-1 was first synthesized by Koh et al.[S176]. It has a chemical formula of $Zn_{24}H_{144}C_{264}O_{78}$. UMCM is built of and has Zn_4O as the inorganic node and a mixture of linkers, the terephthate (BDC) and 1,3,5-benzene-tris(4-benzene-benzoate (BTB). The framework of UMCM-1 consists of Zn_4O clusters linked together by two BDC and four BTB linkers arranged in an octahedral geometry.

In Figure S24, we compare the experimental isotherms of Mu et al.[S177] and Xiang et al.[S178] with the computational ones for CO_2 and N_2.

The predicted isotherms for CO_2 and N_2 are in reasonable agreement with the experimental data. For CO_2 and N_2, we overestimate the loading.

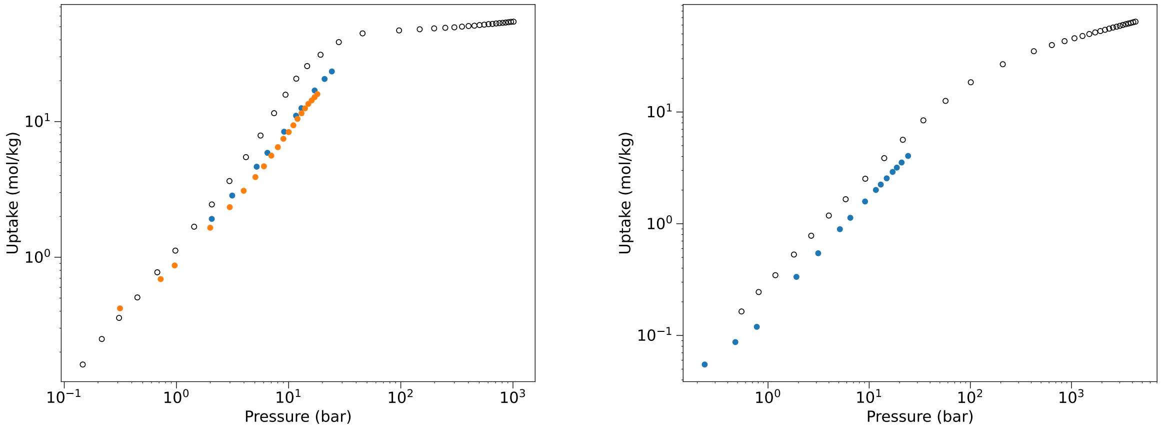

(a) - CO_2 **(b)** - N_2

Figure S24| UMCM-1 | Comparison of the experimental isotherms (blue[S177] and orange[S178]) with the computational isotherms (open symbols) for (a) CO_2 and (b) N_2.

5.3.12 Ca-SQA

Ca-SQA was first synthesized by Li et al.[S179]. The chemical formula is $Ca_8H_{16}C_{32}O_{40}$. Its 3-D structure consists of 1-D chains of edge-sharing Ca(II) polyhedra linked together by squarate ligands (the deprotonated form of the squaric acid (3,4-dihydroxy-3-cyclobutene-1,2-dione)).

In Figure S25, we compare the experimental isotherms of Tu et al.[S180] and this work with the computational ones for CO_2 and N_2. Our model for both CO_2 and N_2 overestimates the adsorption, particularly in the Henry regime.

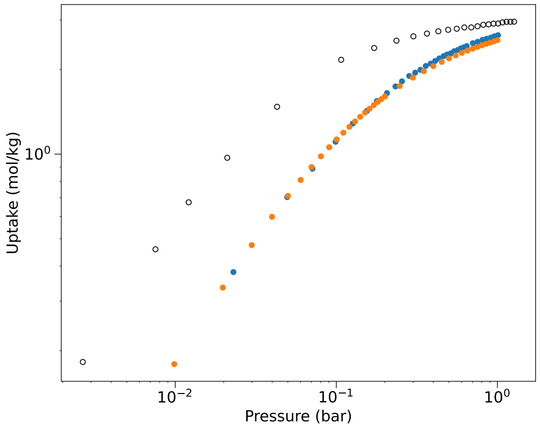

(a) - CO_2

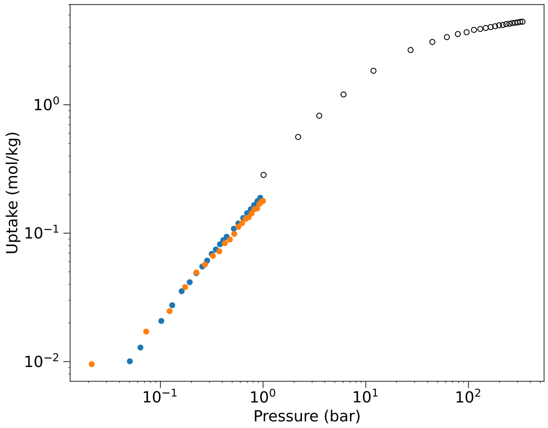

(b) - N_2

Figure S25| Ca-SQA | Comparison of the experimental isotherms (blue[S180] and orange; this work) with the computational isotherms (open symbols) for (a) CO_2 and (b) N_2.

5.4 Zeolites

5.4.1 Zeolite-13X

Zeolite-13X, also known as NaX, is a Faujasite-type zeolite. The crystal structure has a Si to Al ratio of 1.18, corresponding to 104 silicon atoms and 88 aluminum atoms, in addition to 88 sodium cations.[S181] In our simulations, the crystal structure is assumed to be rigid, while sodium ions are free to move (only translation). We used the force field developed by Vujić and Lyubartsev[S182] to generate the CO_2 and N_2 pure component isotherms. The force field developed by Joos et al.[S183] was implemented for water calculations. Joos et al.[S183] showed that this force field very well predicts the experimental water isotherms.

In Figure S26, we compare the experimental isotherms of McEwen et al.[S184], Najafi et al.[S185], Cavenati et al.[S186], Wilkins and Rajendran[S187], and Wang and LeVan[S188] with the computational ones for CO_2 and N_2. Our model for CO_2 overestimates the adsorption, particularly in the Henry regime. While, for N_2, the predicted isotherm is in excellent agreement with the experimental data.

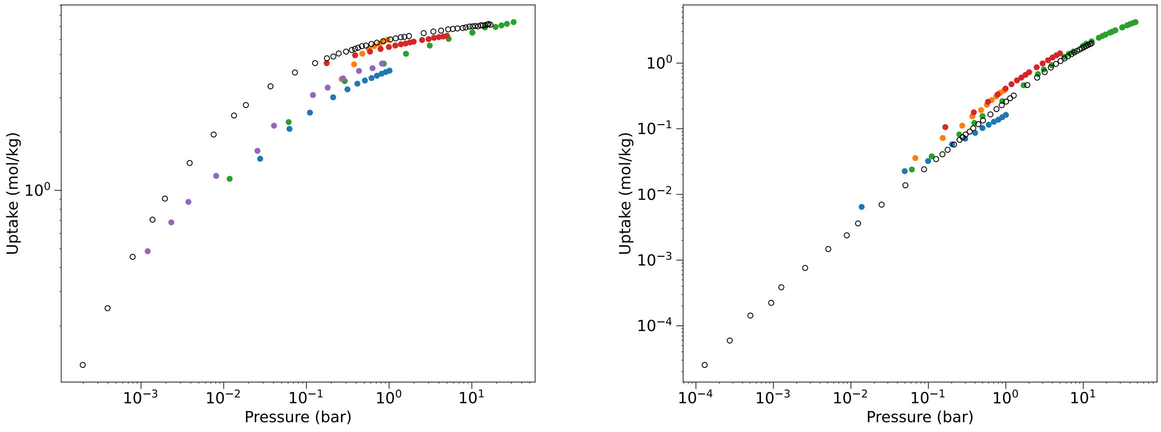

(a) - CO_2 **(b)** - N_2

Figure S26| Zeolite-13X | Comparison of the experimental isotherms (blue,[S184] orange,[S185] green,[S186] red,[S187] and purple[S188]) with the computational isotherms (open symbols) for (a) CO_2 and (b) N_2.

5.5 Discussion of the results

In the previous sections, we have compared the predicted adsorption isotherms with experimental data from the literature and data from this work. Our conclusions from this work are that except for Al-MOFs, the UFF force field gives good to excellent predictions of the CO_2 and N_2 isotherms. However, the simple scaling introduced by Boyd et al.[S33] for the Al-atoms in the MOF significantly improves the predictions. Hence, we have used this correction for all Al containing MOFs for our screening studies.

A comparison for water isotherms in MOFs is significantly more difficult because far fewer water isotherms have been measured, and these isotherms show significant scatter (see, for example, the work of Rudenko et al.[S189], Burtch et al.[S190]). For those structures we could compare, the simulated isotherms were reasonable agreement with the experimental ones. Interestingly, MOFs have recently been published for water harvesting, and we expect that much more data will become available,[S191] which will allow us to further validate our water isotherms.

For some structures, we do see significant deviations for some materials. There can be several reasons for these differences. First, our simulations assume that our MOF is a perfect, fully-activated crystal. Experimentally, it is difficult, if not impossible, to obtain such perfect crystals. Ongari et al.[S192] compared the reported (experimental) pore volume for MOFs that different groups have synthesized. For some MOFs, the reported experimental pore volumes can vary by a factor of 2 to 10. Some MOFs have been extensively studied, and for those MOFs, we know how to synthesize crystals with a pore volume close to the theoretical pore volume computed from the crystal structure. Equally important, we know how to activate these materials to remove the solvent used in the synthesis from the pores. Park et al.[S193] made a detailed comparison of the reproducibility of the adsorption isotherms and also concluded the scatter in the experimental isotherms makes it difficult to draw firm conclusions. Yet, there are a few MOFs for which such a comparison is possible, and the agreement is generally good for these MOFs.

A second assumption in our simulations is that a MOF is a rigid structure. This speeds up the calculation as we only have to generate new configurations for the adsorbed molecules. However, some MOFs change structure upon adsorption (e.g., MIL-53-NH2, see Section 5.2.4), and we fail to predict the correct adsorption behavior for such MOFs. Therefore, we flag those structures for which we expected the framework's flexibility to have a large impact (see Section 3.1.3) on the adsorption properties.

Hence, our conclusion of this comparison is that for a large screening, the Al-scaled UFF force field is sufficiently reliable to make a first selection. However, once we have identified a promising material, it is important to validate the force field further.

5.6 Error propagation

In the previous section, we have made a detailed comparison with the experimental data. From this comparison, we can make the following conclusions:

- We assume an infinite, perfect crystal. However, synthesizing such perfect crystals in practice is experimentally difficult, if not impossible. One can expect to have a 10-20% lower accessible pore volume.
- In our screening study, we assume a universal force field (UFF) with an adjustment for Al. The comparison with the experimental data shows that the error in the Henry coefficient can be as much as one order of magnitude.

To study the propagation of these uncertainties in the calculation of the material properties, we fitted the simulated pure component isotherms of CO_2 and N_2 with a Langmuir isotherm.

$$\sigma_i = \sigma_{\mathrm{sat},i} \frac{H_i p}{1 + H_i p}$$

where $\sigma_{\mathrm{sat},i}$ is the saturation loading and H_i the Henry coefficient of component i. We then used the uncertainties in the Henry coefficients and saturation loading to generate the

isotherms for which we compute the nCAC. For the other parameters, we assume a 10% error.

The resulting propagation of the uncertainties for one of the top-performing materials (Mg-MOF-74) is shown in the tornado plot in Figure S27. As expected, the most important factor is the low-pressure part of the CO_2 adsorption isotherm. The most important result is that if we are one order of magnitude off, we overestimate the nCAC by 4 €$t_{CO_2}^{-1}$.

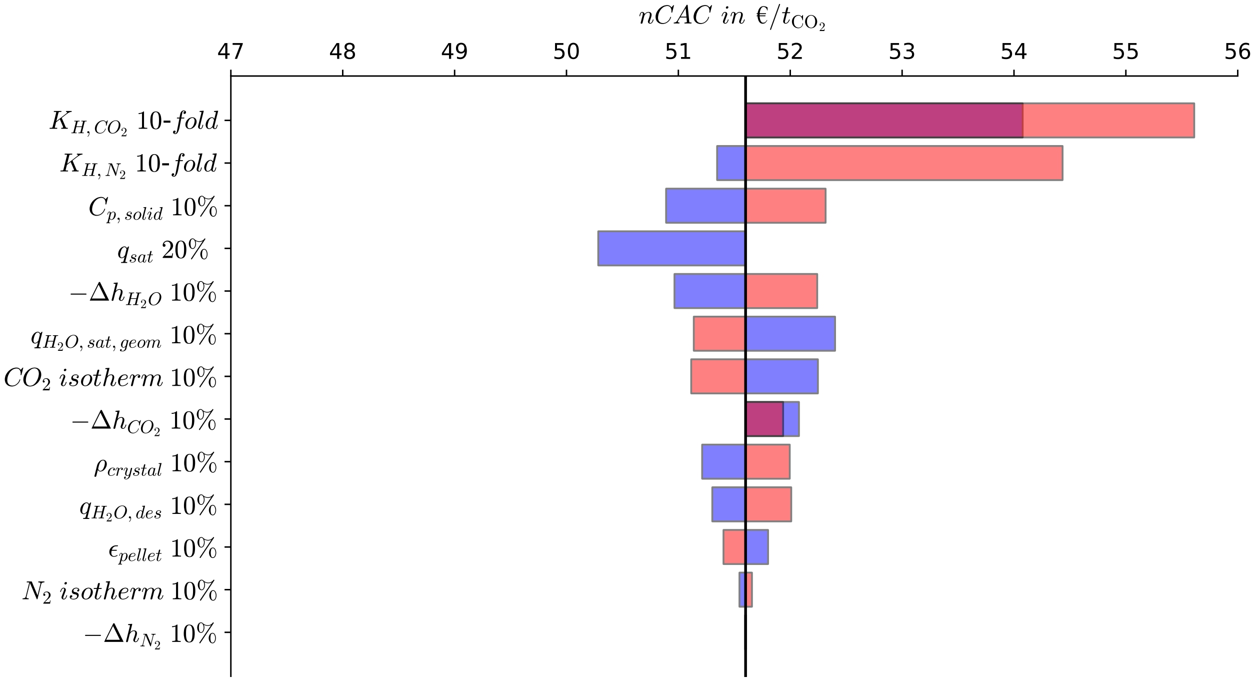

Figure S27| Tornado plot for the parameters of the materials layer We computed the change in nCAC for one of the top-performing materials (Mg-MOF-74). The parameters are ordered in decreasing impact on the nCAC. A red bar indicates a positive change in the parameters, a blue bar indicates a negative change, and a purple bar indicates if both a positive and a negative change have the same effect.

6 Output of the PrISMa platform

The PrISMa platform generates two types of outputs: Key Performance Indicators (KPIs) and Foreground data (FG). The KPIs are used to measure the performance of the materials or process. FG are data computed in one platform layer and used in the next layer. At the materials layer, we provide additional data that can be used to test the accuracy of the IAST approximation and indicate the materials' affinity towards water.We also flag the potential flexibility of the structure. Table S5 summarizes the output of the PrISMa platform.

Table S5: Summary of all Key Performance Indicators (KPIs) and foreground data (FG) generated by the PrISMa platform.

No	Description	Unit	Type	Section
**1. Materials**				
1.1	Henry coefficient	$mol\,kg^{-1}\,Pa^{-1}$	KPI	(6.1.1)
1.2	Henry selectivity	-	KPI	(6.1.2)
1.3	Pure component capacity	$mol\,kg^{-1}$	KPI	(6.1.3)
1.4	Water resistance coefficient	-	KPI	(6.1.4)
Foreground data for the Process layer				
1.5	Crystal density	$g\,cm^{-3}$	FG	(6.1.5)
1.6	Pure component isotherm	$mol\,kg^{-1}$ (list)	FG	(6.1.5)
1.7	Isosteric heat of adsorption	$kJ\,mol^{-1}$ (list)	FG	(6.1.5)
1.8	Average heat of adsorption	$kJ\,mol^{-1}$	FG	(6.1.5)
1.9	H_2O desorption loading	$mol\,kg^{-1}$	FG	(6.1.5)
1.10	Heat capacity	$J\,g^{-1}\,K^{-1}$	FG	(6.1.5)
Flag data				
1.11	IAST check	-	Flag	(6.1.6)
1.12	Flexibility	-	Flag	(6.1.6)
**2. Process**				
2.1	Purity	%	KPI	(6.2.1)
2.2	Recovery	%	KPI	(6.2.2)
2.3	Effective recovery	%	KPI	(6.2.3)
2.4	Working capacity	$mol\,m^{-3}$	KPI	(6.2.4)
2.5	Productivity	$mol\,kg^{-1}\,h^{-1}$	KPI	(6.2.5)
2.6	Specific thermal energy for heating	MJ_{th}/mol	KPI	(6.2.6)
2.7	Specific thermal energy for cooling	MJ_{th}/mol	KPI	(6.2.7)
2.8	Specific electrical energy	MJ_{el}/mol	KPI	(6.2.8)
Foreground data for the TEA layer				
2.9	Cycle time and step times (list)	s	FG	(6.2.9)
2.10	CO_2 captured	$kg\,h^{-1}$	FG	(6.2.9)
2.11	Water-loaded fraction of the sorbent bed, α	-	FG	(6.2.9)

Table S5: (continued)

No	Description	Unit	Type	Section
2.12	Amount of product of each gas at HS (list)	mol	FG	(6.2.9)
2.13	Amount of waste of each gas at VS (list)	mol	FG	(6.2.9)
2.14	List of P, T and molar fraction of CO_2 at vacuum	kPa, K, -	FG	(6.2.9)
2.15	Bed density	$kg\,m^{-3}$	FG	(6.2.9)
**3. TEA (entire capture plant)**				
3.1	CAPEX	M€	KPI	(6.3.1)
3.2	OPEX	M€	KPI	(6.3.2)
3.3	CAC	€$\,t^{-1}$	KPI	(6.3.3)
3.4	nCAC	€$\,t^{-1}$	KPI	(6.3.4)
3.5	LCOC	€$\,t^{-1}$	KPI	(6.3.5)
3.6	CCC	€$\,t^{-1}$	KPI	(6.3.6)
3.7	LCOE	€$\,MW^{-1}\,h$	KPI	(6.3.7)
3.8	CO_2 avoided	$kg\,h^{-1}$	KPI	(6.3.8)
3.9	Specific CO_2 emissions	$kgCO_2$/ unit	KPI	(6.3.9)
3.10	SPECCA	$MJ\,kg^{-1}$	KPI	(6.3.10)
Foreground data for the LCA layer				
3.11	Total mass of CO_2 captured	kg	FG	(6.3.11)
3.12	Total mass of required adsorbent material	kg	FG	(6.3.11)
3.13	Total amount of parasitic load	MJ	FG	(6.3.11)
3.14	Total heat amount supplied by each utility system	MJ_{th}	FG	(6.3.11)
3.15	Total power amount supplied by each utility system	MJ_{el}	FG	(6.3.11)
3.16	Total cooling amount supplied by each utility system	MJ_{th}	FG	(6.3.11)
3.17	Total mass of carbon steel of the equipment	kg	FG	(6.3.11)
**4. LCA (functional unit: 1 kg CO_2-captured)**				
4.1	Climate Change	kg CO_2-Eq	KPI	(6.4.1)
4.1.1	Climate Change: Biogenic	kg CO_2-Eq	KPI	(6.4.1)
4.1.2	Climate Change: Fossil	kg CO_2-Eq	KPI	(6.4.1)
4.1.3	Climate Change: Land Use and Land Use Change	kg CO_2-Eq	KPI	(6.4.1)
4.2	Water Use	m^3 world eq. deprived	KPI	(6.4.2)
4.3	Energy Resources: Non-Renewable	MJ, net calorific value	KPI	(6.4.2)
4.4	Material Resources: Metals/Minerals	kg Sb-Eq	KPI	(6.4.2)
4.5	Land Use	-	KPI	(6.4.2)
4.6	Acidification	mol H+-Eq	KPI	(6.4.3)
4.7	Ecotoxicity: Freshwater	CTUe	KPI	(6.4.3)
4.7.1	Ecotoxicity: Freshwater, Inorganics	CTUe	KPI	(6.4.3)
4.7.2	Ecotoxicity: Freshwater, Metals	CTUe	KPI	(6.4.3)
4.7.3	Ecotoxicity: Freshwater, Organics	CTUe	KPI	(6.4.3)

Table S5: (continued)

No	Description	Unit	Type	Section
4.8	Eutrophication: Freshwater	kg PO_4-Eq	KPI	(6.4.3)
4.9	Eutrophication: Marine	kg N-Eq	KPI	(6.4.3)
4.10	Eutrophication: Terrestrial	mol N-Eq	KPI	(6.4.3)
4.11	Human Toxicity: Carcinogenic	CTUh	KPI	(6.4.4)
4.11.1	Human Toxicity: Carcinogenic, Inorganics	CTUh	KPI	(6.4.4)
4.11.2	Human Toxicity: Carcinogenic, Metals	CTUh	KPI	(6.4.4)
4.11.3	Human Toxicity: Carcinogenic, Organics	CTUh	KPI	(6.4.4)
4.12	Human Toxicity: Non-Carcinogenic	CTUh	KPI	(6.4.4)
4.12.1	Human Toxicity: Non-Carcinogenic, Inorganics	CTUh	KPI	(6.4.4)
4.12.2	Human Toxicity: Non-Carcinogenic, Metals	CTUh	KPI	(6.4.4)
4.12.3	Human Toxicity: Non-Carcinogenic, Organics	CTUh	KPI	(6.4.4)
4.13	Particulate Matter Formation	disease incidence	KPI	(6.4.4)
4.14	Ozone Depletion	kg CFC-11-Eq	KPI	(6.4.4)
4.15	Photochemical Ozone Formation: Human Health	kg NMVOC-Eq	KPI	(6.4.4)
4.16	Ionising Radiation: Human Health	kBq U235-Eq	KPI	(6.4.4)

6.1 Output at the materials level

6.1.1 Henry coefficient

The Henry coefficients in $\mathrm{mol\,kg^{-1}\,Pa^{-1}}$ for CO_2, N_2, and H_2O at the reference temperature (298.15 K) as obtained from the NVT simulations (see Section 3.1.1).

6.1.2 Henry selectivity

Henry selectivity of species i over species j is calculated from Henry's coefficient of each adsorbable component (see Section 6.1.1) and is governed by

$$\text{Selectivity}_{i/j} = \frac{K_{Hi}}{K_{Hj}}. \tag{S34}$$

Here, component i is CO_2 and component j is N_2.

6.1.3 Pure component capacity

Pure component capacity in $\mathrm{mol\,kg^{-1}}$ of species i is obtained from the pure component isotherm corresponding to that species at the adsorption conditions of temperature and pressure ($T = 310.15\,\mathrm{K}$ and $P = 1.013\,25\,\mathrm{bar}$). Here, component i is CO_2 or N_2.

6.1.4 Water resistance coefficient

This property measures the impact of water on the capacity of the material to adsorb CO_2. It is defined as the ratio of the amount of CO_2 adsorbed in the material from a wet flue gas over the amount of CO_2 adsorbed in the material from a dry flue gas. The adsorption data have been obtained from GCMC calculations at the adsorption conditions of temperature and partial pressures for the wet and dry flue gases and is governed by

$$q_{\mathrm{ratio}} = \frac{q_{\mathrm{CO_2,\,ternary}}}{q_{\mathrm{CO_2,\,binary}}}. \tag{S35}$$

The adsorption data obtained from the GCMC calculations also allow us to compute the ternary selectivity of species i over species j that is governed by

$$\mathrm{Selectivity}_{i/j}^{\mathrm{tern}} = \frac{q_i}{q_j}\frac{y_j}{y_i}. \tag{S36}$$

y_i and y_j are the compositions of either CO_2 or N_2 and either N_2 or H_2O in the ternary mixture, respectively. Here, we compute $\mathrm{Selectivity}_{\mathrm{CO_2/N_2}}^{\mathrm{tern}}$, $\mathrm{Selectivity}_{\mathrm{CO_2/H_2O}}^{\mathrm{tern}}$, and $\mathrm{Selectivity}_{\mathrm{N_2/H_2O}}^{\mathrm{tern}}$.

6.1.5 Foreground data for the Process layer

Crystal density The density of the crystal in $\mathrm{g\,cm^{-3}}$ as obtained from Zeo^{++} calculations (see Section 3.1.1).

Pure component isotherm The pure component isotherms in mol kg^{-1} for CO_2 and N_2 at the reference temperature (298.15 K) as obtained from the GCMC simulations (see Section 3.1.1).

Isosteric heat of adsorption The filtered loading-dependent heat of adsorption in kJ mol^{-1} of N_2 and CO_2 as obtained from the GCMC simulations at the reference temperature (298.15 K) (see Section 3.1.1).

Average heat of adsorption The average heat of adsorption in kJ mol^{-1} of N_2 and CO_2 as calculated from the filtered loading-dependent heat of adsorption given in Section 6.1.5. For H_2O, we use the heat of adsorption at the desorption conditions.

Water desorption loading The loading mol kg^{-1} of H_2O at the desorption conditions is calculated using single-point GCMC simulations. The desorption pressure of water ($P_{\mathrm{des,H_2O}}$), at the process desorption temperature (393.15 K), is obtained using[S194]

$$P_{\mathrm{des,H_2O}} = P_{\mathrm{des,pro}} \frac{P_{\mathrm{sat,FF}}}{P_{\mathrm{sat,exp}}}, \tag{S37}$$

where $P_{\mathrm{des,pro}}$ is the process desorption pressure (1.013 25 bar), $P_{\mathrm{sat,exp}}$ is the experimental water saturation pressure (1.9886 bar), and $P_{\mathrm{sat,FF}}$ is the water saturation pressure as predicted by the force field (0.689 bar).[S195]

Heat capacity The heat capacity in J g^{-1} K^{-1}, as predicted by our machine learning model (see Section 3.1.1).

6.1.6 Flags at the materials layer

IAST check To test the accuracy of IAST for a binary mixture at the adsorption conditions of temperature and partial pressures, we compare the IAST predictions with the GCMC calculations. As a measure of the accuracy, we use the logarithm of the ratio:

$$\text{ratio}_i = \left| \log \left(\frac{q_i, \text{IAST}}{q_i, \text{GCMC}} \right) \right| . \tag{S38}$$

Here, component i is CO_2 or N_2.

Flexibility check We use the relative standard deviation of the Henry coefficients obtained during a molecular dynamics simulation, in which we allow the material's atoms to move. If this standard deviation is above a critical threshold, we flag the structure as flexible (see Section 3.1.3 for details).

6.2 Output at the process level

6.2.1 Purity

Purity in % is defined by the molar fraction of the key species, in this case, CO_2, in the product stream and is determined by the amount of CO_2 product collected at the outlet of the compressor train, $n_{CO_2,\text{ out}}$ (mol_{CO_2}), divided by the total amount of each species i (total products) that leaves the column at outlet of the compression train, as

$$\text{Purity}_{\ CO_2} = \frac{n_{CO_2,\text{ out}}}{\sum_i^{N_\text{comp}} n_{i,\text{ out}}} \cdot 100\,\%. \tag{S39}$$

Here, N_comp refers to the number of components considered in the adsorption process.

6.2.2 Recovery

Recovery in % is defined by the amount of the key species, in this case CO_2, collected at the Heating Step (HS) divided by the total amount of that species entering the column at the Adsorption Step (AS), $n_{CO_2,\text{ in(AS)}}$ (molCO_2), and the Cooling Step (CS), $n_{CO_2,\text{ in(CS)}}$, as

$$\text{Recovery}_{\,CO_2} = \frac{n_{CO_2,\text{ out (HS)}}}{n_{CO_2,\text{ in (AS)}} + n_{CO_2,\text{ in (CS)}}} \cdot 100\,\%. \tag{S40}$$

6.2.3 Effective recovery

Effective recovery in % is defined as the recovery corrected by the CO_2-eq. emissions of the entire life cycle of the carbon capture plant and defines the effective amount of CO_2 that is avoided. The effective recovery can be calculated from the recovery and the climate change impact CC, evaluated in the LCA layer, as

$$\text{Eff. recovery}_{\,CO_2} = \text{Recovery}_{\,CO_2} \cdot (1 - \text{CC}). \tag{S41}$$

6.2.4 Working Capacity

The working capacity in $\text{mol}\,\text{m}^{-3}$ is defined by the amount of CO_2 (mol) product collected at Heating Step (HS) per m^3 as

$$\text{Working Capacity}_{\,CO_2} = \frac{n_{CO_2,\text{ out (HS)}}}{V_{\text{bed}}}. \tag{S42}$$

The reduction in working capacity of CO_2 due to competitive co-adsorption, i.e., the impact of water, is considered by using the computed L_{H_2O} (see Section 3.2.1) to define $q_{CO_2,\text{wet}}$ ($\text{mol}\,\text{kg}^{-1}$). The local equilibrium molar loadings for the adsorbent during the feed adsorption step for the water-loaded and water-free zones within the column are taken respectively

from the ternary and binary GCMC simulations (see Section 3.1.2). The overall expression for $q_{\mathrm{CO_2,wet}}$ is then

$$q_{\mathrm{CO_2,wet}} = \begin{cases} \mathrm{WRC} \cdot q_{\mathrm{CO_2,dry}} & Z \leq L_{\mathrm{H_2O}} \\ q_{\mathrm{CO_2,dry}} & Z > L_{\mathrm{H_2O}} \end{cases} \tag{S43}$$

where Z (m) is the position in the column.

The working capacity considering the impact of H_2O sorption, can then be calculated as the weighted average of the respective working capacities for the dry and wet parts:

$$\text{Working Capacity}_{\mathrm{CO_2}} \approx \alpha \text{ Working Capacity}_{\mathrm{CO_2,wet}} + (1-\alpha)\text{Working Capacity}_{\mathrm{CO_2,dry}} \tag{S44}$$

where α gives the fraction of the sorbent bed that is water-loaded at the end of the feed adsorption step at CSS (cf. Equation (S50)).

6.2.5 Productivity

Productivity in $\mathrm{mol\,kg^{-1}\,h^{-1}}$ is defined by the working capacity (cf., Equation (S44)) and the cycle time, t_{cycle}, as

$$\text{Productivity}_{\mathrm{CO_2}} = \text{Working Capacity}_{\mathrm{CO_2}} \frac{1}{\rho_{\mathrm{bulk}}\, t_{\mathrm{cycle}}} \tag{S45}$$

t_{cycle} (h) is the total time that is required to operate a complete cycle of the process. For the five-step TVSA, it is defined by the predicted time required to complete the AS, VS, IHS, HS, and CS (see Section 3.2.2). ρ_{bulk} ($\mathrm{kg\,m^{-3}}$) is the bulk bed density which is defined in Equation (S51) (see Section 6.2.9).

6.2.6 Specific thermal energy

The specific thermal energy for heating, q_{th} in $\mathrm{kJ\,mol^{-1}}$, is defined as the sum of the sensible heat and multi-component enthalpy of adsorption (cf. Equation (S9) in Section 3.2.3).

6.2.7 Specific thermal energy for cooling

The specific thermal energy for cooling, $q_{\text{th, cooling}}$ in kJ mol^{-1}, is the sum of the energy required for the CS in the capture process and the cooling duty of the direct contact cooler, of the condenser, and of an inter-cooled compressor if, for a particular case study, the CO_2 sink needs a compressor (cf. Equation (S13) in Section 3.2.3).

6.2.8 Specific electrical energy

The specific electrical energy, w_{el} in kJ mol^{-1}, is defined as the energy needed to operate the booster fan, the vacuum pump and the compressor[2] and is derived from the expression of the polytropic compression work (cf. Equation (S13) in Equation (S23)).

6.2.9 Foreground data for the TEA layer

Cycle time and step times. The cycle time, t_{cycle} (h), is the total time required to operate all steps that a single column undergoes, and it is defined as:

$$t_{\text{cycle}} = t_{(\text{AS})} + t_{(\text{VS})} + t_{(\text{IHS})} + t_{(\text{HS})} + t_{(\text{CS})}. \tag{S46}$$

Section 3.2.2 describes how we compute the time to operate each step.

CO_2 captured. Most economic KPIs depend on the scale of the capture plant. The basis for the scaling is provided by the total amount of CO_2 that is captured, $\dot{m}_{CO_2\text{ captured}}$ in kgCO_2/h, in the process and is defined as

$$\dot{m}_{CO_2\text{ captured}} = \frac{n_{CO_2\text{, out (HS)}}\, N_{\text{columns}}\, N_{\text{trains}}\, \text{MW}_{CO_2}}{t_{\text{cycle}}}. \tag{S47}$$

[2]This compression step will depend on the CO_2 sink.

Here, $n_{CO_2,\,\text{out (HS)}}$ (molCO_2) is the amount of CO_2 obtained per column and, therefore, the total amount of CO_2 product is obtained by multiplying with N_{columns}, the number of columns in the capture plant per train, and N_{trains}, the number of trains in the capture plant:

$$N_{\text{columns}} = \frac{t_{\text{cycle}}}{t_{\text{(AS)}}} \tag{S48}$$

$$N_{\text{trains}} = \frac{\dot{n}_{\text{feed, tot}}}{\dot{n}_{\text{feed}}} \tag{S49}$$

where $\dot{n}_{\text{feed, tot}}$ is the total molar flow rate of the feed stream to the capture plant ($\text{mol}\,\text{s}^{-1}$) and $\dot{n}_{\text{feed}}$ is the molar flow rate of the feed stream to the column ($\text{mol}\,\text{s}^{-1}$). MW_{CO_2} in $\text{kg}\,\text{mol}^{-1}$ is the molecular weight of CO_2.

Water-loaded fraction of the sorbent bed α describes the fraction of the sorbent bed, which is water-loaded at the end of the feed adsorption step at Cyclic Steady state.

$$\alpha = \frac{L_{H_2O}}{L_{CO_2}} = \frac{1}{\text{Recovery}_{CO_2}} \frac{WC_{CO_2}}{WC_{H_2O}} \frac{y_{H_2O,\text{feed}}}{y_{CO_2,\text{feed}}} \tag{S50}$$

Amount of product of each gas at HS. A list containing the total amount of product (mol) of each species collected at the end of the Heating Step per column.

Amount of waste of each gas at VS. A list containing the total amount of waste (mol) of each species exiting the column at the end of the steps where vacuum is applied, i.e., at the end of the Vacuum Step, Intermediate Heating Step, and Heating Step.

List of P, T, and molar fraction of CO_2 at vacuum. This list contains information on the operation of the vacuum step (i.e., bulk-gas pressure in the column, column temperature, molar fraction of CO_2 in the bulk-gas phase of the column).

Bed density. The bed density ρ_{bulk} in kg m^{-3} is calculated in the process model using material parameters (i.e., crystal density, pellet density, and pellet porosity) and column characteristics (i.e., bed porosity):

$$\rho_{\text{bulk}} = (1.0 - \epsilon_{\text{bed}})\, \rho_{\text{pellet}}. \tag{S51}$$

Here, ϵ_{bed} is the void fraction of the bed (tubes, in case of packed multi-tubular heat exchanger column). In this study, we use $\epsilon_{\text{bed}} = 0.37$. ρ_{pellet} in kg m^{-3} presents the density of the pellet, which can be either known/measured experimentally or defined by the pellet void fraction, ϵ_{pellet}, and the density of the solid/crystal, ρ_{crystal} (from the materials layer, see Section 6.1.5), as

$$\rho_{\text{pellet}} = (1.0 - \epsilon_{\text{pellet}})\, \rho_{\text{crystal}}. \tag{S52}$$

In this work, we assume $\epsilon_{\text{pellet}} = 0.35$.

6.3 Output at the TEA layer

6.3.1 Capital Expenditure (CAPEX)

The Capital Expenditure (CAPEX) of the capture plant, in M€, is calculated by following the guidelines of Woods[S73], which are based on the Bare Module method for developing screening cost estimates (see Section 3.3.2 for details).

6.3.2 Operational Expenditure (OPEX)

The Operational Expenditure (OPEX), in M€, is calculated annually and takes into account all costs to operate the plant. It is calculated as the sum of fixed operational costs, variable operational costs, and adsorbent replacement (see Section 3.3.3). Please note that we consider solely the OPEX from a plant's operator perspective as KPI and not the OPEX from the system's perspective.

6.3.3 Carbon Avoidance Cost (CAC)

CAC in €$\,t^{-1}$ is a metric that quantifies the cost incurred in avoiding a mass unit of CO_2 into the atmosphere considering the energy-related life cycle CO_2-eq. emissions of the capture plant. For power generation case studies, CAC is calculated from the levelized cost of electricity and the carbon intensity according to:

$$\text{CAC} = \frac{\text{LCOE}_{\text{cc}} - \text{LCOE}_{\text{ref}}}{e_{\text{CO}_2\text{ intensity, ref}} - e_{\text{CO}_2\text{ intensity, cc}}} \tag{S53}$$

In Equation (S53), LCOE_{ref} and LCOE_{cc} (€/MWh) represent the levelized cost of electricity for a reference power plant without and with carbon capture, respectively. $e_{\text{CO}_2\text{ intensity, ref}}$ (tCO_2/MWh) represents the total CO_2-eq. emissions per unit of electricity for the reference power plant without carbon capture and is calculated as the sum of the direct CO_2-eq emissions as defined by the case study and the indirect CO_2-eq emissions estimated based on LCIA data taken from commercial LCIA databases. $e_{\text{CO}_2\text{ intensity, cc}}$ (tCO_2/MWh) indicates the total CO_2-eq. emissions per unit of electricity produced for the power plant with carbon capture considering the energy-related life cycle CO_2-eq. emissions of the capture plant (for details, see Section 6.3.9).

For industrial case studies, Roussanaly et al.[S196] have shown that Equation (S53) reduces to Equation (S54), if the production of the industrial site is not affected by the integration of the carbon capture plant.

$$\text{CAC} = \frac{\text{TCR}_{\text{cc}} + \sum \frac{\text{OPEX}_{\text{cc}}(yr)}{(1+DCF)^{yr}}}{\sum \frac{\dot{m}_{\text{CO}_2\text{ avoided}} hr(yr)}{(1+DCF)^{yr}}} \tag{S54}$$

In this equation, TCR is the Total Capital Required, which equals CAPEX plus the interest during construction, which is estimated according to EBTF guidelines[S75]. $\dot{m}_{\text{CO}_2\text{ avoided}}$ are the CO_2-eq. emissions avoided as defined in Section 6.3.8, and $hr(yr)$ are the operating hours

per year of the plant during its lifetime. These calculation methods have been implemented in the PrISMa platform to estimate the CAC (€ t^{-1}).

6.3.4 Net Carbon Avoidance Cost (nCAC)

The nCAC in € t^{-1} is a metric that quantifies the overall cost incurred in avoiding a mass unit of CO_2 into the atmosphere over the entire life cycle of the process, i.e., considering the total life cycle CO_2-eq. emissions of the capture plant. In contrast to the CAC, the nCAC considers the emissions of the entire life cycle of the capture process, e.g., the additional CO_2-eq. emissions from the material's synthesis, disposal, and plant construction, and not solely the energy-related life cycle CO_2-eq. emissions of the capture plant (cf. Section 6.3.9). The nCAC is defined from a system's perspective and thus also accounts for the costs and emissions associated with the compensation of the loss of product in case the production is affected by the carbon capture process (e.g., for power plant cases). This KPI thus requires input from the LCA layer.

The nCAC can be calculated according to Equation (S55)

$$\text{nCAC} = \frac{\text{TCR}_{\text{cc}} + \sum \frac{\text{OPEX}_{\text{cc}}(yr)}{(1+DCF)^{yr}}}{\sum \frac{\dot{m}_{\text{CO}_2\text{ avoided NET}} hr(yr)}{(1+DCF)^{yr}}} \tag{S55}$$

where $\text{OPEX}_{\text{cc}}(yr)$ denotes the system-based operational cost as defined in Section 6.3.2 and the $\dot{m}_{\text{CO}_2\text{ avoided NET}}$ considers the amount of CO_2 avoided considering the total CO_2-eq. emissions over the entire life cycle of the capture plant as calculated in the LCA layer. Equation (S55) can be written in terms of the system-based LCOC or CCC, which are identical for the system's perspective (see Section 6.3.5 and Section 6.3.6) and the climate change impact CC evaluated in the LCA layer, as illustrated in Equation (S56)

$$\text{nCAC} = \frac{\text{LCOC}}{1 - \text{CC}} = \frac{\text{CCC}}{1 - \text{CC}}. \tag{S56}$$

The nCAC is larger than the CAC defined in Section 6.3.3 due to the consideration of the non-energy-related life cycle CO_2-eq. emissions from the LCA in the denominator and the additional costs and emissions from the compensation. The nCAC is thus preferred as a KPI compared to the CAC from an environmental perspective.

6.3.5 Levelised Cost of Capture (LCOC)

The Levelised Cost of Capture (LCOC) is a metric presented in the Techno-economic Methodology Report by BEIS[S197], and it is calculated as the discounted lifetime cost of building and operating a CO_2 capture system (€ t^{-1}).

$$\mathrm{LCOC} = \frac{\mathrm{TCR_{cc}} + \sum \frac{\mathrm{OPEX_{cc}}(yr)}{(1+DCF)^{yr}}}{\sum \frac{\dot{m}_{\mathrm{CO_2\ captured}} hr(yr)}{(1+DCF)^{yr}}}. \tag{S57}$$

See Section 6.3.3 for the definition of the parameters.

6.3.6 Carbon Capture Cost (CCC)

The Carbon Capture Cost (CCC) is a metric that quantifies the cost incurred in capturing a mass unit of CO_2 (€ t^{-1}). This metric is used to assess the economic viability of a CO_2 capture system relative to a market price for CO_2 as an industry commodity[S198]. For industrial case studies, the CCC reduces to the LCOC if the production of the industrial site is not affected by the integration of the carbon capture plant. This is because the LCOC does not consider the power output penalty due to the carbon capture system.

For a power generation plant, CCC can be defined as

$$\mathrm{CCC} = \frac{\mathrm{LCOE_{cc}} - \mathrm{LCOE_{ref}}}{\frac{\sum \frac{\dot{m}_{\mathrm{CO_2\ captured}} hr(yr)}{(1+DCF)^{yr}}}{\sum \frac{(\dot{W}_{\mathrm{cc}}) hr(yr)}{(1+DCF)^{yr}}}}. \tag{S58}$$

where $\dot{m}_{\mathrm{CO_2\ captured}}$ is the total mass of CO_2 captured and $\dot{W}_{\mathrm{cc}}$ is the net power output of the plant with capture. Equation (S58) reduces to Equation (S59) if the total mass of CO_2

captured and the net power output of the plant with capture is constant over the lifetime of the plant:

$$\text{CCC} = \frac{\text{LCOE}_{\text{cc}} - \text{LCOE}_{\text{ref}}}{\frac{\dot{m}_{\text{CO}_2\text{ captured}}}{\dot{W}_{\text{cc}}}} \tag{S59}$$

See Section 6.3.3 for the definition of the parameters.

For the nCAC (Section 6.3.4) calculation, the CCC considers the system-based operational cost (cf. Section 3.3.3) and a power output equal to the reference power plant without carbon capture due to the compensation of the parasitic load. In this case, the definition of CCC from a system's perspective reduces to the LCOC defined in Equation (S57) when the system-based operational cost is used. For industrial plants, the system-based CCC is equal to the operator-based CCC if the production of the industrial site is not affected by the integration of the carbon capture plant.

6.3.7 Levelized Cost Of Electricity (LCOE)

LCOE is the discounted lifetime cost of building and operating a generation asset, expressed as a cost per unit of electricity generated (€/MWh).[S199] In the PrISMa platform, the levelized cost of electricity of the power plant with carbon capture LCOE_{cc} encompasses the capital costs of the generator (power plant), which is assumed to be the same after the integration of carbon capture, the capital costs of the capture plant, all relevant costs for operating the power plant and the capture plant. It excludes financing costs. The LCOE is calculated according:

$$\text{LCOE}_{\text{ref}} = \frac{\text{TCR}_{\text{ref}} + \sum \frac{\text{OPEX}_{\text{ref}}(yr)}{(1+DCF)^{yr}}}{\sum \frac{\dot{W}_{\text{ref}} hr(yr)}{(1+DCF)^{yr}}} \tag{S60}$$

$$\text{LCOE}_{\text{cc}} = \frac{\text{TCR}_{\text{ref}} + \sum \frac{\text{OPEX}_{\text{ref}}(yr)}{(1+DCF)^{yr}} + \text{TCR}_{\text{cc}} + \sum \frac{\text{OPEX}_{\text{cc}}(yr)}{(1+DCF)^{yr}}}{\sum \frac{\dot{W}_{\text{cc}} hr(yr)}{(1+DCF)^{yr}}} \tag{S61}$$

where TCR_{ref} is the total capital required (€) including interest during construction for the reference plant, DCF is the discount cash flow rate, TCR_{cc} is the total capital required for the

capture plant, and $\dot{W}_{\text{ref}}$ and $\dot{W}_{\text{cc}}$ are the power output of the reference power plant and the power plant with carbon capture, respectively, and $hr(yr)$ represents the annual operating time in hours.

6.3.8 Carbon Dioxide Avoided

The mass flow rate of CO_2 avoided quantifies the total CO_2 emission reduction from a capture plant considering its energy-related life cycle CO_2-eq. emissions. We compute the total mass flow rate of CO_2 avoided, $\dot{m}_{\text{CO}_2\text{ avoided}}$ (kgCO_2/h), from the total mass flow rate of CO_2 captured $\dot{m}_{\text{CO}_2\text{ captured}}$ (cf., Section 6.2.9) and the total mass flow rate of CO_2 emissions of the energy supply by utilities to the capture plant, $\dot{m}_{\text{CO}_2\text{ emissions, cc}}$, as

$$\dot{m}_{\text{CO}_2\text{ avoided}} = \dot{m}_{\text{CO}_2\text{ captured}} - \dot{m}_{\text{CO}_2\text{ emissions, cc}}, \tag{S62}$$

with

$$\dot{m}_{\text{CO}_2\text{ emissions, cc}} = \sum e_{\text{CO}_2\text{ intensity, utility}} \, \dot{W}_{\text{utility}} \, N_{\text{utility}}. \tag{S63}$$

Here, $e_{\text{CO}_2\text{ intensity, utility}}$ (tCO_2/MWh) is the total CO_2-eq emission rate of a given utility (i.e., heat, cooling, power/electricity), $\dot{W}_{\text{utility}}$ (MW) is the power, heat or cooling requirement of a utility unit, and N_{utility} corresponds to the number of equipment per utility needed in the entire capture plant. The total CO_2 intensity per utility $e_{\text{CO}_2\text{ intensity, utility}}$ is taken from the commercial LCIA database and includes direct and indirect emissions of the utility. It is important to note that the CO_2-eq. emissions from 'in plant fuel use' are excluded from $\dot{m}_{\text{CO}_2\text{ emissions, cc}}$ since they are included in the CO_2 source. Moreover, the $\dot{m}_{\text{CO}_2\text{ emissions, cc}}$ do not consider the CO_2-eq. emissions from materials synthesis and disposal and capture plant construction.

6.3.9 Specific carbon dioxide emissions

The specific CO_2-eq. emissions or CO_2 intensity $e_{CO_2\ \text{intensity, cc}}$ represents the total CO_2 emitted per unit product in kgCO_2/unit of product for industrial cases with carbon capture, or per unit electricity for power plant cases with carbon capture. For power plant case studies, $e_{CO_2\ \text{intensity, cc}}$ is given by the total emissions of the CO_2 source plant with carbon capture divided by the power generation of the power plant with carbon capture, $\dot{W}_{\text{cc}}$.

For the power plant with carbon capture, $e_{CO_2\ \text{intensity, cc}}$ is calculated as:

$$e_{CO_2\ \text{intensity, cc}} = \frac{e_{CO_2\ \text{intensity, ref}} \cdot \dot{W}_{\text{ref}} - \dot{m}_{CO_2\ \text{captured}} + \dot{m}_{CO_2\ \text{emissions, cc}}}{\dot{W}_{\text{cc}}} \tag{S64}$$

For cement and other industrial cases, $e_{CO_2\ \text{intensity, cc}}$ is calculated as:

$$e_{CO_2\ \text{intensity, cc}} = \frac{e_{CO_2\ \text{intensity, ref}} \cdot \text{Product}_{\text{ref}} - \dot{m}_{CO_2\ \text{captured}} + \dot{m}_{CO_2\ \text{emissions, cc}}}{\text{Product}_{\text{ref}}}, \tag{S65}$$

where $e_{CO_2\ \text{intensity, ref}}$ (tCO_2/MWh) represents the total specific emissions of the reference plant without carbon capture, $\dot{W}_{\text{ref}}$ (MW) represents the power output of the reference plant without carbon capture, $\dot{m}_{CO_2\ \text{captured}}$ represents the total mass flow rate of CO_2 captured, $\dot{m}_{CO_2\ \text{emissions, cc}}$ represents the total mass flow rate of energy-related CO_2 emitted by the capture plant and $\text{Product}_{\text{ref}}$ is the production of an industrial plant without carbon capture, which, for industry cases, is assumed to be unaffected by the integration of carbon capture.

6.3.10 SPECCA

The Specific Primary Energy Consumption per CO_2 Avoided (SPECCA) is a metric that measures the amount of primary energy consumed in avoiding emitting a mass unit of CO_2 into the atmosphere. The calculation, as per EBTF, assumes constant thermal input of the power plant with and without carbon capture. Following the definition of the specific emission (see Section 6.3.9), the primary energy is calculated based on LCIA data taken

from commercial LCIA database and includes both direct and indirect primary energy of the reference plant and utilities. For power plants, the primary energy consumption is calculated as the sum of the direct primary energy consumption as defined by the case study heat rate ($Q_{\text{th input, ref}}/\dot{W}_{\text{ref}}$) and the indirect primary energy consumption per unit electricity ($\text{PE}_{\text{ind,ref}}$) estimated from commercial LCIA databases. In the case that the capture plant utilities consume additional primary energy, the total primary energy consumption of the utilities is added to the total primary energy consumption of the reference power plant according to:

$$\text{SPECCA} = \frac{\left(\frac{Q_{\text{th input, cc}}}{\dot{W}_{\text{cc}}} - \left(\frac{Q_{\text{th input, ref}}}{\dot{W}_{\text{ref}}} \cdot 3600 + \text{PE}_{\text{ind,ref}}\right)\right)}{e_{\text{CO}_2\text{ intensity, ref}} - e_{\text{CO}_2\text{ intensity, cc}}} \tag{S66}$$

with

$$Q_{\text{th input, cc}} = \left(\frac{Q_{\text{th input, ref}}}{\dot{W}_{\text{ref}}} \cdot 3600 + \text{PE}_{\text{ind,ref}}\right)\dot{W}_{\text{ref}} + \sum \text{PE}_{\text{utility}}\,\dot{W}_{\text{utility}}\,N_{\text{utility}}. \tag{S67}$$

Here, $\text{PE}_{\text{ind,ref}}$ denotes the indirect primary energy consumption of the reference power plant per unit electricity (MJ/MWh), $\text{PE}_{\text{utility}}$ is the total primary energy consumption of a utility per unit energy (MJ/MWh), and $e_{\text{CO}_2\text{ intensity, cc}}$ and $e_{\text{CO}_2\text{ intensity, ref}}$ are the specific CO_2 emission ($kgCO_2$/MWh) of the plant, with and without CO_2 capture, respectively. The factor 3600 is to convert $Q_{\text{th input, ref}}$ from MJ/sec to MJ/h. $\dot{W}_{\text{utility}}$ and $\dot{W}_{\text{ref}}$ are the power output of a utility unit or the reference power plant (MW), respectively. N_{utility} is the number of utilities, and $\dot{W}_{\text{cc}}$ is the power output of the power plant with carbon capture (MW).

For industrial sources, we define:

$$\text{SPECCA} = \frac{\sum \text{PE}_{\text{utility}}\,\dot{W}_{\text{utility}}\,N_{\text{utility}}}{\dot{m}_{\text{CO}_2\text{ avoided}}} \tag{S68}$$

where $\dot{m}_{\text{CO}_2\text{ avoided}}$ is defined by Equation (S62).

6.3.11 Foreground data for the LCA layer

The LCA foreground data calculated in the TEA layer provides data on the energy and material demands of the carbon capture plant during its entire life cycle.

Total mass of CO_2 captured. Indicates the total mass of CO_2 in kg that is captured during the life cycle of the capture plant. The variable is used to scale the environmental impacts to the functional unit of '1 kg CO_2 captured'.

Total mass of required adsorbent material. Indicates the total mass of adsorbent material in kg that is required during the life cycle of the capture plant. The mass includes the initial amount of material required at plant construction and the refilling after the lifetime of the material.

Total amount of parasitic load. Indicates the total parasitic load in MJ, i.e., the loss of electricity of the power plant due to the energy supply of the carbon capture plant (e.g., power supply or heat supply by steam extraction).

Total heat amount supplied by each utility system. Indicates the total amount of thermal energy for heating in MJ provided by each heat supply technology considered in the utility system. Besides the amount of thermal energy, the scenario of the heat supply technology is provided (e.g., natural gas boiler or electrical boiler).

Total power amount supplied by each utility system. Indicates the total amount of electrical energy in MJ provided by each power supply technology considered in the utility system. Besides the amount of power, the scenario of the power supply technology is provided (e.g., electricity grid or wind turbine).

Total cooling amount supplied by each utility system. Indicates the total amount of thermal energy for cooling in MJ provided by each cooling supply technology considered

in the utility system. Besides the amount of cooling energy, the considered cooling supply technology scenario (e.g., cooling water) is provided.

Total mass of carbon steel of the equipment. Indicates the total mass of carbon steel required for the equipment construction. For each piece of equipment, the total mass of carbon steel is obtained by the weight of each equipment unit multiplied by the total number of equipment units required in the capture plant. The mass of carbon steel for each piece of equipment is estimated using linear correlations fitted to Aspen Capital Cost Estimator[S72] data depending on the equipment size.

6.4 Output at the LCA layer

The KPIs of the LCA model are defined according to the life cycle impact assessment (LCIA) method Environmental Footprint 3.0 recommended by the European Commission's Joint Research Centre[S97,S98]. The Environmental Footprint 3.0 LCIA method comprises 28 impact categories (16 main categories and 12 subcategories) that can be clustered into environmental impacts on climate change, resources, ecosystem quality, and human health. For each impact category, the environmental impact h_i can be calculated as:

$$h_i = \sum_{i \in D} c_i \cdot x_i \qquad \text{(S69)}$$

where c_i is the LCIA score taken from LCA databases, x_i is the life cycle inventory of process i, and D is the set of all processes within the system boundaries. All impact categories are briefly described in the following subsections. For details, the reader is referred to the Environmental Footprint 3.0 documentation.[S97]

6.4.1 Climate Change

- **Climate Change:** Indicates the total Global Warming Potential (GWP) due to greenhouse gas emissions to the air and CO_2 uptake from the atmosphere according to the IPCC, 2013.[S200] A GWP baseline of 100 a (GWP100) is considered. (Unit: kg CO_2-Eq)
  - **Climate Change: Biogenic:** Indicates the GWP due to greenhouse gas emissions from biomass utilization (e.g., combustion) and CO_2 uptake from the atmosphere due to biomass growth.
  - **Climate Change: Fossil:** Indicates the GWP due to greenhouse gas emissions from fossil fuel utilization (e.g., combustion).
  - **Climate Change: Land Use and Land Use Change:** Indicates the GWP (CO_2 uptake and emission) due to changes of the carbon stock caused by land use change and land use (e.g., deforestation or road construction).

6.4.2 Resources

- **Water Use:** Indicates the potential of water deprivation to ecosystems or humans in a specific region. The impact category considers the relative available water that remains after supplying the ecosystem and human water demand. (Unit: m^3 world eq. deprived)
- **Energy Resources: Non-Renewable:** Indicates the use of non-renewable fossil resources (e.g., natural gas or coal). The use of fossil resources is measured using the net calorific value. (Unit: MJ, net calorific value)
- **Material Resources: Metals/Minerals:** Indicates the use of non-renewable non-fossil natural resources (e.g., minerals and metals). This environmental impact category takes into account the availability of a mineral or metal on earth and the current mining rate. The use of natural resources like minerals and metals is measured using antimony (Sb) as reference material. (Unit: kgSb-Eq)

- **Land Use:** Indicates the use of the land area (e.g., by agriculture, housing, or mining) depending on the soil quality and the effect on land properties and area changes. (Unit: dimensionless)

6.4.3 Ecosystem Quality

- **Acidification:** Indicates the potential acidification of soils and water due to hydrogen ions (H+) that are released to the environment due to mineralization of substances like NOx or SOx. (Unit: mol H^+-Eq)

- **Ecotoxicity: Freshwater:** Indicates the toxic impact of released substances on a freshwater ecosystem and its organisms, structure, and function. The impact is measured using the comparative toxic unit for ecosystems (CTUe).

  - **Ecotoxicity: Freshwater, Inorganics:** Indicates the toxic impact of inorganic substances on a freshwater ecosystem.

  - **Ecotoxicity: Freshwater, Metals:** Indicates the toxic impact of metals on a freshwater ecosystem.

  - **Ecotoxicity: Freshwater, Organics:** Indicates the toxic impact of organic substances on a freshwater ecosystem.

- **Eutrophication: Freshwater:** Indicates the increase of nutritional elements in a freshwater ecosystem. This increase can result from the emission of substances that contain phosphor. (Unit: kg PO_4-Eq)

- **Eutrophication: Marine:** Indicates the increase of nutritional elements in a marine ecosystem. This increase can result from the emission of substances that contain nitrogen. (Unit: kg N-Eq)

- **Eutrophication: Terrestrial:** Indicates the increase of nutritional elements in a terrestrial ecosystem. This increase can result from the emission of substances that contain nitrogen. (Unit: mol N-Eq)

6.4.4 Human Health

- **Human Toxicity: Carcinogenic:** Indicates the effects of an intake of toxic substances on human health related to cancer. The intake of toxic substances can result, e.g., from air, food, or water intake. (Unit: CTUh (Comparative Toxic Unit for human) that indicates the disease cases in the human population per kg emitted)
  - **Human Toxicity: Carcinogenic, Inorganics:** Indicates the effects of an intake of toxic inorganic substances on human health that are related to cancer.
  - **Human Toxicity: Carcinogenic, Metals:** Indicates the effects of an intake of toxic metals on human health, which are related to cancer.
  - **Human Toxicity: Carcinogenic, Organics:** Indicates the effects of an intake of toxic organic substances on human health, which are related to cancer.
- **Human Toxicity: Non-Carcinogenic:** Indicates the effects of an intake of toxic substances on human health unrelated to cancer and not caused by particulate matter or ionizing radiation. The intake of toxic substances can result, e.g., from air, food, or water intake. (Unit: CTUh (Comparative Toxic Unit for human) that indicates the disease cases in the human population per kg emitted)
  - **Human Toxicity: Non-Carcinogenic, Inorganics:** Indicates the effects of an intake of toxic inorganic substances on human health that are not related to cancer.
  - **Human Toxicity: Non-Carcinogenic, Metals:** Indicates the effects of an intake of toxic metals on human health unrelated to cancer.

  - **Human Toxicity: Non-Carcinogenic, Organics:** Indicates the effects of an intake of toxic organic substances on human health unrelated to cancer.

- **Particulate Matter Formation:** Indicates the effects of particulate matter (PM) emissions and its precursors (e.g., NOx or SOx) on human health and potential disease. (Unit: disease incidence)

- **Ozone Depletion:** Indicates the stratospheric ozone degradation due to emissions of ozone-depleting molecules like chlorofluorocarbons. Trichlorofluoromethane (CFC-11) is used as a reference molecule. (Unit: kg CFC-11-Eq)

- **Photochemical Ozone Formation: Human Health:** Indicates the effect of the formation of the ozone at the troposphere level on human health due to photochemical oxidation. The impact is measured by the increase of the tropospheric ozone concentration in kg NMVOC-Eq (non-methane volatile organic compound).

- **Ionising Radiation: Human Health:** Indicates the effects of radioactive releases (e.g., radionuclide emissions) on human health. Uranium-235 (U235) is used as reference material. (Unit: kBq U235-Eq)

7 Spearman analysis

In our platform, we compute 50 KPIs. Some of these KPIs are important for a specific application. With so many KPIs, observing specific trends across all platform layers for a particular case study is challenging. Also, many KPIs are correlated, so for this purpose, we analyze the Spearman's rank-order correlation coefficients[S201] r_S between KPIs as a first-pass approach to find the strength and direction of association between two ranked KPIs.

The definition of the Spearman correlation coefficient is:

$$r_\mathrm{S} = \frac{cov(R(X)R(Y))}{\sigma_{R(X)}\sigma_{R(Y)}}, \tag{S70}$$

where $R(X)$ denotes the rank of the variable X, $cov(R(X)R(Y))$ is the covariance of the rank for variables X and Y, and $\sigma_{R(X)}$ and $\sigma_{R(Y)}$ denote the standard deviation of the variables X and Y, respectively. The Spearman correlation assesses the monotonicity of the relationship between two variables where a r_S of $+1$ indicates a perfectly monotone increasing relationship between X and Y, while a r_S of -1 indicates a perfectly monotone decreasing relationship.

Note that a well-defined but non-monotone relationship can generate a low/near-zero r_S. For example, the water-related variables (i.e., α, the water penetration length into the column, and WRC) have complex interrelationships with other KPIs that are not readily apparent through this metric. For example, if a material has a low α, this material can have good performance irrespective of the WRC. If a material has a high WRC, the value of α is irrelevant. For these reasons, we assess the water-related impact on the process in a separate section (see Section 9.1). Figure S28 presents the resulting Spearman's rank correlation matrix for the cement case study in the UK with a TVSA process at 0.6 bar.

A minimum Spearman's rank correlation coefficient of $r_\mathrm{S} = 0.28$ is obtained, and a maximum of almost $r_\mathrm{S} = 1$. Thus, all KPI rankings have a positive correlation and range from a weak up to a nearly perfect rank correlation.

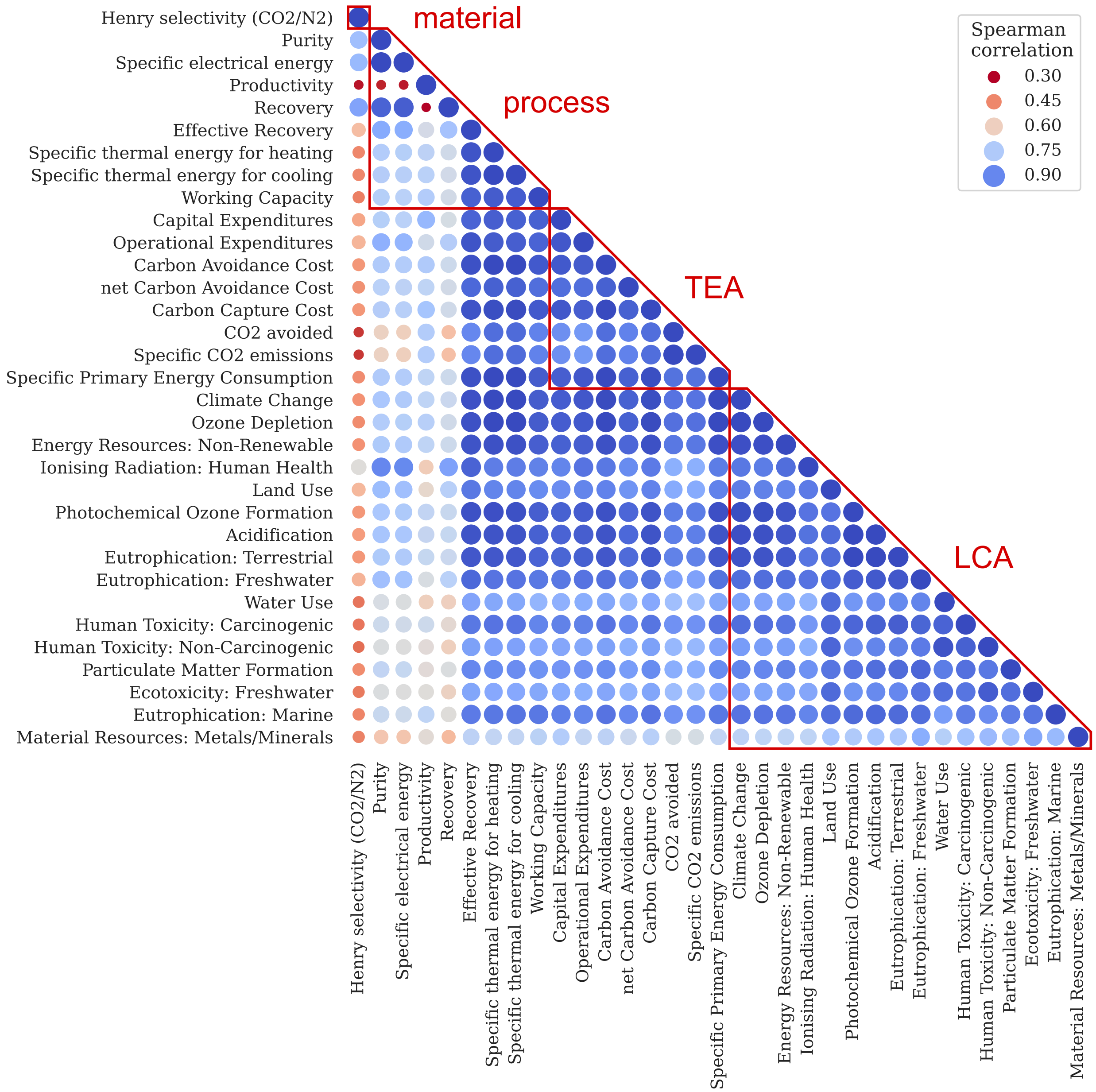

Figure S28| Spearman's rank correlation matrix for the cement case in the UK with TVSA process at 0.6 bar**.** The Spearman's rank correlation matrix of the rankings considers one material KPI, eight process KPIs, eight TEA KPIs, and 16 LCA KPIs. *A dark blue color represents very strong correlations, while dark red represents lower correlations. The size of the circle is proportional to the absolute value of the correlation. The diagonal circles in the matrix have a Spearman's (self) correlation coefficient of 1.*

Materials Layer In many studies, the properties of the materials are used as proxies for typical process KPIs. As our platform directly computes the relevant process KPIs, such proxies are unnecessary in this analysis. It is interesting, however, to illustrate the limitation of a materials' KPI that is ignorant of the process details. For this purpose, we keep the Henry selectivity (S) as an example of such a proxy.

Process Layer We include the eight process KPIs for Spearman analysis. For example, a strong correlation between the purity and specific electrical energy demand is observed. No strong correlation is observed between productivity and the other KPIs in the process layer. The remaining process KPIs (e.g., effective recovery and working capacity) correlate with the TEA-KPIs. Hence, we select purity (Pu) and productivity (P) as reference KPIs, and no additional KPIs are selected from the group that correlates with the TEA-KPIs.

TEA Layer For the TEA layer, all KPIs are strongly correlated; thus, we select only the Net Carbon Avoidance Cost (nCAC). The nCAC combines capital and operational costs and considers the total life cycle CO2-eq. emissions of the capture plant, i.e., not solely the energy-related emissions but also those from material synthesis and disposal and plant construction.

LCA Layer For the LCA layer, we focus the analysis on the 16 main LCA impact categories (cf., Section Output of the PrISMa platform). In this layer, a high correlation is observed for most KPIs except for the KPI Material Resources: Metals/Minerals (MR:MM). Therefore, we select Climate Change (CC) due to its importance for carbon capture applications and the KPI MR:MM. The LCA KPI MR:MM considers the availability of a particular metal on earth and the current mining rate. This KPI captures not only the environmental impact of the metal in the MOF but also the metals and minerals required for, e.g., the energy supply or plant construction.

8 The stakeholders' perspectives

The design and development of a carbon capture plant require a holistic approach, where the perspectives of many stakeholders, from those in the technosphere to those in the ecosphere, must be considered. The stakeholders' perspectives incorporate a variety of requirements and research questions on the plant's development. So far, such a holistic approach has been lacking within the field of carbon capture, with only some examples of design attempts that cross over the materials and process design space.[S202–S204] The stakeholders' perspectives and examples of the corresponding requirements and research questions we take are summarized as follows:

- *The engineer's perspective:* For a given CO_2 source and sink, many technology options exist to capture CO_2. From an engineer's perspective, it is crucial to identify and design the best technology option for a given separation. Adsorption-based processes use temperature and/or pressure swings to separate the component of interest from the gas mixture. An adsorption cycle consists of several steps to achieve the required process specifications, e.g., purity and recovery, and deliver a continuous plant operation. For example, the CO_2 separation from a very diluted gas stream may require adding a vacuum step and/or several intermediate heating steps to meet the purity specifications of the process. The engineer's perspective provides the best technological option for the process and considers the development of a feasible and optimal solution when scaling up the amount of CO_2 captured. This perspective also requires knowledge of available utilities at the host CO_2 emitter and its optimal integration within the capture plant.

- *The environmental manager's perspective:* The perspective of the environmental manager takes into account the environmental impacts of the capture plant. As capturing CO_2 inherently leads to CO_2-eq. emissions, e.g., due to energy supply or material synthesis, the environmental manager aims to maximize the captured CO_2 while simultaneously minimizing the associated CO_2-eq. emissions. Moreover, the environmental

manager's perspective considers environmental impacts beyond climate change to ensure an overall environmentally benign solution.

- *The CO_2 producer's perspective:* The required decarbonization of our energy and industrial systems will imply the mitigation of CO_2 emissions from many sources. These CO_2 emitters define the input conditions to the capture plant, e.g., flue gas composition, flow rate, temperature, and pressure. For a specific CO_2 source, the CO_2 producer aims to find the most cost-efficient way to reduce CO_2 emissions. For example, the available utilities in the host plant can be integrated into the capture process, and the platform can guide the decision on if such an integration reduces costs.

- *The CO_2 buyer's perspective:* The CO_2 buyer defines the requirements on the captured CO_2. From the CO_2 buyer perspective, the developed carbon capture plant has to provide a CO_2 stream that fulfills specific requirements for use or storage, e.g., the purity of the CO_2 or pressure.

- *The investor's perspective:* The investor aims to find the most cost-efficient solution for a carbon capture plant with an optimal trade-off between the capital expenditure and operating cost. From the investor's perspective, the most cost-efficient solution has to be economically competitive with current state-of-the-art carbon capture plants, e.g., absorption-based carbon capture using an amine solvent. The global nature of CO_2 emissions reduction will require the deployment of carbon capture plants in many parts of the world. Due to the region-dependent cost of building and operating a plant, the investor aims to find the most cost-efficient region to invest in a carbon capture plant. The PrISMa platform will give the investor the break-even carbon price for a given CO_2 source and sink in a particular part of the world.

- *The chemist's perspective:* The adsorbent material used in the carbon capture plant is at the heart of the process. For decades, efforts have been devoted to identifying which materials would be "best" for carbon capture applications. The route from the

first synthesis of a new material to its implementation into a commercial process can take many years. It is, therefore, important to provide some guidance from a chemist's perspective on impactful innovation material targets. For example, a chemist has synthesized a novel MOF and uses the PrISMa platform to see if this new material is interesting for carbon capture. Or vice versa, the platform will provide insights into the molecular features that define an optimal material for a given application. This may further inspire chemists to synthesize alternative materials with similar features.

One of the most important applications and benefits of our PrISMa platform is that we can holistically consider all the requirements from the many stakeholders at the early design phase of a carbon capture plant.

In this section, we illustrate the use of the platform for some of our 66 Case Studies, taking these stakeholders' perspectives.

8.1 The engineer's perspective

In the platform, we have implemented two technologies Temperature Swing Adsorption (TSA), and Temperature Vacuum Swing Adsorption (TVSA), for which we consider two levels of vacuum pressure, 0.2 bar and 0.6 bar.

In Figure S29, we compare the nCAC for the top-performing materials for each of the three CO_2 sources and process configurations. For the NGCC power plant, the TVSA process with the lowest vacuum pressure (0.2 bar) gives the lowest nCAC. For coal and cement, we see a similar nCAC for TSA and TVSA with some vacuum (0.6 bar). Our sink is geological storage, which requires a purity of the $CO_2 > 96\%$. Figure S30 gives the purity that is reached for these materials. We see that adding some vacuum greatly improves the purity. For example, if we use TSA for cement, only four materials reach the desired purity, while for TVSA (0.6 bar), most (17 out of 20) reach the desired purity. A similar conclusion holds for coal. After optimization of the process parameters, more materials will reach the required purity (see Section 10).

After the adsorption step, the vacuum rapidly purges the weakly adsorbed components, increasing the purity. Vacuum is most effective if the CO_2 concentration is low. For instance, increasing the vacuum level reduces the residual uptake after the regeneration step, which, in turn, increases the working capacity of the material. As the working capacity is a good proxy of the amount of adsorbent needed, and hence the number of required columns for the separation, the effect of using vacuum is reflected in the CAPEX. However, including a vacuum introduces additional capital and variable operating costs, such as electricity consumption, which may outweigh these cost savings.

Where the feed concentration of CO_2 is low, e.g., for NGCC power plants, the TVSA process with the lowest vacuum pressure (0.2 bar) gives the lowest nCAC. The loss in working capacity by reducing the vacuum level from 0.2 bar to 0.6 bar leads to notable increases in CAPEX, being even more pronounced for TSA. Besides the increase in CAPEX for NGCC power plants, the variable OPEX also increases for decreasing vacuum level, which is mainly caused by the increase in total energy demand. This effect gets amplified for TSA because of the additional drop in the working capacity and increased specific thermal energy demand. For coal and cement, the lowest nCAC is obtained with TVSA process with intermediate vacuum levels, e.g., (0.6 bar) vacuum pressure.

Simple thermodynamic arguments indicate that the higher the CO_2 concentration, the lower the minimal amount of work needed for the separation. However, Figure 4(a) shows a higher nCAC for cement plants (20 % CO_2) than for coal power plants (15 % CO_2). Our calculations show that the cement plant's total energy requirements and capital costs are smaller. However, in a cement plant, none, or very few, of the on-site utilities can be used in the capture process. These utilities have to be either bought externally or built on-site, which is more expensive than for the corresponding coal Case Studies, where the available utilities in the host plant can be efficiently used to satisfy the energy demands of the capture plant. The results highlight that the simple thermodynamic argument holds regarding total

energy demand. Still, the final net costs per CO_2 (avoided) additionally depend strongly on the available utilities and the specifics of the Case Study.

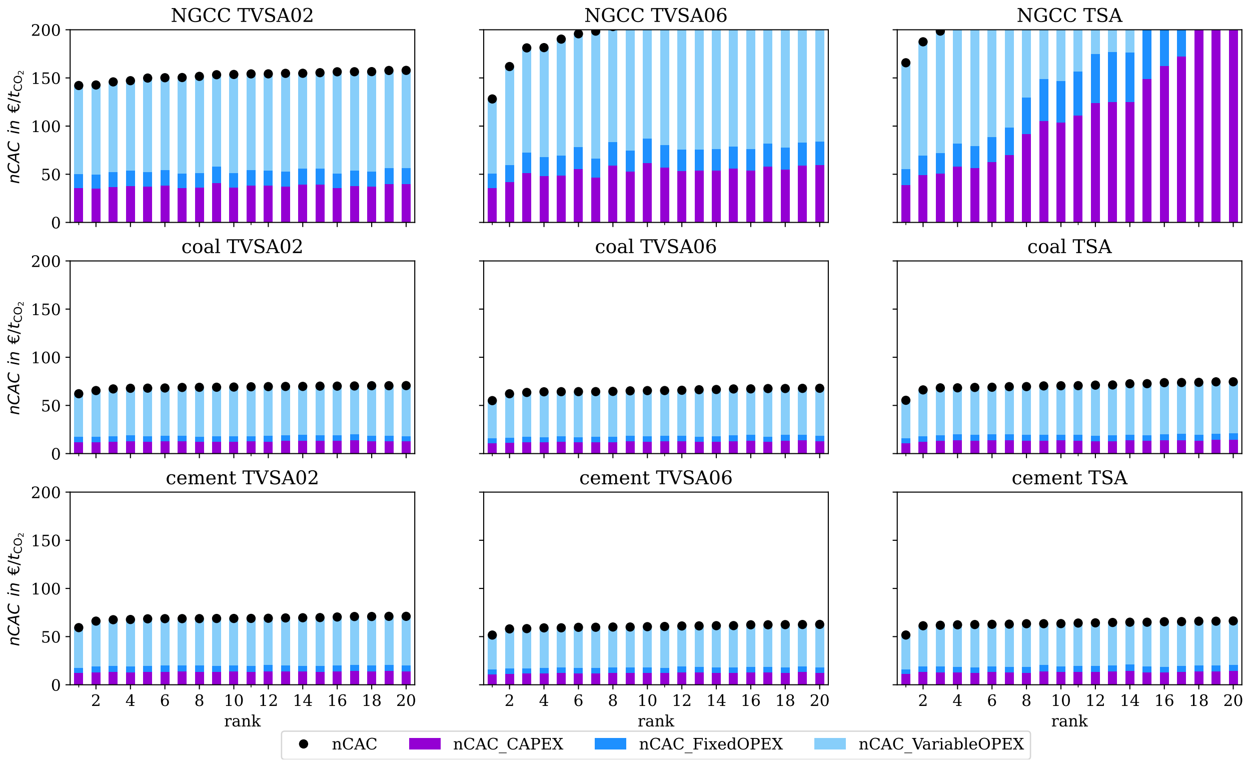

Figure S29| Breakdown of the nCAC of the top 20 materials for (top) NGCC power plants, (middle) coal power plants, and (bottom) cement plants in the UK, for the technologies TVSA with a vacuum pressure of 0.2 bar (left) and 0.6 bar (middle) and TSA (right). The costs are grouped into CAPEX (purple), fixed OPEX (dark blue), which is a percentage of the CAPEX, and variable OPEX (light blue), which mainly involves the cost related to the energy requirements of the process. The black dots are the total nCAC values.

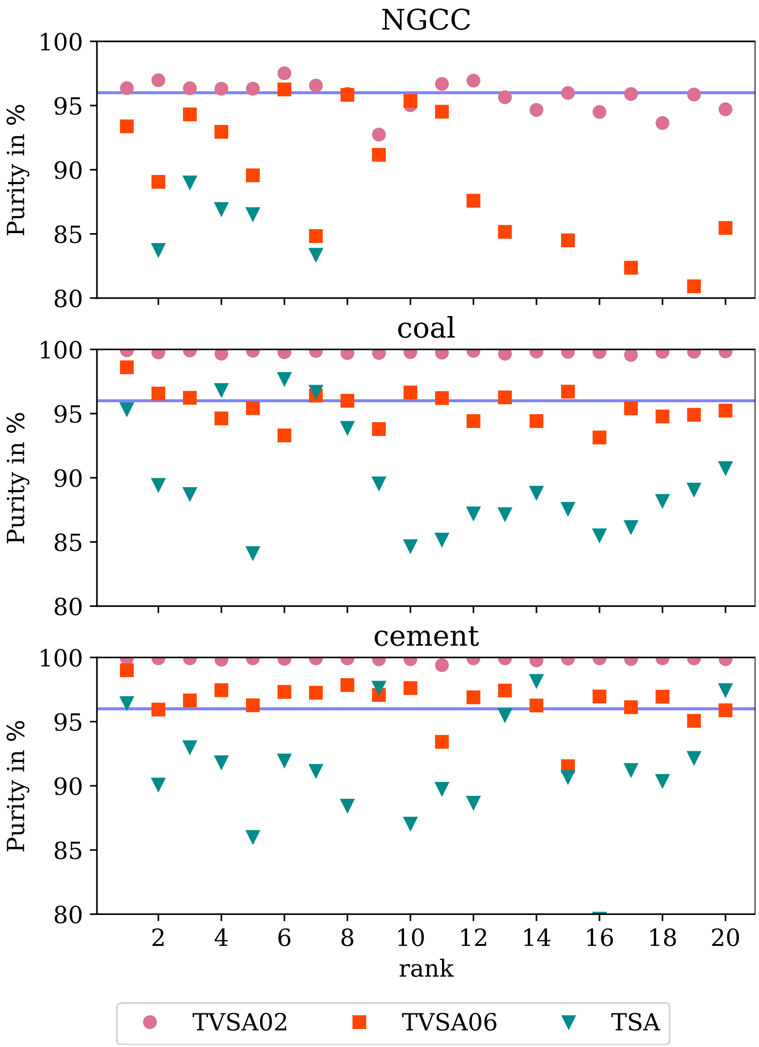

Figure S30| The purity of the top 20 materials for (top) NGCC power plants, (middle) coal power plants, and (bottom) cement plants in the UK, for the different technologies TVSA with a vacuum pressure of 0.2 bar, TVSA with a vacuum pressure of 0.6 bar, and TSA. The blue line is the required purity (96 %) for geological storage. If a point is missing for a material, it means that the purity is less than 80 %

8.2 The environmental manager's perspective

At the LCA layer, 28 environmental impact categories of the Environmental Footprint 3.0 are calculated (cf. Section 6.4).

8.2.1 Climate Change

One of the most important LCA KPIs for carbon capture processes is the climate change impact CC. The climate change impact measures the total CO_2-eq. emissions of the capture process in kg CO_2-eq./kgCO_2-captured (in the following indicated in %) associated with the synthesis and disposal of the adsorbent, the external utility supply, the compensation for the parasitic load for power plant cases, and the plant construction and disposal. To assess the effective amount of CO_2 avoided in the atmosphere, we compute the effective recovery R^* of the carbon capture process. The effective recovery R^* is calculated as the ratio between the avoided CO_2-eq. emissions and the total amount of CO_2 in the flue/industrial gas and can be expressed as:

$$R^* = R \cdot (1 - \mathrm{CC}), \tag{S71}$$

where R denotes the recovery of the process in % (see Section 6.2.2), which is the percentage of the captured CO_2 from the total amount of CO_2 in the flue/industrial gas, and CC the climate change impact in kg CO_2-eq./kg CO_2-captured.

In Figure S31, we compare the climate change impact CC with the recovery and effective recovery of the capture process for the top 20 materials with the lowest nCAC in the UK.

If we only consider the process, we see that for all three CO_2 sources, the recovery is close to 100 % for the TSA processes. In contrast, for TVSA with a vacuum pressure of 0.2 bar, the average recovery decreases for increasing CO_2 concentration of the CO_2 source. The vacuum step increases the purity (see Figure S30) but at the expense of the recovery, and the higher the CO_2 concentration in the feed, the larger this effect.

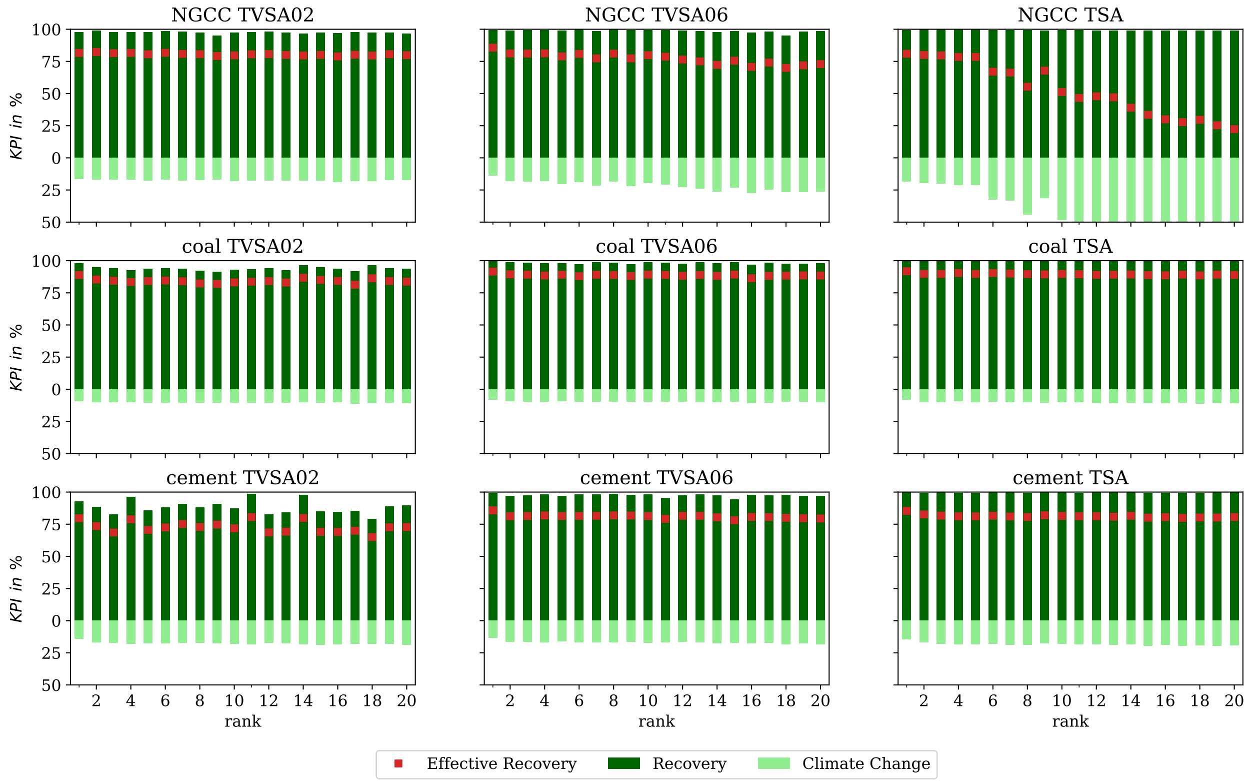

Figure S31| Recovery, effective recovery, and Climate Change (CC) for the top 20 materials (ranked according to the nCAC) for (top) NGCC power plant, (middle) coal power plant, and (bottom) cement plant in the UK, for the TVSA technology with a vacuum pressure of 0.2 bar (left) and 0.6 bar (middle) and TSA (right). The recovery is given by the dark green bar, the CC (in %) by the light green bar, and the red dots give the effective recovery.

We need to correct the recovery from the process for the indirect CO_2 emissions. For the power generation Case Studies (coal and NGCC), the major contributor to climate change (CC) results from the compensation for the loss of electricity production (i.e., the parasitic load) by the average electricity grid (see Section 3.4). As the concentration of CO_2 for the NGCC power plant is lower than for the coal power plant, the specific energy requirements are higher for the NGCC case. As a result, the climate change impact per captured kg of CO_2 is about 18 % for the NGCC case and about 10 % for the coal case (TVSA with a vacuum pressure of 0.2 bar). For NGCC with TSA, the number of columns and energy requirements strongly increase (see Section 8.1), leading to climate change impacts up to 77 % for the top 20 materials (median = 50 %).

For the cement plant, the climate change impact per captured kg of CO_2 is about 18 %, independent of the technology we use. If we focus on the TSA process for the cement and coal case, we see that despite the lower specific energy demand due to the higher CO_2 concentration for cement, the climate change impact is higher than that for coal. For cement, the major contribution to the CC results from the additional external utilities, i.e., the heat demand supplied by natural gas boilers and the electricity demand supplied by the electricity grid. It is assumed that the CO_2 emitted by the external gas boilers is not captured. This point is discussed in more detail in Section 8.2.2. The CO_2-eq. emissions per amount of heat are generally higher for natural gas boilers than for steam extraction and compensation of the parasitic load by the average grid mix in the UK. These emissions are only slightly region-dependent, and the much smaller region-dependence of the CC for cement mainly results from the electricity demand (see Figure 4(c) in the main manuscript). For the cement plant with TVSA and a vacuum pressure of 0.2 bar, we see a larger variation in effective recovery due to the variation in the recovery of up to 15 % for the same nCAC.

The effective recovery depends on the region of the world. In Figure S32, S33, S34, and S35 we show the effective recovery, recovery, and CC for the US, China (Guangdong and Shandong provinces), and Switzerland, respectively. The recovery is independent of the

plant's location. In contrast, the CC and thus also the effective recovery depends on the region, and we see large differences between the regions.

For the NGCC case, we focus on TVSA with 0.2 bar vacuum pressure. We get a near 100 % recovery for all materials. For Switzerland, the emissions associated with CC are the lowest, leading to a median effective recovery of 95 %. This is due to the contribution of hydroelectric and nuclear power to the electricity grid.[S205] In contrast, in China, the median effective recovery drops to 59 % and 40 % in the Guangdong and Shandong provinces, respectively. In China, coal accounts for a large fraction of its electricity production, and in the Shandong province, the use of coal is higher compared to Guangdong.[S206] In the US, the energy grid generated more CO_2 than in the UK, and we find a median effective recovery of 70 % and 81 %, respectively.

For coal, we see the impact of the vacuum on recovery for all countries. Using a lower vacuum level (0.6 bar) or TSA gives a near 100 % recovery. Once we subtract the CC from the recovery, we see that the effective recovery reaches in the UK, US, China (Guangdong), and China (Shandong), 89 %, 83 %, 77 %, and 66 %, respectively, which is again a reflection of how CO_2 emissions derived from the grid, are larger in China than in the US and UK.

The cement case gives a different picture. Here, we see that TSA gives a near 100 % recovery, but now the CC shows much smaller differences between the regions compared to the NGCC and coal cases. The reason is that for cement, the most important contribution to the CC are the emissions from the natural gas boilers. The natural gas boilers provide most of the utilities required to operate the capture plant.

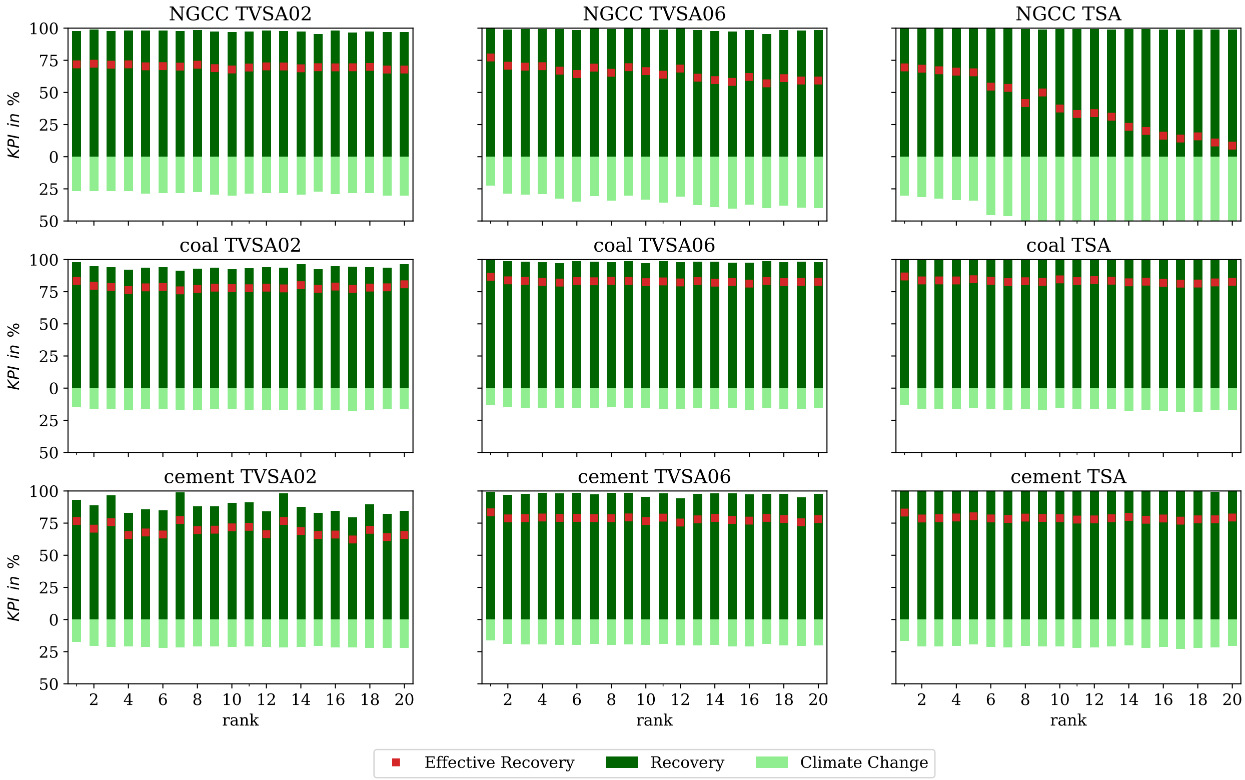

Figure S32| Recovery, effective recovery, and Climate Change (CC) for the top 20 materials (ranked according to the nCAC) for (top) NGCC power plant, (middle) coal power plant, and (bottom) cement plants in the US, for the technologies TVSA with a vacuum pressure of 0.2 bar (left) and 0.6 bar (middle) and TSA (right). The recovery is given by the dark green bar, the CC (in %) by the light green bar, and the red dots give the effective recovery.

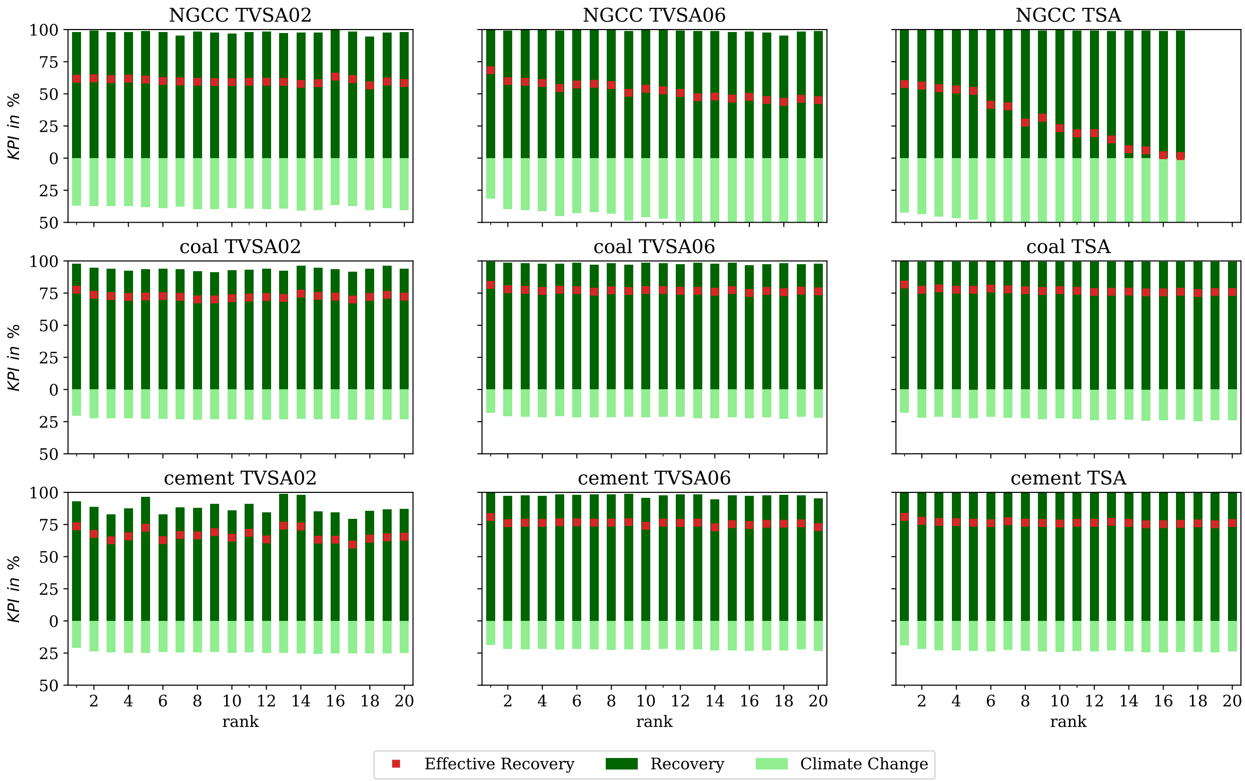

Figure S33| Recovery, effective recovery, and Climate Change (CC) for the top 20 materials (ranked according to the nCAC) for (top) NGCC power plant, (middle) coal power plant, and (bottom) cement plants in China (Guangdong province), for the technologies TVSA with a vacuum pressure of 0.2 bar (left) and 0.6 bar (middle) and TSA (right). The recovery is given by the dark green bar, the CC (in %) by the light green bar, and the red dots give the effective recovery. For TSA, not all materials in the top 20 resulted in an effective recovery above 0; these materials have been omitted.

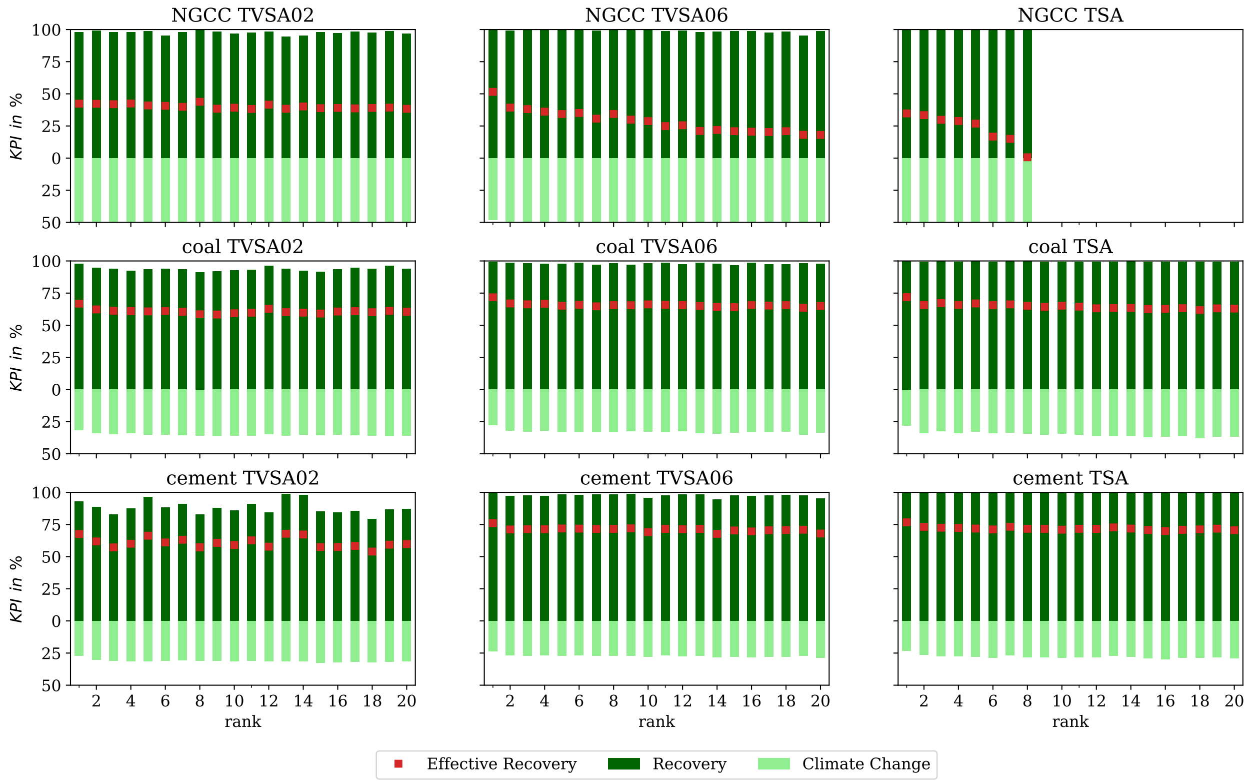

Figure S34| Recovery, effective recovery, and Climate Change (CC) for the top 20 materials (ranked according to the nCAC) for (top) NGCC power plant, (middle) coal power plant, and (bottom) cement plants in China (Shandong province), for the technologies TVSA with a vacuum pressure of 0.2 bar (left) and 0.6 bar (middle) and TSA (right). The recovery is given by the dark green bar, the CC (in %) by the light green bar, and the red dots give the effective recovery. For TSA, not all materials in the top 20 resulted in an effective recovery above 0; these materials have been omitted.

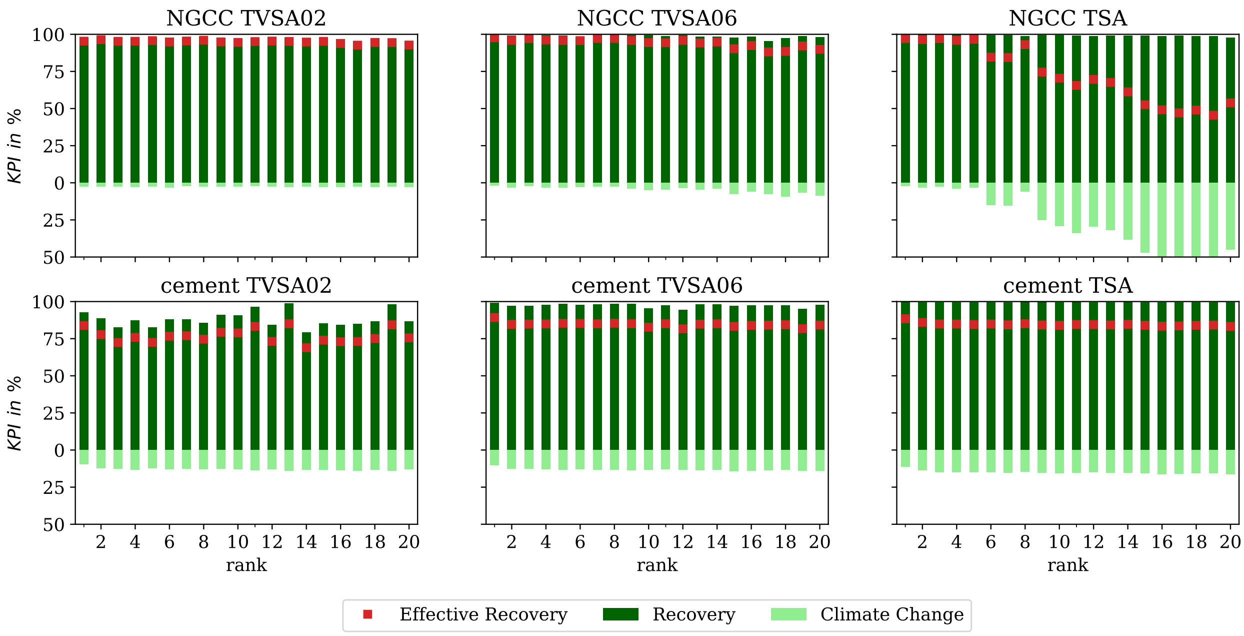

Figure S35| Recovery, effective recovery, and Climate Change (CC) for the top 20 materials (ranked according to the nCAC) for (top) NGCC power plant and (bottom) cement plants in Switzerland, for the technologies TVSA with a vacuum pressure of 0.2 bar (left) and 0.6 bar (middle) and TSA (right). The recovery is given by the dark green bar, the CC (in %) by the light green bar, and the red dots give the effective recovery.

8.2.2 Impact of different utilities

Power plants Heat integration with the existing host plant is the default strategy for power plant producers.

In Figure S36a, we compare those cases where heat extraction from the power plants (NGCC and coal) is not possible (e.g., no access to the Intermediate-Pressure - Low-Pressure crossover in the steam turbines in the case of a retrofit). In those cases, natural gas boilers are considered to provide the required heat for the regeneration process. We observe that the effective recovery decreases for both NGCC and coal since the impact of climate change increases. Climate change increases because we use a natural gas boiler to produce the steam instead of steam extraction from the power plant with compensation of the parasitic load, and this natural gas boiler's emitted CO_2 is not captured. In Figure S36b, we also see that the use of natural gas boilers increases the nCAC because of these increased emissions. Moreover, the operating costs associated with using external utilities are generally more expensive than those when the onsite utilities are used.

Cement plants For cement producers, however, the available utilities at the host plant are very limited; a natural gas boiler has to be built on-site to satisfy heat energy demand. The emissions derived from these gas boilers are the main factor contributing to the indirect emissions from the capture plant. In practice, those emissions would also be abated, and one could mix these with the flue gasses of the cement plant. The external energy demand depends on the (unknown) performance of a material. Hence, the total amount of flue gases will be dependent on the performance of the material, requiring iterative solutions of the equations. Therefore, we have not implemented this solution but use the (worst-case) option of non-abating the CO_2.

From an environmental perspective, the replacement of gas boilers with electrical heaters, e.g., boilers or heat pumps, is an interesting case. For electricity-based heating, an electric boiler's coefficient of performance (i.e., the ratio of useful heating provided to work required)

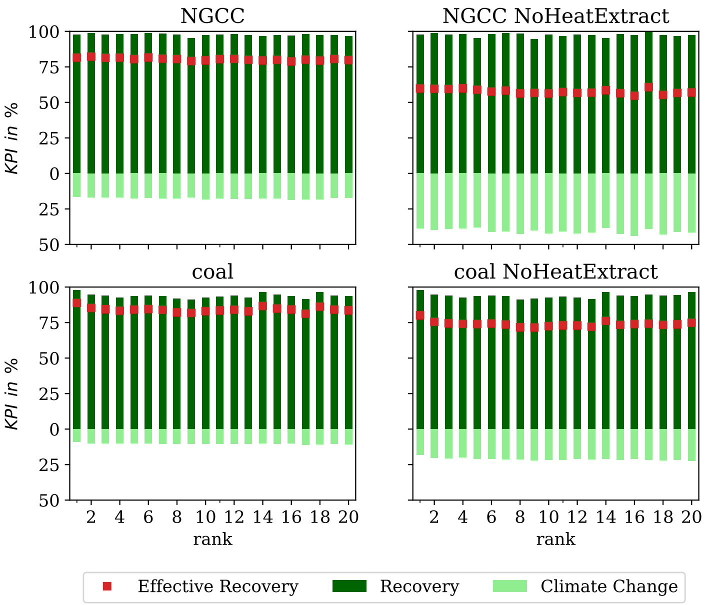

(a) Effective recovery

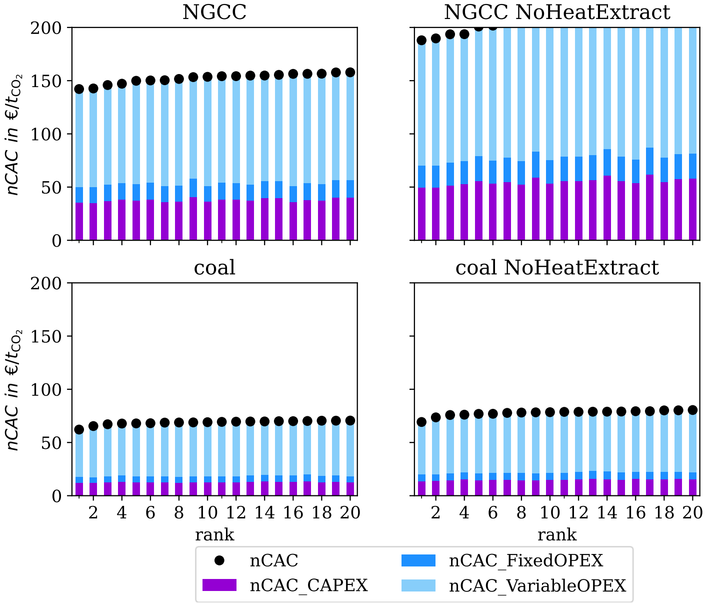

(b) nCAC

Figure S36| (a) Recovery, effective recovery, and Climate Change (CC) and (b) nCAC for the top 20 materials (ranked according to the nCAC) for an NGCC (top) and coal (bottom) plant in the UK with TVSA with heat integration (left) and without heat integration (right). The recovery is given by the dark green bar, the CC (in %) by the light green bar, and the red dots give the effective recovery. For nCAC, the costs are grouped into CAPEX (purple), fixed OPEX (dark blue), which is a percentage of the CAPEX, and the variable OPEX (light blue), which mainly involves the cost related to the energy requirements of the process. The black dots are the total nCAC values.

would be lower than that of a heat pump and thus a heat pump would be preferred. However, from a thoughtful review of the current status of high-temperature heat pumps for industrial applications and careful analysis, we consider that the technology is not ready nor suitable to supply the heat for sorbent regeneration in the cement Case Study because of the following reasons:

1. Industrial heat pumps have limitations regarding their thermal output. The highest thermal energy supply reported in existing literature when heat is extracted from a flue gas stream is approximately 1.5 MWth.[S207] To put this into perspective, in our work, the heat demand for the cement plant Case Study ranges from 48 to 68 MWth for identified top-performing materials. For instance, in the case of CALF20, the heat demand is ca. 88 MWth. Consequently, meeting these heat requirements would necessitate a substantial number of heat pumps.

2. To attain a Coefficient of Performance (COP) exceeding 2, the temperature lift (i.e., the temperature difference between the source and the sink) in a heat pump should be lower than 60 K.[S208] This requirement implies that a heat source at ca. 70 °C is necessary to supply steam at 120 °C. In a cement plant, this heat source is only available in the flue gas stream. However, this is not feasible for our reference cement plant because the heat is already utilized in the raw mill, as outlined below.

3. Incorporating heat pumps would result in a substantial upfront investment cost and increase the operational complexity of the capture plant. Utilizing an intermediate working fluid (i.e., refrigerant) also introduces heat losses/irreversibility due to the pinch points in the subsequent heat exchangers. A more practical alternative would involve the utilization of low-pressure steam coupled with vapor re-compression. However, this alternative is not an option in our cement case, as indicated below.

Our reference cement plant aligns with the Best Available Technology (BAT) cement kiln outlined in the Horizon 2020 CEMCAP project. In this study, an in-depth evaluation of

heat integration options was conducted, and it was ultimately determined that heat recovery can only be achieved from the cooling air stream exiting the clinker cooler. Approximately 8.23 MWth is recovered from the clinker cooler in our reference plant (representing a portion ranging from 12 % to 17 % of the total heat demand for identified top-performing materials). Due to the characteristics of the raw material in the cement plant, i.e., mostly limestone, most of the heat in the flue gases (at ca. 210 °C) at the pre-heater outlet is needed in the raw mill (in interconnected operation). The plant only turns off the raw mill 2 hours/day (in direct operation). The exhaust flue gas leaves the raw mill at ca. 110 °C (130 °C if low air leak is considered in the mill). Therefore, producing steam at 120 °C would not be possible. This may differ from the situation in other cement plants.

A noteworthy heat integration approach is being implemented in the full-scale carbon capture plant currently under construction at the Heidelberg Norcem's Brevik cement plant in Norway.[S209] The carbon capture process incorporates Aker Carbon Capture's proprietary technology, and the heat integration strategy encompasses the recovery of heat from multiple sources, including the clinker cooler, the exhaust flue gases, and the CO_2 compression train. Steam is generated at varying pressures, and a steam compressor is employed to achieve the necessary pressure and temperature levels for sorbent regeneration. Despite this setup, an electric boiler remains essential for start-up and shut-down procedures. One distinctive feature of this approach is the substantial amount of heat recovery from the flue gas. This is made possible by the relatively dry nature of their raw material, primarily composed of limestone, which allows for the recovery of a significant amount of energy from the exhaust flue gases.

We investigate the impact of different types of boilers on the heat supply in a cement plant in Switzerland, as we can reduce the CO_2-eq. emissions by replacing the natural gas boilers with electrical ones. Figure S37a shows that a change from natural gas to electric boilers increases the median effective recovery by 15 %, as the CC is close to zero with electrical boilers due to the low CO_2 intensity of the electricity grid in Switzerland. However,

Figure S37b shows that the higher operating costs of these electric boilers are not fully compensated by the reduced CC, as the median nCAC is about 16 €$t_{CO_2}^{-1}$ more expensive.

8.2.3 Trends in environmental impact categories

Besides climate change, the other environmental impact categories should also be considered for material selection to ensure an environmentally benign solution. In its present implementation, the LCA model for material synthesis focuses on the metals used in the MOFs, as the model assumes that all organic linkers have the same environmental impact.

Impact of the metal used in the MOF Figure S38 shows the 20 top-performing materials in Net Carbon Avoidance Cost (nCAC) across ranks in the 16 main LCA impact categories for (a) the NGCC power plant with TVSA at 0.2 bar vacuum pressure and (b) the cement plant with TVSA at 0.6 bar in the UK. In the simplified LCA model, most top-performing materials rank well in all LCA impact categories. These materials contain environmentally friendly metals, and the ranks are mainly defined by the environmental impacts associated with the energy supply. However, some top-performing materials in nCAC should be avoided due to the used metal. For example, two materials in the top 20 contain Holmium or Gadolinium, leading to a drop in the ranking in Material Resources: Metals/Minerals, Land Use, Ecotoxicity: Freshwater, Eutrophication: Marine, and Human Toxicity: Carcinogenic and Non-Carcinogenic. Materials in the top 20 that contain Cobalt in the metal node have strong outliers in Water Use, Material Resources: Metals/Minerals, Land Use, and Human Toxicity: Carcinogenic. The materials with Praseodymium drop in the ranking for Material Resources: Metals/Minerals, Land Use, Ecotoxicity: Freshwater, and Eutrophication: Marine. One material in the top 20 of the cement case contains silver leading to a drop in 10/16 impact categories. Moreover, materials with rare earth oxides like Samarium or Yttrium drop in the ranking in Eutrophication: Marine due to their production in ion-adsorption clays that have direct ammonium and sulfate emissions to water[S210].

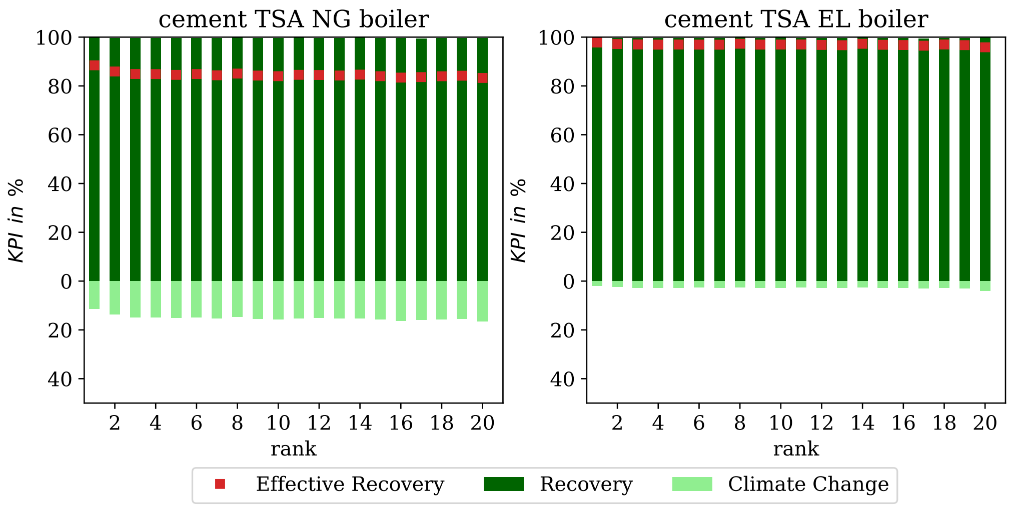

(a) Effective recovery

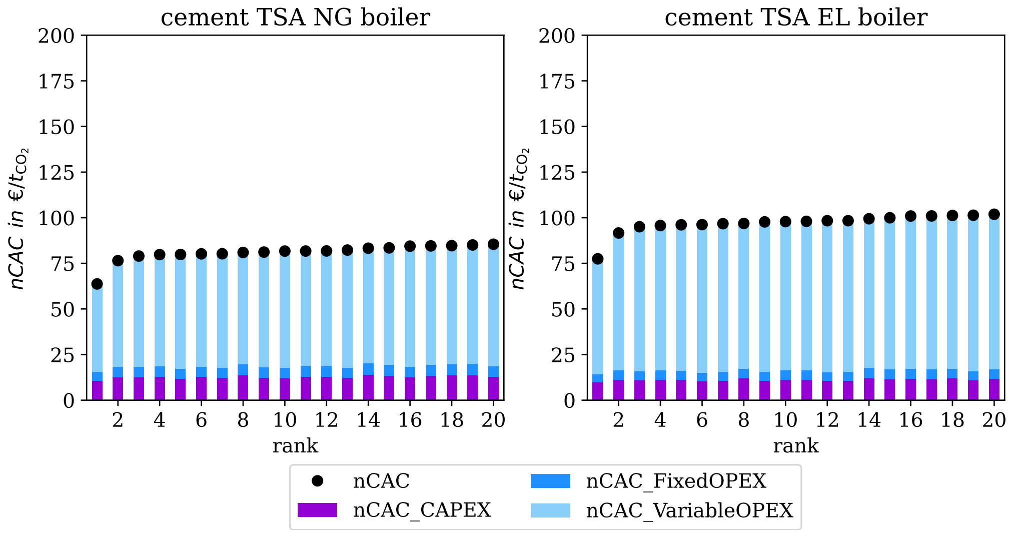

(b) nCAC

Figure S37| (a) Recovery, effective recovery, and Climate Change (CC) and (b) nCAC for the top 20 materials (ranked according to the nCAC) for a cement plant in Switzerland with TSA with natural gas boiler (left) and electrical boilers (right). The recovery is given by the dark green bar, the CC (in %) by the light green bar, and the red dots give the effective recovery. For nCAC, the costs are grouped into CAPEX (purple), fixed OPEX (dark blue), which is a percentage of the CAPEX, and the variable OPEX (light blue), which mainly involves the cost related to the energy requirements of the process. The black dots are the total nCAC values.

Solvent in MOF synthesis We also find some trends in the top-performing materials in nCAC depending on the solvent used for the synthesis. In total, water is selected as the solvent for over 420 materials, ethanol for over 460 materials, and methanol for only 2 materials following the solvent selection procedure described in Section 3.4. For 260 materials, the proxy solvent is selected as the solvent because the phase split between the organic linkers and the solvents could not be calculated due to the current limits on SMILES inputs.

Figure S39 shows the 10 top-performing materials in Net Carbon Avoidance Cost (nCAC) for each solvent across ranks in the 16 main LCA impact categories for (a) the NGCC power plant with TVSA at 0.2 bar vacuum pressure and (b) the cement plant with TVSA at 0.6 bar in the UK. Since methanol is selected solely for two materials, these are also highlighted. Please note that several effects overlap in this figure: For example, the strong outliers in Figure S39 mainly result from the metals used as metal nodes in the materials. However, we can see some general trends in the selected solvents: water as a green solvent ranks very high for all impact categories and should thus be used as the solvent if possible. In contrast, materials synthesized using ethanol as the solvent tend to rank worse in Water Use, Land Use, Ecotoxicity: Freshwater, and Human Toxicity: Carcinogenic and Non-Carcinogenic. For the materials that are synthesized using the proxy solvent (average of all five solvents), the ranks tend to drop in almost all impact categories due to organic solvents like DMF, DMA, or ethanol.

This analysis highlights the impact of the metal and solvent used for the material synthesis on the environmental impacts of the entire capture plant. This impact becomes particularly important for producing the materials on an industrial scale. Our PrISMa platform allows us to identify materials that outperform the benchmark in terms of nCAC and to pinpoint the anticipated environmental hotspots derived from the synthesis. Chemists can use these findings to focus on materials with similar characteristics but look for alternative metals or solvents.

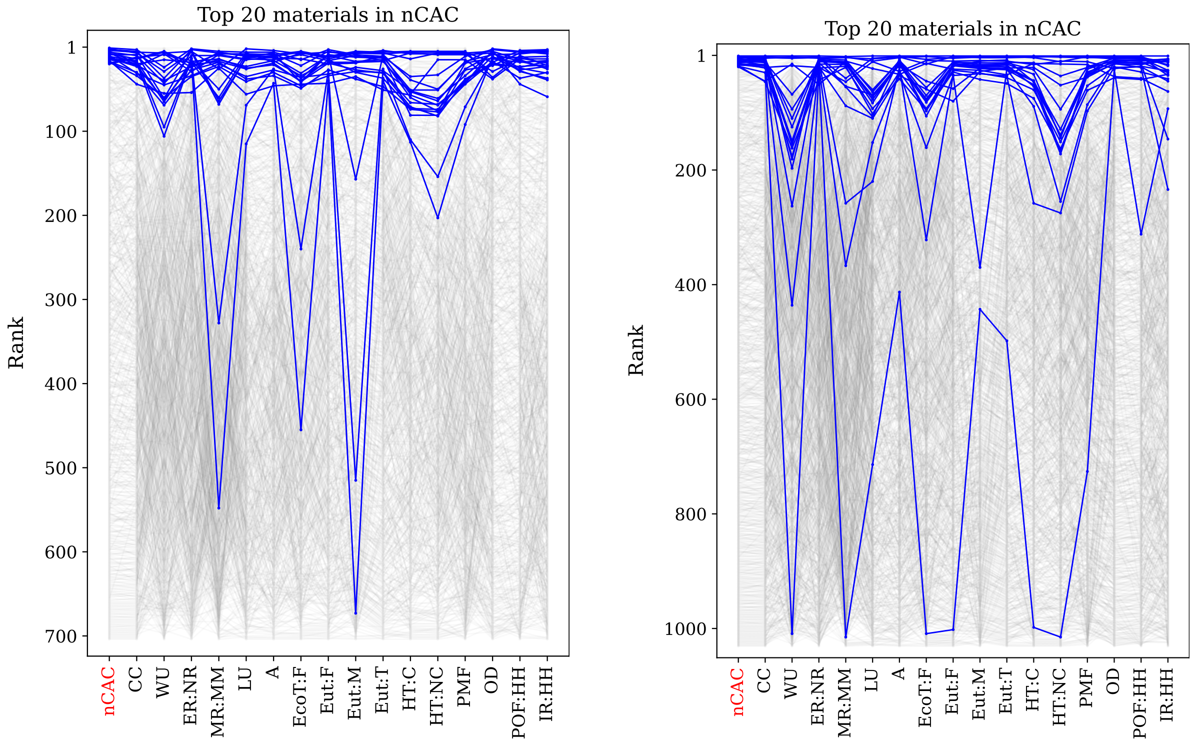

(a) NGCC power plant with carbon capture in the UK (TVSA at 0.2 bar)

(b) Cement plant with carbon capture in the UK (TVSA at 0.6 bar)

Figure S38| Material ranking for all 16 main LCA KPIs: Climate Change (CC), Water Use (WU), Energy Resources: Non-Renewable (ER:NR), Material Resources: Metals/Minerals (MR:MM), Land Use (LU), Acidification (A), Ecotoxicity: Freshwater (EcoT:F), Eutrophication: Freshwater (Eut:F), Eutrophication: Marine (Eut:M), Eutrophication: Terrestrial (Eut:T), Human Toxicity: Carcinogenic (HT:C), Human Toxicity: Non-Carcinogenic (HT:NC), Particulate Matter Formation (PMF), Ozone Depletion (OD), Photochemical Ozone Formation: Human Health (POF:HH), and Ionising Radiation: Human Health (IR:HH). The colored lines show the top 20 materials for nCAC shown in the first column (red).

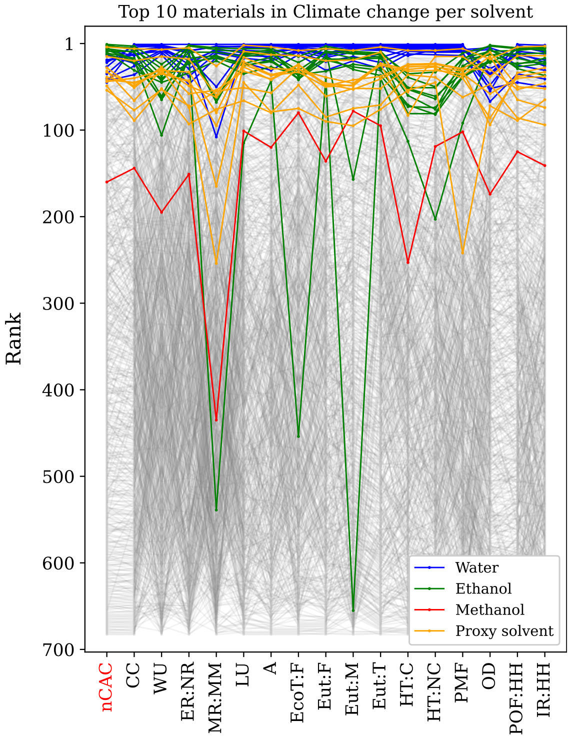

(a) NGCC power plant with carbon capture in the UK (TVSA at 0.2 bar)

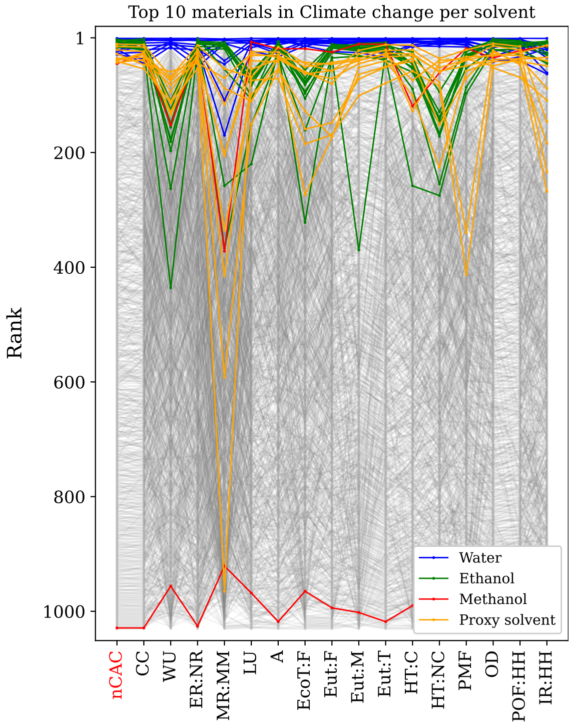

(b) Cement plant with carbon capture in the UK (TVSA at 0.6 bar)

Figure S39| Material ranking for all 16 main LCA KPIs with solvents highlighted: Climate Change (CC), Water Use (WU), Energy Resources: Non-Renewable (ER:NR), Material Resources: Metals/Minerals (MR:MM), Land Use (LU), Acidification (A), Ecotoxicity: Freshwater (EcoT:F), Eutrophication: Freshwater (Eut:F), Eutrophication: Marine (Eut:M), Eutrophication: Terrestrial (Eut:T), Human Toxicity: Carcinogenic (HT:C), Human Toxicity: Non-Carcinogenic (HT:NC), Particulate Matter Formation (PMF), Ozone Depletion (OD), Photochemical Ozone Formation: Human Health (POF:HH), and Ionising Radiation: Human Health (IR:HH). The colored lines show the top 10 materials for nCAC of each solvent (if available) shown in the first column (red).

Materials with Climate Change larger than 1 In Extended Data Figure 2(a), we have indicated those materials for which the total CO_2-eq. emissions of the capture plant are larger than the amount of CO_2 that is captured (cement in the UK with TVSA process at 0.6 bar). Some of these materials rank high for other KPIs, yet using them in a capture plant would have a negative effect on CO_2 emissions. It is interesting to discuss some examples of materials that have such a large impact (CC > 1) on Climate Change.

For instance, in Extended Data Figure 2(a), a group of materials that rank top or very high in CO_2/N_2 Henry selectivity presents a CC > 1. A high selectivity does not guarantee a high CO_2 working capacity. Indeed, this is the case for some of the materials, where their CO_2 working capacity is so low ($< 0.01\,\mathrm{mol\,m^{-3}}$) that a very small amount of CO_2 per volume of bed is captured per process cycle, i.e., the materials show very low productivity values. As a result, the number of columns required to process the feed gas stream is large, and in turn, the energy demand for regeneration is also so significant that more CO_2-eq. emissions are generated than captured over the life cycle time of the capture plant.

Other examples of materials with CC > 1 are those containing precious metals like Au and Rh. Two MOFs fall into this category. Even though these materials have comparatively good working capacity values and moderate heat demands, for these materials, the environmental impact of their synthesis is so large that it leads to CC impacts higher than one.

Our visualization tool allows the reader to inspect these materials further.

8.3 The Carbon dioxide producer's perspective

In this section, we compare capturing CO_2 from two power plants, namely NGCC and coal-fired power plants, and from a cement plant. These plants are based in the UK. We use a TVSA-based carbon capture process with a vacuum level of 0.2 bar for the NGCC case and 0.6 bar for the coal and cement case, and the captured CO_2 is compressed for geological storage.

8.3.1 Different carbon capture cost metrics

In the literature, one can find various cost-related KPIs, and in this section, we discuss four of them: Levelised Cost of Capture (LCOC), Carbon Capture Cost (CCC), Carbon Avoidance Cost (CAC), and Net Carbon Avoidance Cost (nCAC).

The LCOC has been introduced by Department for Business, Energy and Industrial Strategy of the UK government (BEIS) to be able to compare the extra costs of building and operating a capture plant per mass of captured CO_2. In the power plant Case Studies, i.e., coal and NGCC, utilities are available at the host plant and can be integrated with the carbon capture plant. However, using these on-site utilities reduces the power output of the power plant, and this so-called parasitic load is accounted for in the CCC, i.e., by an increased LCOE of the power plant with carbon capture. Running a carbon capture plant leads inherently to CO_2-eq. emissions due to, for instance, utility supply or material synthesis, effectively decreasing the amount of CO_2 that is avoided in the atmosphere. Moreover, for power plants, the parasitic load also impacts the specific CO_2 emissions. This correction to the carbon capture costs gives us the CAC if solely the CO_2-eq. emissions of the energy supply are considered. The nCAC considers the total CO_2-eq. emissions of the entire life cycle and additional operating costs and CO_2-eq. of the compensation of the parasitic load from a system's perspective. The nCAC calculation thus requires the climate change impact of the entire life cycle and thus integration of TEA and LCA, which our PrISMa platform

enables. The available utilities at the host plant are limited for the cement case. Hence, additional utilities must be built on-site or bought/imported externally.

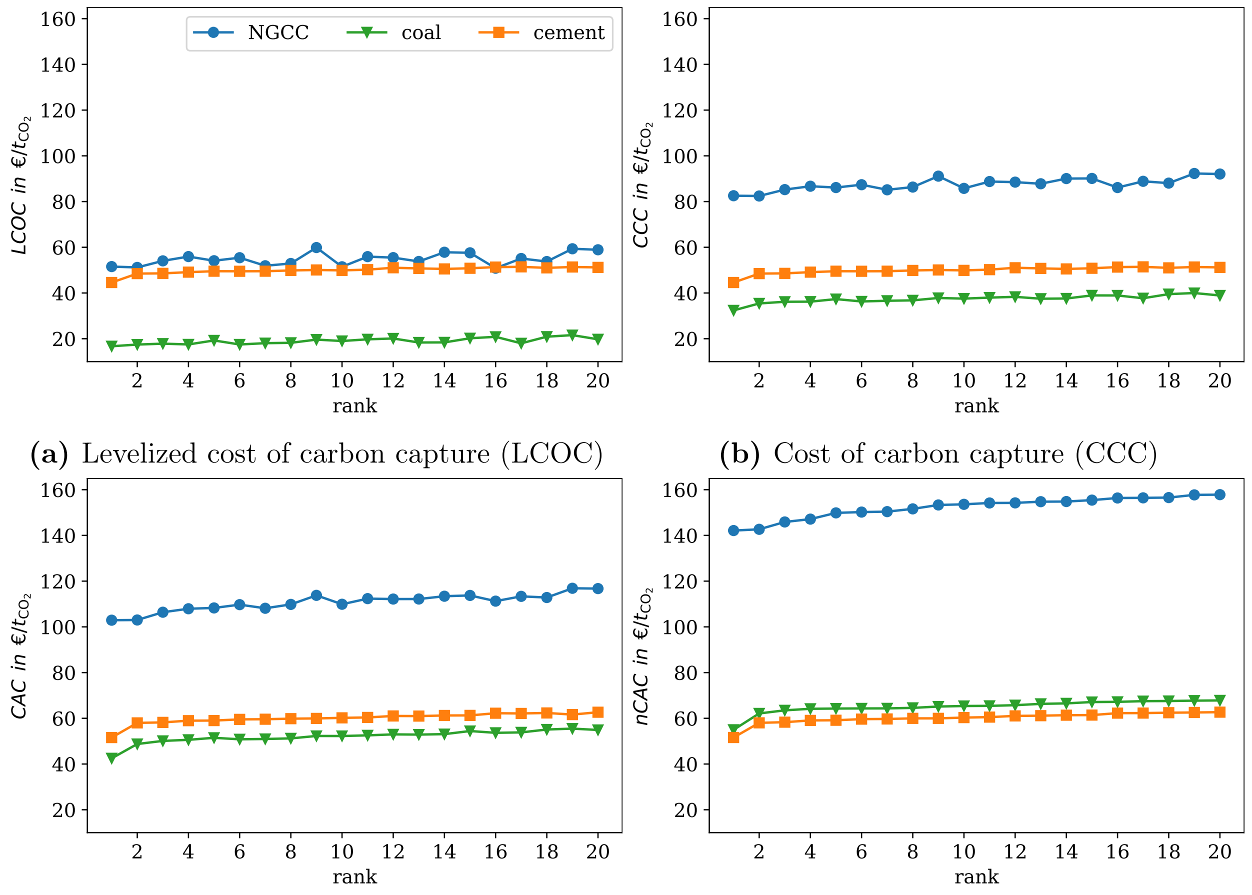

(a) Levelized cost of carbon capture (LCOC) **(b)** Cost of carbon capture (CCC)

(c) Carbon avoidance costs (CAC) **(d)** Net carbon avoidance costs (nCAC)

Figure S40| Top 20 materials in terms of four economic KPIs (LCOC, CCC, CAC and nCAC) for a TVSA carbon capture process at 0.2 bar from an NGCC power plant (blue) and a TVSA carbon capture process at 0.6 bar from coal power plant (green) and cement plant (orange) in the UK. The top 20 materials have been ranked for the nCAC, and this ranking is used for all KPIs. Hence, material ten for coal is the same for all KPIs associated with coal, but material ten can differ for cement and NGCC.

In Figures S40 and S41, we show the four economic KPIs the platform computes and the LCA KPI CC for the top 20 materials with the lowest nCAC. We can identify approximately 17-18 materials for each Case Study that have more or less the same cost (those in the corresponding "plateau" sections we can find for all the cost KPIs in Figure S40), and a few materials that have significantly lower costs. In this section, we focus on discussing the cost

associated with the group of 17-18 materials as we want to avoid discussing cost estimates that rely on a single material. In Section 11, we show how we use feedback from the platform to identify more top-performing materials.

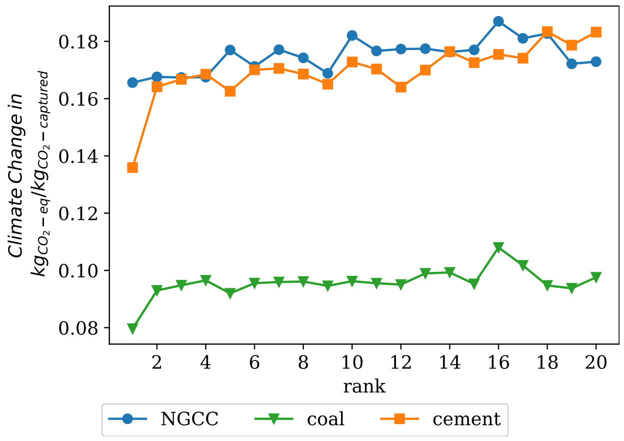

Figure S41| Top 20 materials in terms of the LCA KPI (CC) for a TVSA carbon capture process at 0.2 bar from an NGCC power plant (blue) and a TVSA carbon capture process at 0.6 bar from coal power plant (green) and cement plant (orange) in the UK. The top 20 materials have been ranked for the nCAC, and this ranking is used for all KPIs. Hence, material ten for coal is the same for all KPIs associated with coal, but material ten can differ for cement and NGCC.

Figure S40a shows that the LCOC, which gives us the discounted cost of running and operating the capture plant, is the highest for the NGCC power plant and cement plants and lowest for the coal-fired power plant. For the power plant Case Studies, we can use the available utilities at the host plant for the top-performing materials. Compared to the NGCC power plant, the coal-fired power plant additionally benefits from the higher CO_2 concentration in the flue gas. In a cement plant, the available utilities are insufficient to run the capture plant. As a result, in the considered Case Study, the electricity required is imported from the grid. A natural gas boiler supplies heat built on-site to regenerate the adsorbent. In some Case Studies, we use an electric boiler for regeneration (see Section 8.2.2). As only the costs of the additional utilities are accounted for in the LCOC, we get the high LCOC for cement compared to the power plant cases despite the high CO_2 concentration for the cement case.

For power plant cases, the loss of electricity production caused by the use of the utilities for the capture plant (parasitic load) increases the Levelised Cost of Electricity (LCOE) and thus the CCC due to a decreased amount of product (=electricity). In contrast, for cement, the amount of product (=cement) does not change and, thus, LCOC equals the CCC. Both economic KPIs would also be equal for power plants if all energy demands were supplied externally. In the CCC in Figure S40b, the parasitic load is accounted for. This increases the median CCC by ca. 19 €$t_{CO_2}^{-1}$ for the coal case and ca. 33 €$t_{CO_2}^{-1}$ for the NGCC case compared to the median LCOC.

The CCC is normalized by the amount of CO_2 captured. However, this KPI does not consider the CO_2 emissions from additional utilities, material synthesis and disposal, plant construction, and compensation of the parasitic load (in the case of power plants). In the CAC, the energy-related CO_2-eq. emissions from additional utilities and the impact of the parasitic load on the levelized cost of electricity and CO_2 intensity of the power plant are accounted for. The median CAC increases compared to the median CCC by ca. 15 €$t_{CO_2}^{-1}$, 10 €$t_{CO_2}^{-1}$, and 24 €$t_{CO_2}^{-1}$ for the coal, cement, and NGCC case, respectively (see Figure S40c).

Our platform also computes climate change impact (CC), including the CO_2-eq. emissions not only from additional utilities and the compensation of the parasitic load but also from material synthesis and disposal and plant construction. In Figure S41, the CC is shown for NGCC, coal, and cement; for every t of CO_2 we capture, we get a climate change impact of ca. 0.10, 0.18, and 0.17 t of CO_2-eq. for the coal, NGCC, and cement case, respectively. This implies that, for instance, for coal, if we capture 100 t of CO_2, 10 t of CO_2 will be emitted back into the atmosphere, hence effectively reducing the amount that is avoided to 90 t of CO_2. The CAC must be corrected to ensure all CO_2-eq. emissions are taken into account. For this correction, we introduce the Net Carbon Avoidance Cost (nCAC) (see Section 6.3.4) shown in Figure S40d. Note that the nCAC considers the system's perspective and accounts for operational costs and CO_2-eq. emissions of the parasitic load for power plant cases. As expected, the nCAC results in higher cost than the CAC. While the increase of the median

nCAC compared to the median CAC is very small for the cement case ($0.1\,€t^{-1}_{CO_2}$), it is more significant for the power plant cases, i.e., $13\,€t^{-1}_{CO_2}$ for the coal case and $42\,€t^{-1}_{CO_2}$ for the NGCC case. As a result, compared to the CCC, we see an increase in the median nCAC of $28\,€t^{-1}_{CO_2}$, $10\,€t^{-1}_{CO_2}$, and $66\,€t^{-1}_{CO_2}$ for the coal, cement, and NGCC case, respectively (see Figure S40d) if we take all emissions and the system's perspective into account. From this discussion, we conclude that the nCAC is the best cost metric to compare the various Case Studies.

8.3.2 Different sources

Capturing CO_2 from NGCC power plants gives the highest nCAC and from cement plants the lowest. Interestingly, while the results from the CAC differs from what we would expect from only thermodynamic arguments, which would predict the lowest cost for the source with the highest CO_2 concentration, the results for the nCAC is now in line with the thermodynamic arguments. In Figure S42, we break down the nCAC into contributions from CAPEX and OPEX. The OPEX is separated into the fixed OPEX, which is a percentage of the CAPEX, and the variable OPEX, which includes the costs associated with the utilities and replacement of the adsorbent. Cement and coal have the lowest CAPEX and fixed OPEX; $18\,€t^{-1}_{CO_2}$ for the coal and cement case versus $53\,€t^{-1}_{CO_2}$ for the NGCC case. This reflects the thermodynamic argument: the higher the CO_2 concentration in the flue/industrial gas, the larger the working capacity of the materials. Hence, we need less material and equipment to capture a unit mass of CO_2. We also observe that the variable OPEX for coal and cement is notably lower than that for NGCC, indicating lower energy requirements per tonne of captured CO_2 due to the higher CO_2 concentrations. Despite this, the predominant cost component for coal and cement remains the variable OPEX, accounting for approx. $47\,€t^{-1}_{CO_2}$ and $42\,€t^{-1}_{CO_2}$, respectively. This constitutes a contribution to the nCAC of around 72 % for coal and 70 % for cement.

The results highlight that the simple thermodynamic argument holds regarding total energy demand. Still, the final net costs per CO_2 (avoided) additionally depend strongly on the available utilities and the specifics of the Case Study. Thus, considering our new proposed Net Carbon Avoidance Cost (nCAC) definition is crucial for comparing the costs for carbon capture applications.

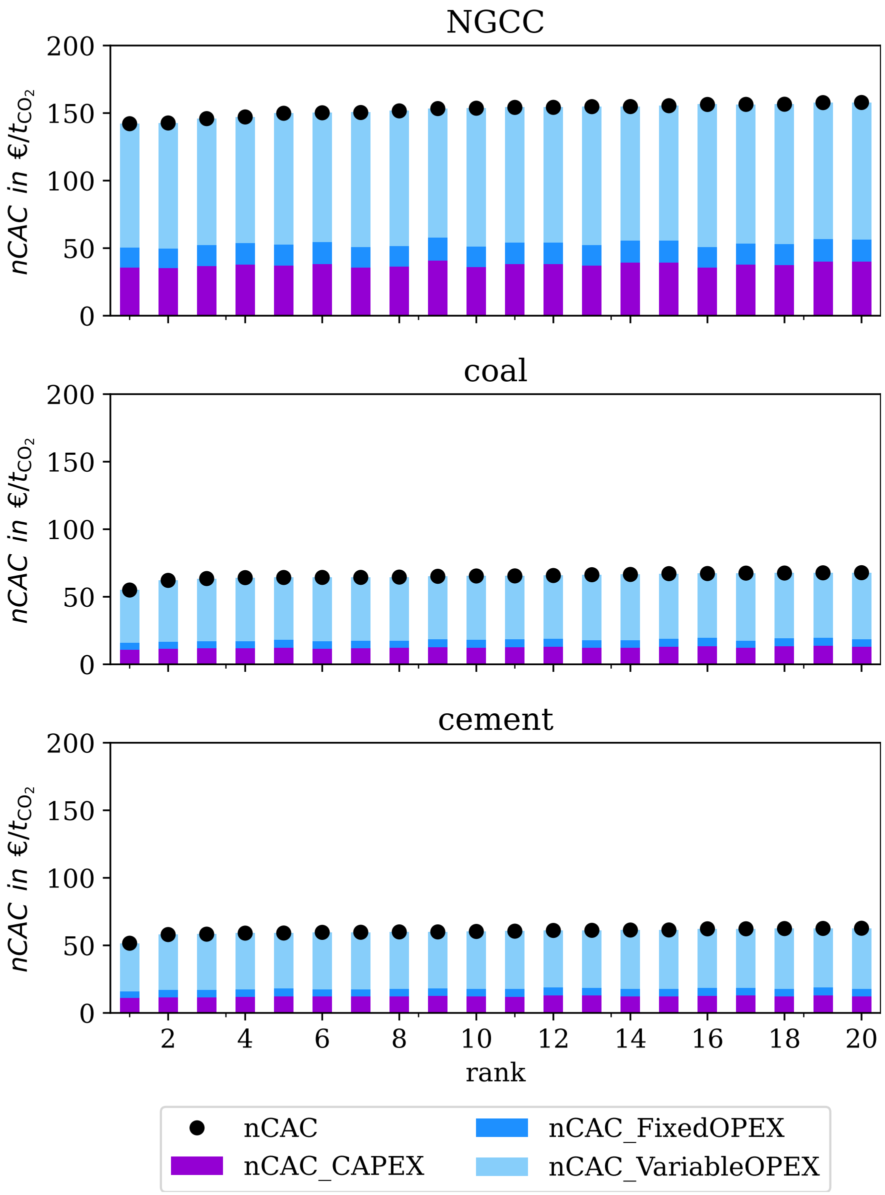

(a)

Figure S42| Breakdown of the nCAC of the top 20 materials for (a) NGCC power plants using a TVSA process with 0.2 bar, (b) coal power plants and (c) cement plants using a TVSA process with 0.6 bar in the UK . The costs are grouped into CAPEX (purple) and OPEX (blue). For the OPEX, we have separated the fixed OPEX (dark blue), which is a percentage of the CAPEX, and the variable OPEX (light blue), which mainly involves the cost related to the energy requirements of the process. The black dots are the total nCAC values.

8.4 The investor's perspective

So far, we have mainly restricted our comparison in terms of nCAC to power generation and cement plants in the UK. In our platform, we can change the region, and we have also computed the breakdown of the nCAC for the three CO_2 sources, the three technologies, and for the other four regions, i.e., the US (Figure S43), China (Figure S44 for Guangdong and Figure S45 for Shandong), and Switzerland (Figure S46). Coal is not used for power generation in Switzerland.

8.4.1 Different regions in the world

We arrive at the same conclusions concerning the preferred technologies for the US, China, and Switzerland as in the UK. For the NGCC power plant, we obtain the lowest nCAC for TVSA with a vacuum pressure of 0.2 bar. For the coal power plant and cement, the nCAC for TVSA with a vacuum pressure of 0.6 bar is the lowest. A summary of these data for these preferred technologies is given in Figures S47 to S49.

These figures show some interesting trends. For power generation, the calculation of the CAC considers that, when possible, the energy required for carbon capture is supplied by the host power plant, and this is the case for the top 20 materials (see Section 6.3). The higher CAC in comparison to the CCC arises from the decrease in power output. On the contrary, the region-dependent CO_2-eq. emissions from the energy supply have a significant impact on the CAC for cement plants. The higher increase in costs when comparing the CCC and the CAC is observed for the GD region in China, which presents the highest CO_2-eq. emissions from the energy supply. The high CO_2-eq. emissions from the energy supply is also reflected in the CC, as illustrated in Figure S49.

Another important region-dependent factor on the CAC in the power plant cases is also the CO_2 intensity of the reference power plant. In our calculations of the CAC, we assume that the reference power plant itself is the same (i.e., has the same direct CO_2 intensity), but we consider a region-dependent indirect CO_2 intensity (e.g., for coal mining, production,

or transportation). Consequently, the CAC values are very similar for the Chinese regions despite the very different CC because the CO_2 intensity of the reference power plant is very similar in both provinces. In contrast, for the coal case, the indirect CO_2 intensity of the reference power plant is lower for the US, leading to smaller CAC values.

The nCAC is directly linked to the CC and accounts for the cost and region-dependent CO_2-eq. emissions associated with compensating for the parasitic load (see Section 6.3). Hence, it is essential to take into account the impact of region-dependent CO_2-eq. emissions (i.e., the CC) and the regional electricity price when comparing the nCAC across different regions.

Regions with high CC, such as China, present a higher increase in nCAC. This is particularly noticeable for the NGCC plant in the GD region in China (see Figure S47). For regions with high electricity prices but substantially lower CC, such as Switzerland, the nCAC becomes smaller than the CAC since the large CO_2 avoided emissions compensate for the increase in variable OPEX (see Figure S47).

In Figure S48, we see that for coal, the Carbon Capture Cost (CCC) in the two provinces of China and the US are the lowest. However, the corresponding CO_2-eq. emissions (i.e., the CC) in these two Chinese provinces are about 40 % higher for the GD region and more than 100 % higher for the SD region than those in the US. The strong difference in CC in the two Chinese provinces is counteracted by the lower electricity price in the SD region, and consequently, we obtain similar nCAC, i.e., on average $57\,€\,t_{CO_2}^{-1}$ for GD and $59\,€\,t_{CO_2}^{-1}$ for SD. These values are about $13\,€\,t_{CO_2}^{-1}$ to $15\,€\,t_{CO_2}^{-1}$ higher than the one in the US, i.e., $45\,€\,t_{CO_2}^{-1}$.

For NGCC Case Studies, shown in Figure S47, the CCC is clearly highest in Switzerland, mainly due to the highest CAPEX and OPEX. As Switzerland also has one of the highest indirect CO_2 intensities of the reference NGCC power plant, the CAC is still the highest in Switzerland, whilst they stay lowest in the US. In contrast, the nCAC and CC in Switzerland are the lowest amongst all studied countries because of the very low carbon intensity of the

electricity grid, accounted for in the nCAC when evaluating the avoided emissions over the lifetime of the plant.

For cement, we obtained the highest costs in Switzerland and the lowest costs in the US. In Switzerland, the CC of the cement plant is higher than the CC of the NGCC power plant due to the CO_2-eq. emissions from the natural gas boilers used for the heat supply. As a result, the low CC in Switzerland cannot compensate for the high OPEX and the nCAC is still highest in Switzerland. For the other countries, the region-dependency of the CC balances off the CAC and nCAC.

8.4.2 Utility prices

An important factor in the cost is the utility prices. In our calculations, we have used 2019 as a reference. In 2022, the prices of utilities increased significantly because of global tensions. In Figure S50, we show the effect of these increases in energy prices for the cement Case Study in the UK. For cement, the nCAC has increased by about 50 % to 54 % .

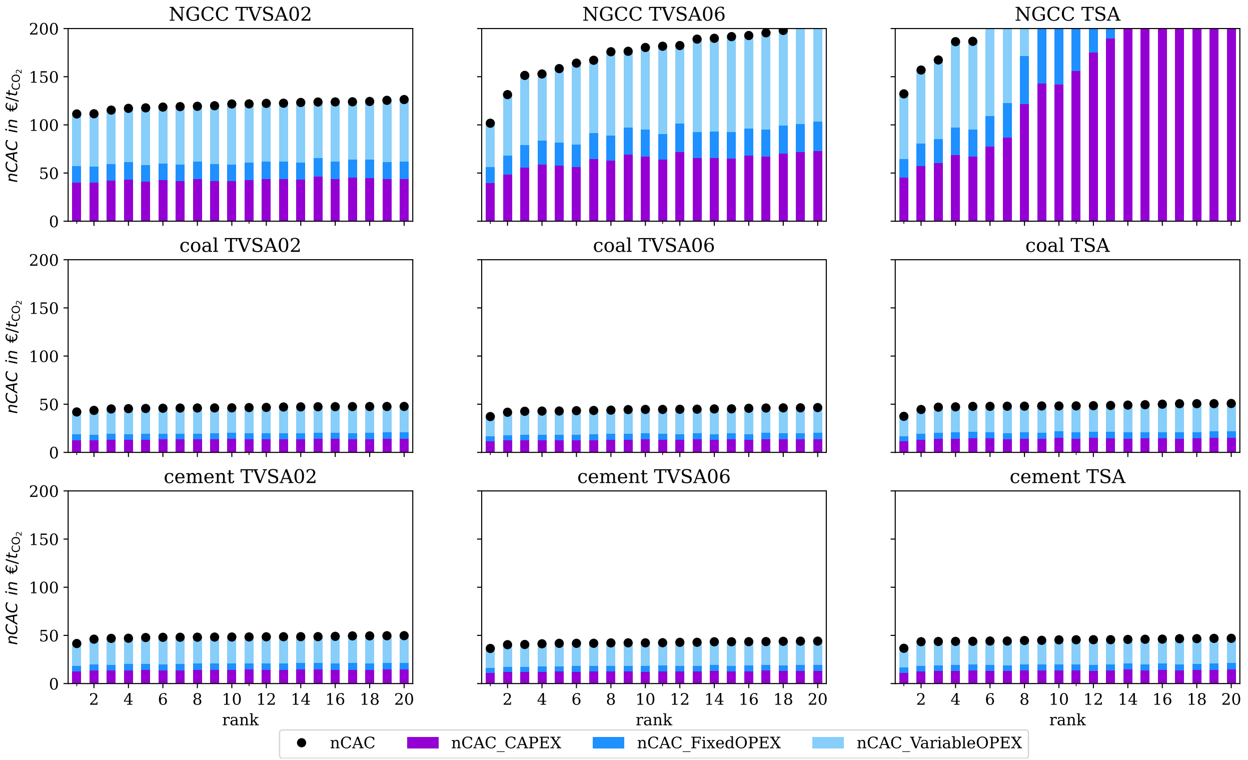

Figure S43| Breakdown of the nCAC of the top 20 materials for (top) NGCC power plants, (middle) coal power plants, and (bottom) cement plants in the US, for the technologies TVSA with a vacuum pressure of 0.2 bar (left) and 0.6 bar (middle) and TSA (right). The costs are grouped into CAPEX (purple), fixed OPEX (dark blue), which is a percentage of the CAPEX, and variable OPEX (light blue), which mainly involves the cost related to the energy requirements of the process. The black dots are the total nCAC values.

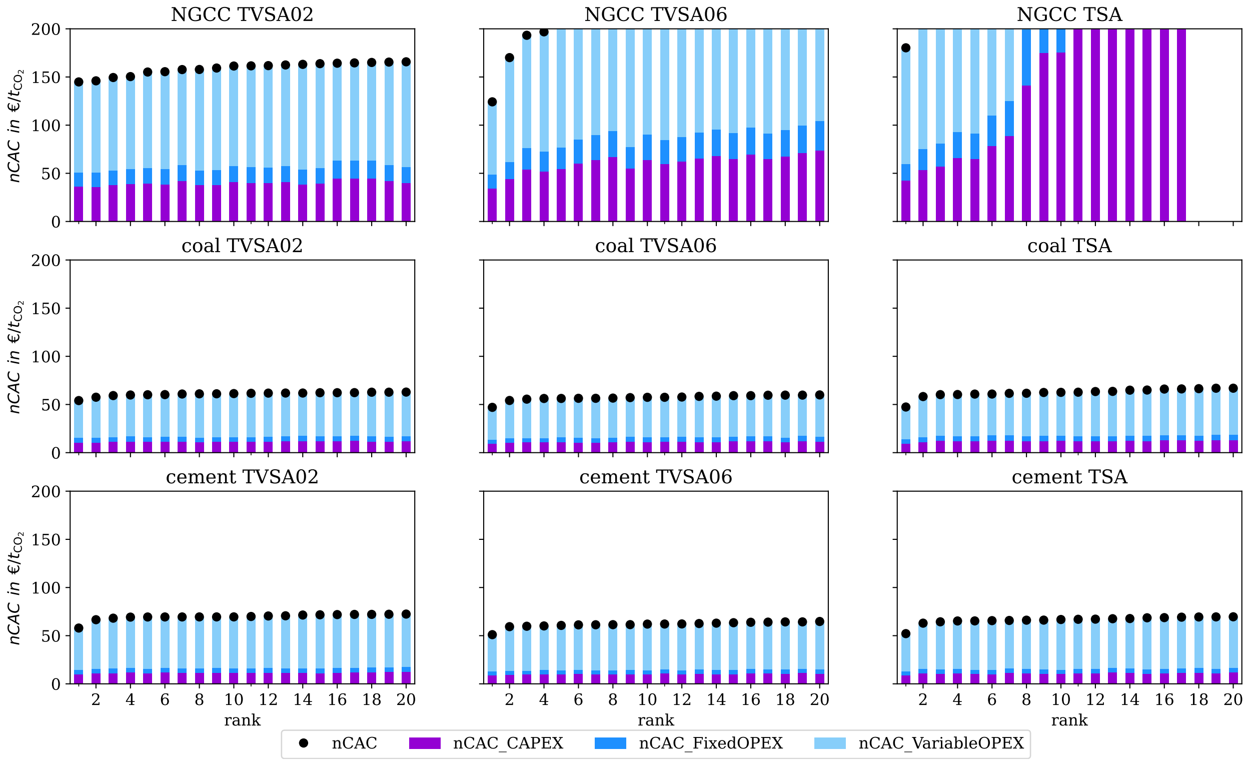

Figure S44| Breakdown of the nCAC of the top 20 materials for (top) NGCC power plants, (middle) coal power plants, and (bottom) cement plants in China (Guangdong province), for the technologies TVSA with a vacuum pressure of 0.2 bar (left) and 0.6 bar (middle) and TSA (right). The costs are grouped into CAPEX (purple), fixed OPEX (dark blue), which is a percentage of the CAPEX, and variable OPEX (light blue), which mainly involves the cost related to the energy requirements of the process. The black dots are the total nCAC values. For TSA, not all materials in the top 20 resulted in an effective recovery above 0; these materials have been omitted.

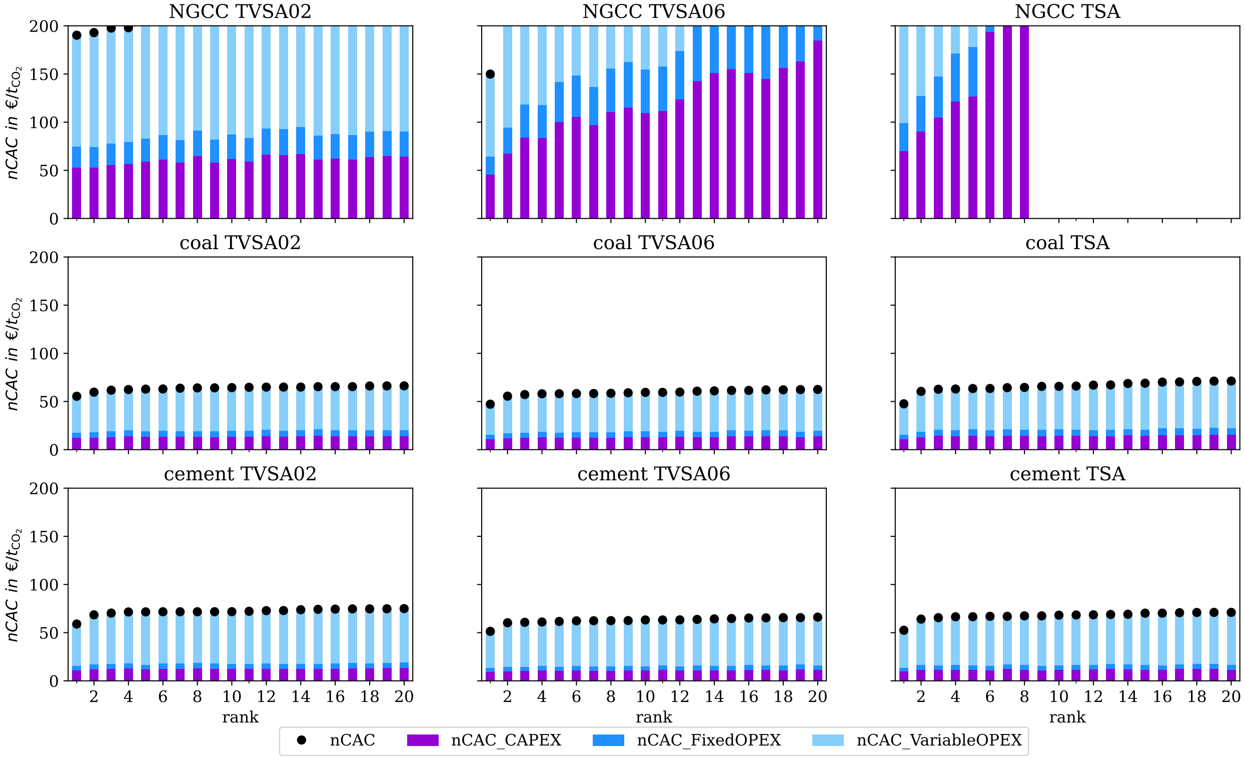

Figure S45| Breakdown of the nCAC of the top 20 materials for (top) NGCC power plants, (middle) coal power plants, and (bottom) cement plants in China (Shandong province), for the technologies TVSA with a vacuum pressure of 0.2 bar (left) and 0.6 bar (middle) and TSA (right). The costs are grouped into CAPEX (purple), fixed OPEX (dark blue), which is a percentage of the CAPEX, and variable OPEX (light blue), which mainly involves the cost related to the energy requirements of the process. The black dots are the total nCAC values. For TSA, not all materials in the top 20 resulted in an effective recovery above 0; these materials have been omitted.

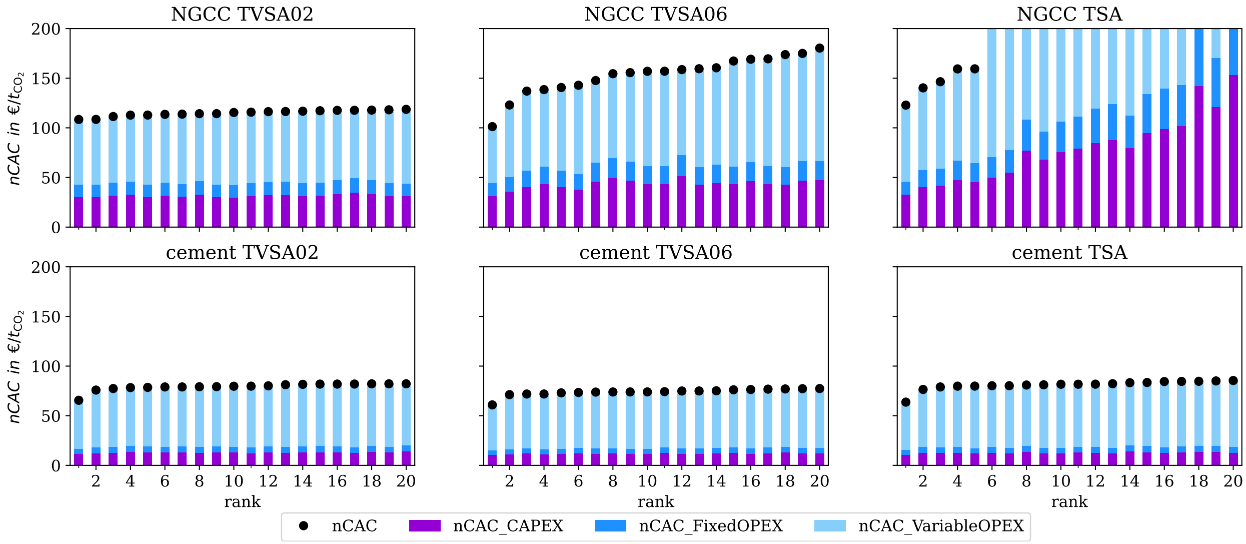

Figure S46| Breakdown of the nCAC of the top 20 materials for (top) NGCC power plants and (bottom) cement plants in Switzerland, for the technologies TVSA with a vacuum pressure of 0.2 bar (left) and 0.6 bar (middle) and TSA (right). The costs are grouped into CAPEX (purple), fixed OPEX (dark blue), which is a percentage of the CAPEX, and variable OPEX (light blue), which mainly involves the cost related to the energy requirements of the process. The black dots are the total nCAC values.

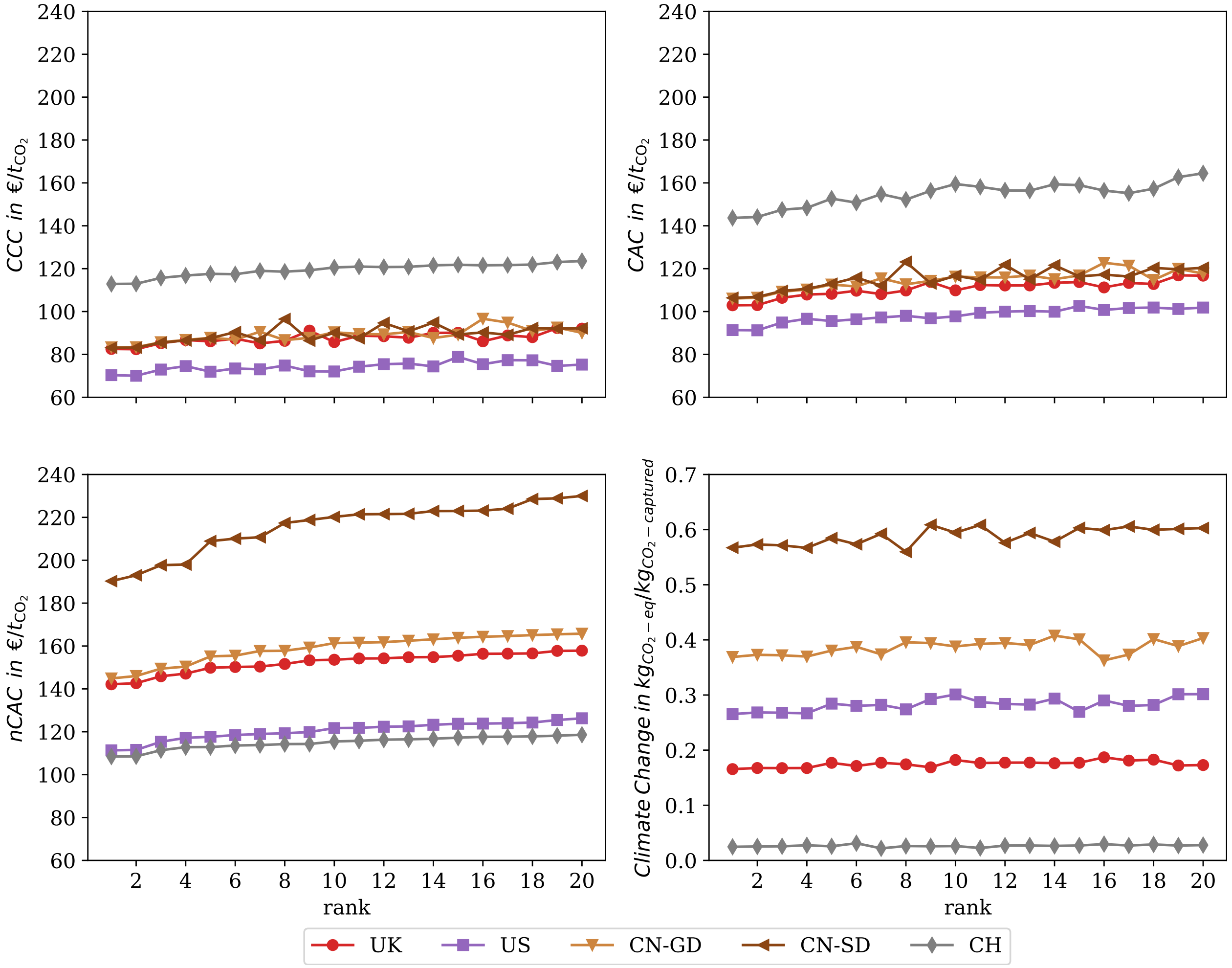

Figure S47| Comparison of KPIs for NGCC in the five considered regions. The figure shows the top 20 materials (ranked according to the nCAC using TVSA with a pressure of 0.2 bar).

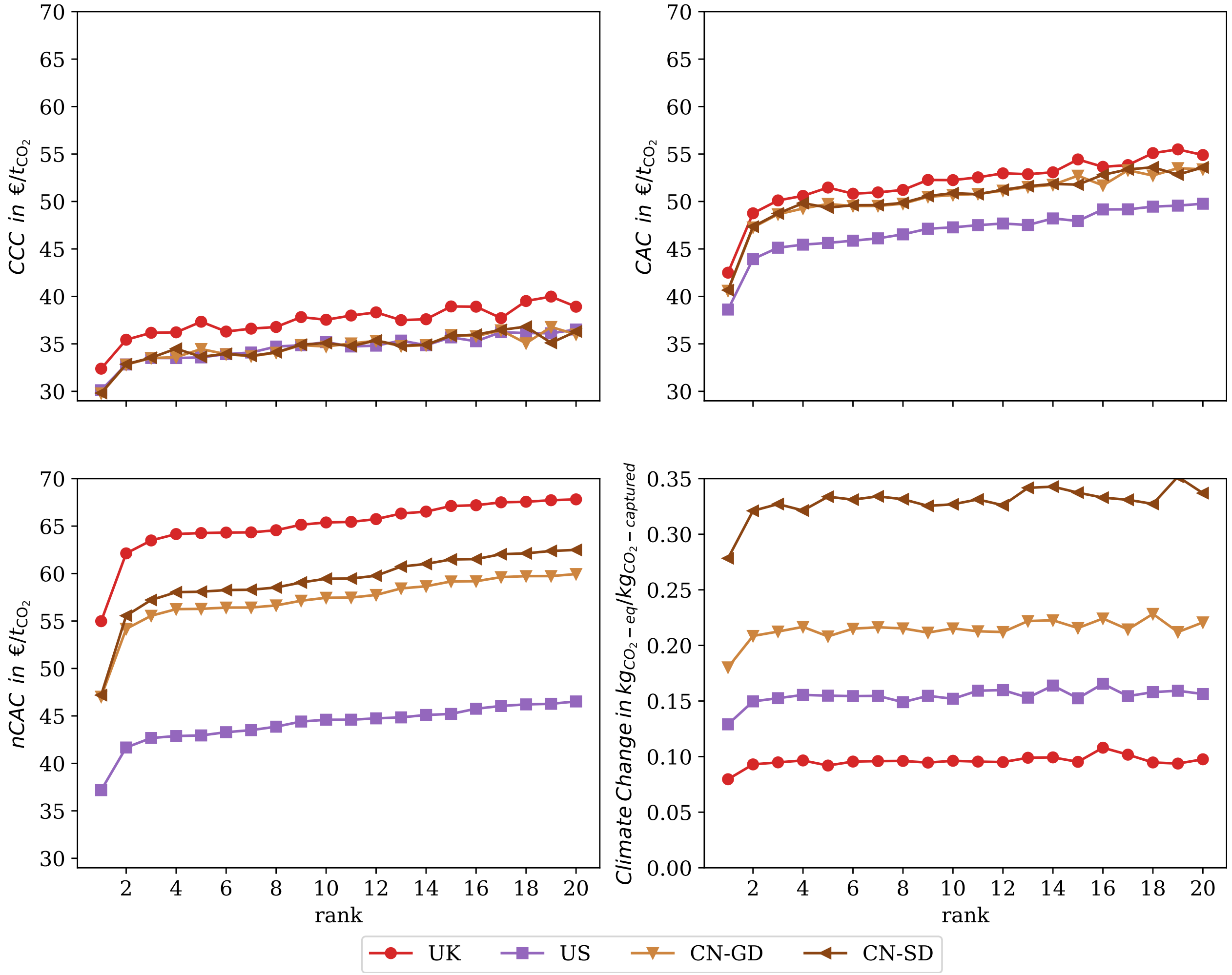

Figure S48| Comparison of KPIs for coal in the four considered regions. The figure shows the top 20 materials (ranked according to the nCAC using TVSA with a pressure of 0.6 bar).

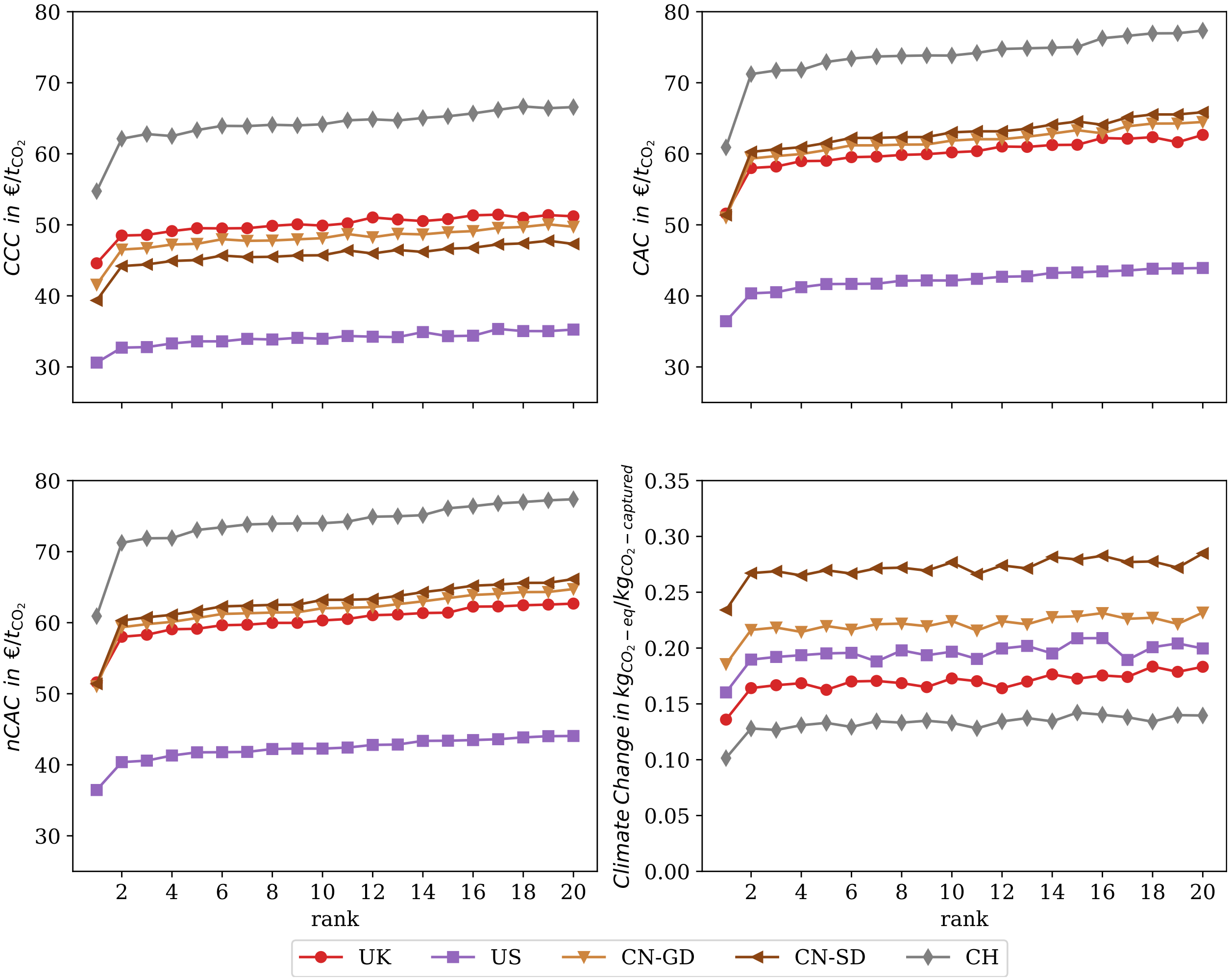

Figure S49| Comparison of KPIs for cement in the five considered regions. The figure shows the top 20 materials (ranked according to the nCAC using TVSA with a pressure of 0.6 bar).

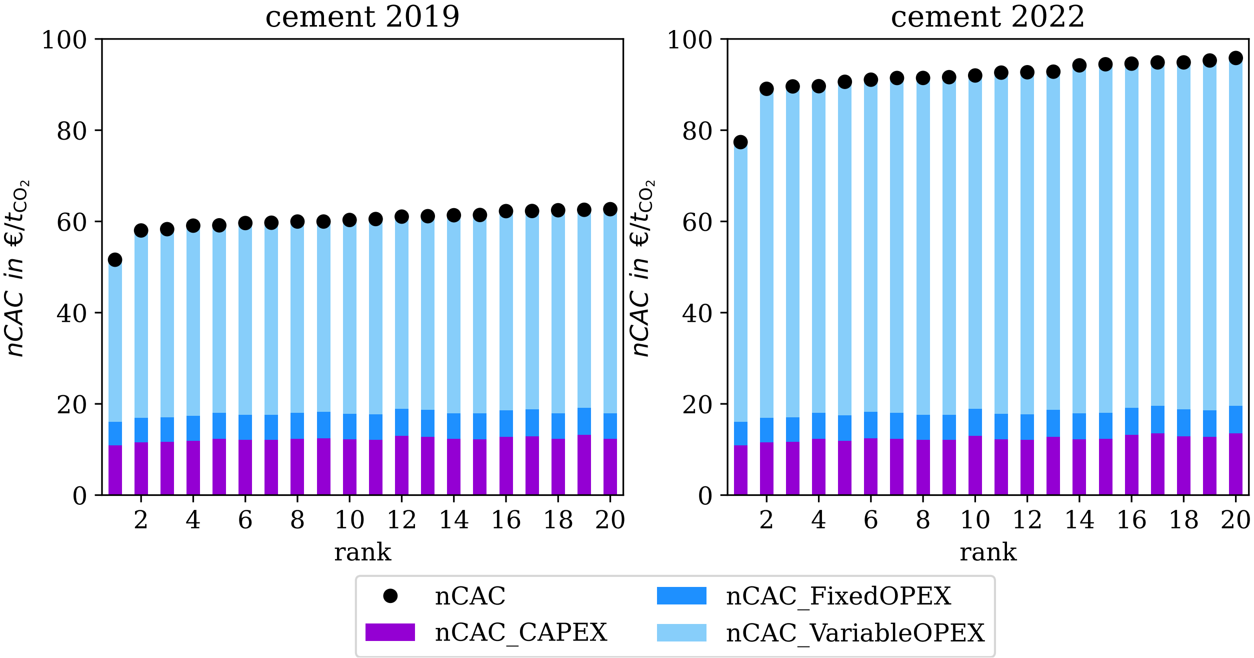

Figure S50| Comparison of the nCAC for cement in the UK using energy prices from 2022 versus those of 2019. The figure shows the top 20 materials (ranked according to the nCAC using TVSA with a pressure of 0.6 bar).

8.4.3 The cost and replacement rates of the MOFs

One of the features of our PrISMa platform is that we predict the nCAC of materials that have not yet been synthesized. Or materials that have been synthesized on the milligram scale in the laboratory. This makes it difficult to estimate the costs reliably. In the LCA KPI, MR:MM, the impact of mining of the metal and its abundance is accounted for. We assume a uniform price of 30 € kg^{-1} for all MOFs. This value is based on a recent analysis of the cost of some MOFS.[S77]

In Figures S51 and S52, we compare how the cost of the MOF impacts the nCAC for a more optimistic scenario in which the cost drops to 7.5 € kg^{-1} and a more pessimistic scenario of 70 € kg^{-1}. These figures show that the impact on the nCAC is rather modest; about 4 €$t_{CO_2}^{-1}$ (7 %) lower for the more optimistic scenario and about 7 €$t_{CO_2}^{-1}$ (12 %) higher for the scenario with the highest cost of the MOF.

The nCAC breakdown also shows that, compared to the base case, in the 7.5 € kg^{-1} scenario, about 2 €$t_{CO_2}^{-1}$ (15 %), 0.7 €$t_{CO_2}^{-1}$ (13 %), and 1.6 €$t_{CO_2}^{-1}$ (4 %) drops are observed for the CAPEX, fixed and variable OPEX contributions, respectively. The decrease in the CAPEX reflects the lower cost of the MOF. As the fixed OPEX is calculated as a percentage of the CAPEX, approximately the same percentage decrease is observed. The change in variable OPEX accounts for the cost of replacing the MOF every five years. For the 70 € kg^{-1} scenario, about 3 €$t_{CO_2}^{-1}$ (23 %) increase in the CAPEX contribution, 1 €$t_{CO_2}^{-1}$ (21 %) increase in the fixed OPEX contribution, and about 3 €$t_{CO_2}^{-1}$ (7 %) increase in the variable OPEX contribution are observed.

Our calculations assume that the sorbent is replaced every five years. In Figures S53 and S54 we show the effect of changing this replacement rate (2, 5, and 7 yr) on the nCAC. These results show that a shorter lifetime increases the nCAC significantly (ca. 4 €$t_{CO_2}^{-1}$) while extending the lifetime beyond five years has less impact (ca. 0.6 €$t_{CO_2}^{-1}$). These conclusions are similar to those obtained by Balogun et al.[S211].

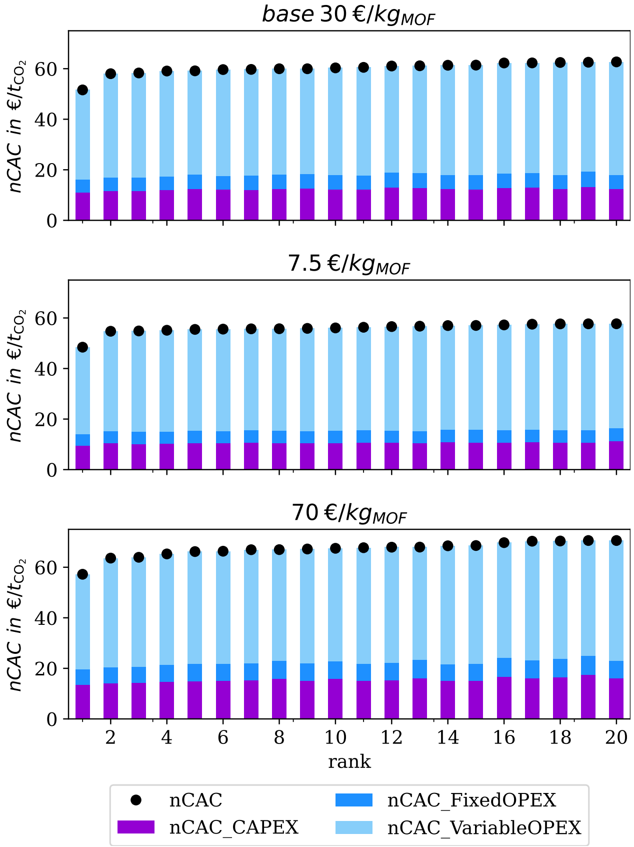

Figure S51| Effect of the cost of a MOF on the nCAC Comparison of the nCAC for our reference case (cement in the UK, TVSA, 0.6 bar) and for the top 20 best-performing materials using a MOF cost of 7.5, 30, and 70 € kg^{-1}.

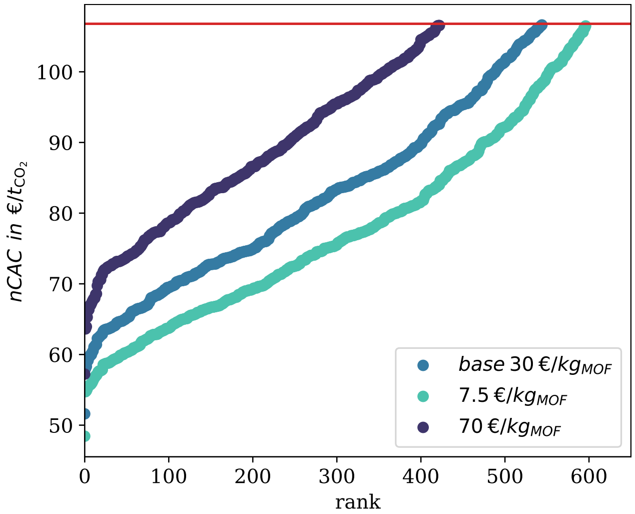

Figure S52| Effect of the cost of a MOF on the nCAC Comparison of the nCAC for our reference case (cement in the UK, TVSA, 0.6 bar) for all materials that outperform MEA using a MOF cost of 7.5, 30, and 70 € kg^{-1}.

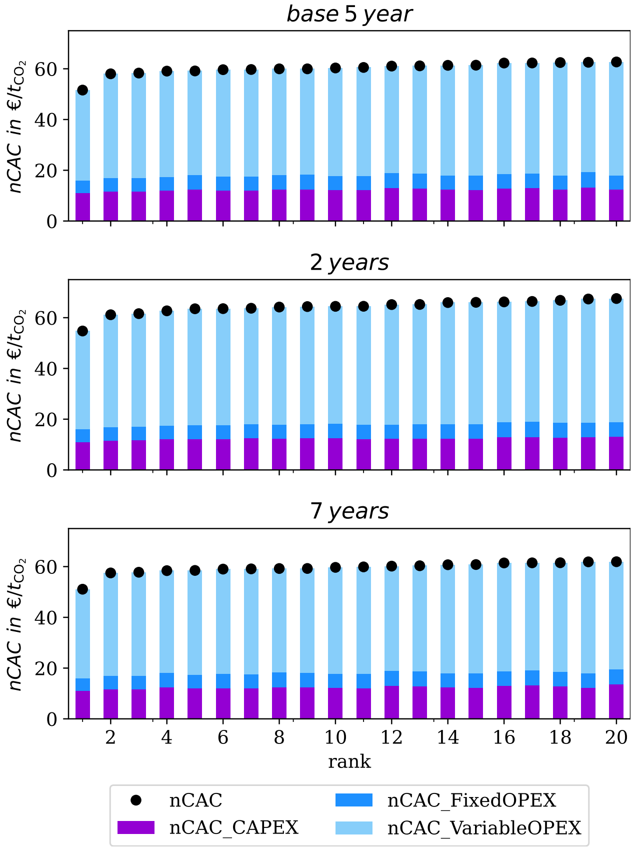

Figure S53| Effect of the replacement rate of a MOF on the nCAC Comparison of the nCAC for our reference case (cement in the UK, TVSA, 0.6 bar vacuum) and for the top 20 best-performing materials using a replacement rate of the sorbent of 2, 5, and 7 yr.

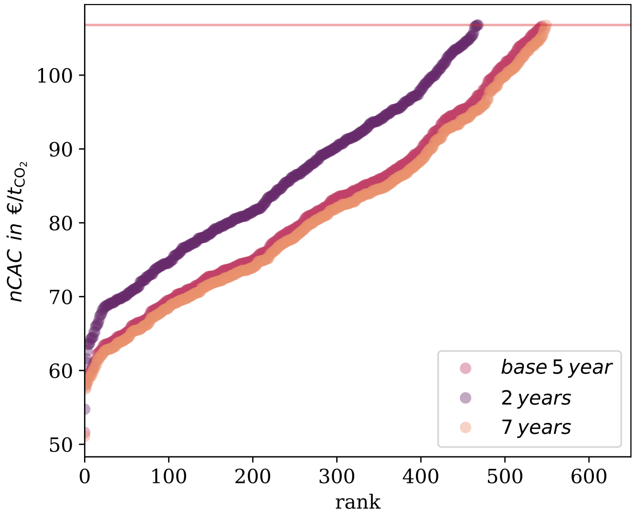

Figure S54| Effect of the replacement rate of a MOF on the nCAC Comparison of the nCAC for our reference case (cement in the UK, TVSA, 0.6 bar) and for all materials that outperform MEA using a replacement rate of the sorbent of 2, 5, and 7 yr.

8.5 The chemist's perspective

8.5.1 Materials across Case Studies

Our platform has ranked materials for various Case Studies and KPIs using three process technologies, TSA, and TVSA with two vacuum levels. Ideally, one would like to use the same top-performing material for all Case Studies. To get some insights if such material exists, we look at our key KPIs (purity, productivity, nCAC, CC, and MR:MM). Figure S55 shows a Venn diagram in which the numbers indicate the total number of common structures when considering overlapping KPIs. This analysis was conducted for the top 50, 100, 150, and 200 materials using all the screening results from a subset of 22 Case Studies: the source-sink-region-utility combinations where a preferred process configuration was selected for each CO_2 source (see Section 8.1).

Suppose we look at the diagram for the top 50. We see that, for instance, among the 22 Case Studies, 25 ($= 23 + 1 + 1$) materials are common in the top 50 in terms of productivity, and 12 ($= 3 + 1 + 1 + 2 + 4 + 1$) materials in terms of CC; and one material in the top 50 for both KPIs. Considering all five key KPIs, we see that not a single material is in common. If we take the top 100, we find six materials in common with all KPIs, and we need to look at the top 150 and 200 to have a more significant number (14 and 26, respectively).

8.5.2 Effect of the flexiblity

We have carried out molecular dynamics simulations on the top 60 of the structures with the lowest nCAC. We assess the flexibility from the simulations by monitoring the standard deviation (see Section 3.1.3). In Section 5.6, we have shown that differences between experimental isotherms and the predictions of our simulations can be of one order of magnitude in the Henry coefficient. We use a similar criterium to flag structures for which flexibility might be an issue. Figure S56 shows that out of these 60 materials, eight are flagged. In addition, there are eleven materials for which the UFF4MOF force field gives a very different

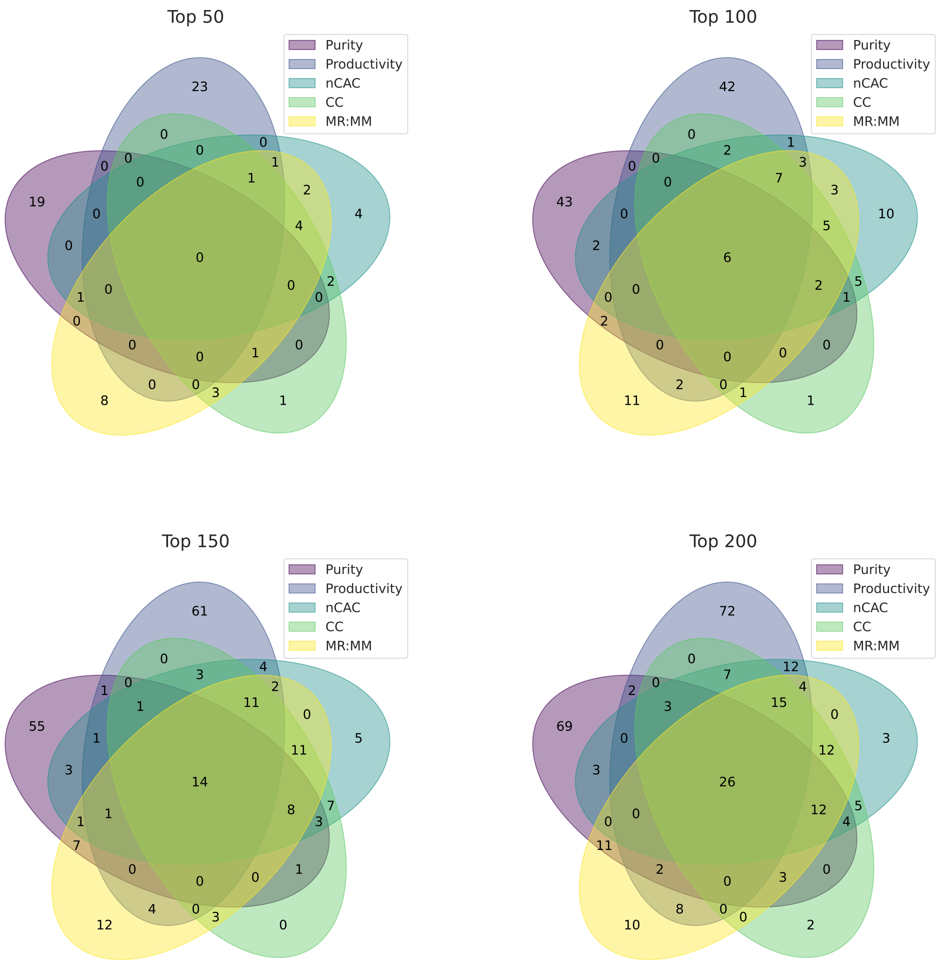

Figure S55| Common materials across 22 Case Studies: The number of common materials for five reference KPIs (purity, productivity, nCAC, CC, and MR:MM) for a subset of 22 Case Studies in the top 50 (a), 100 (b), 150 (c), and 200 (d). This subset includes NGCC with TVSA at 0.2 bar, coal with TVSA at 0.6 bar, and cement with TVSA at 0.6 bar for all regions and different boilers.

structure after energy minimization compared to a minimization using DFT, which we use as part of our workflow (see Section 3.1.1). Those structures most likely contain metals or parts of the linker for which the UFF4MOF fails.

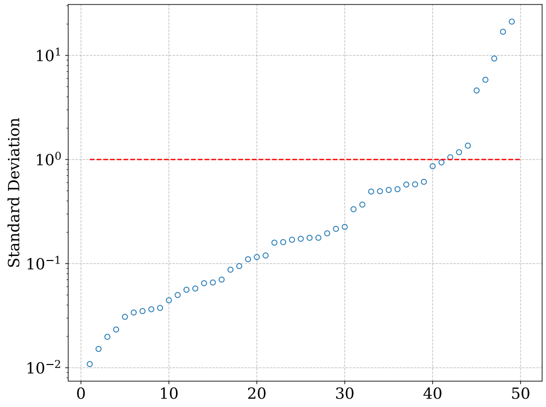

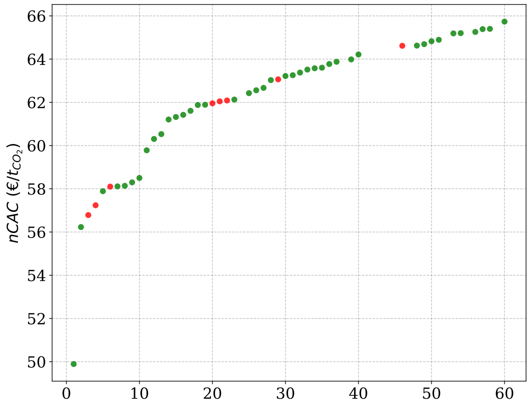

(a) **(b)**

Figure S56| Effect of the flexibility on the adsorption properties (a) ranking of the materials in terms of the absolute error of the log H (b) ranking of the nCAC where materials are labeled red if their standard deviation of the Henry coefficient is more than one order of magnitude.

8.5.3 Identifying the adsorbaphores

We generate large data sets in the PrISMa platform. This data generation allows us to use machine-learning methods to analyze the data. In particular, to identify the molecular characteristics of the top performing materials (e.g., the adsorbaphore[S33]). To identify these characteristics, we first build a machine-learning model with the most general descriptors used in the literature to characterize MOFs. From the analysis of the importance of the features of these descriptors, we can then focus on the most essential features.

We conduct this analysis for our exemplary Case Study, i.e., carbon capture from a cement plant located in the UK where a TSA technology is used, and the CO_2 is sent for geological storage. In the first step, we use a general featurization of the MOFs using atomic-

property labeled autocorrelation functions (APRDF),[S212] revised autocorrelation functions (RACs),[S21,S213] pore geometric features,[S16] and persistence images and persistence homology statistics[S214,S215] as implemented in mofdscribe.[S86]

Table S6: **Hyperparameters ranges considered for the XGBoost models.**[S216]

Parameter	Range
`learning rate`	1×10^{-2}–0.25 (log)
λ	1×10^{-8}–100.0 (log)
α	1×10^{-8}–100.0 (log)
`subsample`	0.1 – 1.0
`colsample_bytree`	0.1–1.0
`max_depth`	1–9 (int)
`n_estimators`	[7000, 15000, 20000]

We subsequently fit a gradient-boosted decision tree as implemented using XGBoost[S216] to classify compounds as being below or above the MEA-based absorption benchmark in terms of nCAC (see Figure 2(a)). We optimize the hyperparameters with the tree of Parzen estimators[S217] strategy as implemented in optuna[S218] for 100 steps over the hyperparameter ranges listed in Table S6. The model is subsequently tested ten times (re-tuning the hyperparameters each time in an inner cross-validation loop) on a holdout set of 20 % the total dataset. We identify the average confusion matrix shown in Figure S57a and metrics summarized in Table S7, indicating that the model is predictive.

Table S7: **Classification performance of an XGBoost model trained on all features.** The F_1 score measures classifier performance, with the perfect score being one and the worst one being 0. It is defined as the harmonic mean of the precision (fraction of correct predictions instances among all predictions for a class) and recall (fraction of true positives among all positives). The κ score is a related metric that ranges from -1 to 1, where a score of 0 would equal chance prediction, and one would be a perfect classifier.

Metric	Mean	Standard deviation
accuracy	0.76	0.03
F_1 macro	0.75	0.04
F_1 micro	0.76	0.03
κ	0.50	0.07

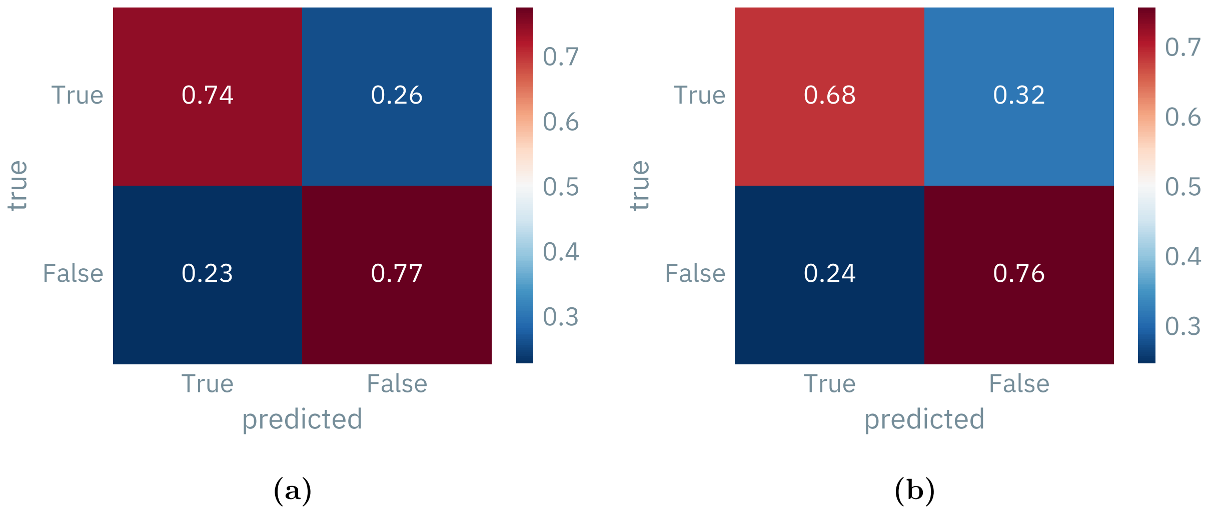

Figure S57| (a) Average confusion matrix for an XGBoost model trained on all features and (b) Average confusion matrix for an XGBoost model trained on persistence images.

We then perform a global feature importance analysis using the gain-based feature importance inherent to the gradient-boosted trees. This analysis highlights that the features derived from the persistence homology are very predictive (Figure S58). Since persistence images, in principle, allow back mapping to structural features, we focus on the persistence images and retrain the XGBoost model using only these persistence images[S219] as features.

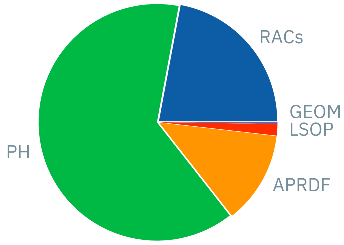

Figure S58| Overall gain feature importance of an XGBoost model trained on all features.

Note that the persistence images implementation in mofdscribe allows for chemical resolution by performing the analysis on substructures spanned by different chemistries (e.g., only metals, only C, H, N, O; see Fig. 4 in Jablonka et al.[S86]). We test the model ten times on a holdout set (again 20 %) and find the average confusion matrix shown in Figure S57b

and summarized in Table S8. The performance is (within the error margins) equivalent to the model trained on the full feature set.

Table S8: **Classification performance of an XGBoost model trained on persistence images.** The F_1 score measures classifier performance, with the perfect score being one and the worst one being 0. It is defined as the harmonic mean of the precision (fraction of correct predictions instances among all predictions for a class) and recall (fraction of true positives among all positives). The κ score is a related metric that ranges from -1 to 1, where a score of 0 would equal chance prediction, and one would be a perfect classifier.

Metric	Mean	Standard deviation
accuracy	0.73	0.02
F_1 macro	0.72	0.02
F_1 micro	0.73	0.02
κ	0.43	0.04

Given the good predictive performance, we compute SHAPley values[S220] for the ten top-performing MOFs after retraining the model on the full dataset. Shapley values attempt to attribute *importance* to features such that the attributions add up to the full predictions such that several desirable properties (efficiency, symmetry, dummy, and additivity) are satisfied. We use SHAPley values since they allow for a local attribution (i.e., the most relevant feature might differ for every structure). In the current analysis, we focus only on *positive* contributions, i.e., favoring the *below benchmark* class compared to the average case. Future analyses might also investigate the other class. We then extract the representative cycles (generators of the point in the persistence diagram) closest (Euclidean distance) to the most important pixel in the persistence images. This functionality is currently available in a development version of mofdscribe.[3]

Interestingly, the representative cycle points us to the atoms responsible for the most important feature determining whether a material performs better than the benchmark. This collection of atoms can be referred to as the adsorbaphore. Examples of these adsorbaphores are shown in Figures S59, S60 and S63. A common theme in the top-performing compounds

[3] Calling the `featurizer.find_relevant_substructure(s, feature_name)` method of the featurizer will return the relevant substructure.

is a geometrical rod of metal atoms, highlighted in these figures. Interestingly, these features are often correlated with stacked delocalized systems (aromatic rings) with separation of 6 Å to 11 Å (see, for example, Figures S60 and S62).

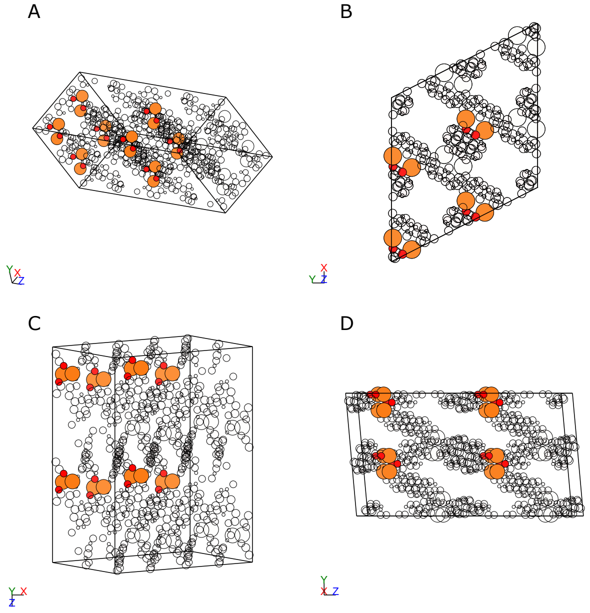

Figure S59| Most important substructure for RSM0851: The orange (Magnesium) and red spheres (Oxygen) are the atoms that form the geometrical rod feature corresponding to a top-performing material.

Prototypes To better understand the data distribution, we utilize the maximum mean discrepancy (MMD) critic framework[S221] (https://github.com/kjappelbaum/MMD-critic/) to select so-called prototypes. Those prototypes are data points for which the distribution optimally matches the original distribution:

$$\mathrm{MMD}^2 = \frac{1}{m^2}\sum_{i,j=1}^{m} k\left(z_i, z_j\right) - \underbrace{\frac{2}{mn}\sum_{i,j=1}^{m,n} k\left(z_i, x_j\right)}_{\text{mismatch between prototypes and data}} + \frac{1}{n^2}\sum_{i,j=1}^{n} k\left(x_i, x_j\right), \qquad \text{(S72)}$$

where k is a kernel function (in our case, the radial basis function kernel with $\gamma = 0.026$), m is the number of prototypes (in our case 6), and n is the number of data points in our

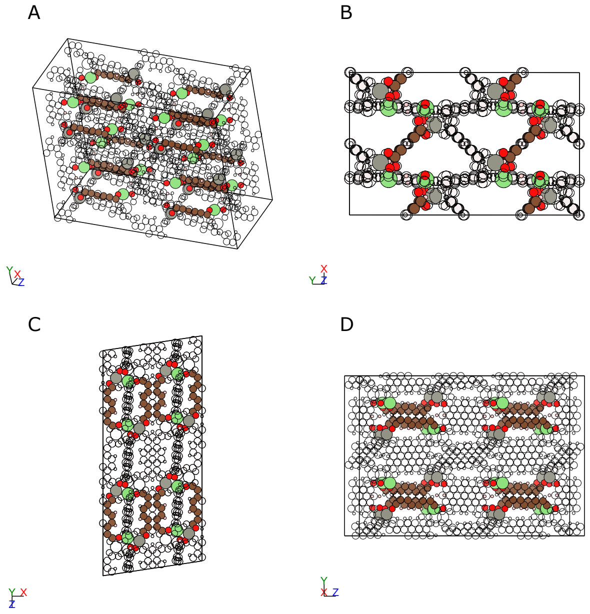

Figure S60| Important substructure for RSM1847: The brown (Carbon) spheres are the atoms that form stacked ring patterns. Gray balls indicate Zn, green Mg, and red O.

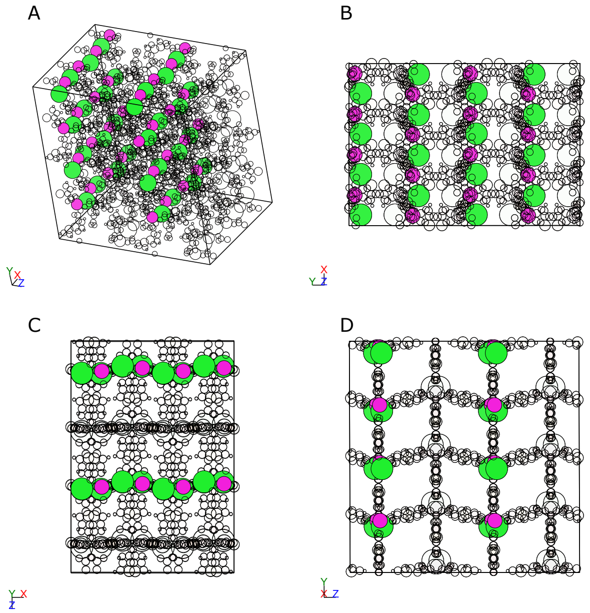

Figure S61| Important substructure for RSM2998: The green (Ba) and pink (Cd) spheres are the atoms that form the geometrical rod feature corresponding to a top-performing material.

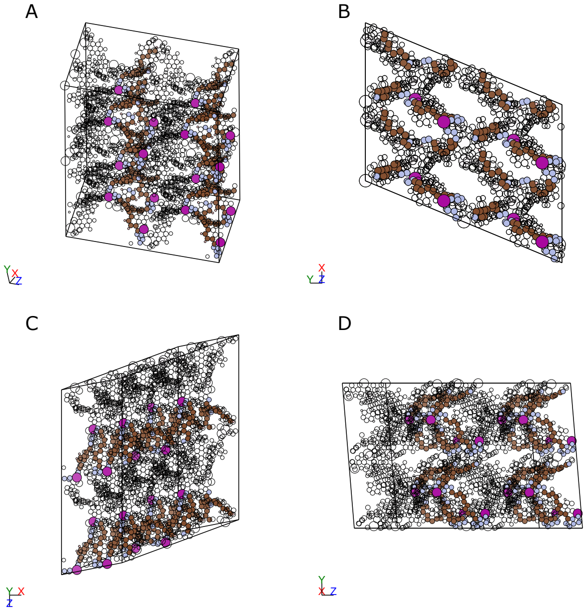

Figure S62| Important substructure for v0+bcu+N131+1164: The brown (carbon) and red (oxygen) spheres are the atoms that form the geometrical rod feature corresponding to a top-performing material. The side view in B shows the stacked aromatic rings. Pink balls indicate Mn and blue balls nitrogen.

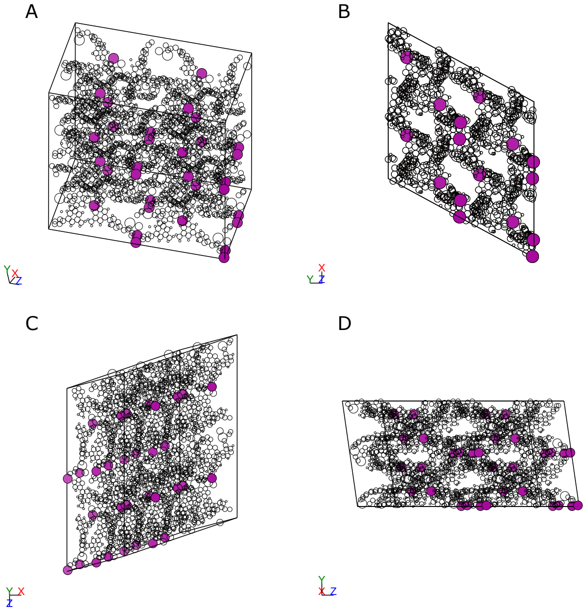

Figure S63| Important substructure for v0+bcu+N131+742: The pink balls (Mn) form a geometric rod.

dataset. Hence, this method identifies those materials that are most representative (i.e., a material with the most features in common) for a particular group.

For example, we can use this method to identify the prototypes of the materials with a high Water resistance coefficient (WRC) and a nCAC better than MEA. These prototypes are shown in Figure S64. The structures tend to have aromatic rings with a 6 Å separation.

In addition, Figure S65 shows the materials with the lowest Water resistance coefficient (WRC) and a CAC better than MEA. These are materials with Open metal site (OMS) and functional groups containing halogens.

Significance test We also performed statistical tests to assess the impact of OMS on water resistance. Comparing the two groups below and above the water resistance threshold, we find that a t-test finds a significant difference ($p < 0.01$) between those groups in the presence of OMS. There, we use the OMS definition in the `mofchecker` Python package (https://github.com/kjappelbaum/mofchecker/tree/main/src/mofchecker).

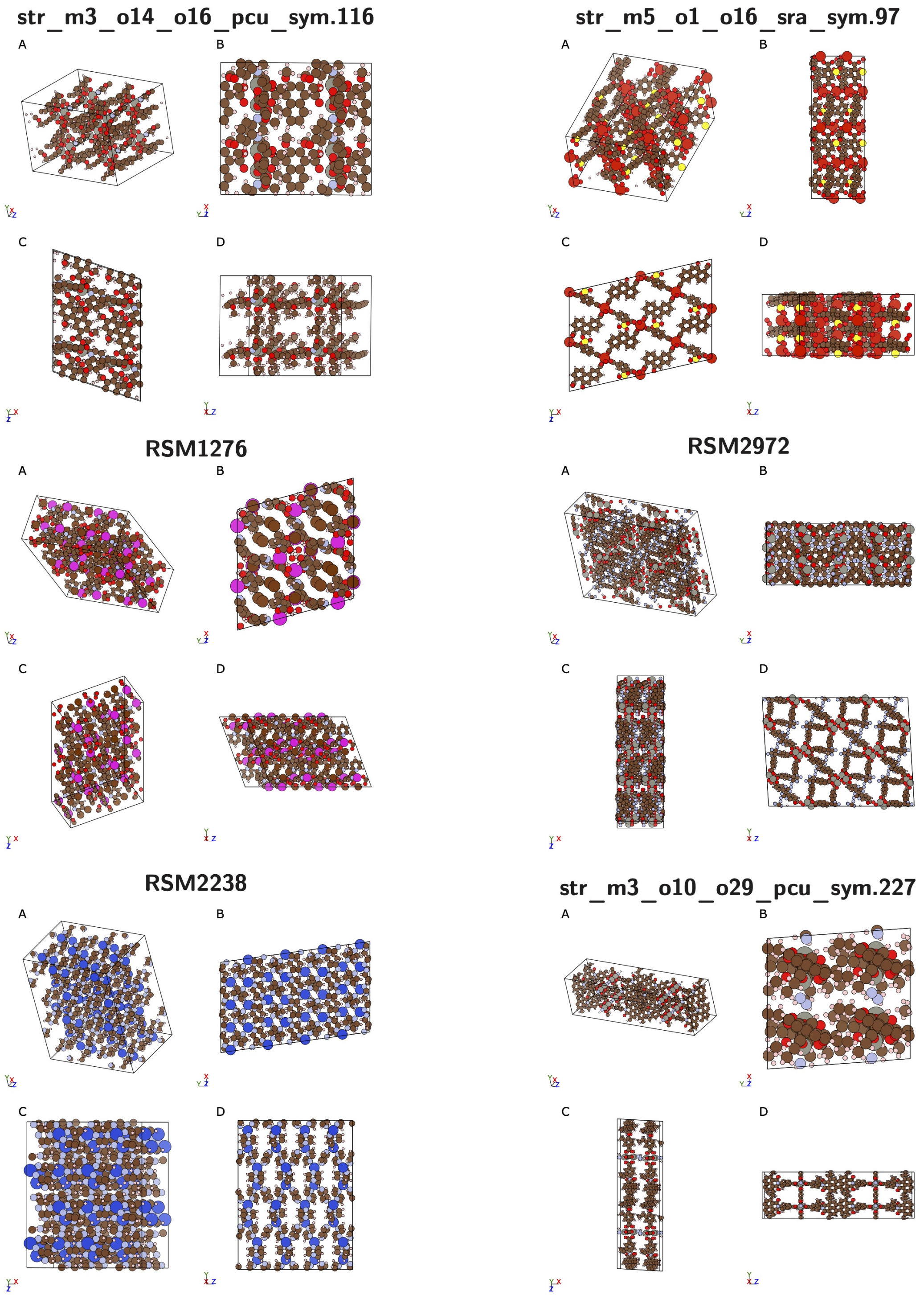

Figure S64| Prototypical structures for CAC better than MEA and good water resistance. `str_m3_o14_o16_pcu_sym.116` has a Zn-paddlewheel node with approx. 10 Å separation between stacked aromatic rings, `str_m5_o1_o16_sra_sym.97` has a vanadium node with sulfate groups on the linker and approx. 7 Å separation between aromatic rings, `RSM1276` is a Cd-structure with water coordinated on OMS and Br-functionalized linkers, `RSM2972` is a structure with OMS and CF_3 functionalized linkers and approx. 8 tstacked aromatic systems, `RSM2238` is a Cu-pyrazine compound, `str_m3_o10_o29_pcu_sym.227` is Zn-paddlewheel (bridged by N_2H_2 compound with large linkers with stacking distance of around 8 Å)

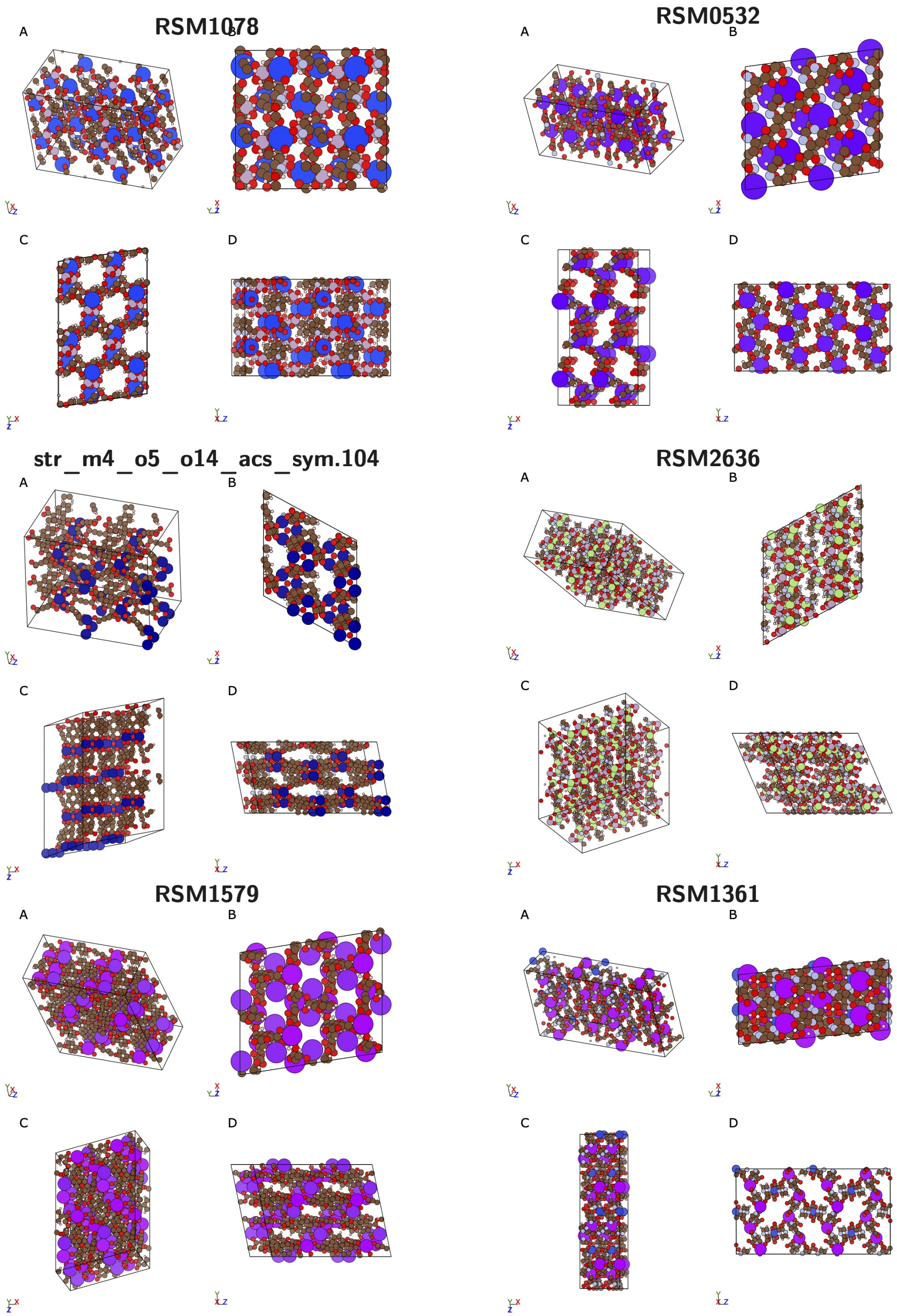

Figure S65| Prototypical structures for CAC better than MEA and bad water resistance. `RSM1078` is a Ho-compound with open coordination site at Ho, `RSM0532` is a Tb compound with an open coordination site at Tb, `str_m4_o5_o14_acs_sym.104` is a Zn compound with OMS, `RSM2636` is a Ga-phosphate compound with the only carbon being in a chelating diethylenetriamine ligand, `RSM1579` is K compound with strong open metal site, `RSM1361` is a Gd compound with OMS.

9 Impact of water

9.1 Direct impact of moisture on the capture performance

The impact of water on the capture performance can be quantified by comparing the ternary ($CO_2/N_2/H_2O$) separation and that for a hypothetical process without moisture which considers only CO_2 and N_2 in the feed gas stream.

In Figure S66, the variation of the nCAC, productivity, and required specific thermal energy for heating are plotted relative to the degree of moisture penetration into the column, α, for representative case studies with different feed CO_2 concentration levels. The data points are color-coded by the nCAC. Increased moisture penetration into the sorbent bed decreases productivity and increases the regeneration heat consumption. Overall, the presence of moisture entails a higher nCAC, with increasing α leading to more substantial increases in cost. For the cement case study, this increase is at least of 5.0 €$t^{-1}_{CO_2}$ (8%).

In Figure 2 and Figure S67, we have observed that the top-performing materials (i.e., lowest specific energy consumption and nCAC) are also those materials that have the highest productivity. It is interesting to focus on those materials for which the difference in productivity between the performance under wet and dry flue gasses is the smallest. One would expect that these materials would also be top-performing for wet flue gasses. However, these materials do not give us the lowest nCAC for cement plants Figure S66. One contributing reason is that the actual wet productivity of these materials remains too low to be competitive with other high-productivity materials, which are comparatively more susceptible to competitive co-adsorption. In the subsequent section, we show that relative losses in CO_2 capture productivity due to competitive co-adsorption by moisture are not especially significant at moderate feed CO_2 concentrations. On the other hand, structures that minimize the change in specific thermal energy for heating are among the best cost performers for a similar degree of water penetration. This implies that these materials can be more efficiently regenerated from water. For the NGCC case, the trends in the ΔKPIs are qualitatively

similar. However, at the lower feed CO_2 concentration, the impact of moisture is amplified in magnitude. Increases in nCAC and in the required specific thermal energy for heating range from 26.7 €$t_{CO_2}^{-1}$ (22.1%) and from 0.9 MJth per tonne CO_2 (27%), respectively, which is highly significant. This suggests a greater need to manage moisture at lower feed CO_2 partial pressures.

9.2 Impact of moisture on the material rankings

In Figure S68, we compare the materials ranking for nCAC for both wet ($nCAC_{wet}$) and dry flue gasses ($nCAC_{dry}$). In addition to the nCAC, we also show the ranking of some of the properties of these materials. We also highlight (with green lines) the materials that drop in ranking more than 100 places if we compare the wet nCAC with the dry one. Or (with yellow lines) increase in ranking if we compare the dry nCAC with the wet one. Here, we have to be careful as, in absolute terms, the wet nCAC is higher for all materials. Hence, the materials we discuss here have limited their increase.

As can be expected, the top-performing materials for dry flue gasses all have a high CO_2 working capacity under dry flue gas conditions ($WC_{CO_2,dry}$). If we look at their performance for wet flue gasses ($nCAC_{wet}$), we see that some of these materials remain in the group of top 10 performers (purple lines), while the rest are excluded (red lines) and are replaced by other materials (blue lines). As the feed CO_2 concentration decreases, fewer structures retain their top 10 ranking under wet conditions.

We also expect top-performing materials for wet CO_2 capture to have relatively high wet CO_2 working capacities ($WC_{CO_2,wet}$). For the cement and coal case studies, the materials with the highest ($WC_{CO_2,wet}$) are among those with the lowest nCAC under wet conditions. WC_{CO_2} is also a strong determinant for nCAC in the materials that significantly change in ranking when moisture is considered. The materials that lose places in the nCAC ranking due to moisture also lose places in ranking when WC_{CO_2} is considered, and vice versa. The

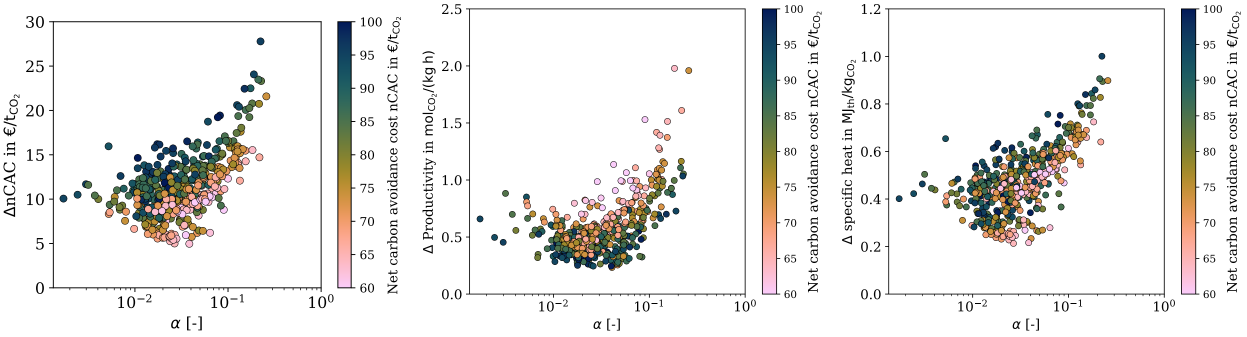

(a) Cement TVSA06

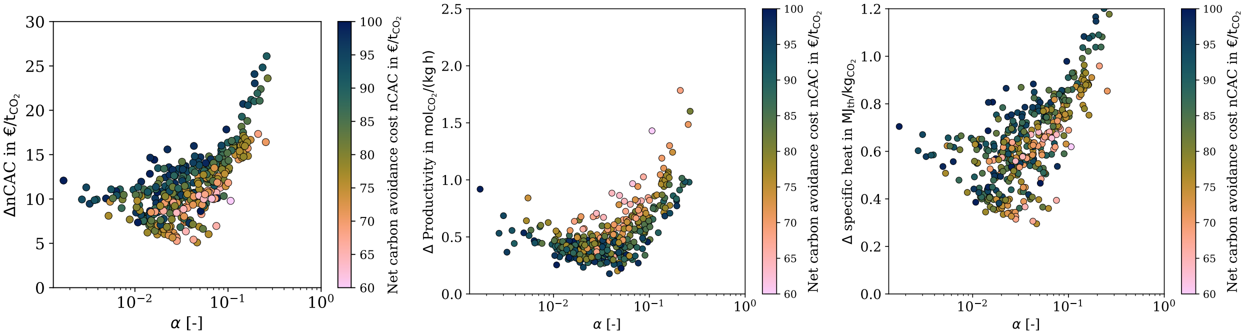

(b) Coal TVSA06

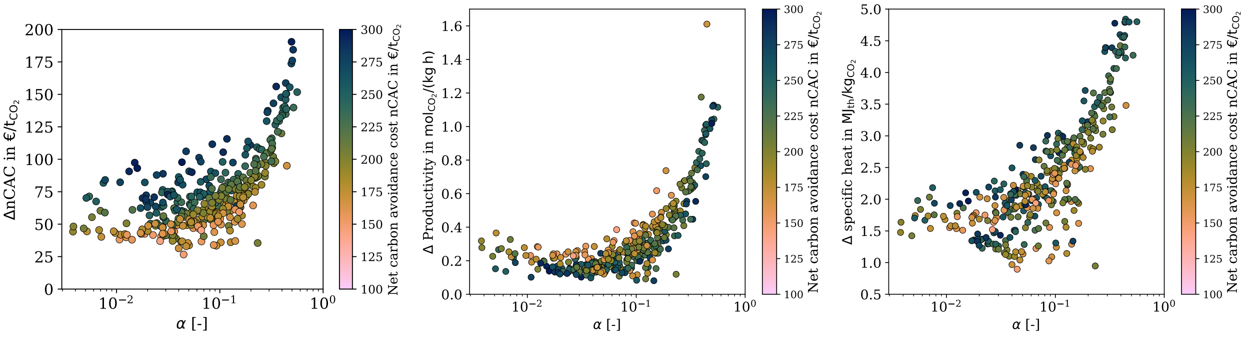

(c) NGCC TVSA02

Figure S66| Scatter plots of the increase in nCAC, decrease in CO_2 capture productivity, and increase in specific heat demand for sorbent regeneration due to the presence of moisture in the feed as a function of the fraction of bed that is moisture-loaded (α) for different configurations. All configurations are assumed to operate in the UK.

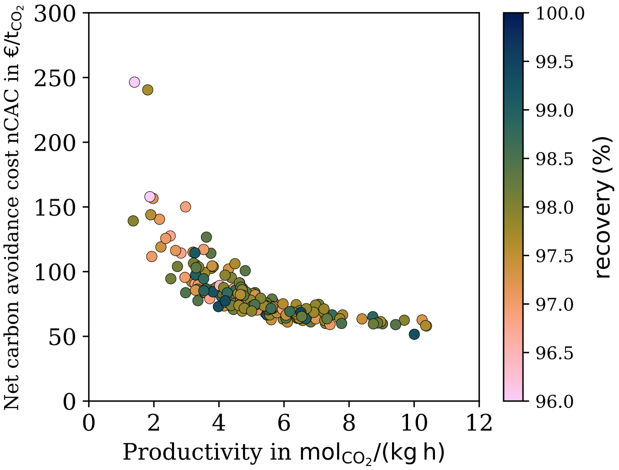

(a) Cement TVSA06

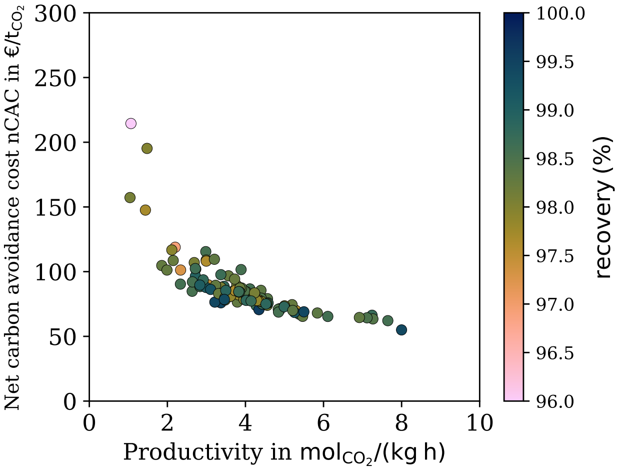

(b) Coal TVSA06

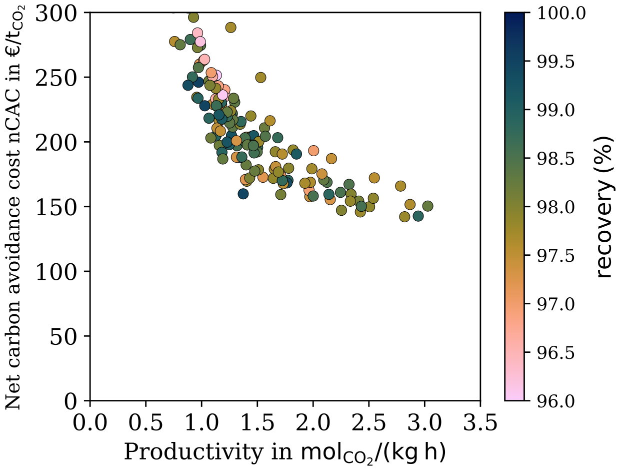

(c) NGCC TVSA02

Figure S67| nCAC versus productivity with color coding the recovery for different process configurations in the UK. Materials with purity less than 96% are not shown.

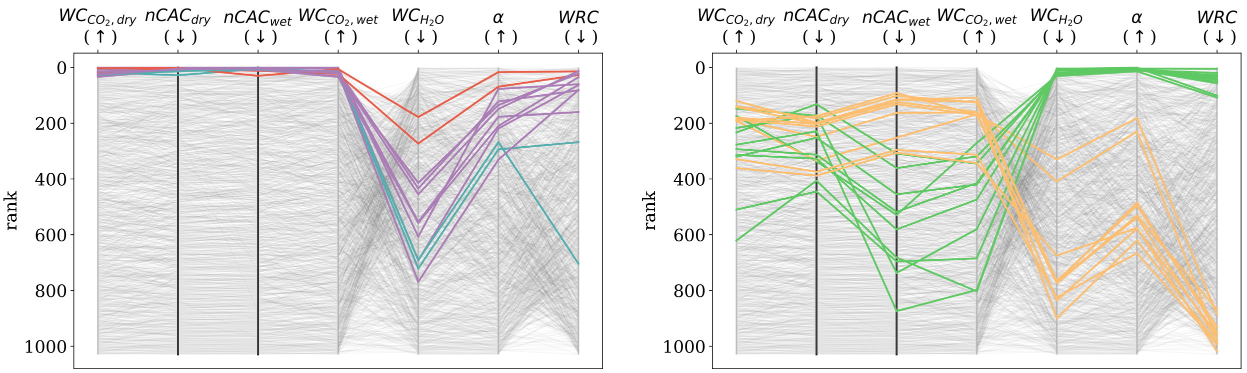

(a) Cement TVSA06

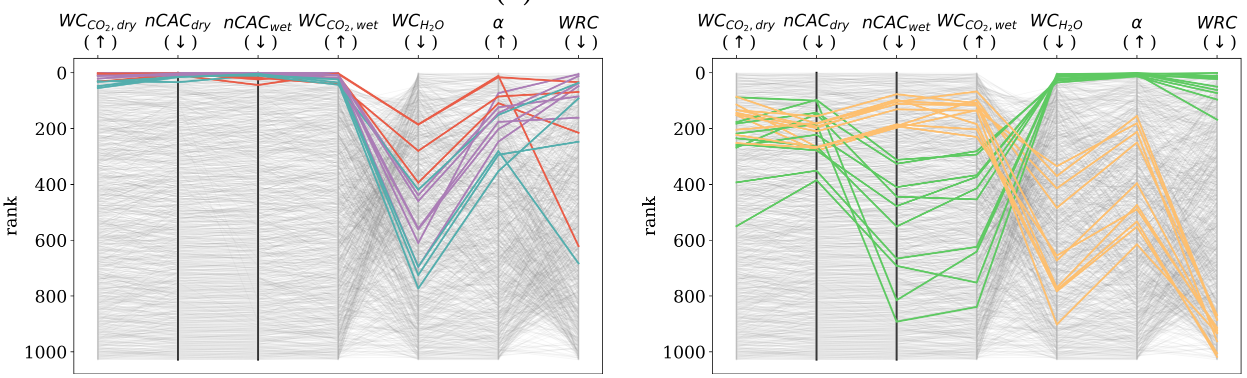

(b) Coal TVSA06

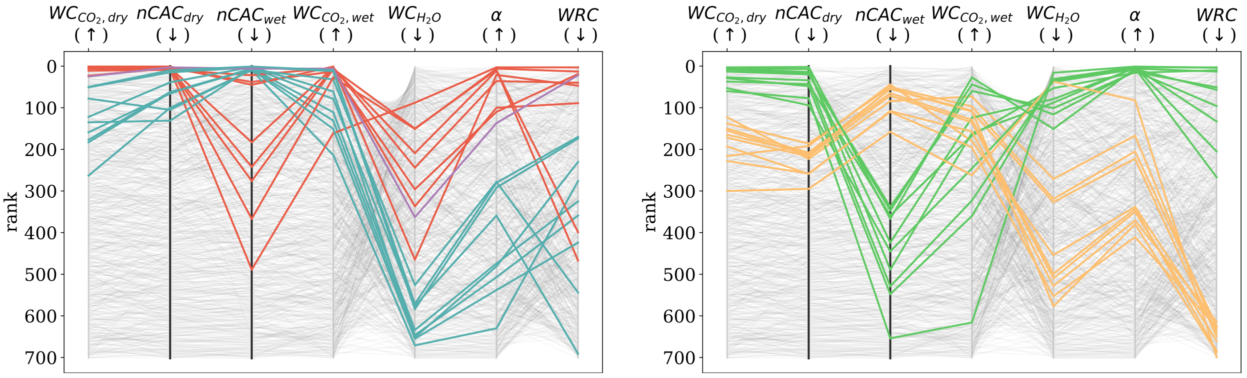

(c) NGCC TVSA02

Figure S68| Comparison of the ranking of materials for CO_2 capture from dry and wet flue gases. (left column) The top 10 materials ranked based on $nCAC_{dry}$ (red lines), $nCAC_{wet}$ (blue lines) are indicated. Materials ranked in the top 10 for both $nCAC_{dry}$ and $nCAC_{wet}$ are shown as purple lines. (right column) Materials that drop (green lines) or increase (yellow lines) in ranking more than 100 places, if we compare the wet nCAC with the dry one, are also presented.

materials that lose more performance have higher α and lower WRC, while the materials that gain rankings have higher WRC and moderate to high rankings for α.

A higher α implies the fraction of the wet column is higher, and in the wet part, the material loses capacity, as indicated by a low WRC. When WRC is high, the co-adsorption uptake for CO_2 remains at a similar level whether dry or wet operation, which makes α less significant.

On average, the materials with lowest $nCAC_{dry}$ have higher working capacities for H_2O, along with lower α, than the materials with lowest $nCAC_{wet}$. Note that WC_{H_2O} and α are inversely correlated (Refer to Section 6.2.4). Although the materials displaced in the ranking have higher α, they do not necessarily show inferior WRC or $WC_{CO_2,wet}$ to either the retained materials or the new top performers.

The abundance of low WRC materials among top nCAC performers in the cement and coal case studies also suggests that low CO_2-H_2O selectivity does not critically undermine performance when CO_2 feed concentrations are relatively high. This is because the extent of moisture penetration remains comparatively low (see Figure S69). Without prior desiccation, the humidity levels of the feed gas are near saturation, which leads to water adsorption uptakes considerably higher than those for CO_2. Under our chosen regeneration conditions, a non-negligible amount of sorbed water can be removed, and therefore WC_{CO_2} remains lower than WC_{H_2O}. For these case studies, the relative molar amount of moisture in the feed flue gas is $\approx$ 35 to 50% that of CO_2) (see Figure S69). The dominant contribution to α is the product of the ratio of feed concentrations and the ratio of working capacities for CO_2 and H_2O(see Section 6.2.4). α remains $<$ 10% of the column length for the majority of the screened structures (1091 out of 1185 materials evaluated). Therefore, a pronounced ranking improvement in α (leading to decreased moisture penetration) does not entail a significant CO_2 working capacity increase. This implies that materials with higher α may face additional cost disadvantages beyond the suppression of CO_2 uptake by moisture. For

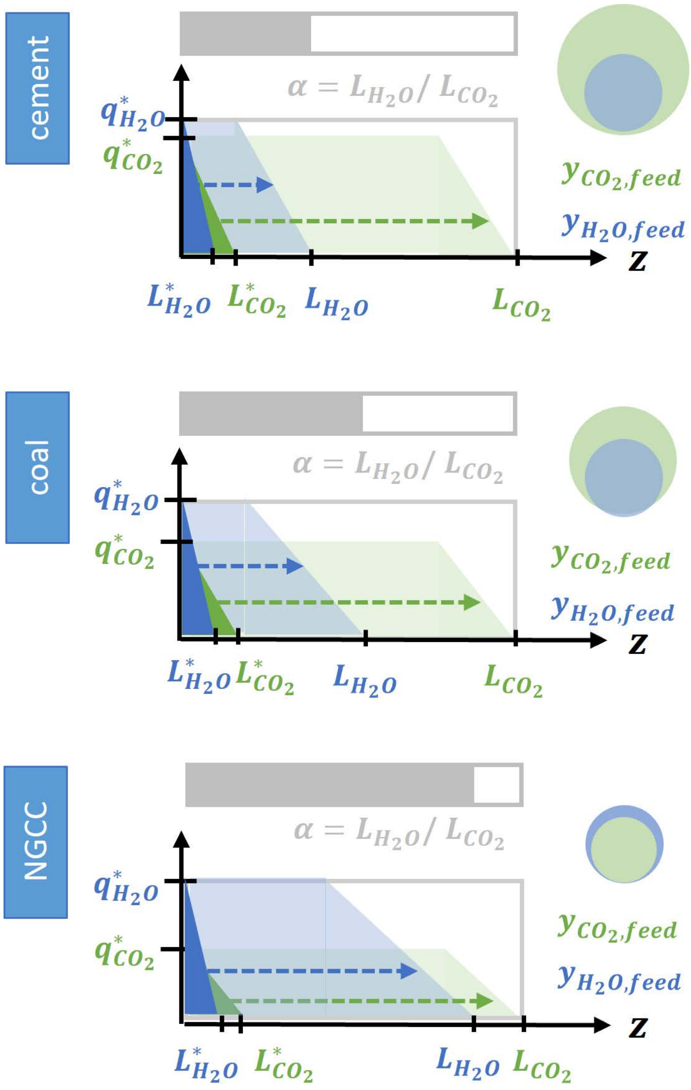

Figure S69| Illustration of the relative movement of concentration profiles in the solid during the adsorption step for different feed concentrations corresponding to the cement (top), coal (middle), and NGCC (bottom) case studies. $L^*_{\mathrm{H_2O}}$ and $L^*_{\mathrm{CO_2}}$ are the solid concentration profiles at the beginning of the feed adsorption step. $L_{\mathrm{H_2O}}$ and $L_{\mathrm{CO_2}}$ are the solid concentration profiles at the end of the feed adsorption step. Due to partial regeneration of the bed during cyclic operation, the residual solid concentrations are typically nonzero. $q^*_{\mathrm{H_2O}}$ and $q^*_{\mathrm{CO_2}}$ are theH_2O and CO_2 uptakes, respectively, of the sorbent when equilibrated with a gas mixture at the feed condition. (inset) Comparison of relative molar amounts of CO_2 and H_2O in the feed gas.

example, we have observed (in section 9.1) that materials with higher α experience increased additional energy costs for sorbent regeneration.

For NGCC case studies, the relative molar amount of moisture in the feed flue gas is 40% higher than its molar CO_2 content (see Figure S69). At the feed condition's low CO_2 partial pressures, the CO_2 equilibrium uptake reduces. Accordingly, the mean WC_{CO_2} decreases on the whole for the database and also for the top performers. A CO_2 working capacity decrease will counteract the impact of increasing relative moisture content in the feed on α. While α generally increases for individual materials, the number of materials for which α remains $<$ 10% of the column length remains significant (937 out of 1185 materials). Therefore, it remains feasible to identify a material that performs similarly otherwise but possesses a lower α. On average, for the top 10 performers, the mean α increases from 5.1% for the cement case study to 5.8% for the NGCC case study.

For the NGCC case study, there is very little overlap between top-performing materials under dry conditions (lowest $nCAC_{dry}$) and those under wet conditions (lowest $nCAC_{wet}$) Figure S68. The top performers for wet flue gasses have, in general, lower CO_2 working capacities, higher H_2O working capacities, and lower α. The top performers for NGCC also vary widely in WRC. However, compared to the cement and coal case studies, there is a trend towards higher WRC in the top 10. This preference appears to be related to the correlation of WRC and α, rather than any direct impact on $WC_{CO_2,wet}$ due to CO_2-H_2O competitive co-adsorption. The materials that show the largest losses (gains) in nCAC rankings for NGCC after considering moisture belong in clusters with high α, and low WRC. This is similar to the materials displaced from the top 10 performers (lowest $nCAC_{dry}$) under dry conditions.

Clustering top performers into groups with distinct α and WRC levels holds potential material design implications because the underlying phenomena driving such clusters are associated with specific structural motifs (see Section 8.5.3). Importantly, the performance of a particular group relative to the broader MOF space is case-dependent, with no assembly of features showing clear superiority. Nevertheless, the strong correlations of the cost perfor-

mance with respect to α and WRC highlight the importance of quantifying these properties even for rapid screening efforts.

9.3 Limits of the model

Two factors in our model can give "false positives." First, our estimate of the water penetration length (α) is too optimistic. The second factor is that the dry part of the column remains completely dry during cyclic operations. In this section, we discuss the impact of both factors.

9.3.1 Impact of mass transfer limitations on the wet performance

In our model, the CO_2 working capacity is calculated using the penetration length of the water concentration front as the weighting parameter (See Section 6.2.4), and under the assumption of ideal breakthrough behavior in the adsorption step, i.e., negligible mass transfer resistances.

Under non-ideal mass transfer conditions, the water penetration length will increase. Thus, more of the bed will be subject to competitive co-adsorption. Based on multi-component (binary CO_2/H_2O) dynamic column breakthrough experiments on zeolite 13X, Wilkins et al.[S57] observed up to 98% loading reductions at 74.4% relative humidity, which implies a low WRC of 0.02 at a CO_2 concentration of 15%.[S57] By combining the CO_2 equilibrium information with literature water equilibrium data,[S222] we can also estimate α values of 0.15 (coal/cement) to 0.30 (NGCC) for zeolite 13X, which is at the 96th percentile and 94th percentile of all materials in the PrISMa database, respectively.

To investigate the sensitivity of the results to mass transfer spreading within our model, we apply a conservative weighting parameter (three times the penetration length) and recalculate the platform KPIs for comparison (see Figure S70). By using such a high proportionality constant, we can reproduce semi-quantitatively the productivity reductions observed in literature-reported cyclic wet experiments ($\approx$ 22% at lower feed moisture concentrations).[S56] Based on the indicative values for WRC and α, our choice of proportionality constant implies productivity loss of nearly 90% for wet NGCC capture by the zeolite 13X process due to uptake suppression by moisture.

Mass transfer limitations impact performance disproportionately, with materials with high α and low WRC being more affected (see previous section). Overall, working capacity reductions of $\approx$ 20 to 25% are observed at the different feed CO_2 concentrations. In comparison, the nCAC increases between 4 to 16%. The minimum nCAC increases for cement from 51.6 euros to 57.7 per tonne (+12%). The highest working capacity within the top 10 performers decreases from 3.96 to 3.18 mol kg $^{-1}$ ($-$20%). For coal, the minimum nCAC increases from 55.0 euros to 63.8 per tonne (+16%). The highest working capacity within the top 10 performers decreases from 3.55 to 2.70 mol kg $^{-1}$ ($-$24%). For NGCC, the minimum nCAC increases from 142.1 to 147.7 euros per tonne (+4%). The highest working capacity within the top 10 performers decreases 0.98 to 0.78 mol kg^{-1} ($-$20%).

Under non-ideal mass transfer conditions, the average α for the top 10 performers with respect to $nCAC_{wet}$ decreases from 5.1 to 4.2% for cement, 5.5 to 4.5% for coal, and 5.8 to 2.8% for NGCC. For cement and coal case studies, 7 out of the top 10 performers are retained in the new top 10 rankings, with the rest ranking within the top 20 performers. For the NGCC case study, 6 out of the top 10 performers are retained in the new top 10 performers after consideration of mass transfer limitations.

9.3.2 Impact of moisture slippage

Models for adsorption processes generally assume axially-dispersed plug flow.[S223,S224] This assumption permits the separation of the adsorber column into dry and wet zones during cyclic operation because the mixing of the gas phase between different zones is substantially limited. Yet, one cannot exclude that some water will permeate the dry zone during the cyclic operation of the column due to hydrodynamic non-idealities such as near-wall channeling.[S225] One can mitigate these effects by removing the accumulated water in the dry zone after extended periods of operation, as discussed in some patent literature.[S226–S230] The frequency of this drying procedure will depend on the material. In our model, the cost of this drying step has not been included. Therefore, it is important to exclude those materials from our

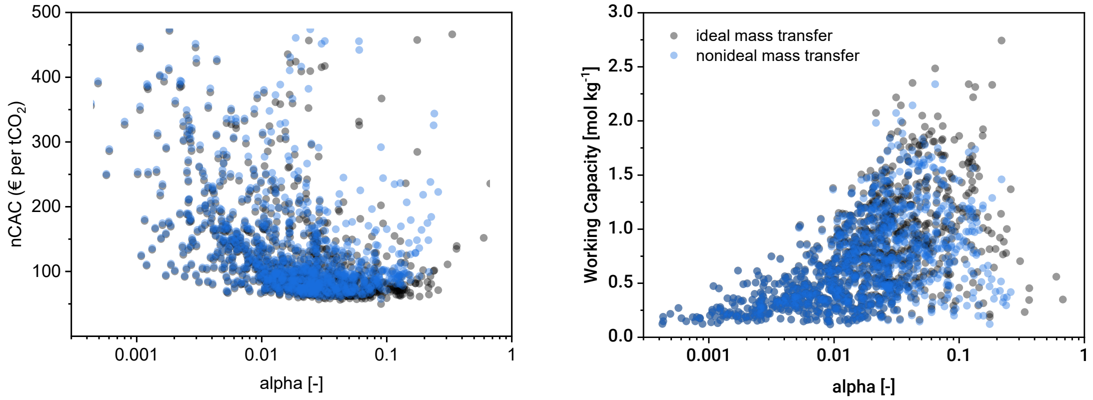

(a) Cement TVSA06

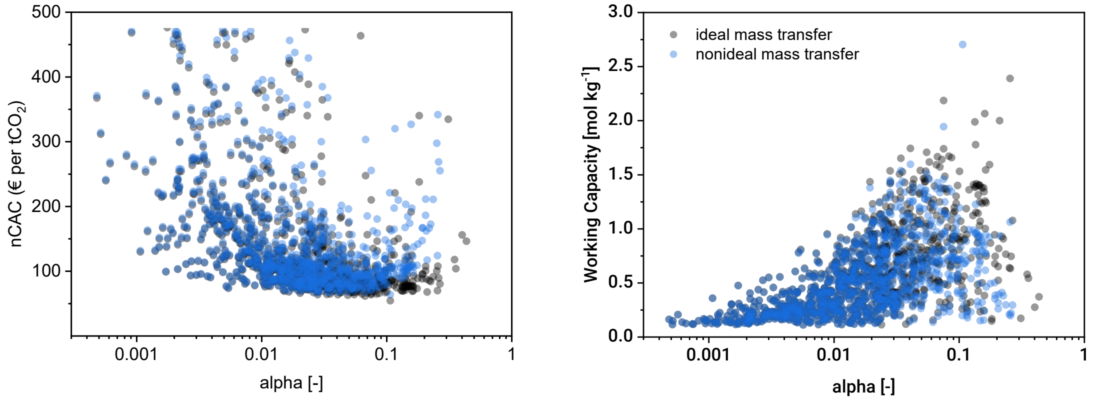

(b) Coal TVSA06

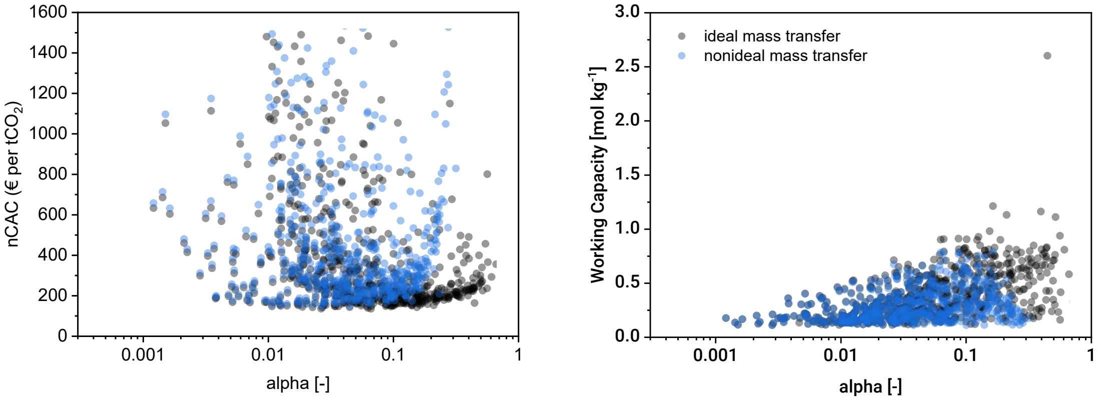

(c) NGCC TVSA02

Figure S70| Comparison of the nCAC (left) and CO_2 working capacities (right) as a function of α, in the absence (grey symbols), or presence (blue symbols), of mass transfer limitations. The effect of mass transfer limitations is modeled by modifying the weighting parameter with a proportionality constant of 3 during the calculation of wet co-adsorption uptake of CO_2.

screening for which we expect a significant loss in performance due to the moisture ingress and for which the nCAC increases significantly due to the need for such additional drying.

We assume a worst-case scenario to assess the effect of moisture slipping through to the dry zone. The maximum amount of water that can accumulate in the dry zone is equal to the water content of the wet zone after the regeneration step. We can now have the following system, assuming the "dry" zone has this maximum water content. The difference with the wet zone is that the water content will not increase during the adsorption step. From a modeling point of view, this implies that we need to compute the pure component isotherms in a material with "permanent" water present. Experimentally, it is impossible to measure such an isotherm. However, in a molecular simulation, this can be done straightforwardly. We compute the CO_2 and N_2 isotherms in a material with water molecules. In these simulations, the water molecules can move in the material, but no Monte Carlo moves will be applied that increase or decrease the number of water molecules.

To quantify these effects, we compare the performance of CALF-20 and zeolite 13X for the cement reference Case Study. In Figure S71, we compare the CO_2 isotherms of the completely dry materials and the water "contaminated" dry materials. We see a marked difference between the two sorbents. Water-contaminated zeolite 13X has almost completely lost its capacity to capture CO_2, while for CALF-20, we see only a small impact.

We can then simply estimate the Water resistance coefficient (WRC) of the water-contaminated materials. The WRC assumes that water is displacing $(1 - \text{WRC}) \times 100\%$ of the CO_2 molecules from the adsorption sites at adsorption conditions. As the water content is lower at desorption conditions, we assume that at these conditions, water is displacing a corresponding fraction. This gives us a value of the Water resistance coefficient in a water-contaminated material (WRC*) that we can use to correct the adsorption capacity of the dry zone for water contamination:

$$(1 - \text{WRC}^*) = \frac{q_{\text{water, desor}}}{q_{\text{water, adsor}}}(1 - \text{WRC}), \tag{S73}$$

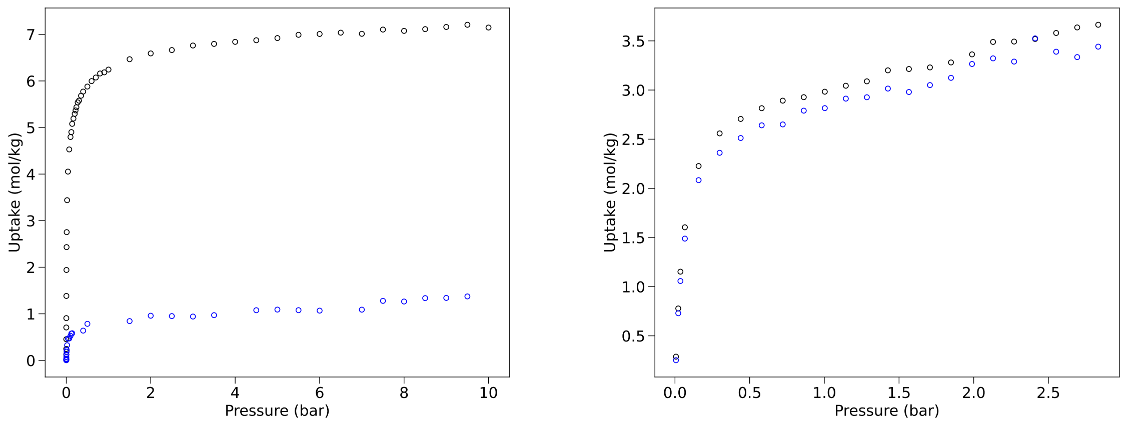

(a) 13X **(b)** CALF-20

Figure S71| Comparison of the CO_2 isotherms in zeolite 13X (a) and CALF-20 (b) in dry conditions (black circles) and after water "contamination" in dry conditions (blue circles).

where $q_{\text{water, desor}}$ is the water content after the desorption step (cf. Section 3.1.2) and $q_{\text{water, adsor}}$ is the water content after the adsorption step (cf. Equation (S43)) The ratio $q_{\text{water, desor}}/ q_{\text{water, adsor}}$ depends on the adsorption-desorption conditions and is computed in the process layer. We do not expect this linear relation to hold for materials with a very low WRC; for these materials, the actual WRC* will be much lower.

In Figure S72a, we show the WRC as a function of $q_{\text{water, desor}}/q_{\text{water, adsor}}$. Materials that have a strong affinity for water typically have a large $q_{\text{water, desor}}/q_{\text{water, adsor}}$ and a low WRC. Figure S72b shows the WRC* versus WRC. In both graphs, we have indicated some materials (Zeolite 13X, Mg-MOF-74, CALF-20, and MIP-212) that are of interest for the discussion. Zeolite 13X and Mg-MOF-74 have a strong affinity for water because of the cations and Open metal site, respectively.[S183,S231] For those materials, the water affinity is so strong that moisture slippage significantly undermines the capacity of the sorbent. Therefore, the viability of wet gas separation rests heavily on the plug-flow assumption made for the fluid-flow behavior. In contrast, for CALF-20 and MIP-212, water contamination is far less of a problem. Interestingly, for different reasons. CALF-20 has a relatively low WRC compared to MIP-212. However, at the desorption conditions, the amount of water that remains in

CALF-20 is lower (2%) than for MIP-212 (8%), which compensates for CALF-20's lower WRC.

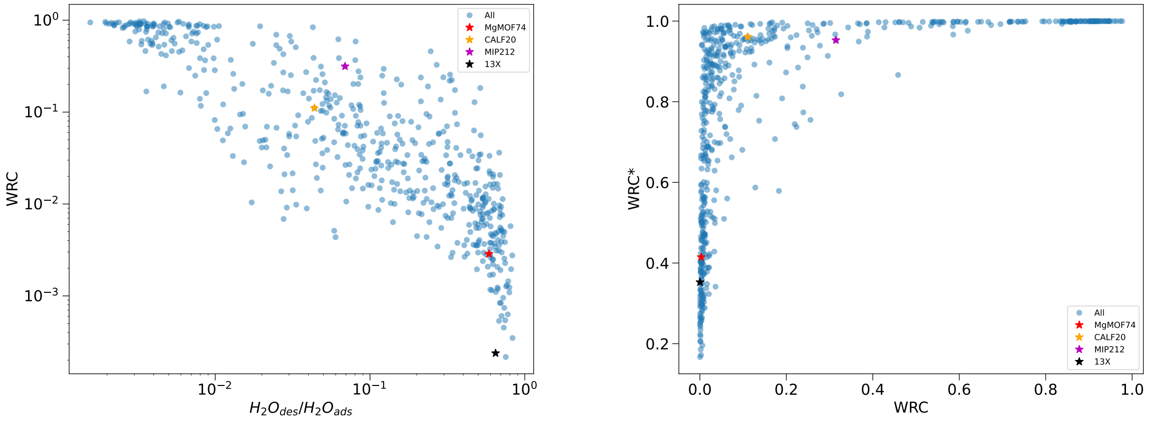

(a) **(b)**

Figure S72| The effect of water on the adsorption capacity (a) the Water resistance coefficient (WRC) as a function of $q_{\text{water, desor}}/q_{\text{water, adsor}}$ and (b) the Water resistance coefficient in a water-contaminated material (WRC*) as a function of the WRC.

If moisture slipping through to the dry zone causes a significant decrease in the material's performance, a practical solution is to introduce a drying step after a certain number of cycles. In such a drying step, water is removed from the dry part of the column. The frequency and intensity (e.g., temperature and amount of desorbing inert gas) of this drying step will depend on the material. In our model, we have not costed such a drying step. In any case, such a drying step will only be carried out if it results in a lower nCAC than not using a drying step. This allows us to make a worst-case estimate of the increase in nCAC caused by water contamination. This worst case is presented in Figure S73. This figure shows that there are a number of materials for which this drying step can be avoided (green materials). In addition, there are materials that, without a drying step, perform worse than the MEA benchmark (red materials).

Let us focus on the materials highlighted in Figure S72. The nCAC of "water-contaminated" CALF-20 and MIP-212 is about $2\,€t_{CO_2}^{-1}$ and $3\,€t_{CO_2}^{-1}$ higher compared to their completely

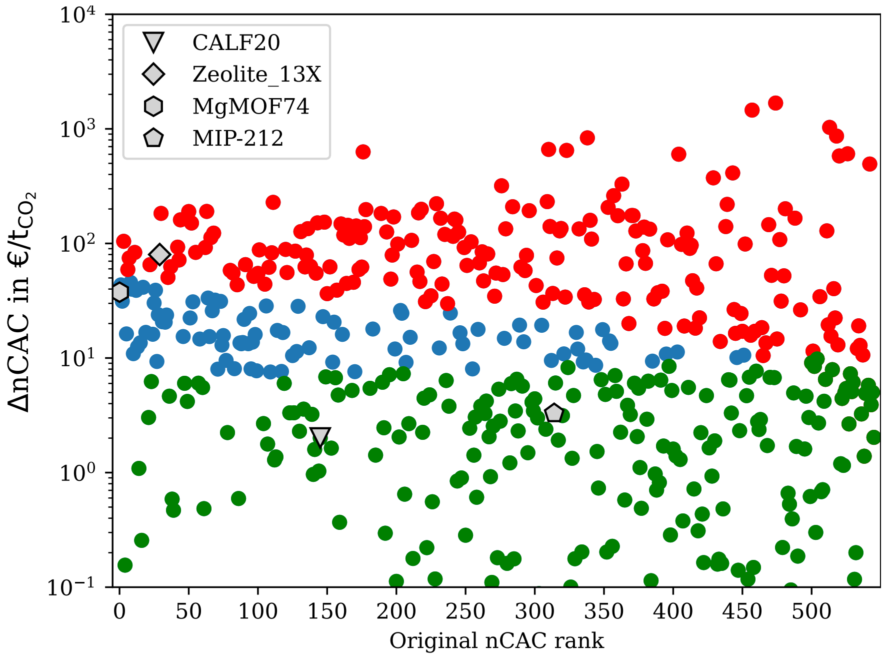

Figure S73| The effect of water contamination on the nCAC Worst-case estimate of the increase in nCAC caused by water contamination. The green dots represent materials for which the maximum increase is less than 10%, and for the red materials, the contamination has such a large effect that the expected nCAC is above that of MEA. The blue materials are in between these two thresholds. The materials highlighted in Figure S72a and Figure S72 are also shown as grey symbols.

dry structures, respectively. In contrast, “water-contamination” increases the nCAC for zeolite 13X and Mg-MOF-74 by $\approx 80\,€t_{CO_2}^{-1}$ and $37\,€t_{CO_2}^{-1}$, respectively. This makes the nCAC of Mg-MOF-74, which was previously a top-performing material, drop 100 positions in ranking and makes zeolite 13X more expensive than the MEA benchmark.

10 Process optimization

In our screening studies, we use a set of given process and process conditions in the PrISMa platform, and we screen for the optimal materials for three process configurations (i.e., TSA and TVSA at 0.6 bar and 0.2 bar). In this section, we investigate if further optimization of the process will result in a significantly lower Net Carbon Avoidance Cost (nCAC) and will change the ranking of the materials.

We first perform a sensitivity analysis of the model parameters to identify degrees of freedom with strong impact on the Net Carbon Avoidance Cost (nCAC) (Section 10.1). The resulting optimization problem is introduced in Section 10.2. The results of the process optimization are shown in Section 10.3.

10.1 Tornado plot analysis

We perform tornado analysis to identify and prioritize the most significant factors that can impact the Net Carbon Avoidance Cost (nCAC). In this analysis, we change those variables and parameters related to the operation of the process, the design of the process, the economics, and the life cycle assessment.

We have not included the material properties in the Tornado analysis. The only way we change the material properties is to select a different material. In Section 5, we present a separate analysis on how errors in predicting the material properties propagate in the platform are important.

Figure S74 shows the Tornado plot if we make a 10 % change of all relevant variables for Mg-MOF-74 in a TVSA process with 0.6 bar vacuum to capture CO_2 from a cement plant. Mg-MOF-74 is among the top-performing MOFs.

Below, we list the variables, in decreasing order of importance, that are presented in the Tornado plot. The value between brackets is the reference value we used in the calculation.

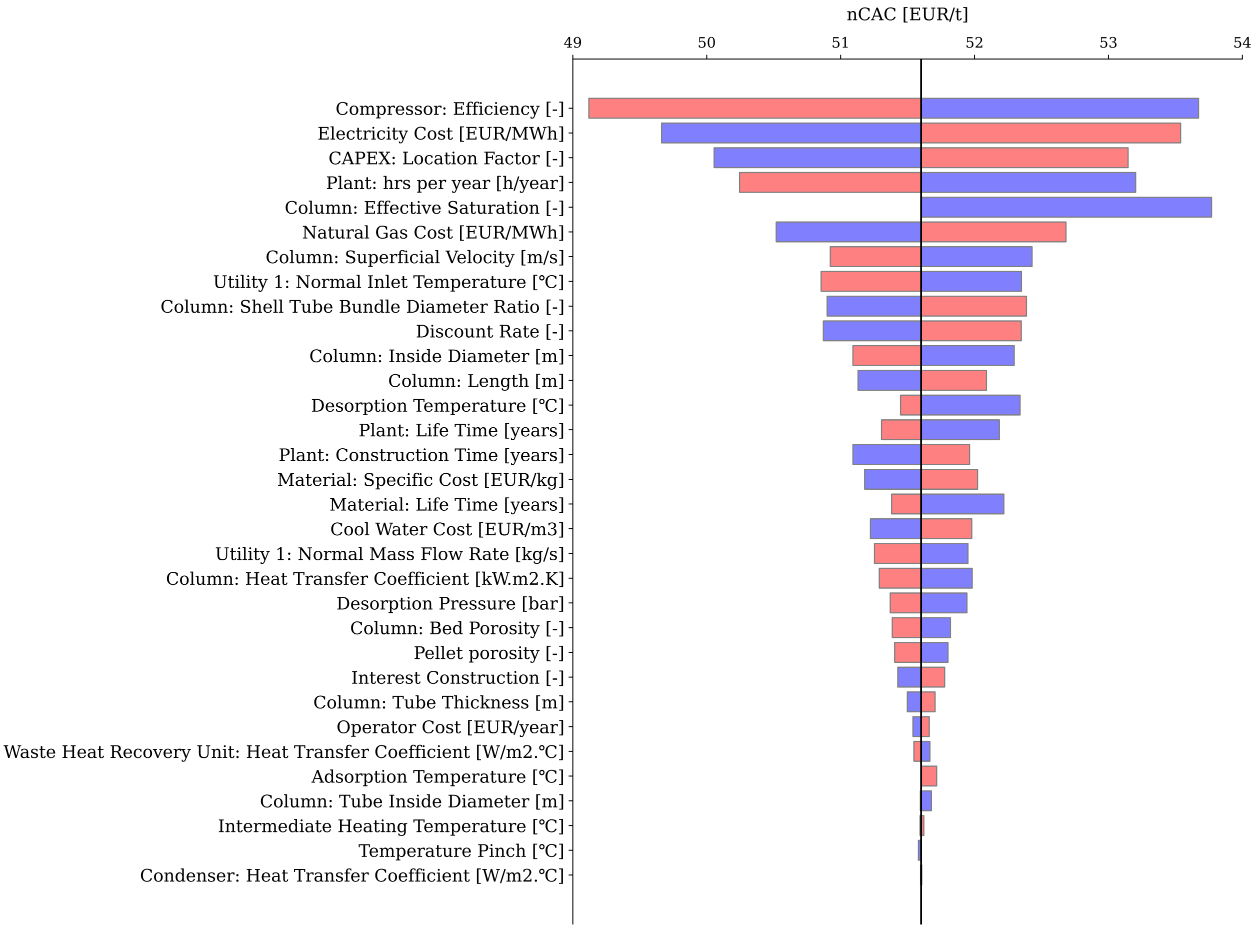

Figure S74| Tornado plot for the parameters of all layers We computed the change in nCAC caused by a ± 10% change in the parameters. The parameters are ordered in decreasing impact on the nCAC. A red bar indicates a positive change in the parameter, a blue bar indicates a negative change, and a purple bar indicates if both a positive and a negative change have the same effect. The absence of a bar indicates that the change considered results in a calculation outside the assumed ranges and is not calculated. The LCA parameters only have a minor impact. Therefore, these results are not shown.

For this material, we found that the ± 10% change in the LCA parameters has a minor impact. Therefore, these variables are not further discussed here.

Compressor efficiency (TEA layer, η_p =0.86 [−]) Gives the polytropic efficiency of the compressor for geological storage.

Electricity Cost (TEA layer, 131.7 € $\mathrm{MWh^{-1}}$) The UK electricity cost of the grid at a commercial rate.

CAPEX: Location Factor (TEA layer, 1 [−]) The location factor is used to multiply the Total Direct Cost to consider the differences between countries (see Table S2).

Plant: hrs per year (TEA layer, 7997.88 $\mathrm{h\,yr^{-1}}$) Operating hours per year for approximately 91.3% capacity factor.

Column: Effective Saturation (Process layer, 1 [−]) Ratio of the actual adsorbed amount to the maximum adsorption capacity of the column at the end of the adsorption step.

Natural Gas Cost (TEA layer, 24.63 € $\mathrm{MWh^{-1}}$) Natural gas cost in the UK in 2019.

Column: Superficial Velocity (Process layer, 1.75 $\mathrm{m\,s^{-1}}$) Superficial velocity of the flue gas passing through the column.

Utility 1: Normal Inlet Temperature (TEA layer, 285 °C) Temperature of the waste heat stream to be recovered at the host cement plant.

Column: Shell Tube Bundle Diameter Ratio (TEA layer, 1.01 [−]) Ratio of the shell diameter to tube bundle diameter.

Discount Rate (TEA layer, 0.08 [−]) Discounted cash flow rate.

Column: Inside Diameter (TEA layer, 6.15 m) Inside diameter of the column shell.

Column: Length (Process layer, 8 m) Length of the column.

Desorption Temperature (Process layer, T_{HIGH} =120 °C) is the temperature at which we carry out the desorption step (see Section 3.2.1).

Plant: Life Time (TEA layer, 25 yr) Lifetime of reference plant.

Plant: Construction Time (TEA layer, 3 yr) Construction periods for reference plant.

Material: Specific Cost (TEA layer, 30 € kg^{-1}) Specific cost of the material.

Material: Life Time (TEA layer, 5 yr) Lifetime of the material, i.e., the interval that the material needs to be replaced.

Cool Water Cost (TEA layer, 2 € m^{-3}) Cost of drinking water.

Utility 1: Normal Mass Flow Rate (TEA layer, 46.2 $\text{kg}\,\text{s}^{-1}$) Mass flow rate of the waste heat stream to be recovered at the host cement plant.

Column: Heat Transfer Coefficient (Process layer, 0.04 $\text{kW}\,\text{m}^2\,\text{K}$) Heat transfer coefficient from the heating or cooling medium to the column.

Desorption Pressure (Process layer, P_{vac} =0.6 bar) is the pressure at which we carry out the desorption step (see Section 3.2.1 and Section 3.2.1).

Column: Bed Porosity (Process layer, 0.37 [−]) Ratio of the volume of void spaces between particles to the total volume of the bed.

Pellet porosity (Process layer, 0.35 [−]) Fraction of macropore void spaces within an adsorbent pellet.

Interest Construction (TEA layer, 0.08 [−] Interest rate during construction.

Column: Tube Thickness (TEA layer, 0.0021 m) Thickness of the tube wall in which the material is packed.

Operator Cost (TEA layer, 61 180 € yr^{-1}) Annual wage of an operator.

Waste Heat Recovery Unit: Heat Transfer Coefficient (TEA layer, $65\,\mathrm{W\,m^{-2}\,°C^{-1}}$)
Heat transfer coefficient of the waste heat recovery unit.

Adsorption Temperature (Process layer, 37 °C) Temperature at which we carry out the adsorption step (see Section 3.2.1).

Column: Tube Inside Diameter (TEA layer, 0.025 m) Inside diameter of the tube in which the material is packed.

Intermediate Heating Temperature (Process layer, T_{MED} =47 °C) is the pressure at which we carry out the intermediate heating step (see Section 3.2.1).

Temperature Pinch (TEA layer, 7.5 °C) Minimum temperature difference between hot and cold streams at any point in the heat exchanger.

Condenser: Heat Transfer Coefficient (TEA layer, $600\,\mathrm{W\,m^{-2}\,°C^{-1}}$) Heat transfer coefficient of the condenser.

Figure S74 ranks the parameters in decreasing effect on the nCAC. Most variables have a red and a blue bar. However, for some parameters, such as *Column: Effective Saturation* and *Adsorption Temperature*, we can only decrease or increase its value (e.g., an effective saturation greater than 1 is infeasible), or it is outside the (physical) limits (e.g., an adsorption temperature lower than the minimum temperature cooling water can supply). For those cases, we only see one bar.

The most notable finding from the tornado plot is that a 10 % change in the specific cost of MOF only leads to a minor change in nCAC (less than $1\,€\mathrm{t}^{-1}_{\mathrm{CO_2}}$). The maximum effect we observe on the nCAC is a change of about 5%. That we do not see that a small change in one variable has an exponential effect on the nCAC does give us some confidence in the robustness of the platform.

The *Compressor efficiency* has the largest impact in this case. This is due to the large power consumption of the compressors. This compression is needed for geological storage.

A 10 % increase/decrease in polytropic efficiency will lead to a substantial change in the compression work (S26). The compressor we have selected is state-of-the-art in industrial compressors. Any gains/losses in efficiency will affect both the MEA and our process.

The *Electricity Cost* is the second-highest ranked parameter, which underscores that the total power consumption contributes to the overall cost considerably. Two parameters associated with the plant's location and operation, *CAPEX: Location Factor* and *Plant: hrs per year* rank third and fourth, respectively. In Section 1, more details are given about the sources from which the values of these four parameters were obtained. The tornado plot shows the importance of monitoring changes in these (external) parameters.

The first TVSA process-related parameter is the *Column: Effective Saturation*, which holds the fifth position. This shows the potential impact of mass transfer resistances, which are not considered in the current thermodynamic model. In Section 9.3.1, we analyze the impact of mass transfer in more detail.

For the optimization study, we focus on the most important design variables that we have fixed in our screening study, but that would typically be optimized in a real process design. To limit the number of variables in our optimization step, we focus on the most important ones related to the process conditions (e.g., desorption temperature and pressure), column specifications (e.g., column inside diameter and length), and utility limits (e.g., temperature pinch).

For the considered case study, the impact of the parameters from the LCA layer is far below that of those from the process and TEA layer. Hence, these parameters are not shown in Figure S74. For the cement case, the environmental impacts are dominated by the use phase. This high impact from the use phase is particularly dominant for good-performing materials like Mg-MOF-74 because less material is required. Please note that the impact of the parameters from the synthesis and disposal phase can change for other case studies, materials, or environmental impact categories.

In 8.4.3, we give a more detailed analysis of the impact of a more substantial change in the cost of MOF and the impact of different MOF replacement rates.

10.2 Problem formulation

The process optimization of the CO_2 capture process, can be formulated as a Mixed-Integer Nonlinear Program (MINLP) problem:

$$\begin{aligned} \min_{x} \quad & f(x) \\ \text{s.t.} \quad & h(x) = 0 && \rightarrow \text{PrISMa model} \\ & g(x) \leq 0 && \rightarrow \text{Process constraints} \\ & x_{\text{lb}} \leq x \leq x_{\text{ub}} \in \mathbb{R}^{\text{n}} \times \mathbb{Z}^{\text{m}} \end{aligned} \tag{S74}$$

In this optimization problem, a process-related objective function $f(x)$ (e.g., the Net Carbon Avoidance Cost (nCAC)) is optimized. For our PrISMa platform, the objective function can be any KPI from the process, TEA, or LCA layers. The objective function depends on the process degrees of freedom x, which can be continuous variables (e.g., pressure or temperature levels) or discrete variables (e.g., the number of tubes in the column). If solely continuous degrees of freedom are considered, the problem reduces to a Nonlinear Program (NLP) problem. The objective function $f(x)$ is minimized subject to equality constraints $h(x)$ representing the entire model of the PrISMa platform and inequality constraints $g(x)$. The inequality constraints can be limits on degrees of freedom (e.g., $P_{\text{vac}} \leq P_{\text{ads}}$) or on KPIs (e.g., the minimal purity or recovery that has to be achieved). As a result, the optimization problem identifies the optimal process degrees of freedom x that minimize the objective function $f(x)$ for a specific material.

This work considers the Net Carbon Avoidance Cost as the objective function of the process optimization. From the tornado analysis (Section 10.1), we select 7 degrees of freedom for TSA processes and 9 degrees of freedom for the TVSA process as given in Table S9. As all degrees of freedom are continuous variables, the entire optimization problem reduces to an NLP problem. The initial guesses, the lower bounds, and the upper bounds of the degrees

of freedom are given in Table S9. The initial guesses are the parameters considered within the screenings.

The upper bound of the desorption temperature T_{high} and the lower bound of the adsorption temperature T_{low} are limited by the heating/cooling utility and the pinch temperature. The lower bound of T_{high} is selected by considering 90 % of the base value. Since there is no benefit in heating the flue gas stream, we fix the upper bound of T_{low} as the base value. To ensure realistic industrial operations, we set the lower bound of the vacuum pressure P_{vac} to be 0.1 bar.

The lower bound of the adsorption temperature T_{low} depends on the water cooling system design, which has a lower limit design temperature of 19.0 °C). The temperature range considered for the water cooling system is from 5.0 °C) to 10.0 °C) and the temperature pinch range is from 3.0 °C) to 7.5 °C). These ranges are reasonable within the design of heat exchange systems and aim to show the gains of having an improved exchange design that maximizes heat transfer. The lower bound of the adsorption temperature T_{low}, assuming co-current contact in the adsorption contactor, is set to 27.5 °C) to account for the temperature increase in the water cooling system and the temperature pinch. The upper bound of the desorption temperature T_{high} depends on the design pressure of the steam supply to the capture plant, which is set to 5.0 bar. This pressure ensures that the desorption temperature T_{high} can supply 150.0 °C after considering temperature losses and heat exchange. The rationale for the temperature pinch is the same as for the adsorption temperature. The temperature losses are set to a constant value of 3.0 °C following the EBTF guidelines.[S75] With these considerations, the upper bound for the desorption temperature $T_{\text{high}}$is set to 150.0 °C, which is reasonable for materials stability.

To ensure high purity and recovery of the resulting carbon capture processes, we limit both KPIs as inequality constraints of the optimization:

$$\text{Purity} \geq 96\,\% \tag{S75}$$

$$\text{Recovery} \geq 90\,\% \tag{S76}$$

The purity limit is defined by the requirements of the CO_2 sink, in this case, a CO_2 storage side. To ensure feasible heat transfer, we consider additional inequality constraints on the temperature levels with 0.05 K as extra margin to prevent numerical issues at the boundaries:

$$T_{\text{low}} - \Delta T_{\text{pinch}} - T_{\text{cool,lim}} \geq 0.05\,\text{K} \tag{S77}$$

and

$$T_{\text{heat,lim}} - T_{\text{loss}} - \Delta T_{\text{pinch}} - T_{\text{high}} \geq 0.05\,\text{K} \tag{S78}$$

with $T_{\text{heat,lim}} = T_{\text{sat}}(5\,\text{bar}) \approx 151.8\,^{\circ}\text{C}$ and $T_{\text{loss}} = 3\,\text{K}$ as defined in the TEA layer. The other variables are described in Table S9. Moreover, for TVSA processes, we additionally constrain the temperature difference between the intermediate temperature T_{med} and adsorption temperature T_{low} to be larger than 5 K:

$$T_{\text{med}} - T_{\text{low}} \geq 5\,\text{K}. \tag{S79}$$

The optimization problem is solved using the local optimization solver KNITRO[S232] and the Sequential Quadratic Programming algorithm[S233].

Table S9: Overview of the selected process degrees of freedom x, initial guesses x_{init}, lower bounds x_{lb}, and upper bounds x_{ub}.

Degree of freedom	Type	Initial guess	Lower bound	Upper bound
Desorption temperature T_{high}	continuous	120 °C	108 °C	150 °C
Adsorption temperature T_{low}	continuous	37 °C	27.5 °C	37 °C
Column inside diameter d_{col}	continuous	6.15 m	6 m	10 m
Tube inside diameter d_{tube}	continuous	0.025 m	0.025 m	0.1 m
Column length l_{col}	continuous	8 m	5 m	9 m
Temperature Pinch ΔT_{pinch}	continuous	7.5 °C	3 °C	7.5 °C
Limiting cooling temperature $T_{\text{cool,lim}}$	continuous	29 °C	24 °C	29 °C
Solely for TVSA				
Vacuum pressure P_{vac}	continuous	1.013 25 bar	0.1 bar	1.013 25 bar
Intermediate temperature T_{med}	continuous	47 °C	32.5 °C	65 °C

10.3 Optimization results

We apply the optimization for the cement plant case studies in the UK. We optimize the process and TEA design variables for the top 30 materials in nCAC identified in the screenings for the TSA process and TVSA process at 0.6 bar.

10.3.1 Optimization results for the TSA case

In these case studies, the CO_2 sink is geological storage, which requires a purity above 96 %. In the screening of the TSA process, only four materials out of the top 30 materials in nCAC achieve a purity above 96 % (see Figure S75 and Table S10). After optimization, two additional materials give a purity above 96 %. The optimization increases the purity for the other 25 materials (see Figure S75a), but not above the threshold of 96 %. All degrees of freedom are either at the bounds or limited by a constraint for these materials. For the six structures that meet the purity requirement, trade-offs are observed for the desorption temperature T_{high}, adsorption temperature T_{low}, and the limiting cooling temperature $T_{\text{cool,lim}}$, while the other four degrees of freedom are still at the bounds.

Generally, the optimization decreases the nCAC on average by 9 €$\text{t}_{CO_2}^{-1}$ (see Figure S75b). The Pearson correlation coefficient of 0.76 and Spearman correlation coefficient of 0.64 indicate how well correlated the materials rankings are for non-optimized and optimized results.

This result demonstrates that screening based on fixed parameters accurately captures the trend regarding materials ranking. However, the potential exploit by the optimization depends strongly on the material, leading to changes in the ranks. The subsequent optimization can be used to fine-tune the ranking of the top materials.

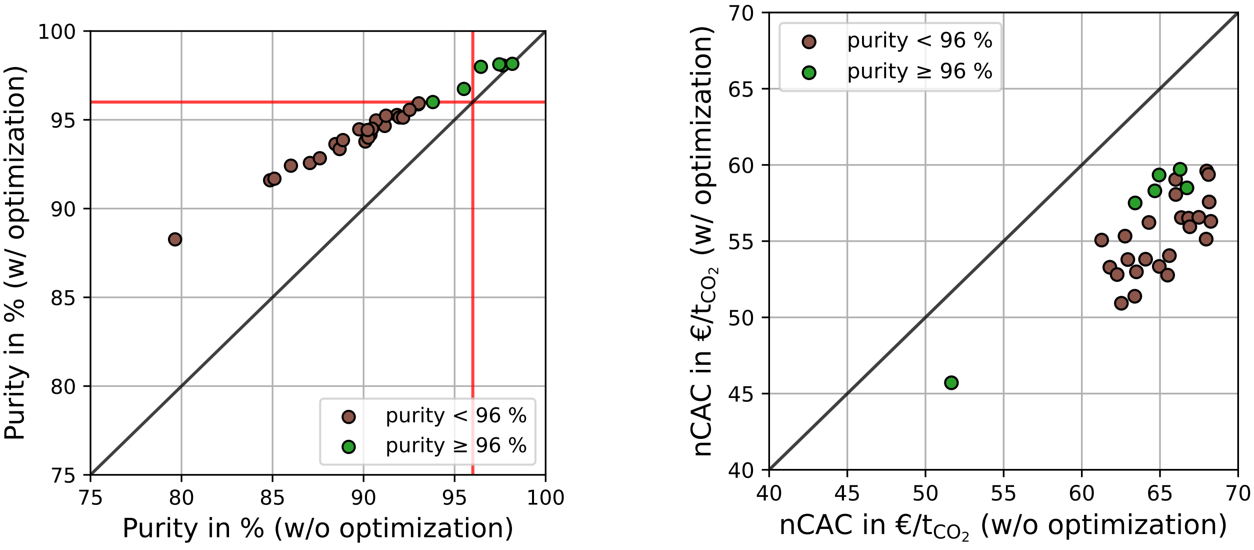

(a) Purity **(b)** Net Carbon Avoidance Cost

Figure S75| Comparison of the results without and with optimization for the TSA process: Purity (left) and nCAC (right). Green dots indicate materials that achieve purity above 96 % after the optimization. The red lines represent the threshold in purity required by the CO_2 sink.

10.3.2 Optimization results for the TVSA case

In contrast to the TSA case, we achieve significantly higher purities with TVSA (see Figure S76 and Table S11). Without any optimization, we have 23 materials out of the top 30 materials in nCAC that achieve a purity above 96 %. For the other seven materials, the optimization increases the purity above 96 % (see Figure S76a). Thus, all materials of the TVSA top 30 meet the purity specification after the optimization. We observe trade-offs for the desorption temperature T_{high}, adsorption temperature T_{low}, the limiting cooling temperature $T_{\text{cool,lim}}$, the vacuum pressure P_{vac}, and the intermediate temperature T_{med}. The other four degrees of freedom are at the bounds for all materials. Generally, the optimization decreases

Table S10: Results obtained from the optimization of the TSA case for the top 30 materials: purity and nCAC with and without optimization as well as the optimized degrees of freedom of the optimization. Acceptable purity is highlighted in bold.

	Without optimization		With optimization								
Rank (Sim)	Purity	nCAC	Purity	nCAC	T_{high}	T_{low}	d_{col}	d_{tube}	l_{col}	ΔT_{pinch}	$T_{cool,lim}$
1	**96.4**	51.7	**98.0**	45.7	145.8	32.1	10	0.025	5	3	29
2	90.1	61.3	93.8	55.1	145.8	27.5	10	0.025	5	3	24.5
3	93.0	61.8	95.9	53.3	145.8	27.5	10	0.025	5	3	24.5
4	91.8	62.3	95.3	52.8	145.8	27.5	10	0.025	5	3	24.5
5	86.0	62.5	92.4	50.9	145.8	27.5	10	0.025	5	3	24.5
6	92.0	62.8	95.1	55.3	145.8	27.5	10	0.025	5	3	24.5
7	91.2	62.9	94.7	53.8	145.8	27.5	10	0.025	5	3	24.5
8	88.5	63.4	93.6	51.4	145.8	27.5	10	0.025	5	3	24.5
9	**97.6**	63.4	**98.3**	57.5	123.1	30.3	10	0.025	5	3	27.3
10	87.1	63.5	92.6	53.0	145.8	27.5	10	0.025	5	3	24.5
11	89.8	64.1	94.5	53.8	145.8	27.5	10	0.025	5	3	24.5
12	88.7	64.3	93.4	56.2	145.8	27.5	10	0.025	5	3	24.5
13	95.5	64.7	**96.7**	58.3	137.5	37	10	0.025	5	3	29
14	**98.2**	64.9	**98.2**	59.3	112.4	32.1	10	0.025	5	3	29
15	90.7	65.0	95.0	53.3	145.8	27.5	10	0.025	5	3	24.5
16	79.6	65.5	88.3	52.8	145.8	27.5	10	0.025	5	3	24.5
17	91.2	65.6	95.2	54.1	145.8	27.5	10	0.025	5	3	24.5
18	90.4	66.0	94.2	59.0	145.8	27.5	10	0.025	5	3	24.5
19	92.2	66.0	95.1	58.1	145.8	27.5	10	0.025	5	3	24.5
20	**97.5**	66.3	**98.1**	59.7	137.4	34.4	10	0.025	5	3	29
21	90.5	66.4	94.5	56.5	145.8	27.5	10	0.025	5	3	24.5
22	93.8	66.7	**96.0**	58.5	131.7	27.8	10	0.025	5	3	24.8
23	90.3	66.8	94.0	56.5	145.8	27.5	10	0.025	5	3	24.5
24	88.9	66.9	93.9	55.9	145.8	27.5	10	0.025	5	3	24.5
25	87.6	67.5	92.8	56.6	145.8	27.5	10	0.025	5	3	24.5
26	84.9	68.0	91.6	55.1	145.8	27.5	10	0.025	5	3	24.5
27	93.0	68.0	95.9	59.6	145.8	27.5	10	0.025	5	3	24.5
28	92.5	68.1	95.6	59.4	145.8	27.5	10	0.025	5	3	24.5
29	90.2	68.1	94.4	57.6	145.8	27.5	10	0.025	5	3	24.5
30	85.1	68.3	91.7	56.3	145.8	27.5	10	0.025	5	3	24.5

the level of vacuum pressure P_{vac} (i.e., less vacuum needs to be applied and the absolute vacuum pressure increases), compared to the 0.6 bar used in the screening, to reduce the nCAC. Moreover, optimization tends to reduce purity for the 23 materials with purity above 96 % after the screening. Interestingly, for six materials, the optimal vacuum pressure is at the adsorption pressure of 1.013 25 bar (upper bound), and thus, no vacuum step is selected by the optimization.

For TVSA, the improvement in nCAC due to the optimization is slightly less than for the TSA case (on average by 7.2 €$t_{CO_2}^{-1}$ —see Figure S76b). With a Pearson correlation coefficient of 0.94 and a Spearman correlation coefficient of 0.90, the correlation between the rankings from non-optimized results and optimized ones is even higher than for the TSA case. Thus, the TVSA screening based on fixed parameters accurately captures the trends between the materials and provides a very accurate material ranking compared to the one obtained after optimization. Small rank changes can be observed among the top 30 materials, which is within the model uncertainty. These results prove that our approach to screening all materials for three process configurations is suitable and efficient for identifying the top candidates for a given case study.

10.3.3 Comparison of the TSA and TVSA cases

For the cement case in the UK, 22 materials are contained in the top 30 rankings of both the TSA and the TVSA processes. To compare both cases, we optimize the eight materials contained in the TSA ranking but not in the TVSA ranking also for the TVSA process and vice versa. Figures S77 and S78 compares the purity, recovery, and nCAC of the two process configurations without and with optimization. The optimization reduces the large differences in purity between TVSA and TSA. However, for the TSA process, the purity cannot be increased above the threshold for most of the materials. For both processes, recovery of the considered materials is above the threshold of 90 % in the simulations and optimizations.

Table S11: Results obtained from the optimization of the TVSA case for the top 30 materials: purity and nCAC with and without optimization as well as the optimized degrees of freedom of the optimization. Acceptable purity is highlighted in bold.

	Without optimization		With optimization										
Rank (Sim)	Purity	nCAC	Purity	nCAC	T_{high}	T_{low}	d_{col}	d_{tube}	l_{col}	ΔT_{pinch}	$T_{cool,lim}$	P_{vac}	T_{med}
1	**99.0**	51.6	**98.7**	45.6	145.8	32.1	10	0.025	5	3	29	1.01325	53.5
2	95.9	58.0	**96**	50.5	131.3	27.8	10	0.025	5	3	24.8	0.93	51.1
3	**96.7**	58.3	**96.5**	50.9	145.8	29.0	10	0.025	5	3	26.0	0.86	45.0
4	**97.4**	59.1	**96.9**	52.3	129.6	28.3	10	0.025	5	3	25.2	0.93	47.4
5	**96.3**	59.1	**96**	52.7	140.8	28.0	10	0.025	5	3	24.9	0.88	43.7
6	**97.3**	59.6	**96.7**	53.6	136.2	27.5	10	0.025	5	3	24.5	0.86	40.8
7	**97.3**	59.7	**96.9**	53.2	145.8	27.5	10	0.025	5	3	24.5	0.96	45.6
8	**97.9**	60.0	**96.9**	52.7	115.9	28.4	10	0.025	5	3	25.4	0.94	45.9
9	**97.1**	60.0	**96.5**	53.5	138.6	27.8	10	0.025	5	3	24.8	1.01325	45.5
10	**97.6**	60.3	**96.9**	53.7	132.8	27.8	10	0.025	5	3	24.7	0.94	44.7
11	93.4	60.5	**96**	52.5	130.1	27.5	10	0.025	5	3	24.5	0.80	53.1
12	**96.9**	61.1	**96**	53.8	124.7	28.4	10	0.025	5	3	25.3	1.01325	48.5
13	**97.4**	61.1	**96.8**	55.0	136.8	28.4	10	0.025	5	3	25.4	0.97	45.6
14	**96.3**	61.4	**96.4**	54.6	132.0	28.4	10	0.025	5	3	25.4	0.69	38.0
15	91.5	61.4	**96**	52.1	126.8	27.5	10	0.025	5	3	24.5	0.69	50.9
16	**97.0**	62.3	**96.5**	54.9	145.8	27.7	10	0.025	5	3	24.6	0.95	44.4
17	**96.1**	62.3	**96**	55.8	145.8	29.1	10	0.025	5	3	26.0	1.01325	44.5
18	**96.9**	62.5	**97.1**	55.2	109.0	27.5	10	0.025	5	3	24.5	0.59	33.2
19	95.1	62.5	**96**	54.6	145.8	29.5	10	0.025	5	3	26.4	0.78	42.9
20	95.9	62.7	**96.3**	53.5	113.4	27.5	10	0.025	5	3	24.5	0.61	33.9
21	93.0	62.8	**96**	55.2	128.3	27.5	10	0.025	5	3	24.5	0.65	48.0
22	**97.0**	62.9	**96.2**	55.5	145.8	30.6	10	0.025	5	3	27.6	0.92	44.9
23	**97.2**	63.3	**96.3**	56.1	135.8	27.5	10	0.025	5	3	24.5	1.01325	44.5
24	95.6	63.4	**96**	56.0	141.6	27.5	10	0.025	5	3	24.5	0.85	43.7
25	**96.1**	63.5	**97.2**	54.8	145.8	27.5	10	0.025	5	3	24.5	0.64	34.6
26	**96.3**	63.6	**96**	56.0	125.0	28.5	10	0.025	5	3	25.4	0.80	43.4
27	**97.8**	63.6	**97.5**	57.4	141.7	30.4	10	0.025	5	3	27.4	0.81	41.2
28	**97.1**	63.6	**96.6**	56.4	141.3	28.3	10	0.025	5	3	25.3	0.84	40.2
29	**97.6**	63.6	**97.1**	57.2	113.0	30.9	10	0.025	5	3	27.8	0.65	38.0
30	**99.3**	63.7	**98.7**	57.5	124.4	30.6	10	0.025	5	3	27.5	1.01325	46.6

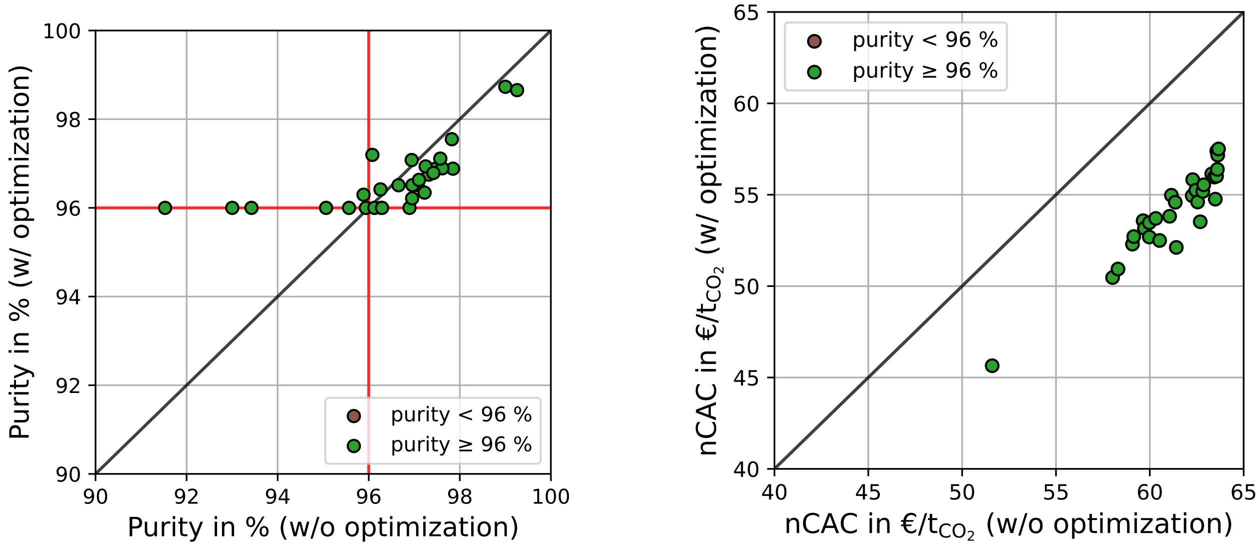

(a) Purity **(b)** Net Carbon Avoidance Cost

Figure S76| Comparison of the results without and with optimization for the TVSA process: Purity (left) and nCAC (right). Green dots indicate materials that achieve purity above 96 % after the optimization. The red lines represent the threshold in purity required by the CO_2 sink.

However, the recovery of the TSA process is much higher than for the TVSA process. The optimization increases recovery for the TVSA process, but TSA is still beneficial.

Interestingly, no strong preference for one process type can be identified based on nCAC. While the nCAC tends to be lower for the TVSA process at 0.6 bar without optimization, the benefit in nCAC is small after the optimization and very similar nCAC is obtained for TVSA and TSA. The optimization increases the Pearson correlation coefficient of nCAC (TSA vs. TVSA) from 0.69 (without optimization) to 0.99 (with optimization) and the Spearman correlation coefficient from 0.51 to 0.99. Thus, for the materials that achieve the required purity for TSA, the TSA process is preferred due to the higher recovery. In contrast, for the materials that cannot achieve the required purity for TSA, the required TVSA process does not lead to higher costs but lower recovery.

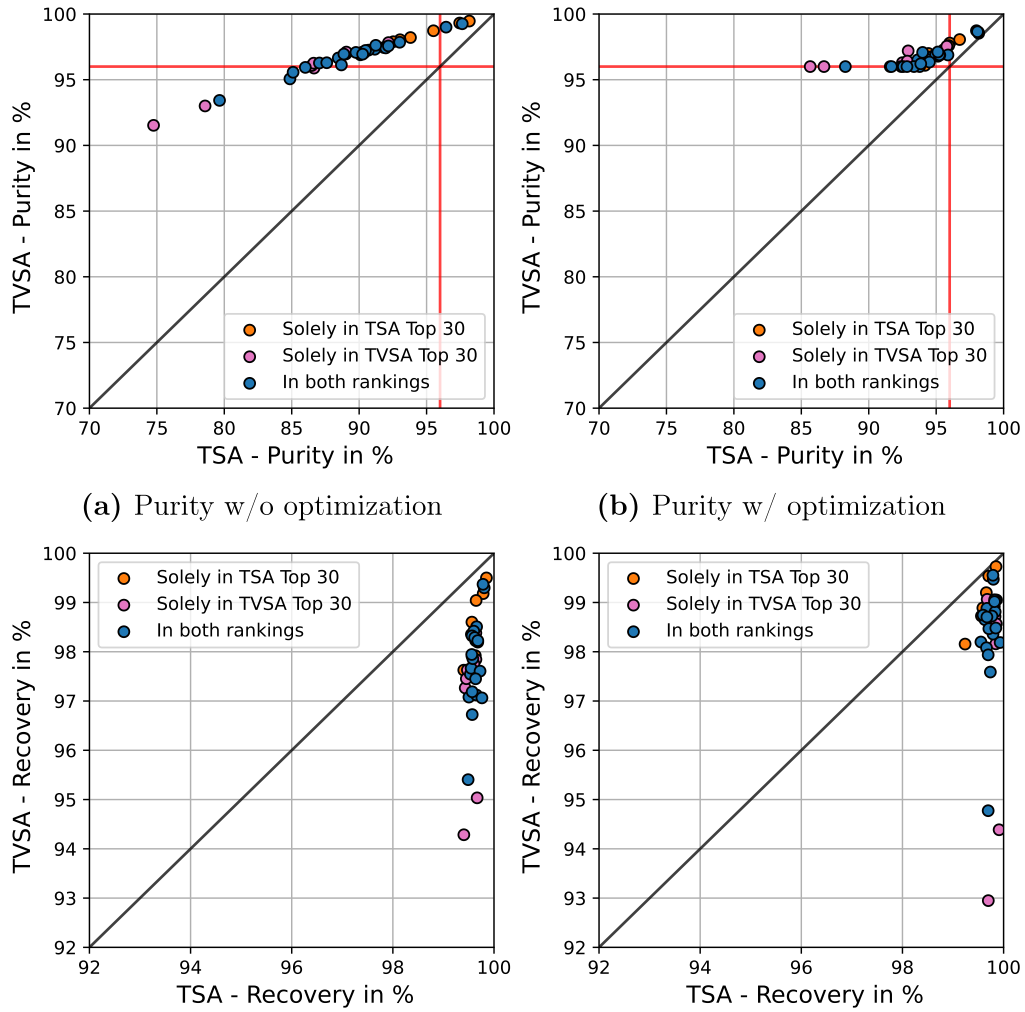

(a) Purity w/o optimization

(b) Purity w/ optimization

(c) Recovery w/o optimization

(d) Recovery w/ optimization

Figure S77| Comparison of the TSA and TVSA process for the cement case in the UK: Without optimization (left) and with optimization (right) for the two selected KPIs: purity (top), recovery (bottom), the third KPI, the nCAC, see Figures S78a and S78b). Blue dots are materials contained in the top 30 of both TSA and TVSA, orange dots are solely contained in the top 30 of TSA, and pink dots are materials solely contained in the top 30 of TVSA.

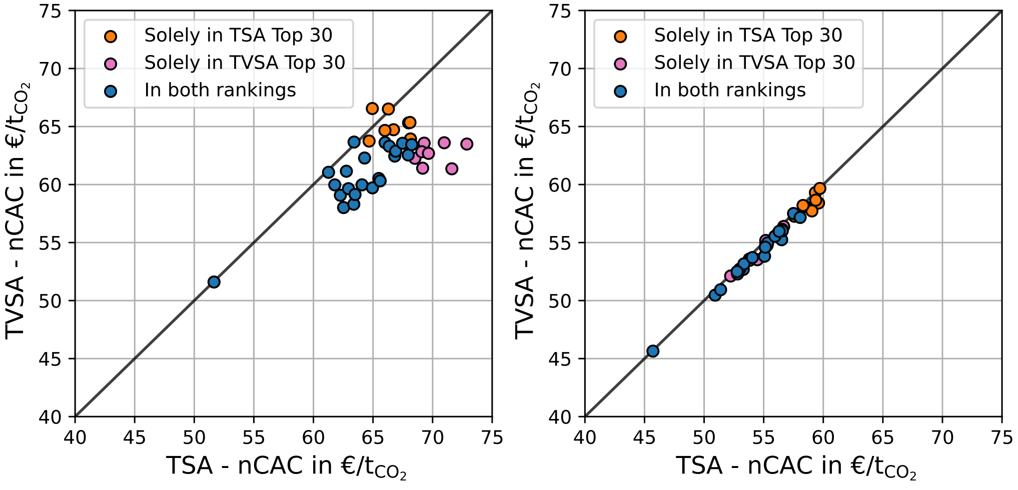

(a) nCAC w/o optimization (b) nCAC w/ optimization

Figure S78| Comparison of the TSA and TVSA process for the cement case in the UK: Without optimization (left) and with optimization (right) for the nCAC for the other two selected KPIs: purity (see Figures S77a and S77b), recovery (see Figures S77c and S77d). Blue dots are materials contained in the top 30 of both TSA and TVSA, orange dots are solely contained in the top 30 of TSA, and pink dots are materials solely contained in the top 30 of TVSA.

11 Feedback loops of the platform

11.1 Feedback to the materials layer

An important application of the PrISMa platform is that we can analyze the impact of uncertainties in calculating material's properties on the different KPIs. This analysis showed the importance of the heat capacity of materials for accurately predicting the heating requirements of the process. It also showed that the ranking of materials depended on how the adsorption isotherms were fitted. This feedback from an earlier version of the platform has resulted in the improvements described in the next subsections.

In addition, we use the platform's output to optimize the different operation and process design parameters. This optimization is described in Section 10.2. Another way to use the platform's feedback is to efficiently screen much larger databases using a machine learning approach (see Section 11.2). In Figure S79, these feedback loops are shown schematically.

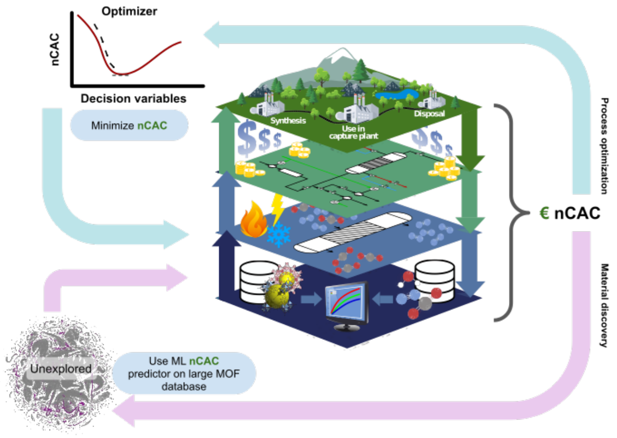

Figure S79| Lowering the cost of carbon capture: Two strategies were developed to lower the nCAC. An iterative feedback strategy using Machine Learning to identify more top-performing materials and an optimization strategy that minimized the nCAC by tweaking process parameters.

11.1.1 Heat capacity

In the platform's first version, we assumed the heat capacity (c_p) to be the same for all MOFs. The reason for this assumption was that the heat capacity was known only for very few materials. We assumed a constant heat capacity equal to the one used in previous work.[S234] The feedback from the platform informed us about how sensitive the heat requirements by the process were to the exact value of the c_p. To address this point, we studied the dependence of the heat capacity on the detailed MOF structure and showed that one can expect a difference in process energy requirements by as much as a factor of two.[S25] Based on this feedback, our current version of the platform uses a machine learning model to predict the heat capacities of all materials (see Section 3.1.1).

11.1.2 Adsorption isotherms

The other important feedback of the platform to the process layer was related to the accuracy at which we needed to compute the mixture adsorption isotherms. In the literature, one can find different models (e.g., Ideal Adsorbed Solution Theory (IAST) or Extended Dual Site Langmuir (EDSL)) that are used to predict mixture isotherms from the pure component isotherms. We showed that the different models to fit the data impacted the ranking of the materials for low CO_2 concentrations in the feed gas stream.[S44] As IAST provided a much more accurate description (75% of the structures were shown to have better accuracy than 7%) of the simulated mixture adsorption data, we used IAST for our mixture data predictions.

11.2 Feedback to extend the materials database

In the PrISMa platform, we have screened about 1,200 curated MOF structures (see Section 3.1.1). The computation at the materials level involves DFT calculations to optimize the structure and get the charges on the atoms needed to carry out the various GCMC calculations to compute the adsorption thermodynamics. We have a dedicated server for these

calculations, which allows us to compute the thermodynamic data for about 50 materials in a few days. Therefore, screening all trillion MOFs proposed by Lee et al.[S37] is practically impossible. Hence, there are a large number of MOF structures that have remained unexplored in the PrISMa platform.

Here, we use the PrISMa platform to leverage knowledge and develop a machine learning model to classify whether a structure has a Net Carbon Avoidance Cost (nCAC) below a given threshold. Such a classification model typically works well if the data is balanced. Ideally, we would like to have an equal number of structures above and below the threshold. However, we typically have a small number of top-performing structures.

To address this issue, we used a feedback loop in which we start with a sufficiently high nCAC to carry out classification with reasonable certainty. From this classification, we select those structures we expect to perform well. For these structures, we compute the complete thermodynamic data. We use these data to retrain our model with a lower threshold. We repeat this procedure for a few iterations until we reach the desired threshold. In this section, we focus on cement in the UK using TVSA (vacuum pressure = 0.6 bar).

11.2.1 Detailed description of the methodology

Machine learning model A gradient boosting classifier model (CatBoost[S235]) was trained using the results from the platform, i.e., for the MOFs in the PrISMa dataset. The available training data was increased with each iteration of the feedback loop, beginning at 1,185 MOFs. Hyperparameter optimization was completed within an inner loop using Parzen estimators via Optuna.[S218] The number of Catboost iterations was selected based on a validation set of 20% of the training data. The feature sets are available in Moosavi et al.[S21]. Features were selected based on their importance via feature importance tests on the validation set. All features above a cut-off were used; the cut-off was chosen by calculating the feature importance of samples from a Gaussian distribution.

The model accuracy was evaluated on 80% of the data and then tested on the 20%. The split was performed based on a stratified random split. Model accuracy varied per round from 80 to 89%. Uncertainty metrics were generated using the virtual ensemble method.[S236]

Strategy for the feedback loop We used the above-mentioned machine-learning model to screen large MOF databases. The BW20K, CoRE2019, and ARABG databases (see Moosavi et al.[S21] for details on databases and organized CIF files) were considered here. The total number of MOFs in these databases is 29,291. The following feedback loop was implemented (see Figure S80):

1. All the materials in the PrISMa database are run through the platform, and KPIs are computed.
2. Our ML model is trained to predict whether the nCAC is higher or lower than a given threshold.
3. As we initially set the threshold for a relatively high value of the nCAC, we use a set of filters to shortlist materials that are expected to be top-performing:
   - Filter 1: The nCAC prediction beats the threshold
   - Filter 2: The material is porous to N_2
   - Filter 3: The material has high pure component CO_2 uptake
   - Filter 4: The material has a high predicted working capacity in the presence of water.
4. The shortlisted MOFs are run through the platform.
5. The process is then repeated from step 2, and the threshold is decreased. The initial threshold was set to 105 €$t_{CO_2}^{-1}$, then reduced to 80 €$t_{CO_2}^{-1}$ in the second round, and then to 70 €$t_{CO_2}^{-1}$ in the third round.

In this way, we can leverage the availability of vast amounts of MOF structures.

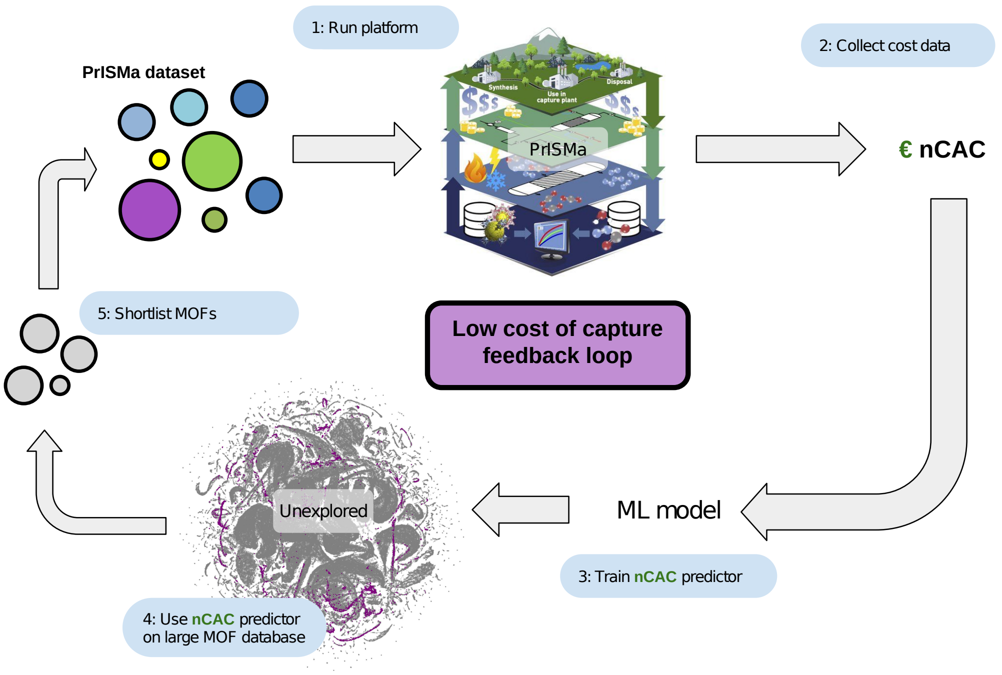

Figure S80| Extending the materials database: An iterative feedback strategy was used to identify more top-performing materials with promising characteristics. The Machine Learning (ML) model is a classifier model that is trained to predict whether a material has a nCAC below a given threshold or not. Out of the materials from the large MOF database with a predicted nCAC below the threshold, we make a short list for running the complete PrISMa platform. We can use a lower threshold value for the nCAC for the next round or iteration loop as we get more top-performing structures.

11.2.2 Performance of the Machine learning model

The results of the first round are shown in Figure S81. The confusion matrix on the test set shows that in 79.5% of the cases, the model correctly predicts whether the nCAC is above or below the 105 €$t^{-1}_{CO_2}$ threshold.

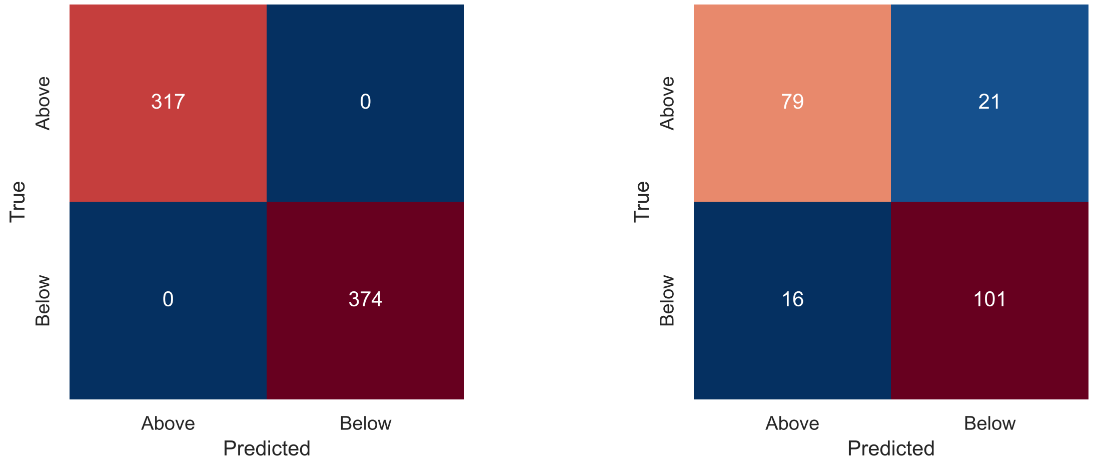

(a) (b)

Figure S81| Model performance: At each round, a model was trained to predict whether the nCAC was above or below a given threshold value. The confusion matrices of the training (a) and test (b) sets show the model performance for a nCAC threshold value of 105 €$t^{-1}_{CO_2}$. The accuracy of this model is 79.5% on the test set.

In Extended Data Figure 6(d), we show for each material of the large database (with approximately 30 000 materials), whether it is above (grey) or below (purple) the threshold. In this representation, two materials plotted close to each other have similar properties.

For each of these materials, we can also estimate the uncertainty of these predictions. These results are shown in Figure S82a for materials below the threshold of 105 €$t^{-1}_{CO_2}$ and in Figure S82b for materials above the threshold. In Figure S82a, the grey dots represent materials that have an nCAC above the threshold, while in Figure S82b the grey dots are the material with an nCAC below the threshold. In these figures, the color coding gives a measure of the accuracy. For example, in Figure S82b, the large pink area is a part of the chemical design space, for which we predict with high certainty that there are no interesting

materials. We also see that the border between the two different areas contains the materials with the highest uncertainty.

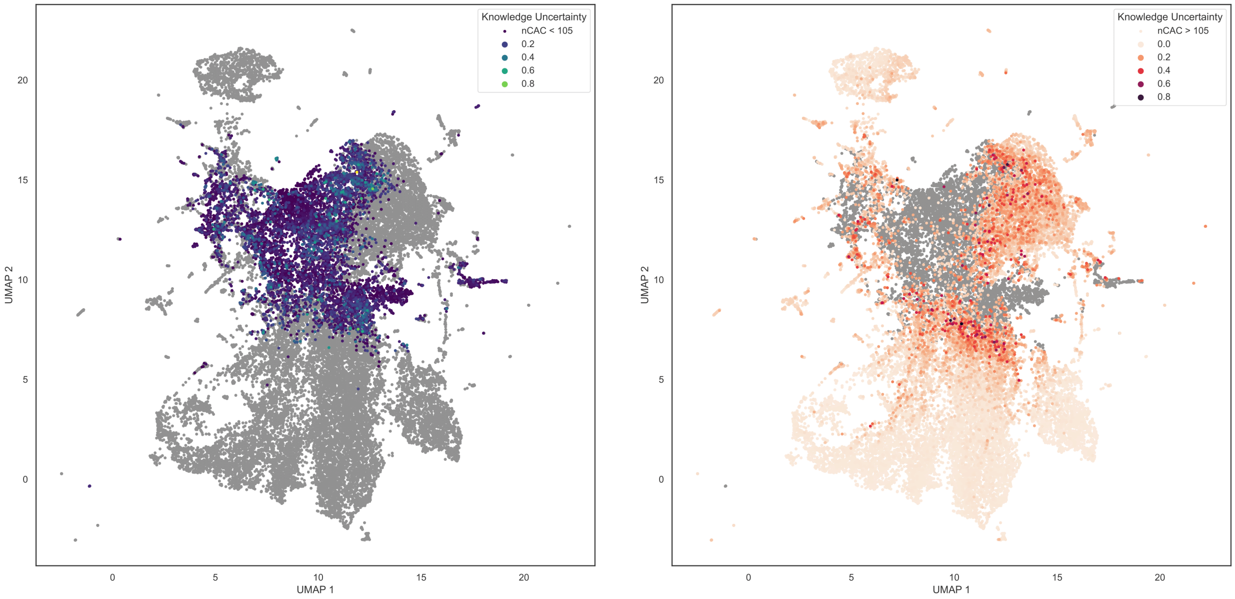

(a) **(b)**

Figure S82| Uncertainty of the Machine Learning model: The color coding gives the uncertainty of the prediction for each material (a) the uncertainty at which we predict that the nCAC is lower than 105 €$t_{CO_2}^{-1}$ and (b) that nCAC is higher than 105 €$t_{CO_2}^{-1}$.

11.2.3 Results of the feedback loop

We started with the model described in the previous section and the feedback strategy was performed three times, each growing the total number of MOFs with a low nCAC and increasing the model's ability to identify low nCAC MOFs.

After each round, the distribution of nCAC values is shown in Figure S84. The PrISMa materials database contains 1,185 materials, 45% of which beat the nCAC benchmark of 105 €$t_{CO_2}^{-1}$. By using the feedback strategy described previously, we obtained 138 shortlisted new materials. When these materials were run through the platform, 137 of them beat the nCAC benchmark of 105 €$t_{CO_2}^{-1}$. These results demonstrate the ability to select top-performing MOFs from large materials databases efficiently.

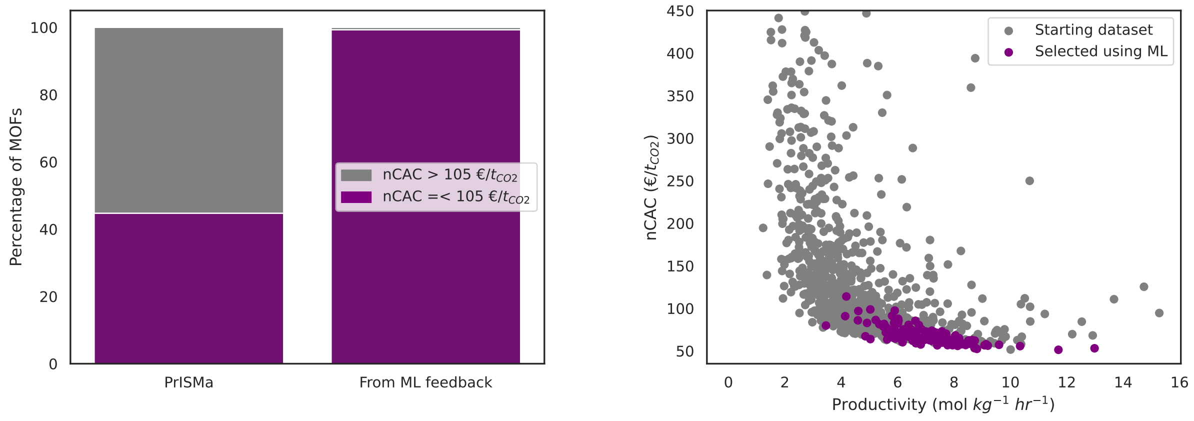

(a) **(b)**

Figure S83| Feedback strategy results after the first round: The starting dataset is the PrISMa materials database, which contains 530 materials (45 %) which beat the nCAC benchmark of 105 € $/t_{CO2}$. A feedback strategy was used to identify more top-performing materials with promising characteristics in large databases. The number of materials that meet the benchmark is shown in a), and their absolute nCAC value is shown in b). Materials selected by the feedback strategy are colored in purple.

In our screening, we cannot include some factors of practical importance (e.g., long-term stability, mechanical properties, resistance against trace components). Hence, we may identify a particular material as top-performing but which fails in practice. For these reasons, we used in our discussion of the case studies the average nCAC of the twenty best-performing materials instead of basing our conclusion on a single material. Hence, our high throughput screening workflow must efficiently generate a large subset of good performers rather than a very small selection of top performers.[S237] The effectiveness of our feedback strategy can be further demonstrated by counting the number of materials with a nCAC lower than 80 €$t_{CO_2}^{-1}$, i.e., resulting from the second feedback Round; only 22% (260 MOFs) of the PrISMa materials database meet this requirement while 86% (119) of the MOFs selected using the feedback strategy do. Hence, we have almost quadrupled our chances of finding a successful material.

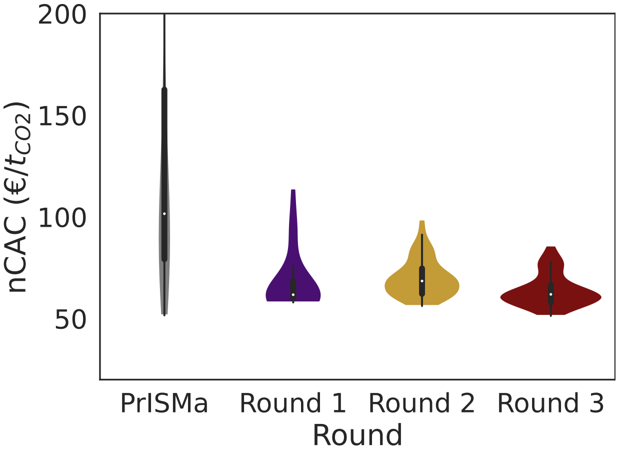

Figure S84| Iterative materials discovery: The iterative feedback strategy generates subsets of MOFs. After each round, the model progressively improves and returns a subset of MOFs with lower nCAC. Violin plots show the distribution of the nCAC for the MOFs identified by the iterative feedback strategy after each round.

It is also interesting to evaluate if our ML model also works for the TSA process. The advantage of a simple TSA process is that it gives a higher recovery, but we do not obtain the required purity for most materials. In our PrISMa database, we only identified four materials within the top 30 that meet the purity requirement, and after optimization, we found two more structures (see Section 10).

Figure S85a shows that for the TVSA process many materials from our ML approach obtain the required purity. Figure S85b shows that our ML approach identified two new materials for the TSA process, without optimization. The top materials that were identified using the iterative feedback strategy were optimized. The goal was to test their performance in a TSA process. We selected all MOFs that achieved a purity over 93 % in the TSA process (41 MOFs) and optimized them using the strategy outlined in Section 10. Figure S86 shows that most MOFs move above the target purity of 96 %. The nCAC is also decreased to a minimum of 46 €$t_{CO_2}^{-1}$. In our initial screening study, we would discard TSA because there are insufficient materials for a reasonable chance of success. These results indicate we may have to revisit this conclusion.

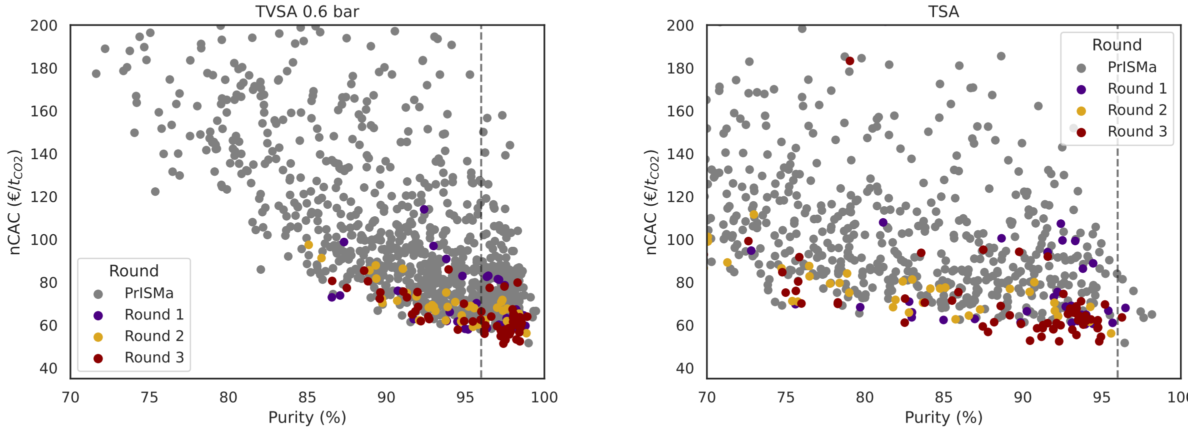

(a) **(b)**

Figure S85| Feedback strategy results: The MOFs identified from the iterative feedback strategy are superimposed against all PrISMa materials for (a) TVSA (vacuum pressure = 0.6 bar) (b) TSA.

Extended Data Figure 6(b) shows the distribution of nCAC for the different process technologies and sources. The top 20 ML materials (in blue) are superimposed onto the top 20 PrISMa materials (in red). A favorable nCAC distribution is observed for coal and cement, whereas NGCC is seen as unfavorable. The iterative feedback strategy was trained using results from a TVSA process (operating with 0.6 bar vacuum) taken from the flue gas of a cement-producing facility. The concentration of CO_2 from a coal flue gas is quite similar to that of cement (14 % vs 18 %), whereas the concentration of CO_2 from an NGCC flue gas is much lower (4 % CO_2). Extended Data Figure 4(a) shows that the ranking of materials for cement and coal is similar but very different from NGCC. This already suggested that we need to retrain our model for our NGCC Case Studies. Our ML model's results are consistent with this observation.

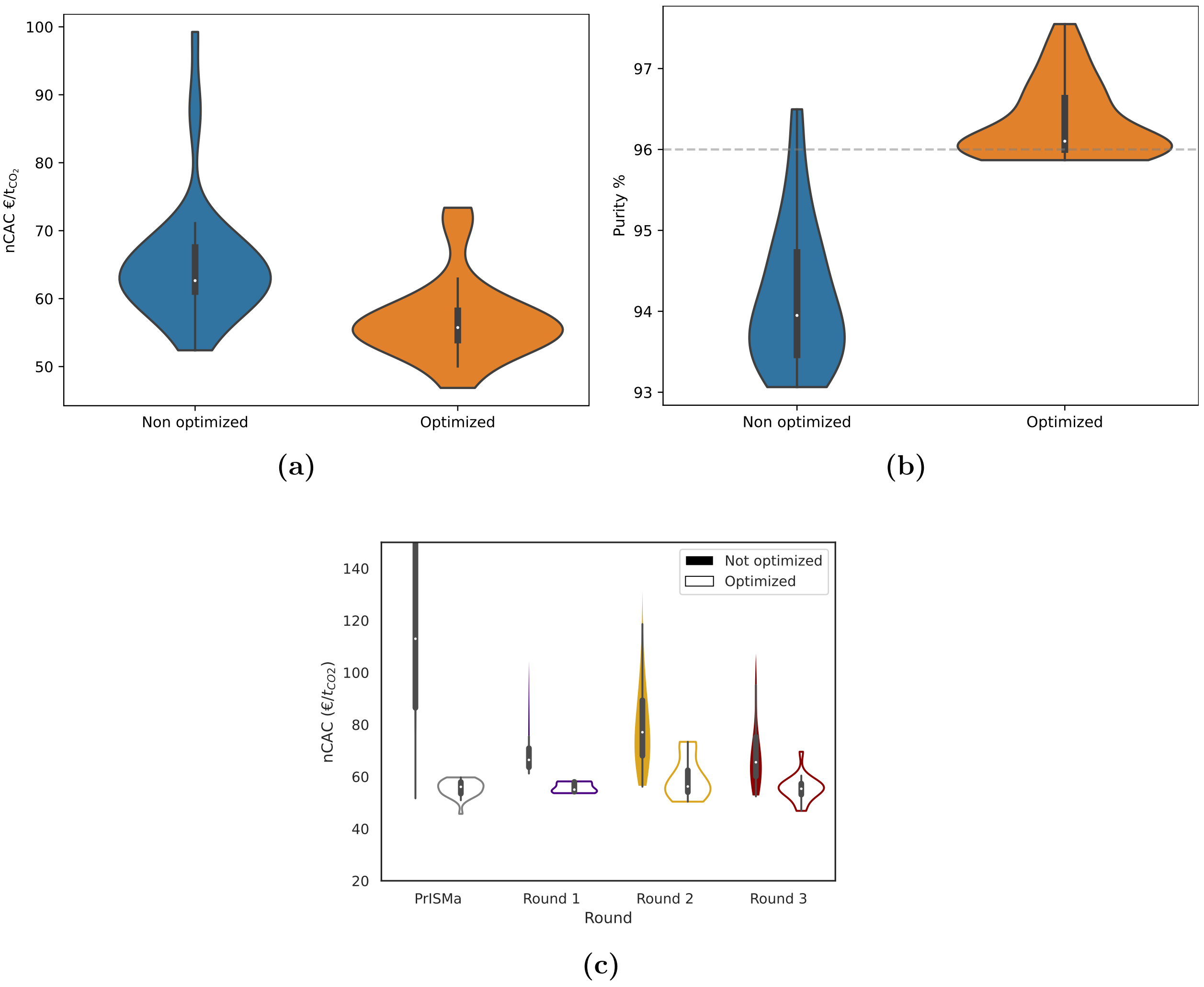

Figure S86| Optimizing the best ML results: Fourty-one MOFs identified from the iterative feedback strategy are optimized (Section 10) for their performance in a TSA process. This figure illustrates that many ML-selected MOFs achieve low nCAC (a) and high purity (b). Figure (c) shows the performance of the ML-selected structures per iterative round for a TSA process in their optimized and non-optimized state. The optimization was set to minimize nCAC with a purity above 96 % as a constraint.

12 Experimental structures

In this section, we describe the experimental data that has been obtained and used to validate the platform's predictions. We focus on two materials: MIP-212 and CALF-20. MIP-212 is a novel MOF, and an initial screening on the platform suggested it would be promising material. CALF-20 is currently used as sorbent in a commercial process to capture CO_2 from a cement source.

12.1 MIP-212

12.1.1 Structure and synthesis

The novel bimetallic copper-aluminum MOF, denoted MIP-212 (MIP stands for Materials from the Institute of Porous Materials of Paris), is formulated as $[Al(\mu_2{-}OH)Cu(\mu_2{-}H_2O)\text{-}(PyC)_2]\cdot$n-solvent, where PyC stands for the pyrazolate 4-carboxylate ligand. It can be obtained as a purple microcrystalline powder from a one-pot synthesis reacting hydrated aluminum nitrate, hydrated copper nitrate, Pyrazole-4-carboxylic acid ($H_2{-}PyC$) and sodium hydroxide in a mixture of deionized water, ethanol, and acetic acid at 100 °C over 48 h. MIP-212 crystalizes in an orthorhombic symmetry with Pmma (№51) space group (a = 6.8114 Å, b = 11.9985 Å, c = 9.6021 Å).

The structure of MIP-212 was solved from synchrotron high-resolution Powder X-ray diffraction (PXRD) data, see Figure S87. It consists of two chains composed of $AlO_4(OH)_2$ and $CuN_4(H_2O)_2$ octahedra along [100] crystallographic orientation. These chains are linked by the pyrazole-4-carboxylate (PyC) hetero-functional ligand where the nitrogen atoms of the pyrazole rings are coordinating the Cu^{2+} ions, and the oxygens of the carboxyl group are coordinated to the Al^{3+} ions. Such crystallographic arrangement forms nanometric lozenge-shaped channels, similar to MIL-53, but exhibiting two types of openings (labeled I and II) with different chemical environments. In pore I, where the Cu-chains are the closest along the c-axis, the Cu ions are coordinated by water molecules pointing to the center of the

channels, and their removal upon sample activation can lead to the formation of OMS. It is worth mentioning that in situ PXRD studies showed that only about half of these water molecules can be removed upon activation at 240 °C under a vacuum. In pore II, where the Al-chains are the closest along the c axis, the Al ions are coordinated by μ_2−hydroxyl groups (μ_2−OH), pointing to the center of the channels. The diameters of both of them vary between ca. 4 Å and ca. 6 Å (including Van der Waals radii) depending on whether the Cu site is in its OMS form or fully coordinated by additional water molecules. More details on this structure can be found in Mansouri et al.[S238].

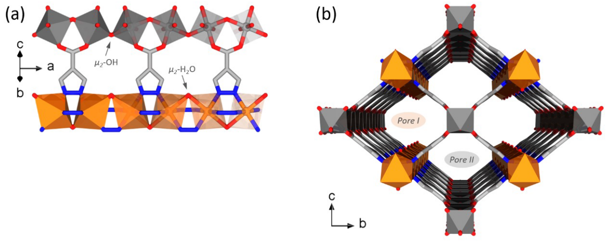

Figure S87| Crystal structure of MIP-212: (a) 1D-chains of $AlO_4(\mu_2-OH)_2$ and $CuN_4(\mu_2-H_2O)_2$ polyhedral linked by the PyC-molecules. (b) View of the 3D structure along the 1D channels. H-atoms are not shown for clarity. Color code: N, blue; O, red; C, grey; Al, grey polyhedron; Cu, orange polyhedron.

MIP-212 can be obtained with very good crystallinity, which remains very well preserved after washing (water or organic solvents) and activation (up to 240 °C under vacuum) steps (Figure S88.a). Interestingly, MIP-212 demonstrated a high CO_2 uptake at ambient temperature and pressure (ca. 2.4 mmol g^{-1} and 3.6 mmol g^{-1} at 0.2 bar and 1 bar of CO_2, respectively) as shown in (Figure S88.b). The obtained experimental pure component isotherms shown in Figure S88b were complemented with the computationally predicted heats of adsorption, density, and heat capacity.

12.1.2 NMR-characterisation of residual water

To make a reliable model for the simulations, we need information about the number of water molecules that are part of the structure. In the simulations, these water molecules

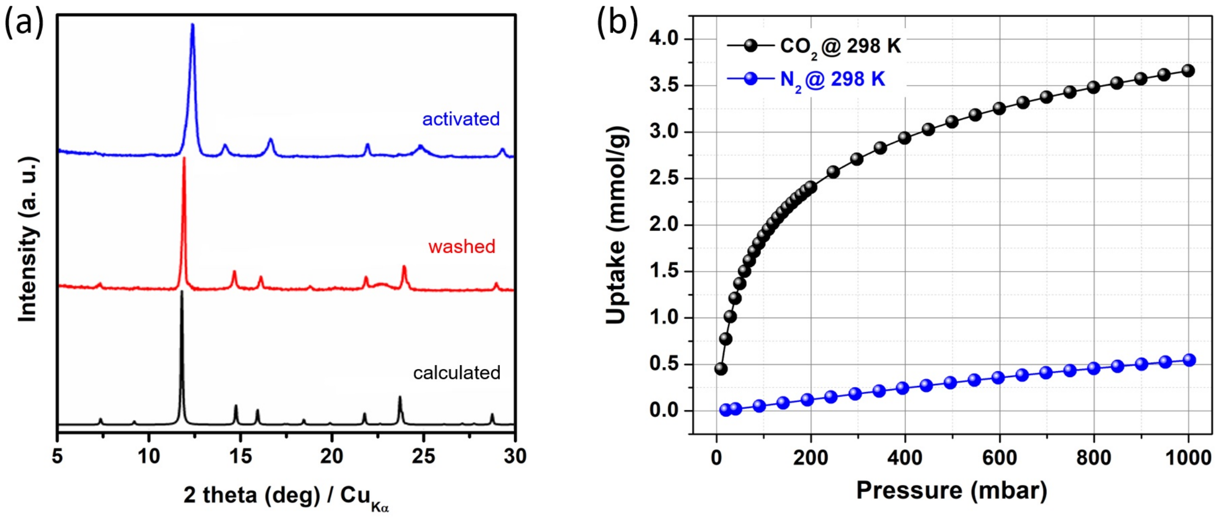

Figure S88| Characterization of MIP-212: (a) Comparison of MIP-212 PXRD patterns; and (b) single component CO_2 and N_2 isotherms measured at 298 K.

are considered "permanent" ligands. The in situ PXRD has shown that (see Section 12.1.1) about 50% of the water molecules stay in the structure. Here, we use solid-state NMR to determine more accurately the number of water molecules that cannot be removed after the activation procedure.

Experimental methods - deuterium exchange: To assign the chemical shift in ^{1}H NMR spectra, the synthesized material was washed with D_2O a couple of times and immersed in the excessive D_2O overnight. Considering H_2O is easily exchanged with deuterium, the disappearance of the signal is associated with the exchange of deuterium in the material.

Experimental methods - solid-state NMR experiments: ^{1}H solid-state nuclear magnetic resonance (^{1}H NMR) spectra were collected with Bruker Avance NEO (^{1}H Larmor frequency 400 MHz) using a 1.3 mm probe. The activated materials are packed in a rotor under an Ar glovebox and inserted into the magnet. The spectra were acquired with echo pulse at an ultra-fast spinning rate between 60 kHz to 65 kHz. The quantitative proton measurements were performed with a recycle delay of 5 s and deconvoluted by the software Dmfit.[S239]

Results The activation of MIP-212 achieves roughly 50-70% removal of water, yielding Open metal site (OMS) for the adsorption. The remaining water in the activated sample is determined by the ultra-high speed magic angle spinning 60 kHz to 65 kHz ^1H solid-state Nuclear Magnetic Resonance in Figure S89. The presence of metal ion Cu^{2+} contributes to a paramagnetic chemical shift to higher and lower resonances. Notably, the comparison with the deuterium exchanged sample (black, bottom) identifies H_2O coordinating with Cu at 12.1 ppm (D), -55 ppm (G), -90 ppm (H), OH coordinating with Al at 1 ppm (E), and PyC linker at 50 ppm (A), 30 ppm (B), 20 ppm (C), and 7 ppm (D). The spectral deconvolution enables the integration of the protons associated with each and results in x = 30–50% of water amount from the expected chemical formula $AlCu(\mu_2{-}OH)(C_8N_4O_4H_4)(\mu_2{-}H_2O)x$.

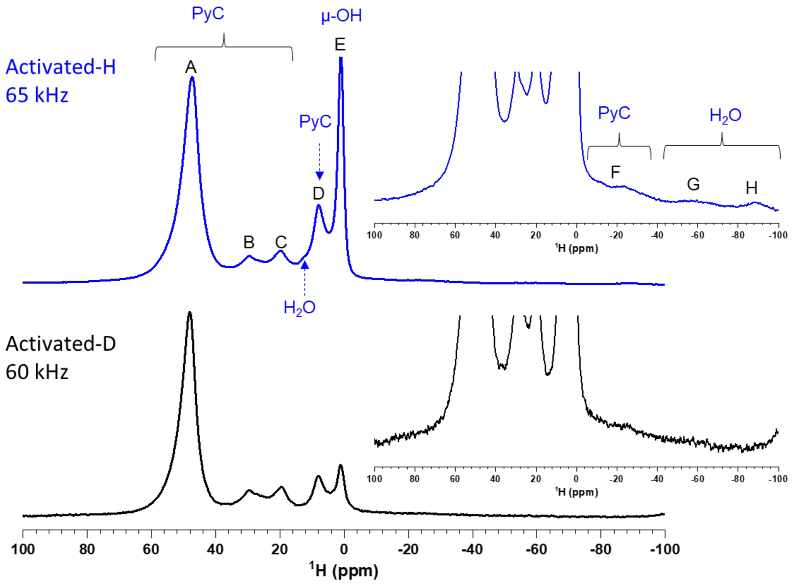

Figure S89| NMR spectra of MIP-212: the ^1H solid-state NMR spectra of the activated MIP-212, with (black signal) and without (blue signal) deuterium exchange.

12.1.3 Performance testing: Breakthrough curves

Experimental method The breakthrough experiments for MIP-212 were carried out in a bespoke system shown in Figure S90. The piping system was built with 6.4 mm stainless-steel tubing, Swagelok fittings, and a stainless-steel adsorption column (length 220 mm,

external diameter 9.5 mm, internal diameter 7.1 mm) equipped with a heating unit. The inlet gas composition was controlled with three mass flow controllers (Bronkhorst EL-Flow) connected to carbon dioxide (99.8 %), helium (99.996 %), and nitrogen (99.998 %) with a flow range of 0.001 mL_n/min to 2.000 mL_n/min, 0.1 mL_n/min to 100 mL_n/min, and 0.1 mL_n/min to 100 mL_n/min, respectively.

Before beginning the breakthrough tests, the MIP-212 sample was activated at 240 °C for 7 h under a secondary vacuum. For dry breakthrough tests, 300 mg of MIP-212 were loaded into the column and held in place with glass wool (Merck). The system was then purged with a Helium (He) flow (30 mL_n/min) to eliminate residual gases from exposure to the atmosphere during the loading of the column. Helium (30 mL_n/min) is then passed through the column whilst the column is heated at 240 °C, and this step was maintained for a minimum of 1 hour to activate the MOF sample. Next, the system was cooled to 37 °C, the adsorption temperature for the cement case study. The feed gas mixture (20 % CO_2 and 80 % N_2) was then passed through the column (10 mL min^{-1}) during the adsorption step, with a constant flow of He (20 mL min^{-1}) going via the reactor bypass. The outlet mass flow rate was measured by a Coriolis flow meter (Bronkhorst mini Cori-flow). The outlet gas composition was monitored using a Hiden HPR-20 mass spectrometer, calibrated using gas mixtures of known compositions.

For wet breakthrough experiments, N_2 is passed through a temperature-controlled water-filled bubbler to wet the gas stream (85 percent relative humidity). The system was properly insulated and heated such that condensation of water did not occur. The temperature of the lines, bubbler, and adsorption column was monitored through a series of K-type thermocouples. The outlet mass flow rate was determined using the He reference flow method for the wet breakthrough tests.[S240] This was required to avoid condensation in the Coriolis meter. It has already been shown that the two methods achieve similar results; hence, a comparison between breakthrough profiles can be made.[S241] Blank experiments were performed using the

same conditions (dry & wet) and inlet flow rates using glass beads instead of the absorbate, to account for the intraparticular and bed void space.

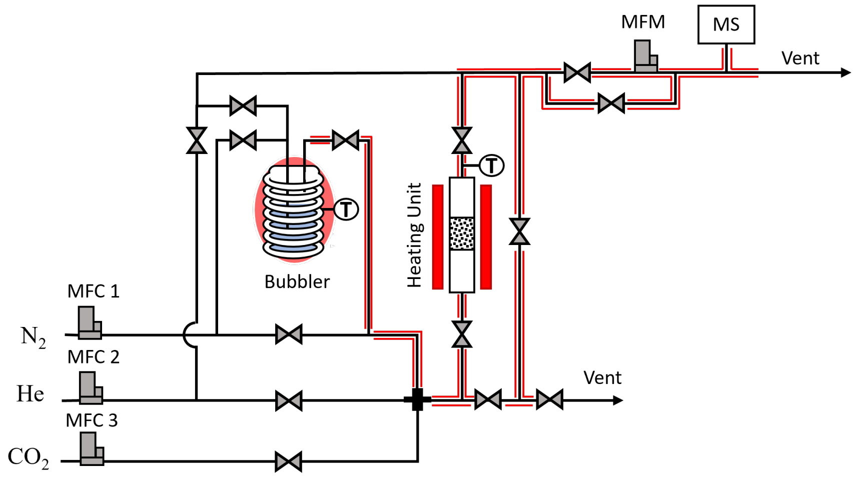

Figure S90| System used for dynamic breakthrough testing of MIP-212. MFC = Mass Flow Controller; MFM = Mass Flow Meter; MS = Mass Spectrometer.

Results The results of the binary and ternary breakthrough experiments for MIP-212 are presented in Figure S91.

For the wet experiment, the experimental breakthrough transient for CO_2 starts at ≈2 min. The outlet molar flow $y(t)Q(t)$ reaches the feed rate $y_{in}Q_{in}$ at ≈5 min, during which ≈1.1 mol kg^{-1} CO_2 was adsorbed in the column. The concentration transient matches substantially that of the dry experiment. The dynamic CO_2 uptakes in both runs are ≈50% lower than the corresponding single-component CO_2 uptake (≈2.2 mol kg^{-1}) measured by volumetry at 20 kPa and 37 °C. This is likely due to a combination of heat release upon CO_2 and H_2O adsorption and limited cooling available to the column exterior under the experimental conditions. Besides, it is important to note that the volumetric CO_2 isotherm was measured on a sample pre-activated under dynamic vacuum at 240 °C, while for breakthrough measurements, the activation was performed under hot gas flow, which may not be optimal to remove all Cu-coordinated water molecules. Some material's capacity may be recovered by applying active cooling to the adsorber/heat-exchangers during cyclic operation.

The experimental breakthrough transient for H_2O starts at ≈100 minute and completes at ≈350 minutes, corresponding to an integral dynamic H_2Ouptake of 13.5 mol kg^{-1} at 37 °C. The dynamic H_2O uptake is ≈30% lower than the reference H_2O uptake (≈19.0 mol kg^{-1}) for the material at 85% RH and 37 °C based on gravimetric isotherm measurements. Between 6 min<t<310 min, a pronounced roll-up phenomena wherein $y(t)Q(t) > y_{\text{in}}Q_{\text{in}}$ is observed for CO_2, indicating substantial displacement by H_2O due to competitive co-adsorption. The same is observed for N_2 in both the dry and wet runs as the most weakly adsorbed component in the system.

The significant disparity between the breakthrough times of CO_2 and H_2O indicates moisture penetration of $< 5\%$ of the bed length when the feed adsorption step is operated to incipient CO_2 breakthrough. This corroborates reasonably well with the maximum water penetration length (approx. 2%) derived for MIP-212 for the TSA cycle modeled on the PrISMa platform. The CO_2/N_2 selectivity on MIP-212 is evident from the rapid (<2 min) elution of N_2 from the outlet of the column. This suggests a high likelihood of operating a stable wet CO_2 capture process under typical TSA regeneration conditions.

12.2 CALF-20

For CALF-20, we use the isotherm and heat of adsorption data reported in Nguyen *et al.*[S132] for structured CALF-20 particles (with 20 wt% polysulfone binder). To have a fair comparison with other simulated materials, we apply the same particle structural properties (i.e., 0% binder content and 0.35 macroporosity). For this, the CALF-20 isotherms[S132] are scaled by discounting the 20% binder.[S59] The crystal density is computed from the experimental crystal structure.[S59] The heat capacity value is estimated using our ML model (see Section 3.1.1).

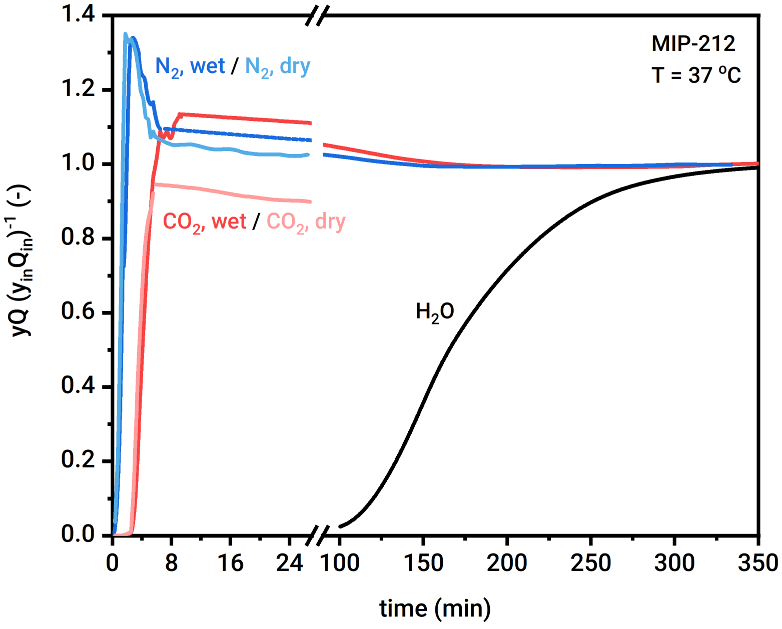

Figure S91| Breakthrough curves of MIP-212 under wet (18 % CO2, 85 % RH, balance % N2) and dry conditions (20 % CO2, balance % N2) .

Acronyms

**AiiDA**	Automated Interactive Infrastructure and Database for Computational Science.
**APOS**	at the Point of Substitution.
**AS**	Adsorption Step.
**BAT**	Best Available Technology.
**BEIS**	Department for Business, Energy and Industrial Strategy of the UK government.
**CAC**	Carbon Avoidance Cost.
**CAPEX**	Capital Expenditure.
**CC**	Climate Change.
**CCC**	Carbon Capture Cost.
**COF**	Covalent Organic Framework.
**COP**	Coefficient of Performance.
**CoRE-MOF**	Computation-Ready Experimental MOF.
**CS**	Cooling Step.
**CSD**	Cambridge Structural Database.
**CSS**	Cyclic Steady state.
**DCF**	Discount cash flow rate.
**DDEC**	Density Derived Electrostatic and Chemical net atomic charges.
**DFT**	Density Functional Theory.
**DMA**	Dimethylacetamide.
**DMF**	Dimethylformamide.
**EA**	Euro Area.

EBTF European Benchmarking Task Force.

EDSL Extended Dual Site Langmuir.

EPC Engineering, Procurement and Construction.

FeOs Framework for Equations of State and Classical Density Functional Theory.

FG Foreground data.

FOB Free-On-Board.

GCMC Grand-Canonical Monte Carlo.

GWP Global Warming Potential.

HICP Harmonised Index of Consumer Prices.

HS Heating Step.

IAST Ideal Adsorbed Solution Theory.

IHS Intermediate Heating Step.

KPI Key Performance Indicator.

KPIs Key Performance Indicators.

LCA Life Cycle Assessment.

LCI Life Cycle Inventory.

LCIA Life Cycle Impact Assessment.

LCOC Levelised Cost of Capture.

LCOE Levelised Cost of Electricity.

LLE liquid-liquid-equilibrium.

LM Labour and Materials.

MEA Mono-Ethanol-Amine.

MINLP Mixed-Integer Nonlinear Program.

MIP Materials from the Institute of Porous Materials of Paris.

ML Machine Learning.

MOF Metal Organic Framework.

MR:MM Material Resources: Metals/Minerals.

nCAC Net Carbon Avoidance Cost.

NGCC Natural Gas Combined Cycle.

NLP Nonlinear Program.

NMR Nuclear Magnetic Resonance.

NRTL nonrandom two-liquid model.

NVT Constant Number of Particles, Volume, and Temperature.

OMS Open metal site.

OPEX Operational Expenditure.

PrISMa **Pr**ocess-**I**nformed design of tailor-made **S**orbent **Ma**terials.

PXRD Powder X-ray diffraction.

SCR Selective Catalytic Reduction.

SMILES Simplified molecular-input line-entry system.

SPECCA Specific Primary Energy Consumption per CO_2 Avoided.

SPT SMILES-to-Properties-Transformer.

TCR Total Capital Requirement.

TDC Total Direct Cost.

TEA Techno-Economic Analysis.

TraPPE Transferable Potentials for Phase Equilibria Force Field.

TSA Temperature Swing Adsorption.

TVSA Temperature Vacuum Swing Adsorption.

UFF Universal Force Field.

VS Vacuum Step.

WRC Water resistance coefficient.

WRC* Water resistance coefficient in a water-contaminated material.

Symbols

A	heat transfer area ($m^2\,m^{-3}$).
A_{tubes}	total cross-sectional area of the tubes per column (m^2).
$C_{material,\ repl}$	cost of material replacement that is a function of years y (M€).
DCF	discount cash flow rate.
H_{ads}	multi-component enthalpy of adsorption (kJ).
$N_{columns}$	number of columns in the capture plant per train.
N_{comp}	number of components in the adsorption process that depends on the case study.
N_{trains}	number of trains in the capture plant.
$N_{utility}$	number of equipment needed in the entire capture plant per utility (i.e., power, heating, cooling).
Q_{sens}	sensible heat (kJ).
$Q_{th\ input,\ ref}$	thermal energy input of the reference power plant (MW).
T_{HEX}	temperature of the heat exchanger media (K).
U	heat transfer resistance ($kJ\,m^{-2}\,s^{-1}\,K^{-1}$).
V_{bed}	volume of the bed that is given by the total volume of the tubes in the packed multi-tubular heat exchanger column (m^3).
$\dot{W}_{cc}$	power output of the power plant with carbon capture (MW).
$\dot{W}_{utility}$	power requirement of a utility unit (MW).
$\dot{W}_{ref}$	power output of the reference power plant (MW).
$\dot{m}_{CO_2\ avoided}$	total mass flow rate of CO_2 avoided from the capture plant considering the energy-related life cycle CO_2-eq. emissions of the capture plant ($kgCO_2/h$).
$\dot{m}_{CO_2\ emissions,\ cc}$	total mass flow rate of CO_2 emissions of the energy supply by utilities to the capture plant ($kgCO_2/h$).

$\dot{m}_{CO_2 \text{ avoided NET}}$ total mass flow rate of CO_2 avoided from the capture plant considering the total life cycle CO_2-eq. emissions of the capture plant ($kgCO_2/h$).

$\dot{m}_{CO_2 \text{ captured}}$ total mass flow rate of CO_2 that is captured in the process ($kgCO_2/h$).

$\dot{n}_{\text{feed, tot}}$ molar flow rate of the feed stream to the capture plant ($mol\,s^{-1}$).

$\dot{n}_{\text{feed}}$ molar flow rate of the feed stream to the column ($mol\,s^{-1}$).

ϵ_{bed} void fraction of the bed, in this case the tubes in the packed multi-tubular heat exchanger column.

ϵ_{pellet} void fraction of the pellet.

γ_{mix} specific heat ratio of the mixture.

$LCOE_{cc}$ levelized cost of electricity for the reference power plant with carbon capture (€/MWh).

$LCOE_{ref}$ levelized cost of electricity for the reference power plant without CCS (€/MWh).

MW_{CO_2} molecular weight of CO_2 ($kg\,mol^{-1}$).

$PE_{ind,ref}$ indirect primary energy consumption of the reference power plant per unit electricity [MJ/MWh].

$PE_{utility}$ total primary energy consumption of a utility per unit energy [MJ/MWh].

TCR_{cc} total capital required including interest during construction for the capture plant (€).

TCR_{ref} total capital required including interest during construction for the reference plant (€).

ρ_{bulk} bulk bed density ($kg\,m^{-3}$).

ρ_{crystal} density of the crystal ($kg\,m^{-3}$).

ρ_{pellet} density of the pellet ($kg\,m^{-3}$).

υ_{out} velocity of the gas leaving the column under vacuum ($m\,s^{-1}$).

$c_{\text{p, bed}}$ heat capacity of the packed bed ($kJ\,m^{-3}\,K^{-1}$).

$c_{\text{p, solid}}$ heat capacity of the solid ($kJ\,kg^{-1}\,K^{-1}$).

c_p heat capacity ($kJ\,kg^{-1}\,K^{-1}$).

$e_{CO_2\ intensity,\ ref}$ total CO_2 intensity per unit of electricity or product produced for the reference power plant without CCS (tCO_2/MWh).

$e_{CO_2\ intensity,\ utility}$ CO_2 intensity per unit of utility (i.e., power, heating, cooling) used (tCO_2/MWh).

$e_{CO_2\ intensity,\ cc}$ total CO_2 intensity per unit of electricity produced for the reference power plant with carbon capture including the energy-related life cycle CO_2-eq. emissions of the capture plant (tCO_2/MWh).

$hr(yr)$ capture plant operational hours per year (h).

m_{solid} mass of the solid in the bed, in this case the tubes in the packed multi-tubular heat exchanger column (kg).

$q_{th,\ cooling}$ specific thermal energy for cooling ($kJ\,mol^{-1}$).

q_{th} specific thermal energy for heating ($kJ\,mol^{-1}$).

t_{cycle} cycle time, the total time that is required to operate a complete cycle of the process (h).

w_{el} specific electrical energy required for the vacuum pump and the compressor ($kJ\,mol^{-1}$).

effective recovery percentage of the amount of key component (in this case CO_2) that is avoided during the entire life cycle of the capture plant over to the one that enters the column at AS and CS (%).

product quantity of key component (in this case CO_2) that is recovered/produced at HS per column ($molCO_2$).

recovery percentage of the amount of key component (in this case CO_2) that is recovered at HS over to the one that enters the column at AS and CS (%).

References

(S1) Sanchez Fernandez, E.; Goetheer, E.; Manzolini, G.; Macchi, E.; Rezvani, S.; Vlugt, T. Thermodynamic assessment of amine based CO_2 capture technologies in power plants based on European Benchmarking Task Force methodology. *Fuel* **2014**, *129*, 318–329, DOI: https://doi.org/10.1016/j.fuel.2014.03.042.

(S2) Manzolini, G.; Sanchez Fernandez, E.; Rezvani, S.; Macchi, E.; Goetheer, E.; Vlugt, T. Economic assessment of novel amine based CO_2 capture technologies integrated in power plants based on European Benchmarking Task Force methodology. *Applied Energy* **2015**, *138*, 546–558, DOI: https://doi.org/10.1016/j.apenergy.2014.04.066.

(S3) Anantharaman, R.; Fu, C.; Roussanaly, S.; Voldsund, M. D3.2 CEMCAP framework for comparative techno-economic analysis of CO_2 capture from cement plants. 2017; https://tinyurl.com/ycyk7t6y.

(S4) Anantharaman, R.; Fu, C.; Roussanaly, S.; Voldsund, M. D4.2 Design and performance of CEMCAP cement plant with MEA post combustion capture. 2016; https://tinyurl.com/3hae3cp5.

(S5) IEAGHG Effects of Plant Location on the Costs of CO_2 Capture. IEAGHG Technical Report 2018-04. 2018; http://documents.ieaghg.org/index.php/s/nsoj0ODazltwJNb.

(S6) CEPCI THE CHEMICAL ENGINEERING PLANT COST INDEX 2022. https://www.chemengonline.com/pci-home, 2022.

(S7) Department for Business, Energy & Industrial Strategy Statistical data set International industrial energy prices: Industrial electricity prices in the IEA (Table 531) & Prices of fuels purchased by the manufacturing industry in GB (Ta-

ble 311 - 414). 2022; https://www.gov.uk/government/statistical-data-sets/prices-of-fuels-purchased-by-manufacturing-industry.

(S8) Global Economic Data, Indicators, Charts and Forecasts, CEIC China Usage Price: Electricity price for industry 35 kW & Above: 36 City Avg. price, Guangdong and Shandong regions. 2022; https://tinyurl.com/5cenrzdk.

(S9) Department for Business, Energy & Industrial Strategy Statistical data set International industrial energy prices: Average prices of fuels purchased by the major UK power producers (Table 321). 2022; https://www.gov.uk/government/statistical-data-sets/prices-of-fuels-purchased-by-major-power-producers.

(S10) Global Economic Data, Indicators, Charts and Forecasts, CEIC China Usage Price: 36 City Avg: Natural Gas: Natural Gas for Industry. 2022; https://tinyurl.com/ywt5jubt.

(S11) EIA, US Energy Information Administration Coal prices and outlook. 2021; https://www.eia.gov/energyexplained/coal/prices-and-outlook.php.

(S12) Global Economic Data, Indicators, Charts and Forecasts, CEIC China Usage Price: Power coal prices prices above 5800 cal (high quality). 2022; https://tinyurl.com/3k825n48.

(S13) Chung, Y. G.; Haldoupis, E.; Bucior, B. J.; Haranczyk, M.; Lee, S.; Zhang, H.; Vogiatzis, K. D.; Milisavljevic, M.; Ling, S.; Camp, J. S.; others Advances, Updates, and Analytics for the Computation-Ready, Experimental Metal–Organic Framework Database: CoRE MOF 2019. *Journal of Chemical & Engineering Data* **2019**, *64*, 5985–5998, DOI: 10.1021/acs.jced.9b00835.

(S14) Computation-Ready, Experimental Metal-Organic Framework (CoRE MOF) 2019 Dataset. doi: 10.5281/zenodo.3370144, Accessed: 2019-06.

(S15) Groom, C. R.; Bruno, I. J.; Lightfoot, M. P.; Ward, S. C. The Cambridge structural database. *Acta Crystallographica Section B: Structural Science, Crystal Engineering and Materials* **2016**, *72*, 171–179, DOI: 10.1107/S2052520616003954.

(S16) Willems, T. F.; Rycroft, C. H.; Kazi, M.; Meza, J. C.; Haranczyk, M. Algorithms and tools for high-throughput geometry-based analysis of crystalline porous materials. *Microporous Mesoporous Mater.* **2012**, *149*, 134–141, DOI: 10.1016/j.micromeso.2011.08.020.

(S17) Ongari, D.; Boyd, P. G.; Kadioglu, O.; Mace, A. K.; Keskin, S.; Smit, B. Evaluating Charge Equilibration Methods To Generate Electrostatic Fields in Nanoporous Materials. *J. Chem. Theory Comput.* **2019**, *15*, 382–401, DOI: 10.1021/acs.jctc.8b00669.

(S18) Wilmer, C. E.; Kim, K. C.; Snurr, R. Q. An extended charge equilibration method. *J. Phys. Chem. Lett.* **2012**, *3*, 2506–2511, DOI: 10.1021/jz301439a.

(S19) Barthel, S.; Alexandrov, E. V.; Proserpio, D. M.; Smit, B. Distinguishing Metal–Organic Frameworks. *Cryst. Growth Des.* **2018**, *18*, 1738–1747, DOI: 10.1021/acs.cgd.7b01663.

(S20) Hagberg, A.; Swart, P.; S Chult, D. Exploring network structure, dynamics, and function using NetworkX. 2008; https://www.osti.gov/servlets/purl/960616.

(S21) Moosavi, S. M.; Nandy, A.; Jablonka, K. M.; Ongari, D.; Janet, J. P.; Boyd, P. G.; Lee, Y.; Smit, B.; Kulik, H. J. Understanding the diversity of the metal-organic framework ecosystem. *Nat. Commun.* **2020**, *11*, DOI: 10.1038/s41467-020-17755-8.

(S22) Momma, K.; Izumi, F. VESTA 3 for three-dimensional visualization of crystal, volumetric and morphology data. *Journal of applied crystallography* **2011**, *44*, 1272–1276, DOI: 10.1107/S0021889811038970.

(S23) Jablonka, K. M.; Zasso, M.; Patiny, L.; Marzari, N.; Pizzi, G.; Smit, B.; Yakutovich, A. V. Connecting lab experiments with computer experiments: Making" routine" simulations routine. *ChemRxiv* **2021**, DOI: 10.33774/chemrxiv-2021-h3381-v2.

(S24) Ongari, D.; Yakutovich, A. V.; Talirz, L.; Smit, B. Building a consistent and reproducible database for adsorption evaluation in covalent–organic frameworks. *ACS central science* **2019**, *5*, 1663–1675, DOI: 10.1021/acscentsci.9b00619.

(S25) Moosavi, S. M.; Novotny, B. Á.; Ongari, D.; Moubarak, E.; Asgari, M.; Özge Kadioglu; Charalambous, C.; Ortega-Guerrero, A.; Farmahini, A. H.; Sarkisov, L.; Garcia, S.; Noé, F.; Smit, B. A data-science approach to predict the heat capacity of nanoporous materials. *Nat. Mater.* **2022**, *21*, 1419–1425, DOI: 10.1038/s41563-022-01374-3.

(S26) Pizzi, G.; Cepellotti, A.; Sabatini, R.; Marzari, N.; Kozinsky, B. AiiDA: automated interactive infrastructure and database for computational science. *Computational Materials Science* **2016**, *111*, 218–230, DOI: 10.1016/j.commatsci.2015.09.013.

(S27) Hutter, J.; Iannuzzi, M.; Schiffmann, F.; VandeVondele, J. cp2k: atomistic simulations of condensed matter systems. *Wiley Interdisciplinary Reviews: Computational Molecular Science* **2014**, *4*, 15–25, DOI: 10.1002/wcms.1159.

(S28) Jablonka, K. M.; Ongari, D.; Moosavi, S. M.; Smit, B. Using collective knowledge to assign oxidation states of metal cations in metal–organic frameworks. *Nature Chemistry* **2021**, *13*, 771–777, DOI: 10.1038/s41557-021-00717-y.

(S29) Perdew, J. P.; Burke, K.; Ernzerhof, M. Generalized gradient approximation made simple. *Physical review letters* **1996**, *77*, 3865, DOI: 10.1103/Physrevlett.77.3865.

(S30) Grimme, S.; Ehrlich, S.; Goerigk, L. Effect of the damping function in dispersion corrected density functional theory. *Journal of computational chemistry* **2011**, *32*, 1456–1465, DOI: 10.1002/jcc.21759.

(S31) Goedecker, S.; Teter, M.; Hutter, J. Separable dual-space Gaussian pseudopotentials. *Physical Review B* **1996**, *54*, 1703, DOI: 10.1103/PhysRevB.54.1703.

(S32) Manz, T. A.; Sholl, D. S. Chemically Meaningful Atomic Charges That Reproduce the Electrostatic Potential in Periodic and Nonperiodic Materials. *J. Chem. Theory Comput.* **2010**, *6*, 2455–2468, DOI: 10.1021/ct100125x.

(S33) Boyd, P. G.; Chidambaram, A.; García-Díez, E.; Ireland, C. P.; Daff, T. D.; Bounds, R.; Gładysiak, A.; Schouwink, P.; Moosavi, S. M.; Maroto-Valer, M. M.; Reimer, J. A.; Navarro, J. A.; Woo, T. K.; Garcia, S.; Stylianou, K. C.; Smit, B. Data-driven design of metal–organic frameworks for wet flue gas CO_2 capture. *Nature* **2019**, *576*, 253–256, DOI: 10.1038/s41586-019-1798-7.

(S34) Park, H.; Majumdar, S.; Zhang, X.; Kim, J.; Smit, B. Inverse design of metal-organic frameworks for direct air capture of CO_2 via deep reinforcement learning. *ChemRxiv* **2023**, DOI: 10.26434/chemrxiv-2023-71mjq-v2.

(S35) Majumdar, S.; Moosavi, S. M.; Jablonka, K. M.; Ongari, D.; Smit, B. Diversifying Databases of Metal Organic Frameworks for High-Throughput Computational Screening. *ACS Appl. Mater. Interfaces* **2021**, *13*, 61004–61014, DOI: 10.1021/acsami.1c16220.

(S36) Boyd, P. G.; Woo, T. K. A generalized method for constructing hypothetical nanoporous materials of any net topology from graph theory. *CrystEngComm* **2016**, *18*, 3777–3792, DOI: 10.1039/c6ce00407e.

(S37) Lee, S.; Kim, B.; Cho, H.; Lee, H.; Lee, S. Y.; Cho, E. S.; Kim, J. Computational Screening of Trillions of Metal–Organic Frameworks for High-Performance Methane Storage. *ACS Appl. Mater. Interfaces* **2021**, *13*, 23647–23654.

(S38) Colón, Y. J.; Gómez-Gualdrón, D. A.; Snurr, R. Q. Topologically Guided, Automated Construction of Metal–Organic Frameworks and Their Evaluation for Energy-Related Applications. *Cryst. Growth Des.* **2017**, *17*, 5801–5810, DOI: 10.1021/acs.cgd.7b00848.

(S39) Rappé, A. K.; Casewit, C. J.; Colwell, K.; Goddard III, W. A.; Skiff, W. M. UFF, a full periodic table force field for molecular mechanics and molecular dynamics simulations. *Journal of the American chemical society* **1992**, *114*, 10024–10035, DOI: 10.1021/ja00051a040.

(S40) Ongari, D.; Boyd, P. G.; Barthel, S.; Witman, M.; Haranczyk, M.; Smit, B. Accurate characterization of the pore volume in microporous crystalline materials. *Langmuir* **2017**, *33*, 14529–14538, DOI: 10.1021/acs.langmuir.7b01682.

(S41) Frenkel, D.; Smit, B. *Understanding molecular simulation: from algorithms to applications*; Elsevier, 2001; Vol. 1.

(S42) Potoff, J. J.; Siepmann, J. I. Vapor–liquid equilibria of mixtures containing alkanes, carbon dioxide, and nitrogen. *AIChE journal* **2001**, *47*, 1676–1682, DOI: 10.1002/aic.690470719.

(S43) Abascal, J. L.; Vega, C. A general purpose model for the condensed phases of water: TIP4P/2005. *The Journal of chemical physics* **2005**, *123*, 234505, DOI: 10.1063/1.2121687.

(S44) Moubarak, E.; Moosavi, S. M.; Charalambous, C.; Garcia, S.; Smit, B. A Robust Framework for Generating Adsorption Isotherms to Screen Materials for Carbon Capture. *ChemRxiv* **2023**, DOI: 10.26434/chemrxiv-2023-hd568.

(S45) Myers, A. L.; Prausnitz, J. M. Thermodynamics of mixed-gas adsorption. *AIChE journal* **1965**, *11*, 121–127, DOI: 10.1002/aic.690110125.

(S46) Sarkisov, L.; Martin, R. L.; Haranczyk, M.; Smit, B. On the Flexibility of Metal-Organic Frameworks. *J. Am. Chem. Soc.* **2014**, *136*, 2228–2231, DOI: 10.1021/ja411673b.

(S47) Moosavi, S. M.; Boyd, P. G.; Sarkisov, L.; Smit, B. Improving the Mechanical Stability of Metal-Organic Frameworks Using Chemical Caryatids. *ACS Central Sci.* **2018**, *4*, 832–839, DOI: 10.1021/acscentsci.8b00157.

(S48) Witman, M.; Ling, S. L.; Jawahery, S.; Boyd, P. G.; Haranczyk, M.; Slater, B.; Smit, B. The Influence of Intrinsic Framework Flexibility on Adsorption in Nanoporous Materials. *J. Am. Chem. Soc.* **2017**, *139*, 5547–5557, DOI: 10.1021/jacs.7b01688.

(S49) Plimpton, S. Fast Parallel Algorithms for Short-Range Molecular-Dynamics. *J Comput Phys* **1995**, *117*, 1–19, DOI: DOI10.1006/jcph.1995.1039.

(S50) Addicoat, M. A.; Vankova, N.; Akter, I. F.; Heine, T. Extension of the Universal Force Field to Metal-Organic Frameworks. *J Chem Theory Comput* **2014**, *10*, 880–891, DOI: 10.1021/Ct400952t.

(S51) Young, J.; García-Díez, E.; Garcia, S.; Van Der Spek, M. The impact of binary water-CO_2 isotherm models on the optimal performance of sorbent-based direct air capture processes. *Energy and Environmental Science* **2021**, *14*, 5377–5394, DOI: 10.1039/d1ee01272j.

(S52) Santori, G.; Charalambous, C.; Ferrari, M. C.; Brandani, S. Adsorption artificial tree for atmospheric carbon dioxide capture, purification and compression. *Energy* **2018**, *162*, 1158–1168, DOI: 10.1016/j.energy.2018.08.090.

(S53) Douglas LeVan, M.; Croft, D. T. Determination of periodic states of pressure swing adsorption cycles. *Gas Separation & Purification* **1995**, *9*, 13–16, DOI: https://doi.org/10.1016/0950-4214(95)92171-8.

(S54) Chan, Y. N. I.; Hill, F. B.; Wong, Y. W. Equilibrium theory of a pressure swing adsorption process. *Chemical Engineering Science* **1981**, *36*, 243–251, DOI: https://doi.org/10.1016/0009-2509(81)85002-6.

(S55) Chlendi, M.; Tondeur, D. Dynamic behaviour of layered columns in pressure swing adsorption. *Gas Separation & Purification* **1995**, *9*, 231–242, DOI: https://doi.org/10.1016/0950-4214(95)00005-V.

(S56) Li, G.; Xiao, P.; Webley, P.; Zhang, J.; Singh, R.; Marshall, M. Capture of CO_2 from high humidity flue gas by vacuum swing adsorption with zeolite 13X. *Adsorption* **2008**, *14*, 415–422, DOI: 10.1007/s10450-007-9100-y.

(S57) Wilkins, N. S.; Sawada, J. A.; Rajendran, A. Measurement of competitive $CO_2$and H_2Oadsorption on zeolite 13X for post-combustion CO_2capture. *Adsorption* **2020**, *26*, 765–779.

(S58) Grande, C. A.; Blom, R.; Middelkoop, V.; Matras, D.; Vamvakeros, A.; Jacques, S. D.; Beale, A. M.; Di Michiel, M.; Anne Andreassen, K.; Bouzga, A. M. Multiscale investigation of adsorption properties of novel 3D printed UTSA-16 structures. *Chemical Engineering Journal* **2020**, *402*, 126166, DOI: https://doi.org/10.1016/j.cej.2020.126166.

(S59) Lin, J. B.; Nguyen, T. T. T.; Vaidhyanathan, R.; Burner, J.; Taylor, J. M.; Durekova, H.; Akhtar, F.; Mah, R. K.; Ghaffari-Nik, O.; Marx, S.; Fylstra, N.; Iremonger, S. S.; Dawson, K. W.; Sarkar, P.; Hovington, P.; Rajendran, A.; Woo, T. K.; Shimizu, G. K. H. A scalable metal-organic framework as a durable physisorbent for carbon dioxide capture. *Science* **2021**, *374*, 1464, DOI: 10.1126/science.abi7281.

(S60) Ribeiro, A. M.; Grande, C. A.; Lopes, F. V. S.; Loureiro, J. M.; Rodrigues, A. E. Four beds pressure swing adsorption for hydrogen purification: Case of humid feed and activated carbon beds. *AIChE Journal* **2009**, *55*, 2292–2302, DOI: https://doi.org/10.1002/aic.11886.

(S61) Rege, S. U.; Yang, R. T.; Qian, K.; Buzanowski, M. A. Air-prepurification by pressure swing adsorption using single/layered beds. *Chemical Engineering Science* **2001**, *56*, 2745–2759, DOI: https://doi.org/10.1016/S0009-2509(00)00531-5.

(S62) Hefti, M.; Mazzotti, M. Postcombustion CO_2 Capture from Wet Flue Gas by Temperature Swing Adsorption. *Industrial & Engineering Chemistry Research* **2018**, *57*, 15542–15555, DOI: 10.1021/acs.iecr.8b03580.

(S63) Li, G.; Xiao, P.; Zhang, J.; Webley, P. A.; Xu, D. The role of water on postcombustion CO_2 capture by vacuum swing adsorption: Bed layering and purge to feed ratio. *AIChE Journal* **2014**, *60*, 673–689, DOI: https://doi.org/10.1002/aic.14281.

(S64) Leperi, K. T.; Snurr, R. Q.; You, F. Optimization of Two-Stage Pressure/Vacuum Swing Adsorption with Variable Dehydration Level for Postcombustion Carbon Capture. *Industrial & Engineering Chemistry Research* **2016**, *55*, 3338–3350, DOI: 10.1021/acs.iecr.5b03122.

(S65) Ahn, H.; Lee, C.-H. Effects of capillary condensation on adsorption and thermal desorption dynamics of water in zeolite 13X and layered beds. *Chemical Engineering Science* **2004**, *59*, 2727–2743, DOI: https://doi.org/10.1016/j.ces.2004.04.011.

(S66) Wang, Y.; LeVan, M. D. Adsorption Equilibrium of Binary Mixtures of Carbon Dioxide and Water Vapor on Zeolites 5A and 13X. *Journal of Chemical & Engineering Data* **2010**, *55*, 3189–3195, DOI: 10.1021/je100053g.

(S67) Charalambous, C. Temperature swing adsorption process for carbon dioxide capture, purification and compression directly from atmospheric air. 2018; https://www.era.lib.ed.ac.uk/handle/1842/33311.

(S68) Hall, S. *Rules of Thumb for Chemical Engineers*; Elsevier: Oxford, UK, 2012.

(S69) Branan, C. R. *Rules of Thumb for Chemical Engineers*; Gulf Professional Publishing, 2005; pp 112–122.

(S70) Ajenifuja, A.; Joss, L.; Jobson, M. A New Equilibrium Shortcut Temperature Swing Adsorption Model for Fast Adsorbent Screening. *Ind. Eng. Chem. Res* **2020**, *59*, 3485–3497, DOI: 10.1021/acs.iecr.9b05579.

(S71) Siemens Process Systems Engineering gPROMS. www.psenterprise.com/, 1997-2023.

(S72) AspenTech Aspen Capital Cost Estimator | CAPEX Estimation | AspenTech. 2022; https://www.aspentech.com/en/products/engineering/aspen-capital-cost-estimator.

(S73) Woods, D. R. *Rules of Thumb in Engineering Practice*; Wiley-VCH Verlag GmbH & Co. KGaA, 2007; pp 37–72.

(S74) Rubin, E. S.; Berghout, N.; Booras, G.; Fout, T.; Garcia, M.; Mohd Nazir, S.; Ramirez, A.; Roussanaly, S.; Van Der Spek, M. Toward improved cost guidelines for advanced low-carbon technologies. 15th International Conference on Greenhouse Gas Control Technologies, GHGT-15. 2021; p 12, DOI: 10.2139/ssrn.3818896.

(S75) EBTF European Benchmarking Task Force. 2018; https://zenodo.org/communities/ebtf/?page=1&size=20.

(S76) Danaci, D.; Bui, M.; Dowell, N. M.; Petit, C. Exploring the limits of adsorption-based CO_2 capture using MOFs with PVSA-from molecular design to process economics. *Mol. Syst. Des. Eng.* **2020**, *5*, 35, DOI: 10.1039/c9me00102f.

(S77) Severino, M. I.; Gkaniatsou, E.; Nouar, F.; Pinto, M. L.; Serre, C. MOFs industrialization: a complete assessment of production costs. *Faraday Discuss.* **2021**, *231*, 326–341.

(S78) Booth, N. D.2.3.4 Economic baseline study for newly defined power plants. 2010; https://co2cesar.eu/site/en/downloads.php.

(S79) Varbanov, P.; Doyle, S.; Smith, R. Modelling and Optimization of Utility Systems. *Chemical Engineering Research and Design* **2004**, *82*, 561–578, DOI: https://doi.org/10.1205/026387604323142603.

(S80) Gottlicher, G. The energetics of carbon dioxide capture in power plants. 2004.

(S81) Rost, M.; Reinhold, M.; Getze, D. CESAR D.2.3.2 Generic CO_2 compression study. 2009; https://co2cesar.eu/site/en/downloads.php.

(S82) Sathre, R.; Chester, M.; Cain, J.; Masanet, E. A framework for environmental assessment of CO_2 capture and storage systems. *Energy* **2012**, *37*, 540–548, DOI: 10.1016/J.Energy.2011.10.050.

(S83) von der Assen, N.; Bardow, A. Life cycle assessment of polyols for polyurethane production using CO_2 as feedstock: insights from an industrial case study. *Green Chemistry* **2014**, *16*, 3272–3280, DOI: 10.1039/C4GC00513A.

(S84) Reuter, M.; Hudson, C.; Van Schaik, A.; Heiskanen, K.; Meskers, C.; Hagelüken, C.; others Metal recycling: opportunities, limits, infrastructure. 2013; https://www.resourcepanel.org/file/313/download?token=JPyZF5_Q.

(S85) Frischknecht, R.; Jungbluth, N.; Althaus, H.-J.; Hischier, R.; Doka, G.; Dones, R.; Heck, T.; Hellweg, S.; Wernet, G.; Nemecek, T.; others Overview and methodology. Data v2. 0 (2007). Ecoinvent report No. 1. 2007; https://ecoinvent.org/wp-content/uploads/2020/08/200712_frischknecht_jungbluth_overview_methodology_ecoinvent2.pdf.

(S86) Jablonka, K. M.; Rosen, A. S.; Krishnapriyan, A. S.; Smit, B. An ecosystem for digital reticular chemistry. *ACS Central Science* **2023**, *9*, 563—581, DOI: 10.1021/acscentsci.2c01177.

(S87) Kim, S.; Chen, J.; Cheng, T.; Gindulyte, A.; He, J.; He, S.; Li, Q.; Shoemaker, B. A.; Thiessen, P. A.; Yu, B.; Zaslavsky, L.; Zhang, J.; Bolton, E. E. PubChem in 2021: new data content and improved web interfaces. *Nucleic Acids Research* **2020**, *49*, D1388–D1395, DOI: 10.1093/nar/gkaa971.

(S88) Sathre, R.; Masanet, E. Prospective life-cycle modeling of a carbon capture and storage system using metal–organic frameworks for CO_2 capture. *RSC advances* **2013**, *3*, 4964–4975, DOI: 10.1039/C3RA40265G.

(S89) Hu, J.; Gu, X.; Lin, L.-C.; Bakshi, B. R. Toward Sustainable Metal-Organic Frameworks for Post-Combustion Carbon Capture by Life Cycle Assessment and Molecular Simulation. *ACS Sustainable Chemistry & Engineering* **2021**, *9*, 12132–12141, DOI: 10.1021/acssuschemeng.1c03473.

(S90) Park, H.; Kang, Y.; Choe, W.; Kim, J. Mining insights on metal–organic framework synthesis from scientific literature texts. *Journal of Chemical Information and Modeling* **2022**, *62*, 1190–1198.

(S91) Rehner, P.; Bauer, G.; Gross, J. FeOs: An Open-Source Framework for Equations of State and Classical Density Functional Theory. *Industrial & Engineering Chemistry Research* **2023**, *62*, 5347–5357.

(S92) Winter, B.; Winter, C.; Schilling, J.; Bardow, A. A smile is all you need: predicting limiting activity coefficients from SMILES with natural language processing. *Digital Discovery* **2022**, *1*, 859–869.

(S93) Winter, B.; Winter, C.; Esper, T.; Schilling, J.; Bardow, A. SPT-NRTL: A physics-guided machine learning model to predict thermodynamically consistent activity coefficients. *Fluid Phase Equilibria* **2023**, *568*, 113731.

(S94) Doka, G. Life cycle inventories of waste treatment services. 2003; https://www.doka.ch/13_I_WasteTreatmentGeneral.pdf.

(S95) Doka, G. Updates to Life Cycle Inventories of Waste Treatment Services-part II: waste incineration. 2013; http://www.lcaforum.ch/inventories/Hintergrund/Doka_2013-ecoinvent_MSWI_updateLCI.pdf.

(S96) Meys, R.; Kätelhön, A.; Bachmann, M.; Winter, B.; Zibunas, C.; Suh, S.; Bardow, A. Achieving net-zero greenhouse gas emission plastics by a circular carbon economy. *Science* **2021**, *374*, 71–76, DOI: 10.1126/science.abg9853.

(S97) European Commission - Joint Research Centre (EC-JRC) Environmental Footprint reference package 3.0 (EF 3.0). 2018; https://eplca.jrc.ec.europa.eu/LCDN/developerEF.xhtml, (Accessed 2022 June).

(S98) Sala, S.; De Laurentiis, V.; Zampori, L.; Diaconu, E.; Fazio, S.; Biganzioli, F.; (European Commission and Joint Research Centre) Supporting information to the characterisation factors of recommended EF Life Cycle Impact Assessment methods: version 2, from ILCD to EF 3.0. **2019**, DOI: doi/10.2760/002447.

(S99) Wernet, G.; Bauer, C.; Steubing, B.; Reinhard, J.; Moreno-Ruiz, E.; Weidema, B. The ecoinvent database version 3 (part I): overview and methodology. *The International Journal of Life Cycle Assessment* **2016**, *21*, 1218–1230, DOI: 10.1007/s11367-016-1087-8.

(S100) Reinert, C.; Deutz, S.; Minten, H.; Dörpinghaus, L.; von Pfingsten, S.; Baumgärtner, N.; Bardow, A. Environmental impacts of the future German energy system from integrated energy systems optimization and dynamic life cycle assessment. *Computers & Chemical Engineering* **2021**, *153*, 107406, DOI: 10.1016/j.compchemeng.2021.107406.

(S101) Singh, B.; Strømman, A. H.; Hertwich, E. Life cycle assessment of natural gas combined cycle power plant with post-combustion carbon capture, transport and storage. *International Journal of Greenhouse Gas Control* **2011**, *5*, 457–466, DOI: 10.1016/j.ijggc.2010.03.006.

(S102) Koornneef, J.; van Keulen, T.; Faaij, A.; Turkenburg, W. Life cycle assessment of a pulverized coal power plant with post-combustion capture, transport and storage of CO2. *International journal of greenhouse gas control* **2008**, *2*, 448–467, DOI: 10.1016/j.ijggc.2008.06.008.

(S103) Moser, P.; Wiechers, G.; Schmidt, S.; Garcia Moretz-Sohn Monteiro, J.; Charalambous, C.; Garcia, S.; Sanchez Fernandez, E. Results of the 18-month test with MEA at the post-combustion capture pilot plant at Niederaussem – new impetus to solvent management, emissions and dynamic behaviour. *International Journal of Greenhouse Gas Control* **2020**, *95*, 102945, DOI: 10.1016/j.ijggc.2019.102945.

(S104) Cadiau, A.; Lee, J. S.; Borges, D. D.; Fabry, P.; Devic, T.; Wharmby, M. T.; Martineau, C.; Foucher, D.; Taulelle, F.; Jun, C. H.; Hwang, Y. K.; Stock, N.; De Lange, M. F.; Kapteijn, F.; Gascon, J.; Maurin, G.; Chang, J. S.; Serre, C. Design of Hydrophilic Metal Organic Framework Water Adsorbents for Heat Reallocation. *Adv Mater* **2015**, *27*, 4775–4780, DOI: 10.1002/adma.201502418.

(S105) Permyakova, A.; Skrylnyk, O.; Courbon, E.; Affram, M.; Wang, S.; Lee, U.-H.; Valekar, A. H.; Nouar, F.; Mouchaham, G.; Devic, T.; De Weireld, G.; Chang, J.-S.;

Steunou, N.; Frère, M.; Serre, C. Synthesis optimization, shaping, and heat reallocation evaluation of the hydrophilic metal-organic framework MIL-160(Al). *ChemSusChem* **2017**, *10*, 1419–1426.

(S106) Shade, D.; Marszalek, B.; Walton, K. S. Structural similarity, synthesis, and adsorption properties of aluminum-based metal-organic frameworks. *Adsorption* **2021**, *27*, 227–236, DOI: 10.1007/s10450-020-00282-9.

(S107) Damasceno Borges, D.; Normand, P.; Permiakova, A.; Babarao, R.; Heymans, N.; Galvao, D. S.; Serre, C.; De Weireld, G.; Maurin, G. Gas adsorption and separation by the Al-based metal–organic framework MIL-160. *The Journal of Physical Chemistry C* **2017**, *121*, 26822–26832, DOI: 10.1021/acs.jpcc.7b08856.

(S108) Silva, M.; Ribeiro, A.; Silva, C.; Nogueira, I.; Cho, K.-H.; Lee, U.-H.; Faria, J.; Loureiro, J.; Chang, J.-S.; Rodrigues, A.; others MIL-160 (Al) MOF's potential in adsorptive water harvesting. *Adsorption* **2021**, *27*, 213–226, DOI: 10.1007/s10450-020-00286-5.

(S109) Wang, S.; Lee, J. S.; Wahiduzzaman, M.; Park, J.; Muschi, M.; Martineau-Corcos, C.; Tissot, A.; Cho, K. H.; Marrot, J.; Shepard, W.; Maurin, G.; Chang, J.-S.; Serre, C. A robust large-pore zirconium carboxylate metal–organic framework for energy-efficient water-sorption-driven refrigeration. *Nature Energy* **2018**, *3*, 985–993, DOI: 10.1038/s41560-018-0261-6.

(S110) Wahiduzzaman, M.; Lenzen, D.; Maurin, G.; Stock, N.; Wharmby, M. T. Rietveld Refinement of MIL-160 and Its Structural Flexibility Upon$H_2O$and N_2 Adsorption. *European Journal of Inorganic Chemistry* **2018**, *2018*, 3626–3632, DOI: 10.1002/ejic.201800323.

(S111) Loiseau, T.; Lecroq, L.; Volkringer, C.; Marrot, J.; Férey, G.; Haouas, M.; Taulelle, F.; Bourrelly, S.; Llewellyn, P. L.; Latroche, M. MIL-96, a porous aluminum

trimesate 3D structure constructed from a hexagonal network of 18-membered rings and μ-oxo-centered trinuclear units. *J Am Chem Soc* **2006**, *128*, 10223–10230, DOI: 10.1021/ja0621086.

(S112) Benzaqui, M.; Pillai, R. S.; Sabetghadam, A.; Benoit, V.; Normand, P.; Marrot, J.; Menguy, N.; Montero, D.; Shepard, W.; Tissot, A.; Martineau-Corcos, C.; Sicard, C.; Mihaylov, M.; Carn, F.; Beurroies, I.; Llewellyn, P. L.; De Weireld, G.; Hadjiivanov, K.; Gascon, J.; Kapteijn, F.; Maurin, G.; Steunou, N.; Serre, C. Revisiting the aluminum trimesate-based MOF (MIL-96): From structure determination to the processing of mixed matrix membranes for CO2 capture. *Chem. Mater.* **2017**, *29*, 10326–10338.

(S113) Liu, D.; Liu, Y.; Dai, F.; Zhao, J.; Yang, K.; Liu, C. Size-and morphology-controllable synthesis of MIL-96 (Al) by hydrolysis and coordination modulation of dual aluminium source and ligand systems. *Dalton Transactions* **2015**, *44*, 16421–16429, DOI: 10.1039/c5dt02379c.

(S114) Reinsch, H.; van der Veen, M. A.; Gil, B.; Marszalek, B.; Verbiest, T.; de Vos, D.; Stock, N. Structures, Sorption Characteristics, and Nonlinear Optical Properties of a New Series of Highly Stable Aluminum MOFs. *Chem Mater* **2013**, *25*, 17–26, DOI: 10.1021/cm3025445.

(S115) Pei, J.; Wen, H.-M.; Gu, X.-W.; Qian, Q.-L.; Yang, Y.; Cui, Y.; Li, B.; Chen, B.; Qian, G. Dense Packing of Acetylene in a Stable and Low-Cost Metal–Organic Framework for Efficient C2H2/CO2 Separation. *Angewandte Chemie International Edition* **2021**, *60*, 25068–25074, DOI: 10.1002/anie.202110820.

(S116) Wiersum, A. D.; Giovannangeli, C.; Vincent, D.; Bloch, E.; Reinsch, H.; Stock, N.; Lee, J. S.; Chang, J.-S.; Llewellyn, P. L. Experimental screening of porous materials for high pressure gas adsorption and evaluation in gas separations: application to

MOFs (MIL-100 and CAU-10). *ACS Combinatorial Science* **2013**, *15*, 111–119, DOI: 10.1021/co300128w.

(S117) Millange, F.; Serre, C.; Férey, G. Synthesis, structure determination and properties of MIL-53as and MIL-53ht:: the first Cr hybrid inorganic-organic microporous solids:: Cr(OH)$\cdot OC - CH - CO.HOC - CH - COH$. *Chem Commun* **2002**, 822–823, DOI: 10.1039/b201381a.

(S118) Devic, T.; Horcajada, P.; Serre, C.; Salles, F.; Maurin, G.; Moulin, B.; Heurtaux, D.; Clet, G.; Vimont, A.; Grenèche, J.-M.; Le Ouay, B.; Moreau, F.; Magnier, E.; Filinchuk, Y.; Marrot, J.; Lavalley, J.-C.; Daturi, M.; Férey, G. Functionalization in flexible porous solids: effects on the pore opening and the host-guest interactions. *J. Am. Chem. Soc.* **2010**, *132*, 1127–1136.

(S119) Ferey, G.; Serre, C. Large breathing effects in three-dimensional porous hybrid matter: facts, analyses, rules and consequences. *Chem. Soc. Rev.* **2009**, *38*, 1380–1399, DOI: 10.1039/b804302g.

(S120) Couck, S.; Denayer, J. F. M.; Baron, G. V.; Rémy, T.; Gascon, J.; Kapteijn, F. An amine-functionalized MIL-53 metal-organic framework with large separation power for CO2 and CH4. *J. Am. Chem. Soc.* **2009**, *131*, 6326–6327.

(S121) Serra-Crespo, P.; Berger, R.; Yang, W.; Gascon, J.; Kapteijn, F. Separation of CO2/CH4 mixtures over NH2-MIL-53—An experimental and modelling study. *Chemical Engineering Science* **2015**, *124*, 96–108, DOI: 10.1016/j.ces.2014.10.028.

(S122) Martinez Joaristi, A.; Juan-Alcañiz, J.; Serra-Crespo, P.; Kapteijn, F.; Gascon, J. Electrochemical synthesis of some archetypical Zn2+, Cu2+, and Al3+ metal organic frameworks. *Crystal Growth & Design* **2012**, *12*, 3489–3498, DOI: 10.1021/cg300552w.

(S123) Couck, S.; Denayer, J. F.; Baron, G. V.; Rémy, T.; Gascon, J.; Kapteijn, F. An amine-functionalized MIL-53 metal- organic framework with large separation power for CO_2 and CH4. *Journal of the American Chemical Society* **2009**, *131*, 6326–6327, DOI: 10.1021/Ja900555r.

(S124) Garcia-Perez, E.; Serra-Crespo, P.; Hamad, S.; Kapteijn, F.; Gascon, J. Molecular simulation of gas adsorption and diffusion in a breathing MOF using a rigid force field. *Physical Chemistry Chemical Physics* **2014**, *16*, 16060–16066, DOI: 10.1039/C3cp55416c.

(S125) Jeremias, F.; Fröhlich, D.; Janiak, C.; Henninger, S. K. Advancement of sorption-based heat transformation by a metal coating of highly-stable, hydrophilic aluminium fumarate MOF. *RSC Adv.* **2014**, *4*, 24073–24082, DOI: 10.1039/c4ra03794d.

(S126) Teo, H. W. B.; Chakraborty, A.; Kitagawa, Y.; Kayal, S. Experimental study of isotherms and kinetics for adsorption of water on Aluminium Fumarate. *International Journal of Heat and Mass Transfer* **2017**, *114*, 621–627, DOI: 10.1016/j.ijheatmasstransfer.2017.06.086.

(S127) Gaab, M.; Trukhan, N.; Maurer, S.; Gummaraju, R.; Müller, U. The progression of Al-based metal-organic frameworks – From academic research to industrial production and applications. *Microporous and Mesoporous Materials* **2012**, *157*, 131–136, DOI: 10.1016/j.micromeso.2011.08.016.

(S128) Bozbiyik, B.; Van Assche, T.; Lannoeye, J.; De Vos, D. E.; Baron, G. V.; Denayer, J. F. M. Stepped water isotherm and breakthrough curves on aluminium fumarate metal–organic framework: experimental and modelling study. *Adsorption* **2016**, *23*, 185–192, DOI: 10.1007/s10450-016-9847-0.

(S129) Wöllner, M.; Klein, N.; Kaskel, S. Measuring water adsorption processes of metal-organic frameworks for heat pump applications via optical calorimetry. *Microporous*

and Mesoporous Materials **2019**, *278*, 206–211, DOI: 10.1016/j.micromeso.2018.11.024.

(S130) Coelho, J. A.; Ribeiro, A. M.; Ferreira, A. F. P.; Lucena, S. M. P.; Rodrigues, A. E.; Azevedo, D. C. S. d. Stability of an Al-Fumarate MOF and Its Potential for CO_2 Capture from Wet Stream. *Industrial & Engineering Chemistry Research* **2016**, *55*, 2134–2143, DOI: 10.1021/acs.iecr.5b03509.

(S131) Yang, S. H.; Sun, J. L.; Ramirez-Cuesta, A. J.; Callear, S. K.; David, W. I. F.; Anderson, D. P.; Newby, R.; Blake, A. J.; Parker, J. E.; Tang, C. C.; Schröder, M. Selectivity and direct visualization of carbon dioxide and sulfur dioxide in a decorated porous host. *Nat Chem* **2012**, *4*, 887–894, DOI: 10.1038/Nchem.1457.

(S132) Nguyen, T. T.; Lin, J.-B.; Shimizu, G. K.; Rajendran, A. Separation of CO_2 and N_2 on a hydrophobic metal organic framework CALF-20. *Chemical Engineering Journal* **2022**, *442*, 136263, DOI: 10.1016/j.cej.2022.136263.

(S133) Nguyen, T. T.; Shimizu, G. K.; Rajendran, A. CO_2 /N_2 separation by vacuum swing adsorption using a metal–organic framework, CALF-20: Multi-objective optimization and experimental validation. *Chemical Engineering Journal* **2023**, *452*, 139550, DOI: 10.1016/j.cej.2022.136263.

(S134) Rosi, N. L.; Kim, J.; Eddaoudi, M.; Chen, B. L.; O'Keeffe, M.; Yaghi, O. M. Rod packings and metal-organic frameworks constructed from rod-shaped secondary building units. *J. Am. Chem. Soc.* **2005**, *127*, 1504–1518, DOI: 10.1021/ja045123o.

(S135) Dietzel, P. D. C.; Besikiotis, V.; Blom, R. Application of metal-organic frameworks with coordinatively unsaturated metal sites in storage and separation of methane and carbon dioxide. *J. Mater. Chem.* **2009**, *19*, 7362–7370, DOI: 10.1039/b911242a.

(S136) Caskey, S. R.; Wong-Foy, A. G.; Matzger, A. J. Dramatic tuning of carbon dioxide uptake via metal substitution in a coordination polymer with cylindrical pores. *J Am Chem Soc* **2008**, *130*, 10870, DOI: 10.1021/Ja8036096.

(S137) Bloch, E. D.; Murray, L. J.; Queen, W. L.; Chavan, S.; Maximoff, S. N.; Bigi, J. P.; Krishna, R.; Peterson, V. K.; Grandjean, F.; Long, G. J.; Smit, B.; Bordiga, S.; Brown, C. M.; Long, J. R. Selective binding of O_2 over N_2 in a redox-active metal-organic framework with open iron(II) coordination sites. *J. Am. Chem. Soc.* **2011**, *133*, 14814–22, DOI: 10.1021/ja205976v.

(S138) Yu, D. C.; Yazaydin, A. O.; Lane, J. R.; Dietzel, P. D. C.; Snurr, R. Q. A combined experimental and quantum chemical study of CO_2 adsorption in the metal-organic framework CPO-27 with different metals. *Chem Sci* **2013**, *4*, 3544–3556, DOI: 10.1039/c3sc51319j.

(S139) Queen, W. L.; Hudson, M. R.; Bloch, E. D.; Mason, J. A.; Gonzalez, M. I.; Lee, J. S.; Gygi, D.; Howe, J. D.; Lee, K.; Darwish, T. A.; James, M.; Peterson, V. K.; Teat, S. J.; Smit, B.; Neaton, J. B.; Long, J. R.; Brown, C. M. Comprehensive study of carbon dioxide adsorption in the metal-organic frameworks M-2(dobdc) (M = Mg, Mn, Fe, Co, Ni, Cu, Zn). *Chem. Sci.* **2014**, *5*, 4569–4581, DOI: 10.1039/c4sc02064b.

(S140) Dzubak, A.; Lin, L.-C.; Kim, J.; Swisher, J. A.; Poloni, R.; Maximoff, S. N.; Smit, B.; Gagliardi, L. Ab-initio Carbon Capture in Open-Site Metal Organic Frameworks. *Nat Chem* **2012**, *4*, 810–816, DOI: 10.1038/NCHEM.1432.

(S141) Yang, C.; Wang, X. P.; Omary, M. A. Fluorous metal-organic frameworks for high-density gas adsorption. *J Am Chem Soc* **2007**, *129*, 15454–+, DOI: 10.1021/ja0775265.

(S142) Moghadam, P. Z.; Ivy, J. F.; Arvapally, R. K.; Dos Santos, A. M.; Pearson, J. C.; Zhang, L.; Tylianakis, E.; Ghosh, P.; Oswald, I. W.; Kaipa, U.; others Adsorption

and molecular siting of CO_2 , water, and other gases in the superhydrophobic, flexible pores of FMOF-1 from experiment and simulation. *Chemical Science* **2017**, *8*, 3989–4000, DOI: 10.1039/C7SC00278E.

(S143) Ma, S. Q.; Sun, D. F.; Simmons, J. M.; Collier, C. D.; Yuan, D. Q.; Zhou, H. C. Metal-organic framework from an anthracene derivative containing nanoscopic cages exhibiting high methane uptake. *J Am Chem Soc* **2008**, *130*, 1012–1016, DOI: 10.1021/Ja0771639.

(S144) Perry IV, J. J.; Teich-McGoldrick, S. L.; Meek, S. T.; Greathouse, J. A.; Haranczyk, M.; Allendorf, M. D. Noble gas adsorption in metal–organic frameworks containing open metal sites. *The Journal of Physical Chemistry C* **2014**, *118*, 11685–11698, DOI: 10.1021/jp501495f.

(S145) Parkes, M. V.; Staiger, C. L.; Perry IV, J. J.; Allendorf, M. D.; Greathouse, J. A. Screening metal–organic frameworks for selective noble gas adsorption in air: effect of pore size and framework topology. *Physical Chemistry Chemical Physics* **2013**, *15*, 9093–9106, DOI: 10.1039/c3cp50774b.

(S146) Cavka, J. H.; Jakobsen, S.; Olsbye, U.; Guillou, N.; Lamberti, C.; Bordiga, S.; Lillerud, K. P. A New Zirconium Inorganic Building Brick Forming Metal Organic Frameworks with Exceptional Stability. *J. Am. Chem. Soc.* **2008**, *130*, 13850–13851, DOI: 10.1021/ja8057953.

(S147) Hu, Z. G.; Faucher, S.; Zhuo, Y. Y.; Sun, Y.; Wang, S. N.; Zhao, D. Combination of Optimization and Metalated-Ligand Exchange: An Effective Approach to Functionalize UiO-66(Zr) MOFs for CO_2 Separation. *Chem-Eur J* **2015**, *21*, 17245–17255, DOI: 10.1002/chem.201503078.

(S148) Andersen, A.; Divekar, S.; Dasgupta, S.; Cavka, J. H.; Nanoti, A.; Spjelkavik, A.; Goswami, A. N.; Garg, M.; Blom, R.; others On the development of Vacuum Swing

adsorption (VSA) technology for post-combustion CO_2 capture. *Energy Procedia* **2013**, *37*, 33–39, DOI: 10.1016/j.egypro.2013.05.082.

(S149) Hu, Z. G.; Peng, Y. W.; Kang, Z. X.; Qian, Y. H.; Zhao, D. A Modulated Hydrothermal (MHT) Approach for the Facile Synthesis of UiO66-Type MOFs. *Inorg Chem* **2015**, *54*, 4862–4868, DOI: 10.1021/acs.inorgchem.5b00435.

(S150) Cmarik, G. E.; Kim, M.; Cohen, S. M.; Walton, K. S. Tuning the adsorption properties of UiO-66 via ligand functionalization. *Langmuir* **2012**, *28*, 15606–15613, DOI: 10.1021/la3035352.

(S151) Zhang, W. J.; Huang, H. L.; Zhong, C. L.; Liu, D. H. Cooperative effect of temperature and linker functionality on CO_2 capture from industrial gas mixtures in metal-organic frameworks: a combined experimental and molecular simulation study. *Phys Chem Chem Phys* **2012**, *14*, 2317–2325, DOI: 10.1039/c2cp23839j.

(S152) Cao, Y.; Zhao, Y. X.; Lv, Z. J.; Song, F. J.; Zhong, Q. Preparation and enhanced CO_2 adsorption capacity of UiO-66/graphene oxide composites. *J Ind Eng Chem* **2015**, *27*, 102–107, DOI: 10.1016/j.jiec.2014.12.021.

(S153) Xian, S. K.; Wu, Y.; Wu, J. L.; Wang, X.; Xiao, J. Enhanced Dynamic CO_2 Adsorption Capacity and CO_2 /CH_4 Selectivity on Polyethylenimine-Impregnated UiO-66. *Ind Eng Chem Res* **2015**, *54*, 11151–11158, DOI: 10.1021/acs.iecr.5b03517.

(S154) Jasuja, H.; Zang, J.; Sholl, D. S.; Walton, K. S. Rational tuning of water vapor and CO_2 adsorption in highly stable Zr-based MOFs. *The Journal of Physical Chemistry C* **2012**, *116*, 23526–23532, DOI: 10.1021/Jp308657x.

(S155) Li, L.-J.; Liao, P.-Q.; He, C.-T.; Wei, Y.-S.; Zhou, H.-L.; Lin, J.-M.; Li, X.-Y.; Zhang, J.-P. Grafting alkylamine in UiO-66 by charge-assisted coordination bonds for carbon dioxide capture from high-humidity flue gas. *Journal of Materials Chemistry A* **2015**, *3*, 21849–21855, DOI: 10.1039/c5ta05997f.

(S156) Li, H.; Eddaoudi, M.; O'Keeffe, M.; Yaghi, O. M. Design and synthesis of an exceptionally stable and highly porous metal-organic framework. *Nature* **1999**, *402*, 276–279, DOI: 10.1038/46248.

(S157) Kloutse, F.; Hourri, A.; Natarajan, S.; Benard, P.; Chahine, R. Experimental benchmark data of CH4, CO_2 and N_2 binary and ternary mixtures adsorption on MOF-5. *Separation and Purification Technology* **2018**, *197*, 228–236, DOI: 10.1016/j.seppur.2018.01.013.

(S158) Saha, D.; Bao, Z.; Jia, F.; Deng, S. Adsorption of CO2, CH4, N_2 O, and N_2 on MOF-5, MOF-177, and zeolite 5A. *Environmental science & technology* **2010**, *44*, 1820–1826, DOI: 10.1021/Es9032309.

(S159) Marco-Lozar, J. P.; Juan-Juan, J.; Suárez-García, F.; Cazorla-Amorós, D.; Linares-Solano, A. MOF-5 and activated carbons as adsorbents for gas storage. *Int J Hydrogen Energ* **2012**, *37*, 2370–2381, DOI: 10.1016/j.ijhydene.2011.11.023.

(S160) Choi, J. S.; Son, W. J.; Kim, J.; Ahn, W. S. Metal-organic framework MOF-5 prepared by microwave heating: Factors to be considered. *Micropor Mesopor Mat* **2008**, *116*, 727–731, DOI: 10.1016/j.micromeso.2008.04.033.

(S161) Zhao, Z.; Li, Z.; Lin, Y. Adsorption and diffusion of carbon dioxide on metal- organic framework (MOF-5). *Industrial & Engineering Chemistry Research* **2009**, *48*, 10015–10020, DOI: 10.1021/Ie900665f.

(S162) Millward, A. R.; Yaghi, O. M. Metal- organic frameworks with exceptionally high capacity for storage of carbon dioxide at room temperature. *Journal of the American Chemical Society* **2005**, *127*, 17998–17999, DOI: 10.1021/ja0570032.

(S163) Furukawa, H.; Ko, N.; Go, Y. B.; Aratani, N.; Choi, S. B.; Choi, E.; Yazaydin, A. O.; Snurr, R. Q.; O'Keeffe, M.; Kim, J.; Yaghi, O. M. Ultrahigh Porosity in Metal-Organic Frameworks. *Science* **2010**, *329*, 424–428, DOI: 10.1126/science.1192160.

(S164) Sim, J.; Yim, H.; Ko, N.; Choi, S. B.; Oh, Y.; Park, H. J.; Park, S.; Kim, J. Gas adsorption properties of highly porous metal–organic frameworks containing functionalized naphthalene dicarboxylate linkers. *Dalton Transactions* **2014**, *43*, 18017–18024, DOI: 10.1039/C4dt02300e.

(S165) Kandiah, M.; Usseglio, S.; Svelle, S.; Olsbye, U.; Lillerud, K. P.; Tilset, M. Post-synthetic modification of the metal-organic framework compound UiO-66. *J Mater Chem* **2010**, *20*, 9848–9851, DOI: 10.1039/c0jm02416c.

(S166) Kandiah, M.; Nilsen, M. H.; Usseglio, S.; Jakobsen, S.; Olsbye, U.; Tilset, M.; Larabi, C.; Quadrelli, E. A.; Bonino, F.; Lillerud, K. P. Synthesis and Stability of Tagged UiO-66 Zr-MOFs. *Chem Mater* **2010**, *22*, 6632–6640, DOI: 10.1021/cm102601v.

(S167) Yoon, M.; Moon, D. New Zr (IV) based metal-organic framework comprising a sulfur-containing ligand: Enhancement of CO_2 and H_2 storage capacity. *Micropor Mesopor Mat* **2015**, *215*, 116–122, DOI: 10.1016/j.micromeso.2015.05.030.

(S168) Chui, S. S.-Y.; Lo, S. M.-F.; Charmant, J. P. H.; Orpen, A. G.; Williams, I. D. A Chemically Functionalizable Nanoporous Material. *Science* **1999**, *283*, 1148–1150, DOI: 10.1126/science.283.5405.1148.

(S169) Moosavi, S. M.; Chidambaram, A.; Talirz, L.; Haranczyk, M.; Stylianou, K. C.; Smit, B. Capturing chemical intuition in synthesis of metal-organic frameworks. *Nat. Commun.* **2019**, *10*, 539, DOI: 10.1038/s41467-019-08483-9.

(S170) Rother, J.; Fieback, T. Multicomponent adsorption measurements on activated carbon, zeolite molecular sieve and metal–organic framework. *Adsorption* **2013**, *19*, 1065–1074, DOI: 10.1007/s10450-013-9527-2.

(S171) Rios, R. B.; Correia, L. S.; Bastos-Neto, M.; Torres, A. E. B.; Hatimondi, S. A.; Ribeiro, A. M.; Rodrigues, A. E.; Cavalcante, C. L.; de Azevedo, D. C. Evaluation of

carbon dioxide–nitrogen separation through fixed bed measurements and simulations. *Adsorption* **2014**, *20*, 945–957, DOI: 10.1007/s10450-014-9639-3.

(S172) Al-Janabi, N.; Hill, P.; Torrente-Murciano, L.; Garforth, A.; Gorgojo, P.; Siperstein, F.; Fan, X. Mapping the Cu-BTC metal–organic framework (HKUST-1) stability envelope in the presence of water vapour for CO_2 adsorption from flue gases. *Chemical Engineering Journal* **2015**, *281*, 669–677, DOI: 10.1016/j.cej.2015.07.020.

(S173) Liang, Z.; Marshall, M.; Chaffee, A. L. Comparison of Cu-BTC and zeolite 13X for adsorbent based CO_2 separation. *Energy Procedia* **2009**, *1*, 1265–1271, DOI: 10.1016/j.egypro.2009.01.166.

(S174) Carné-Sánchez, A.; Stylianou, K. C.; Carbonell, C.; Naderi, M.; Imaz, I.; Maspoch, D. Protecting metal–organic framework crystals from hydrolytic degradation by spray-dry encapsulating them into polystyrene microspheres. *Advanced Materials* **2015**, *27*, 869–873, DOI: 10.1002/adma.201403827.

(S175) Yazaydın, A. O.; Benin, A. I.; Faheem, S. A.; Jakubczak, P.; Low, J. J.; Willis, R. R.; Snurr, R. Q. Enhanced CO_2 adsorption in metal-organic frameworks via occupation of open-metal sites by coordinated water molecules. *Chemistry of Materials* **2009**, *21*, 1425–1430, DOI: 10.1021/cm900049x.

(S176) Koh, K.; Wong-Foy, A. G.; Matzger, A. J. A crystalline mesoporous coordination copolymer with high microporosity. *Angew Chem Int Edit* **2008**, *47*, 677–680, DOI: 10.1002/anie.200705020.

(S177) Mu, B.; Schoenecker, P. M.; Walton, K. S. Gas Adsorption Study on Mesoporous Metal-Organic Framework UMCM-1. *J Phys Chem C* **2010**, *114*, 6464–6471, DOI: 10.1021/jp906417z.

(S178) Xiang, Z.; Peng, X.; Cheng, X.; Li, X.; Cao, D. CNT@ Cu3 (BTC) 2 and metal–organic frameworks for separation of CO2/CH4 mixture. *The Journal of Physical Chemistry C* **2011**, *115*, 19864–19871, DOI: 10.1021/jp206959k.

(S179) Li, L. Y.; Guo, L. D.; Pu, S. Y.; Wang, J. W.; Yang, Q. W.; Zhang, Z. G.; Yang, Y. W.; Ren, Q. L.; Alnemrat, S.; Bao, Z. B. A calcium-based microporous metal-organic framework for efficient adsorption separation of light hydrocarbons. *Chem Eng J* **2019**, *358*, 446–455, DOI: 10.1016/j.cej.2018.10.034.

(S180) Tu, R.; Zhang, W.; Zhang, J.; Wang, M.; Zhang, F.; Yang, K.; Li, J.; Pan, H.; Bernards, M. T.; Xie, P.; others Squarate-calcium metal–organic framework for molecular sieving of CO_2 from flue gas with high water vapor resistance. *Energy & Fuels* **2021**, *35*, 13900–13907, DOI: 10.1021/acs.energyfuels.1c01642.

(S181) Olson, D. H. The crystal structure of dehydrated NaX. *Zeolites* **1995**, *15*, 439–443, DOI: 10.1016/0144-2449(95)00029-6.

(S182) Vujić, B.; Lyubartsev, A. P. Transferable force-field for modelling of CO_2 , N_2, O_2 and Ar in all silica and Na^+ exchanged zeolites. *Modelling and Simulation in Materials Science and Engineering* **2016**, *24*, 045002, DOI: 10.1088/0965-0393/24/4/045002.

(S183) Joos, L.; Swisher, J. A.; Smit, B. Molecular Simulation Study of the Competitive Adsorption of H_2O and CO_2 in Zeolite 13X. *Langmuir* **2013**, *29*, 15936–15942, DOI: 10.1021/la403824g.

(S184) McEwen, J.; Hayman, J.-D.; Ozgur Yazaydin, A. A comparative study of CO_2 ,CH_4 and N_2 adsorption in ZIF-8, Zeolite-13X and BPL activated carbon. *Chemical Physics* **2013**, *412*, 72–76, DOI: 10.1016/j.chemphys.2012.12.012.

(S185) Najafi, A. M.; Soltanali, S.; Khorashe, F.; Ghassabzadeh, H. Effect of binder on CO_2 , CH_4, and N_2 adsorption behavior, structural properties, and diffu-

sion coefficients on extruded zeolite 13X. *Chemosphere* **2023**, *324*, 138275, DOI: 10.1016/j.chemosphere.2023.138275.

(S186) Cavenati, S.; Grande, C. A.; Rodrigues, A. E. Adsorption Equilibrium of Methane, Carbon Dioxide, and Nitrogen on Zeolite 13X at High Pressures. *Journal of Chemical & Engineering Data* **2004**, *49*, 1095–1101, DOI: 10.1021/je0498917.

(S187) Wilkins, N. S.; Rajendran, A. Measurement of competitive CO_2 and N_2 adsorption on Zeolite 13X for post-combustion CO_2 capture. *Adsorption* **2019**, *25*, 115–133, DOI: 10.1007/s10450-018-00004-2.

(S188) Wang, Y.; LeVan, M. D. Adsorption Equilibrium of Carbon Dioxide and Water Vapor on Zeolites 5A and 13X and Silica Gel: Pure Components. *Journal of Chemical & Engineering Data* **2009**, *54*, 2839–2844, DOI: 10.1021/je800900a.

(S189) Rudenko, A. N.; Bendt, S.; Keil, F. J. Multiscale Modeling of Water in Mg-MOF-74: From Electronic Structure Calculations to Adsorption Isotherms. *J Phys Chem C* **2014**, *118*, 16218–16227, DOI: 10.1021/jp503778m.

(S190) Burtch, N. C.; Jasuja, H.; Walton, K. S. Water Stability and Adsorption in Metal-Organic Frameworks. *Chem Rev* **2014**, *114*, 10575–10612, DOI: 10.1021/cr5002589.

(S191) Xu, W. T.; Yaghi, O. M. Metal-Organic Frameworks for Water Harvesting from Air, Anywhere, Anytime. *Acs Central Sci* **2020**, *6*, 1348–1354, DOI: 10.1021/acscentsci.0c00678.

(S192) Ongari, D.; Talirz, L.; Jablonka, K. M.; Siderius, D. W.; Smit, B. Data-Driven Matching of Experimental Crystal Structures and Gas Adsorption Isotherms of Metal-Organic Frameworks. *J. Chem. Eng. Data* **2022**, *67*, 1743–1756, DOI: 10.1021/acs.jced.1c00958.

(S193) Park, J.; Howe, J. D.; Sholl, D. S. How Reproducible Are Isotherm Measurements in Metal-Organic Frameworks? *Chem Mater* **2017**, *29*, 10487–10495, DOI: 10.1021/acs.chemmater.7b04287.

(S194) Datar, A.; Witman, M.; Lin, L. C. Monte Carlo simulations for water adsorption in porous materials: Best practices and new insights. *Aiche J* **2021**, *67*, DOI: ARTNe1744710.1002/aic.17447.

(S195) Vega, C.; Abascal, J. L. F.; Nezbeda, I. Vapor-liquid equilibria from the triple point up to the critical point for the new generation of TIP4P-like models: TIP4P/Ew, TIP4P/2005, and TIP4P/ice. *J Chem Phys* **2006**, *125*, DOI: 10.1063/1.2215612.

(S196) Roussanaly, S.; Berghout, N.; Fout, T.; Garcia, M.; Gardarsdottir, S.; Nazir, S. M.; Ramirez, A.; Rubin, E. S. Towards improved cost evaluation of Carbon Capture and Storage from industry. *International Journal of Greenhouse Gas Control* **2021**, *106*, 103263, DOI: 10.1016/J.IJGGC.2021.103263.

(S197) AECOM Next Generation Carbon Capture Technology. Techno-economic Methodology Report Work Package 5. Department for Business, Energy and Industrial Strategy. *International Journal of Greenhouse Gas Control* **2022**, *60666122-WP5-RP-001*.

(S198) Rubin, E. S. Understanding the pitfalls of CCS cost estimates. *International Journal of Greenhouse Gas Control* **2012**, *10*, 181–190, DOI: 10.1016/j.ijggc.2012.06.004.

(S199) Department for Business, Energy & Industrial Strategy Electricity Generation Costs 2020. 2020; https://assets.publishing.service.gov.uk/government/uploads/system/uploads/attachment_data/file/911817/electricity-generation-cost-report-2020.pdf.

(S200) Intergovernmental Panel on Climate Change *Climate Change 2013 – The Physical Science Basis: Working Group I Contribution to the Fifth Assessment Report of the Intergovernmental Panel on Climate Change*; Cambridge University Press, 2013; DOI: 10.1017/CBO9781107415324.

(S201) *The Concise Encyclopedia of Statistics*; Springer New York: New York, NY, 2008; pp 502–505, DOI: 10.1007/978-0-387-32833-1_379.

(S202) Farmahini, A. H.; Krishnamurthy, S.; Friedrich, D.; Brandani, S.; Sarkisov, L. Performance-Based Screening of Porous Materials for Carbon Capture. *Chem Rev* **2021**, *121*, 10666–10741, DOI: 10.1021/acs.chemrev.0c01266.

(S203) Burns, T. D.; Pai, K. N.; Subraveti, S. G.; Collins, S. P.; Krykunov, M.; Rajendran, A.; Woo, T. K. Prediction of MOF Performance in Vacuum Swing Adsorption Systems for Postcombustion CO_2 Capture Based on Integrated Molecular Simulations, Process Optimizations, and Machine Learning Models. *Environ Sci Technol* **2020**, *54*, 4536–4544, DOI: 10.1021/acs.est.9b07407.

(S204) Subraveti, S. G.; Roussanaly, S.; Anantharaman, R.; Riboldi, L.; Rajendran, A. How much can novel solid sorbents reduce the cost of post-combustion CO_2 capture? A techno-economic investigation on the cost limits of pressure-vacuum swing adsorption. *Appl Energ* **2022**, *306*, DOI: 10.1016/j.apenergy.2021.117955.

(S205) Zuettel, A.; Gallandat, N.; Dyson, P. J.; Schlapbach, L.; Gilgen, P. W.; Orimo, S. I. Future Swiss Energy Economy: The Challenge of Storing Renewable Energy. *Front Energy Res* **2022**, *9*, DOI: 10.3389/fenrg.2021.785908.

(S206) He, Y.; Xing, Y.; Zeng, X.; Ji, Y.; Hou, H.; Zhang, Y.; Zhu, Z. Factors influencing carbon emissions from China's electricity industry: Analysis using the combination of LMDI and K-means clustering. *Environmental Impact Assessment Review* **2022**, *93*, 106724, DOI: 10.1016/j.eiar.2021.106724.

(S207) Pieper, H.; Ommen, T.; Buhler, F.; Paaske, B. L.; Elmegaard, B.; Markussen, W. B. Allocation of investment costs for large-scale heat pumps supplying district heating. *Energy Procedia* **2018**, *147*, 358–367, DOI: 10.1016/j.egypro.2018.07.104.

(S208) Jesper, M.; Schlosser, F.; Pag, F.; Walmsley, T. G.; Schmitt, B.; Vajen, K. Large-scale heat pumps: Uptake and performance modelling of market-available devices. *Renewable and Sustainable Energy Reviews* **2021**, *137*, 110646, DOI: 10.1016/j.rser.2020.110646.

(S209) Solutions, A. C. C. Aker Solutions Awarded Contract for the Brevik Carbon Capture Project. 2020; https://www.akersolutions.com/news/news-archive/2020/aker-solutions-awarded-contract-for-the-brevik-carbon-capture-project/.

(S210) Arshi, P. S.; Vahidi, E.; Zhao, F. Behind the scenes of clean energy: the environmental footprint of rare earth products. *ACS Sustainable Chemistry & Engineering* **2018**, *6*, 3311–3320, DOI: 10.1021/acssuschemeng.7b03484.

(S211) Balogun, H. A.; Bahamon, D.; AlMenhali, S.; Vega, L. F.; Alhajaj, A. Are we missing something when evaluating adsorbents for CO capture at the system level? *Energ Environ Sci* **2021**, *14*, 6360–6380, DOI: 10.1039/d1ee01677f.

(S212) Fernandez, M.; Trefiak, N. R.; Woo, T. K. Atomic property weighted radial distribution functions descriptors of metal–organic frameworks for the prediction of gas uptake capacity. *J. Phys. Chem. C Nanomater. Interfaces* **2013**, *117*, 14095–14105, DOI: 10.1021/jp404287t.

(S213) Janet, J. P.; Kulik, H. J. Resolving Transition Metal Chemical Space: Feature Selection for Machine Learning and Structure–Property Relationships. *J. Phys. Chem. A* **2017**, *121*, 8939–8954, DOI: 10.1021/acs.jpca.7b08750.

(S214) Krishnapriyan, A. S.; Haranczyk, M.; Morozov, D. Topological Descriptors Help Predict Guest Adsorption in Nanoporous Materials. *J. Phys. Chem. C* **2020**, *124*, 9360–9368, DOI: 10.1021/acs.jpcc.0c01167.

(S215) Krishnapriyan, A. S.; Montoya, J.; Haranczyk, M.; Hummelshøj, J.; Morozov, D. Machine learning with persistent homology and chemical word embeddings improves prediction accuracy and interpretability in metal-organic frameworks. *Sci. Rep.* **2021**, *11*, 8888, DOI: 10.1038/s41598-021-88027-8.

(S216) Chen, T.; Guestrin, C. XGBoost: A Scalable Tree Boosting System. Proceedings of the 22nd ACM SIGKDD International Conference on Knowledge Discovery and Data Mining, San Francisco, CA, USA, August 13-17, 2016. 2016; pp 785–794, DOI: 10.1145/2939672.2939785.

(S217) Bergstra, J.; Bardenet, R.; Bengio, Y.; Kégl, B. Algorithms for hyper-parameter optimization. 2011; https://proceedings.neurips.cc/paper_files/paper/2011/file/86e8f7ab32cfd12577bc2619bc635690-Paper.pdf.

(S218) Akiba, T.; Sano, S.; Yanase, T.; Ohta, T.; Koyama, M. Optuna: A Next-generation Hyperparameter Optimization Framework. Proceedings of the 25rd ACM SIGKDD International Conference on Knowledge Discovery and Data Mining. 2019; DOI: 10.1145/3292500.3330701.

(S219) Perea, J. A.; Munch, E.; Khasawneh, F. A. Approximating continuous functions on persistence diagrams using template functions. *Found. Comput. Math.* **2022**, 1–58, DOI: 10.1007/s10208-022-09567-7.

(S220) Lundberg, S. M.; Lee, S.-I. A Unified Approach to Interpreting Model Predictions. 2017; http://papers.nips.cc/paper/7062-a-unified-approach-to-interpreting-model-predictions.pdf.

(S221) Kim, B.; Khanna, R.; Koyejo, O. Examples Are Not Enough, Learn to Criticize! Criticism for Interpretability. 2016; https://proceedings.neurips.cc/paper_files/paper/2016/file/5680522b8e2bb01943234bce7bf84534-Paper.pdf.

(S222) Son, K. N.; Richardson, T.-M. J.; Cmarik, G. E. Equilibrium Adsorption Isotherms forH_2Oon Zeolite 13X. *Journal of Chemical & Engineering Data* **2019**, *64*, 1063–1071, DOI: 10.1021/acs.jced.8b00961.

(S223) Ruthven, D. M.; Farooq, S.; Knaebel, K. S. *Pressure swing adsorption*; John Wiley & Sons, 1996.

(S224) Danckwerts, P. Continuous flow systems: Distribution of residence times. *Chemical Engineering Science* **1953**, *2*, 1–13, DOI: https://doi.org/10.1016/0009-2509(53)80001-1.

(S225) Son, K. N.; Weibel, J. A.; Knox, J. C.; Garimella, S. V. Limitations of the Axially Dispersed Plug-Flow Model in Predicting Breakthrough in Confined Geometries. *Industrial & Engineering Chemistry Research* **2019**, *58*, 3853–3866, DOI: 10.1021/acs.iecr.8b05925.

(S226) Tamura, T. Oxygen concentration process. US Patent US3533221A, 1970; https://patents.google.com/patent/US3533221A/en?oq=US3533221A, Accessed on: December 12, 2023.

(S227) Yearout, J. D. Gas-treating process and system. US Patent US3594983A, 1971; https://patents.google.com/patent/US3594983A/en?oq=US3594983A, Accessed on: December 12, 2023.

(S228) Sircar, S.; Kratz, W. C. Removal of water and carbon dioxide from air. US Patent US4249915A, 1981; https://patents.google.com/patent/US4249915A/en?oq=US4249915A, Accessed on: December 12, 2023.

(S229) Ravi Kumar, S. S.; Kratz, W. C. Adsorptive process for the removal of carbon dioxide from a gas. US Patent US4472178A, 1983; https://patents.google.com/patent/US4472178A/en?oq=US4472178A, Accessed on: December 12, 2023.

(S230) Sharma, P. K.; Wankat, P. C. Hybrid Cycles to Purify Concentrated Feeds Containing a Strongly Adsorbed Impurity with a Nonlinear Isotherm: The PSA-TSA Supercycle. *Industrial & Engineering Chemistry Research* **2009**, *48*, 6405–6416, DOI: 10.1021/ie801661w.

(S231) Lin, L. C.; Lee, K.; Gagliardi, L.; Neaton, J. B.; Smit, B. Force-Field Development from Electronic Structure Calculations with Periodic Boundary Conditions: Applications to Gaseous Adsorption and Transport in Metal-Organic Frameworks. *J. Chem. Theory Comput.* **2014**, *10*, 1477–1488, DOI: 10.1021/ct500094w.

(S232) Byrd, R. H.; Nocedal, J.; Waltz, R. A. *Nonconvex Optimization and Its Applications*; Springer US, 2006; pp 35–59, DOI: 10.1007/0-387-30065-1_4.

(S233) Boggs, P. T.; Tolle, J. W. Sequential quadratic programming. *Acta numerica* **1995**, *4*, 1–51.

(S234) Huck, J. M.; Lin, L.-C.; Berger, A.; Shahrak, M. N.; Martin, R. L.; Bhown, A.; Haranczyk, M.; Reuter, K.; Smit, B. Evaluating different classes of porous materials for carbon capture. *Ener. Env. Sci.* **2014**, *7*, 4136–4146, DOI: 10.1039/C4EE02636E.

(S235) Prokhorenkova, L.; Gusev, G.; Vorobev, A.; Dorogush, A. V.; Gulin, A. CatBoost: Unbiased Boosting with Categorical Features. Proceedings of the 32nd International Conference on Neural Information Processing Systems. Red Hook, NY, USA, 2018; pp 6639–6649.

(S236) Malinin, A.; Prokhorenkova, L.; Ustimenko, A. Uncertainty in Gradient Boosting via Ensembles. http://arxiv.org/abs/2006.10562.

(S237) Cleeton, C.; Oliveira, F. L. d.; Neumann, R. F.; Farmahini, A. H.; Luan, B.; Steiner, M.; Sarkisov, L. A process-level perspective of the impact of molecular force fields on the computational screening of MOFs for carbon capture. DOI: 10.1039/D3EE00858D, Publisher: The Royal Society of Chemistry.

(S238) Mansouri, A.; Pougin, M. J.; Yurduşen, A.; Dovgaliuk, Y.; Nandi, S.; García-Díez, E.; Patriarche, G.; Garcia, S.; Smit, B.; Mouchaham, G.; Serre, C. A bimetallic aluminum-copper-carboxylate-pyrazolate based-MOF for post-combustion CO_2 capture. *Submitted* **2023**,

(S239) Massiot, D.; Fayon, F.; Capron, M.; King, I.; Le Calvé, S.; Alonso, B.; Durand, J.-O.; Bujoli, B.; Gan, Z.; Hoatson, G. Modelling one- and two-dimensional solid-state NMR spectra. *Magn. Reson. Chem.* **2002**, *40*, 70–76, DOI: 10.1002/mrc.984.

(S240) Malek, A.; Farooq, S.; Rathor, M.; Hidajat, K. Effect of velocity variation due to adsorption-desorption on equilibrium data from breakthrough experiments. *Chemical Engineering Science* **1995**, *50*, 737–740, DOI: https://doi.org/10.1016/0009-2509(94)00245-M.

(S241) Goyal, P.; Purdue, M. J.; Farooq, S. Adsorption and Diffusion of N_2 and CO_2 and Their Mixture on Silica Gel. *Industrial & Engineering Chemistry Research* **2019**, *58*, 19611–19622, DOI: 10.1021/acs.iecr.9b02685.
